# Maternal Obesity Induces the Meiotic Defects and Epigenetic Alterations During Fetal Oocyte Development

**DOI:** 10.1002/advs.202309184

**Published:** 2024-06-13

**Authors:** Shoubin Tang, Huihua Wu, Qiuzhen Chen, Tao Tang, Jiashuo Li, Huiqing An, Shuai Zhu, Longsen Han, Hongzheng Sun, Juan Ge, Xu Qian, Xi Wang, Qiang Wang

**Affiliations:** ^1^ State Key Laboratory of Reproductive Medicine and Offspring Health Changzhou Maternity and Child Health Care Hospital Changzhou Medical Center Nanjing Medical University Nanjing 211166 China; ^2^ Department of Nutrition and Food Hygiene School of Public Health Nanjing Medical University Nanjing 211166 China; ^3^ Suzhou Municipal Hospital Nanjing Medical University Nanjing 211166 China

**Keywords:** epigenetics, meiosis, obesity, oocyte, time‐restricted feeding

## Abstract

It has been widely reported that obesity adversely impacts reproductive performance of females. However, the effects of maternal obesity on fetal germ cells remain poorly understood. In the present study, by employing a high‐fat diet (HFD)‐based mouse model, it is discovered that maternal obesity disrupts the chromosomal synapsis and homologous recombination during fetal oogenesis. Moreover, transcriptomic profiling reveales the potential molecular network controlling this process. Of note, the global hypermethylation of genomic DNA in fetal oocytes from obese mouse is detected. Importantly, time‐restricted feeding (TRF) of obese mice not only ameliorate the meiotic defects, but also partly restore the epigenetic remodeling in fetal oocytes. In sum, the evidence are provided showing the deficit fetal oogenesis in obese mother, implicating a mechanism underlying the intergenerational effects of environmental insults. TRF may represent a potentially effective approach for mitigating fertility issues in obese patients.

## Graphical abstract

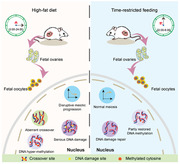

## 1 Introduction

The process of oogenesis starts in the fetal ovaries with the development of oogonia from primordial germ cells (PGCs). Mouse PGCs emerge at embryonic day (E)7.25 at the base of the allantois and migrate by a well‐defined route into the genital ridges.^[^ ^1^ ^]^ At E13.5, female PGCs are entering into meiosis, as they, now termed primary oocytes, begin to condense chromatin into chromosomes and assembly the synaptonemal complex to mediate homologous chromosome alignment, synapsis, and recombination. Recombination is initiated by programmed DNA double‐strand breaks (DSBs) which are generated by SPO11 (SPO11 initiator of meiotic double stranded breaks). These meiotic DSBs recruit a series of recombination proteins, forming recombination foci and facilitating the synapsis of homologous chromosomes during the zygotene stage.^[^ ^2^ ^]^ The subsequent invasion of the homolog's duplex by the 3′ single‐stranded DNA is mediated by recombinases DNA meiotic recombinase 1 and RAD51 (Radiation sensitive protein 51).^[^ ^3^, ^4^ ^]^ The maturation of recombination foci continues while chromosomes achieve full synapsis during the pachytene stage, ultimately leading to either crossover or non‐crossover events.^[^ ^2^ ^]^ During this process, PGCs and primary oocytes undergo a series of coordinated epigenetic reprogramming events, including global DNA methylation erasure.^[^ ^5^, ^6^ ^]^ The average DNA methylation level drops from 77.7% in the E6.5 epiblast to 5.0% in E11.5 PGCs, further decreasing to 3.8% in E13.5 female PGCs, and this highly hypomethylated state persists in E16.5 oocytes (5.6%).^[^ ^7^ ^]^ Epigenetic modifications of germ cells are highly sensitive to environmental exposures, specifically the parental nutrition.^[^ ^8^, ^9^, ^10^ ^]^

Although it is becoming increasingly evident that maternal obesity has permanent effects on a range of physiological processes in the offspring, scant information is available about the consequence of such condition for oogenesis in mammals. Our research and others have shown that exposure to maternal obesity before and during pregnancy leads to delayed early embryo development and fetal growth retardation in mice.^[^ ^8^, ^11^, ^12^ ^]^ It has been suggested that these adverse effects stem from factors within the oocyte rather than the uterine environment.^[^ ^13^, ^14^ ^]^ Emerging evidence indicates that meiotic defects, mitochondrial dysfunction, and abnormal genome methylation in oocytes may contribute to the offspring phenotypes.^[^ ^15^, ^16^, ^17^, ^18^, ^19^, ^20^, ^21^, ^22^ ^]^ However, to date, the effects of maternal obesity on fetal oocytes have not been investigated. On the other hand, several interventions have been proposed to rejuvenate cells and organs, delay the onset of obesity‐related diseases, and extend health span, such as caloric restriction (CR), intermittent fasting (IF), and time‐restricted feeding (TRF).^[^ ^23^, ^24^, ^25^ ^]^ TRF restricts the timing of calorie intake without altering the total calorie consumption.^[^ ^26^, ^27^, ^28^ ^]^ Numerous studies have shown that TRF can prevent the obesity‐related phenotypic defects, including metabolic changes, inflammation and weight gain.^[^ ^29^, ^30^, ^31^, ^32^ ^]^

In this study, we discovered the abnormal meiotic recombination and incomplete genome methylation erasure in fetal oocytes from HFD‐exposed mice. Moreover, we found that TRF intervention is able to partly prevent these adverse effects of maternal obesity on fetal germ cells.

## 2 Results

### 2.1 Maternal Obesity Affects Meiotic Progression in Fetal Oocytes

**Figure 1:**
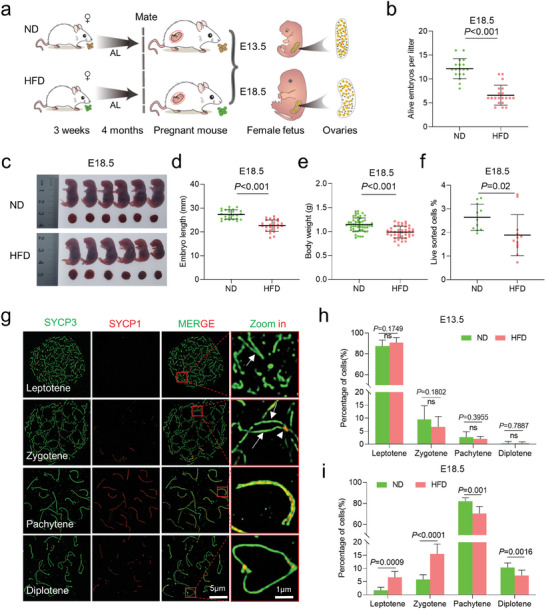
Maternal obesity impairs fetal development. a) Diagram illustrating the high‐fat diet (HFD) model in ICR female mice. Normal diet (ND) mice were provided with a standard diet and HFD with high‐fat diet. Mice were subjected to either ND or HFD feeding for a duration of 4 months, starting at 3 weeks of age. Subsequently, they were mated with ND males, and the fetuses were harvested at embryonic days 13.5 (E13.5) and 18.5 (E18.5). b–e), Maternal obesity exerts adverse effects on embryonic development at E18.5, as evidenced by morphological evaluations of the embryos. Fetal development was assessed by measuring the number of alive fetuses b) ND = 18 and HFD = 20 litters at E18.5), crown–rump length c,d) *n* = 25 for ND and *n* = 26 for HFD) and body weight e) *n* = 55 for ND and *n* = 42 for HFD) in live embryos. f) Quantitative analysis of the number of SSEA‐1^+^ oocytes in each fetus at E18.5 (*n* = 11 for ND and *n* = 12 for HFD). g) Immunofluorescence staining of SYCP1 (red) and SYCP3 (green) in oocyte cytospreads, as observed by SIM. Oocytes were obtained from the ovaries of female fetuses at E13.5 and E18.5. Magnified views highlight the presence of SYCP1 and SYCP3. h,i) Distribution of meiotic stages in oocytes from E13.5 (1065 ND and 1173 HFD cells across ten biological replicates.) and E18.5 (1170 ND and 1245 HFD cells across 10 biological replicates.) fetuses in the ND and HFD groups. Data are presented as mean ± SD. Student's *t*‐test (two‐tailed) was employed for statistical analysis. Significance was set at P‐value <0.05.

While previous studies have documented the impact of maternal obesity on the growth of fetus,^[^ ^16^, ^18^ ^]^ however, the comprehensive assessment of fetal oocyte development was lacking. Female mice were continuously fed either a high‐fat diet (HFD) or a normal diet (ND) for 4 months starting at 3 weeks of age (**Figure** **1**a), as we described before.^[^ ^8^ ^]^ Expectedly, female mice fed the HFD became obese (Figure S1a, Supporting Information), displaying impaired glucose tolerance and insulin resistance (Figure S1b–f, Supporting Information). Then these mice were mated with normal males, and fetuses were harvested for further evaluation (Figure 1a). Significant developmental failure and growth retardation were observed in E18.5 fetuses from HFD mice, as evidenced by the number of live pups, resorptions per litter (Figure 1b; Figure S2e, Supporting Information), and fetal length/weight (Figure 1c–e). Similar results were observed in embryos at E13.5 (Figure S2a–d,f, Supporting Information), indicating that the development of fetuses in HFD mice is already compromised at this early stage.

The evident fetal growth restriction prompted us to investigate whether fetal oocyte development was impaired. Oocytes from ND and HFD female fetuses were isolated from single E13.5 and E18.5 embryos, respectively, using fluorescence‐activated cell sorting (FACS) and SSEA‐1 antibody. A marked reduction in the number of oocytes was observed in E18.5 HFD fetuses compared to ND fetuses (Figure 1f), but no differences were found at E13.5 (Figure S2g, Supporting Information). The numbers of gonad somatic cells were also comparable between E13.5 and E18.5 (Figure S2h,i, Supporting Information). The results suggest that maternal obesity seems to have little effect on gonad development, but rather disturbs the oogenesis.

To determine whether meiotic progression was disrupted in HFD fetal oocytes, we examined the chromosome behavior during meiotic prophase I using high‐resolution structured illumination microscopy (SIM) (Figure 1g). The synaptonemal complex comprises two axial elements (AEs) and a central element (CE), connected by transversal filaments (TEs). AEs labeled by SYCP3 appear as two separate strands (arrows, green signal), while SYCP1 forms homodimers located in the CE (arrowheads, red signal). AEs are fully formed at zygotene stage, and oocytes achieved synapsis on autosomes at pachytene stage, as evidenced by the continuous SYCP1 signals. The proportions of oocytes at different meiotic stages were analyzed at E13.5 and E18.5, respectively (Figure 1h,i). The results indicated that meiotic prophase I progression was not affected in E13.5 oocytes (Figure 1h). However, in E18.5 HFD fetal oocytes, there was a significant increase in the proportion of oocytes at leptotene and zygotene stages and a reduction in pachytene and diplotene stages compared to ND oocytes (Figure 1i). Based on these data, we conclude that maternal obesity impairs fetal development and disturbs meiotic progression in fetal oocytes. The delay in meiosis prophase I may contribute to the reduced oocyte numbers observed in HFD fetal oocytes.

### 2.2 Maternal Obesity Disrupts Chromosomal Synapsis in Fetal Oocytes

**Figure 2:**
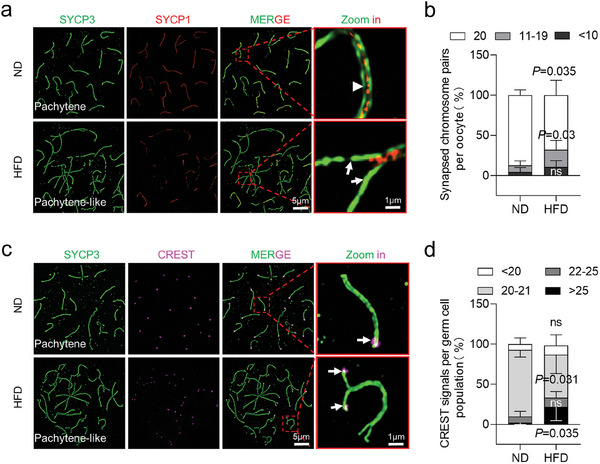
Disrupted chromosomal synapsis in HFD fetal oocytes. a) Chromosome spreads of oocytes from E18.5 ND and HFD fetuses were immunostained for SYCP3 (green) and SYCP1 (red) at pachytene. Arrows indicate synapsed chromosomes, while arrowheads indicate a single chromosome. Magnified views of the synapsed region reveal that SYCP1 was localized in the central region of SCs in a continuous or discontinuous pattern in ND and HFD oocytes. b) The percentage of synapsed chromosome pairs in ND and HFD fetal oocytes. The percentages were determined by counting 180 ND cells across six biological replicates and 210 HFD cells across seven biological replicates. c) Chromosome spreads of oocytes from E18.5 ND and HFD fetuses were immunostained for SYCP3 (green) and CREST (purple) at pachytene. Arrowheads indicate CREST signals. d) CREST staining reveals unpaired chromosomes in HFD E18.5 female oocytes. CREST foci were counted in 180 ND and 168 HFD cells across 5 biological replicates. Data are presented as mean ± SD. Student's *t*‐test (two‐tailed) was employed for statistical analysis. Significance was set at P‐value <0.05.

To determine the potential reason for the disturbed meiosis in HFD fetal oocytes, we assessed the synaptonemal complex structures in pachytene oocytes. In ND fetal oocytes, synapsis was initiated in some chromosomes, and SYCP1 was found along the SYCP3 axis in CE on fully synapsed chromosomes. Notably, SYCP1 signals were absent in HFD fetal oocytes at pachytene stage. There was always a large number of autosomes that remained unsynapsed (**Figure** **2**a, arrows) in comparison to the synapsed chromosomes (Figure 2a, arrowhead). These oocytes are therefore called “pachytene‐like cell” in this paper (defined by more than five synapsed chromosome pairs per cell). Statistical analysis revealed a reduction in the numbers of fully synapsed chromosomes in fetal pachytene oocytes from HFD mother (Figure 2b). Additionally, we used CREST antibodies to label centromeric loci and counted their numbers (Figure 2c, arrows). A significant increase was observed in HFD fetal oocytes, with more than 25 per cell (Figure 2d). Together, we conclude that most autosomes in HFD fetal oocytes failed to complete synapsis.

### 2.3 Impaired Meiotic Recombination in HFD Fetal Oocytes

**Figure 3:**
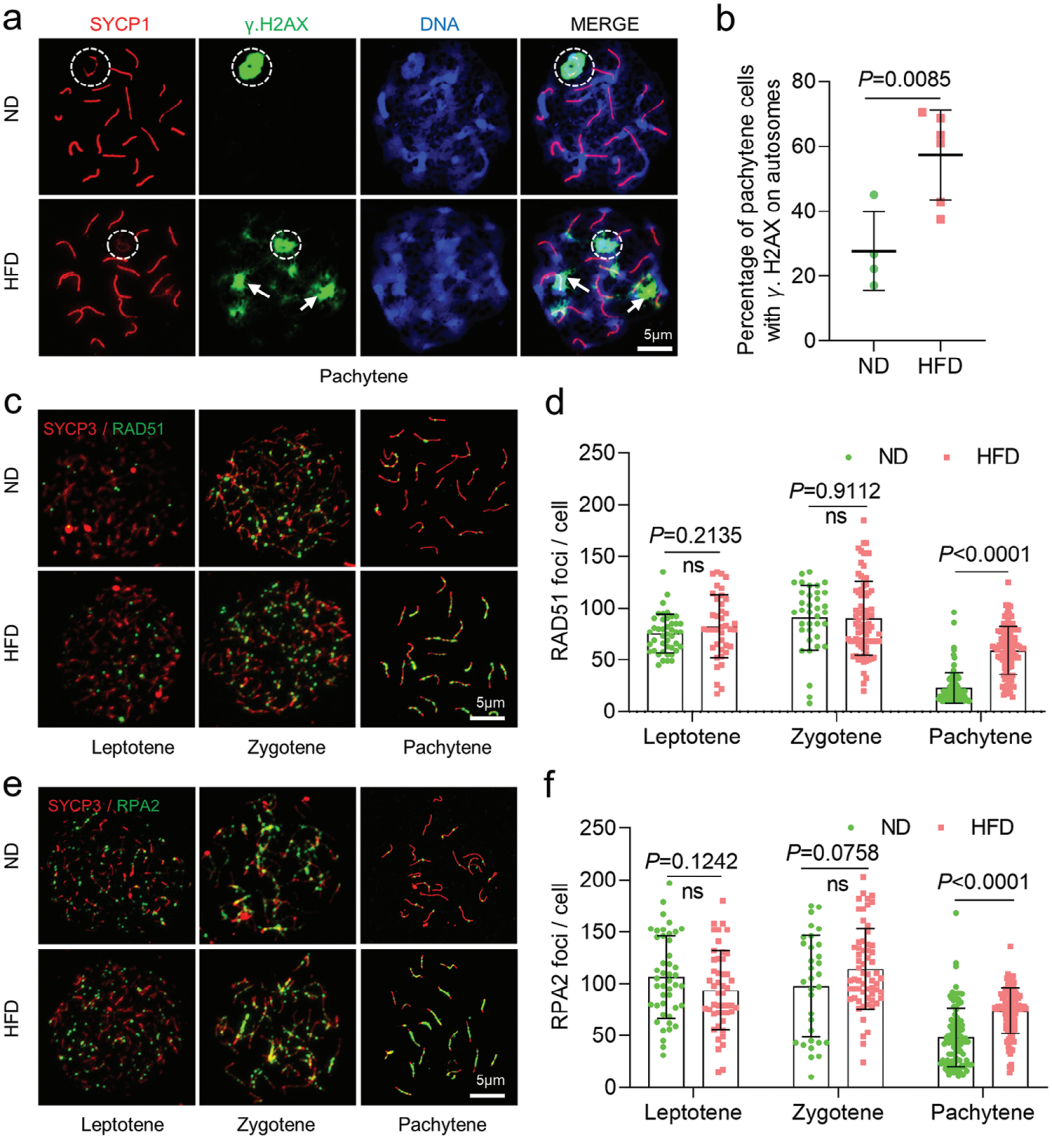
Impact of obesity on DSB formation and repair in fetal oocytes. a) Chromosome spreads of fetal oocytes from E18.5 ND and HFD mice were immunostained for SYCP1 (red), γ.H2AX (green), and counterstained with DAPI (blue) for nuclei at pachytene. A white circle indicates the XY body, and arrows indicate γ.H2AX signals within autosomes. b) Percentage of pachytene oocytes containing γ.H2AX foci on autosomes. *n* = 103 for ND cells across 4 biological replicates and *n* = 177 for HFD cells across 6 biological replicates. c) Immunostaining for RAD51 (green) and SYCP3 (red) was performed on ND and HFD oocytes from E18.5 female fetuses. Representative images of oocytes at the leptotene, zygotene, and pachytene stages are shown. d) Graphs depict the quantification of RAD51 foci numbers per cell at the leptotene, zygotene, and pachytene stages. Each dot represents the number of DNA repair protein foci per cell. *n*  =  176 biologically independent oocytes for ND; *n*  =  220 biologically independent oocytes for HFD. e) Immunostaining for RPA2 (green) and SYCP3 (red) was performed on ND and HFD oocytes from E18.5 female fetuses. Representative images of oocytes at the leptotene, zygotene, and pachytene stages are shown. f) Graphs show quantification of RPA2 foci numbers per cell at leptotene, zygotene, and pachytene stages. *n*  =  177 biologically independent oocytes for ND; *n*  =  210 biologically independent oocytes for HFD. Data are presented as means ± SD, significance was set at P‐value <0.05, and “n.s.” indicates no statistical significance.

Next, we evaluated meiotic DSB repair by analyzing the localization of phosphorylated H2AX (γ.H2AX), a marker for DNA double‐strand breaks. γ.H2AX marks unrepaired DNA lesions and the sex body in pachytene cells (**Figure** **3**a). In ND fetal oocytes, γ.H2AX signal was only observed at the XY bodies (Figure 3a, white circle, green signal), indicating the completion of autosomal recombination. In contrast, HFD fetal oocytes showed the persistent γ.H2AX signals on the autosomes (Figure 3a, arrows, Figure 3b), implying a failure in the repair of DSBs. To more specifically assess meiotic DSB repair and recombination, we conducted immunostaining for two markers, RAD51 recombinase (Figure 3c) and replication protein A2 (RPA2) (Figure 3e). As shown in Figure 3c–f, maternal obesity did not alter quantity of RAD51 and RPA2 foci in leptotene and zygotene oocytes. However, their numbers were markedly elevated in HFD fetal oocytes at pachytene stage, reflecting the defective meiotic recombination.

Successful mismatch repair is critical for DNA recombination and crossover formation during meiotic prophase I. Therefore, we further examined the quantity and location of potential crossover sites at pachytene stage using MLH1 (MutL protein homolog 1) as a marker (Figure S3a, Supporting Information). A significant increase in crossover numbers was observed in HFD compared to ND fetal oocytes (Figure S3a,b, Supporting Information). Similarly, more chromosomes with two MLH1 sites were also found in HFD fetal oocytes (Figure S3a,c, arrows, Supporting Information). These results indicate that the defects in meiotic prophase I in HFD fetal oocytes are likely due to the alterations in homologous recombination.

### 2.4 Identification of Target Genes of Obesity in HFD Fetal Oocytes

**Figure 4:**
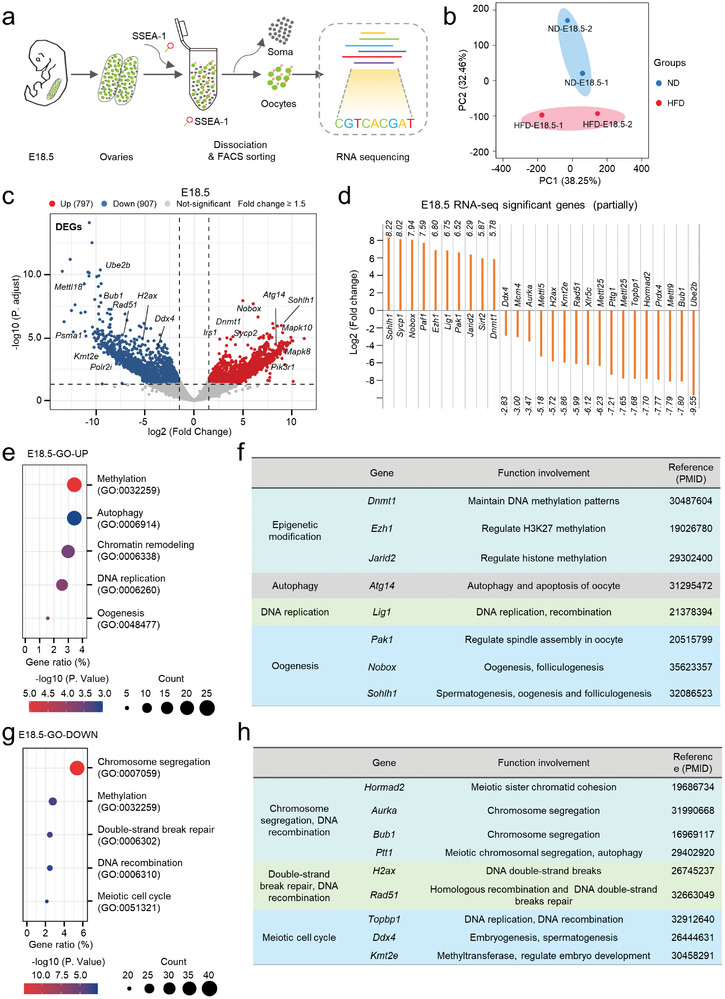
Profiling gene expression of fetal oocytes with RNA sequencing. a) Flowchart overview of fetal oocyte RNA‐seq. Fetal oocytes were collected from E18.5 ND and HFD female mice (300 oocytes from 10 fetuses per sample, two samples for each group). b) Volcano plot depicting differentially expressed genes (DEGs) in HFD fetal oocytes at E18.5 compared to ND ones (downregulated genes in blue, upregulated genes in red). c) Expression of select differentially expressed genes in HFD E18.5 fetal oocytes as measured by RNA‐seq. d,f) GO enrichment analysis results for upregulated and downregulated DEGs in HFD fetal oocytes compared to ND ones. e,g) Function and reference display of differentially expressed genes.

To further dissect the underlying mechanisms mediating the effects of maternal obesity on fetal germ cells, we performed RNA‐Seq analysis of oocytes from E18.5 fetuses (**Figure** **4**a). Principal component analysis (PCA) clearly showed that ND and HFD fetal oocytes at E18.5 were clustered into two groups (Figure 4b). Transcriptome profiles revealed that 907 differentially expressed genes (DEGs) were downregulated and 797 DEGs were upregulated in HFD fetal oocytes (Figure 4c,d; Table S1, Supporting Information). In these DEGs, we found a significant enrichment in oogenesis, DNA recombination, and epigenetic modifications (Figure 4e). For example, gene ontology (GO) analysis revealed the upregulated genes associated with the autophagy (e.g., *Atg14*), DNA methylation (e.g., *Dnmt1, Ezh1*, and *Jarid2*), oogenesis (e.g., *Pak1, Nobox*, and *Sohlh1*), and DNA replication (e.g., *Lig1*) (Figure 4e,f). By contrast, the majority of downregulated genes are enriched in chromosome segregation (e.g., *Hormad2*, *Aurka*, and *Bub1*), histone methylation (e.g., *Mettl15* and *Kmt2e*), double‐strand break repair (e.g., *H2ax*, *Rad51*), and meiotic cell cycle (e.g., *Ddx4* and *Topbp1*) in HFD fetal oocytes (Figure 4g,h). Collectively, these results indicate that epigenetic modifications and genetic stability are evidently altered in fetal oocytes exposed to maternal obesity.

### 2.5 Global Hypermethylation Across the Genome in HFD Fetal Oocytes

**Figure 5:**
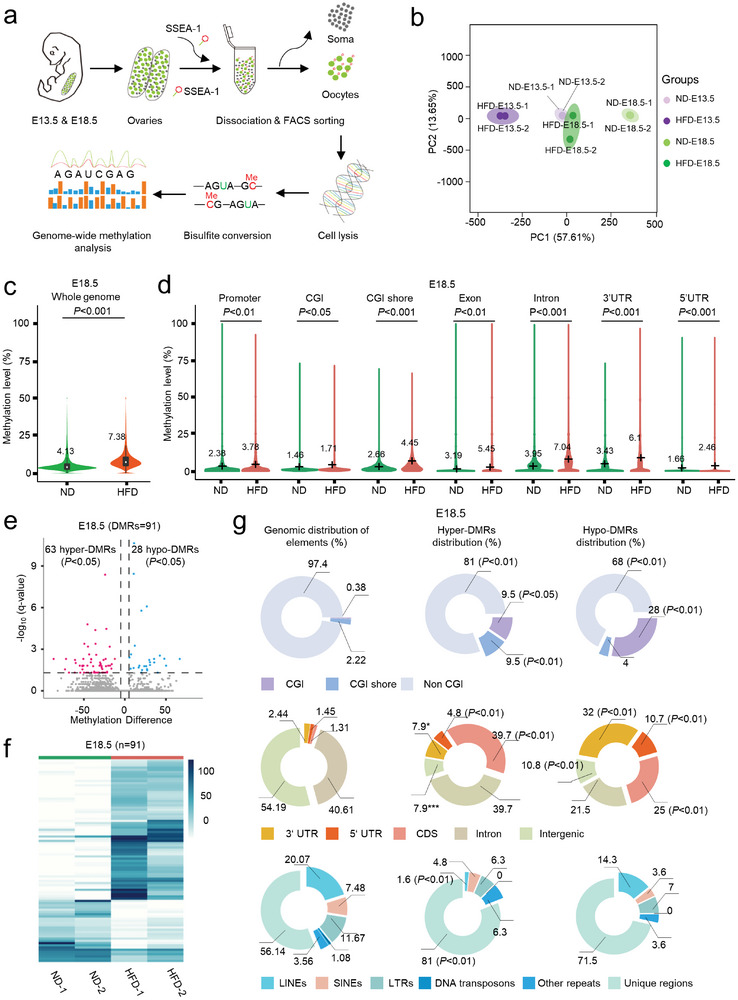
Global DNA hypermethylation across various genomic features in fetal oocytes from HFD mice. a) Flowchart illustrating the BS‐seq procedure for genome‐wide methylation analysis, involving the collection of fetal oocytes, bisulfite conversion of DNA, and library preparation for high‐throughput sequencing. Fetal oocytes were collected from E18.5 ND and HFD female mice (300 oocytes from 10 fetuses per sample, two samples for each group). b) Average methylation levels in the genome of fetal oocytes at E18.5. c) Violin plots displaying methylation levels for different genomic features, including promoters, CGIs, CGI shore, exon, intron, 3′UTR, and 5′UTR in fetal oocytes. The mean methylation levels are denoted by numerical values and black crosses. Statistical analyses were conducted using a bootstrap test. d) Volcano plot depicting the results of DNA methylation microarray analysis in fetal oocytes from ND and HFD backgrounds at E18.5. Dots positioned in the upper left corner represent significantly hypermethylated probes, while those in the upper right corner represent significantly hypomethylated probes with a statistical significance of *P* < 0.05 and a fold change >1.5. e) Heatmap visualization illustrating differentially methylated regions (DMRs) in ND and HFD fetal oocytes at E18.5, where each line represents a distinct DMR. High methylation levels are denoted in blue, while low methylation levels are depicted in white. f) The relative proportion of DMRs among CpG Islands (CGIs) and CGI shores (top), the relative proportion of DMRs among coding and noncoding regions (middle), and the relative proportion of DMRs among distinct sequence and repetitive elements throughout the genome (bottom). Fisher's exact test was employed to assess potential enrichment or depletion of DMRs relative to the genomic distribution of these elements: a008375 significance was set at P‐value <0.05.

In order to examine the effects of maternal obesity on the methylation patterns in fetal oocytes, base‐resolution methylomes were generated using the bisulfite sequencing (BS‐seq) method for small samples (**Figure** **5**a).^[^ ^33^ ^]^ Oocytes from ND and HFD mice were collected at E13.5 and E18.5, respectively. Different groups were well separated as evidenced by PCA (Figure 5b). Of note, HFD fetal oocytes showed a significant increase in global CpG methylation levels relative to ND fetal oocytes (Figure 5c; Figure S4a, Supporting Information). Methylome analysis also presented the hypermethylation across all of the genomic features examined in HFD fetal oocytes, including promoters, introns, exons, UTRs, and CpG islands (CGIs) and shores (Figure 5d; Figure S4b–h, Supporting Information), as well as the major repetitive elements (Figure S5, Tables S2 and S3, Supporting Information). In particular, the pronounced hypermethylation were mainly distributed in the gene body region and adjacent intergenic region at both E13.5 and E18.5. (Figure S6a,b, Supporting Information).

Next, we identified differentially methylated regions (DMRs) with statistical significance at a false discovery rate (FDR) <0.05 between ND and HFD fetal oocytes for gaining a deeper understanding of the altered methylation landscape (Table S4, Supporting Information). At E18.5, we found a total of 91 DMRs, with 63 being hypermethylated (hyper‐DMRs; 69.23%) and 28 hypomethylated (hypo‐DMRs; 30.77%) in HFD compared to ND groups (Figure 5e,f). At E13.5, we identified a total of 2650 DMRs, of which 2449 were hypermethylated (hyper‐DMRs; 92.42%) and 201 were hypomethylated (hypo‐DMRs; 7.58%) in HFD relative to ND fetal oocytes (Figure S6c,d, Supporting Information), indicating a prevalence of hyper‐DMRs. Furthermore, we examined the distribution of these DMRs within unique and repetitive genomic elements. At E18.5, hyper‐DMRs were enriched in CGIs, CGI shores, CDS, 3′UTRs, 5′UTRs, and unique regions, but were depleted in non‐CGI, LINEs and intergenic regions (Figure 5g). A similar distribution was also observed at E13.5 (Figure S6e, Supporting Information). This analysis underscores that DMRs are not randomly distributed in the genome of HFD fetal oocytes. Together, genome‐wide profiling clearly indicates that the methylation landscape in fetal oocytes is altered as a consequence of maternal obesity.

To further understand the potential biological function of these DMRs, we performed GO analysis of genes with DMRs using KOBAS 3.0.^[^ ^34^ ^]^ Results showed that the genes with DMRs at E13.5 were largely enriched in cell differentiation, metabolic process, and embryonic development (e.g., *Mef2b*, *Pygb*, and *Ddx4*) (Figure S7a,b, Supporting Information). Genes with DMRs at E18.5 were primarily enriched in ion transport and mitochondrial function (e.g., *Slc12a3*, *MT‐ND1*) (Figure S7c,d, Supporting Information).

### 2.6 Time‐Restricted Feeding Improves the Fetal Development of HFD Mice

**Figure 6:**
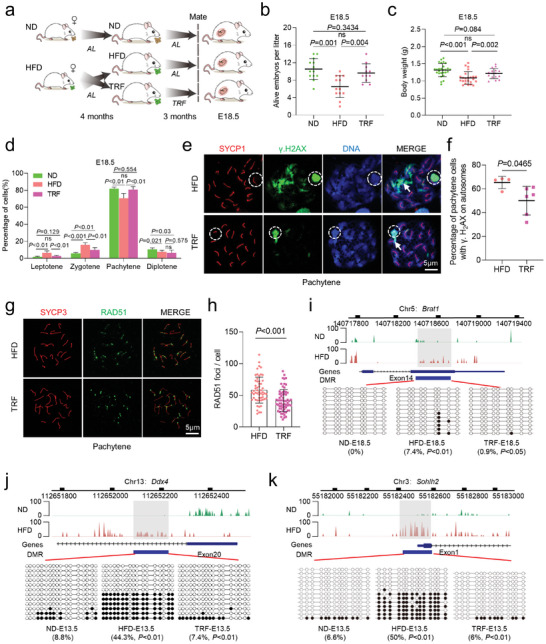
Effects of time‐restricted feeding on fetal oocytes. a) Schematic representation of the dietary conditions in female mice, including ND, HFD, and TRF. TRF mice were subjected to a high‐fat diet from 10 p.m. to 6 a.m. daily for three months. b,c) Assessment of fetal development, including the number of viable embryos (*n* = 13 for ND, *n* = 13 for HFD, and n = 11 for TRF) and their body weight (*n* = 27 for ND, *n* = 24 for HFD and *n* = 16 for TRF) at E18.5. d) Meiotic stage frequencies in fetal oocytes at E18.5 for ND (*n* = 569), HFD (*n* = 543), and TRF (*n* = 514) groups. Analysis included at least three mice per group. e) Immunostaining of fetal oocytes from E18.5 HFD and TRF mice for SYCP1 (red) and γ.H2AX (green), with nuclei counterstained using DAPI (blue) during pachytene. The white circle indicates the XY body, and arrows point to γ.H2AX signals within autosomes. f) Percentage of pachytene oocytes containing γ.H2AX foci on autosomes. *n* = 157 for HFD cells across four biological replicates and *n* = 182 for TRF cells across six biological replicates. g) Immunostaining for RAD51 (green) and SYCP3 (red) in oocytes from E18.5 female fetuses under HFD and TRF conditions. Representative images of oocytes at the pachytene stage are displayed. h) Quantification of RAD51 foci per cell at the pachytene stage. Each dot represents the number of DNA repair protein foci per cell. *n * =  55 biologically independent oocytes for HFD; *n * =  66 biologically independent oocytes for TRF. i–k) Graphical representation of the methylation patterns at the *Brat1*, *Ddx4*, and *Sohlh2* loci in fetal oocytes for ND, HFD, and TRF groups. The gray box highlights the selected region for further validation. Open and filled circles represent unmethylated and methylated CpGs, respectively, with the percentages of methylated CpGs displayed below each panel. Data are presented as mean ± SD. Student's *t*‐test (two‐tailed) was employed for statistical analysis. Significance was set at P‐value <0.05.

Given the promising performance of time‐restricted feeding (TRF) in the prevention of metabolic diseases,^[^ ^34^ ^]^ we administered TRF treatment to HFD mice to investigate whether it has beneficial effect on the pregnancy outcome of obese females (**Figure** **6**a). Food intake was measured at three‐day intervals for each group. There was no significant difference in caloric intake between HFD and TRF groups, confirming that the TRF model did not impose caloric restriction (Figure S9a, Supporting Information). However, TRF mice experienced a remarkable 30% reduction in their body weight (Figure S9b,c, Supporting Information). The weight of pgVAT (perigonadal visceral adipose tissue) and ingWAT (inguinal subcutaneous white adipose tissue) were also decreased in TRF group (Figure S9d, Supporting Information). Glucose tolerance and insulin resistance were accordingly improved in TRF group compared to HFD mice (Figure S9e–i, Supporting Information). Importantly, after mating, the conception rate of TRF females (Figure S10a, Supporting Information) and number of live embryos were markedly elevated (Figure 6b; Figure S10b–d, Supporting Information). Likewise, fetal body weights were higher in TRF mice at both E13.5 and E18.5 (Figure 6c; Figure S10e, Supporting Information), indicative of the improvement of fetal development. We then quantified the number of fetal oocytes by FACS, and no significant differences were observed between two groups (Figure S10f,g, Supporting Information). Nonetheless, the meiotic progression of HFD fetal oocytes (E18.5) was promoted by TRF (Figure 6d; Figure S10h, Supporting Information).

### 2.7 Time‐Restricted Feeding Alleviates the Defective Phenotypes of HFD Fetal Oocytes

To determine whether TRF could prevent DSBs in HFD fetal oocytes, we performed immunofluorescence staining at pachytene stage. The results revealed a marked reduction of γ.H2AX signals in the TRF fetal oocytes compared to HFD group (Figure 6e,f). Similarly, the number of RAD51 foci per cell was also decreased in TRF fetal oocytes (Figure 6g,h). These findings indicate that TRF treatment effectively mitigates DSBs in HFD fetal oocytes. Meanwhile, we assessed the impact of TRF on DNA methylation in fetal oocytes. In specific, we examined the methylation levels of genes such as *Ddx4* (DEAD‐box helicase 4), *Sohlh2* (spermatogenesis and oogenesis specific basic helix‐loop‐helix 2), *Kmt2b* (lysine methyltransferase 2B), *Kmt2d* (lysine methyltransferase 2D) at E13.5, and *Brat1* (BRCA1 associated ATM activator 1) and *USP30* (ubiquitin specific peptidase 30) at E18.5 through bisulfite‐pyrosequencing analysis. We confirmed that the selected regions had significantly more methylation in HFD fetal oocytes, consistent with the methylome data. Although the effects of TRF on the methylation state of these regions varied, the methylation levels of five (*Ddx4*, *Sohlh2*, *Kmt2d*, *Brat1*, and *USP30*) out of eight genes in HFD fetal oocytes were completely restored to normal (Figure 6i–k; Figure S11, Supporting Information). Nonetheless, TRF seemed unable to restore the abnormal methylation status of imprinted genes, such as *Mest* (mesoderm specific transcript) and *Igf2r* (insulin like growth factor 2 receptor). Collectively, these findings indicate that maternal‐obesity‐associated defective phenotypes in fetal oocytes can be partially alleviated through TRF intervention.

## 3 Discussion

**Figure 7:**
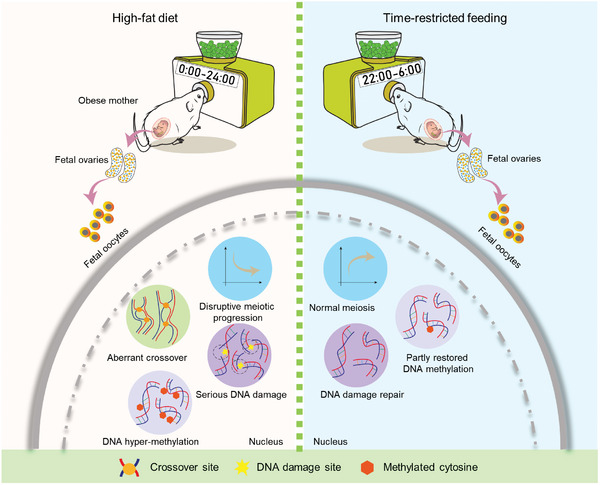
Diagram illustrating the effects of maternal obesity on fetal oocytes and TRF intervention. Maternal obesity induces the abnormal chromosome recombination and altered genome methylation in fetal oocytes. TRF intervention can partially enhance fetal oocyte development, and alleviate the meiotic and epigenetic defects in fetal oocytes from obese mice.

In the present study, we explored the impact of maternal obesity on fetal oocyte development. First, we conducted a detailed phenotypic assessment of fetus at E13.5 and E18.5 from HFD mice; Second, we discovered the disrupted chromosomal recombination in HFD fetal oocytes; Third, the significant effects of obesity on transcriptome and methylome landscape in fetal oocytes were uncovered. Finally, we showed that TRF not only improves metabolic state in HFD mother, but also has beneficial effects on the fetal oocyte development (**Figure** **7**).

Obesity poses a growing global threat, with an escalating prevalence of overweight and obese women of reproductive age. Extensive research has documented the connection between pre‐pregnancy and prenatal weight gain and fetal birth weight.^[^ ^35^, ^36^ ^]^ Utilizing HFD‐based mouse model, we and other researchers have found the developmental delay in early embryos and growth restrictions in fetuses born to obese mothers.^[^ ^15^, ^16^, ^18^ ^]^ Recently, studies using HFD mouse model in conjunction with in vitro fertilization (IVF) and embryo transfer experiments have unveiled that maternal diet induces defects in oocytes, thereby predisposing the offspring to metabolic diseases.^[^ ^13^ ^]^ Nevertheless, the exploration of the impact of maternal obesity on fetal oocytes and its underlying mechanisms remains an ongoing endeavor. Here, we observed the delayed progression through prophase I in HFD fetal oocytes, evidenced by the loss of SYCP1 signal (Figure 1). They are unable to complete DSBs repair during homologous chromosome synapsis (Figure 3). This slower meiotic prophase I likely contribute to oocyte depletion or loss^[^ ^37^ ^]^ and may be resulted from the reduced expression of developmental genes associated with meiotic progression (Figure 4). However, considering that the vast majority of oocytes formed in the fetal ovary do not survive beyond birth, so currently, we are not sure whether a decrease 25.9% in the number of fetal oocytes at E18.5 would result in diminished ovarian reserve after puberty. In addition, transcriptome data showed an upregulation of *Sycp1* mRNA in HFD fetal pachytene oocytes, which is inconsistent with the reduction in SYCP1 protein. Given the unique mechanism controlling mRNA accumulation and protein degradation in oogenesis, this phenomenon may reflect the differential regulation of transcription and post‐transcription in these cells.

Recombination is essential for generating genetic diversity in species, with DSBs produced during the leptotene stage giving rise to crossovers.^[^ ^38^, ^39^ ^]^ Only a small fraction of these DSBs eventually become crossovers, with the majority being repaired from zygotene to pachytene stage, except on the sex chromosomes.^[^ ^40^ ^]^ We found that in HFD fetal oocytes, recombinases RAD51 and RPA2 persist in unsynapsed regions and fail to be released, indicative of the deficient recombination (Figure 3c,e). In line with this, reduced transcript levels of key genes involved in DSBs repair and recombination were detected in HFD fetal oocytes (Figure 4).

Two waves of genome‐wide DNA methylation reprogramming are critical for germ cell development.^[^ ^38^, ^41^ ^]^ Methylome analysis revealed the significant hypermethylation across all genomic features in HFD fetal oocytes (Figure 5), indicating elevated DNA methylation during meiotic prophase I. Normally, E13.5 fetal oocytes undergo global demethylation, but maternal obesity clearly disrupts this process (Figure 5). GO analysis showed that DMRs in HFD fetal oocytes are involved in several biological processes including autophagy, metabolism, and embryonic development at E13.5 (Figure S7a,b, Supporting Information), and mitochondrial function at E18.5 (Figure S7c,d, Supporting Information). DNA cytosine methylation is a potential mediator of maternal effects on fetal growth and metabolic phenotypes. We confirmed the failure of demethylation in genes such as *Ddx4*, *Sohlh2*, *USP30*, *Kmt2d*, *Kmt2b*, and *Brat1* at E13.5 or E18.5 (Figure 6; Figure S11, Supporting Information). Notably, *Ddx4* and *Sohlh2* are genes related to meiosis and crucial for oocyte development; *Sohlh2* knockout mice were infertile.^[^ ^42^, ^43^, ^44^ ^]^ Such a comprehensive alteration in oocyte methylation patterns could potentially serve as the mechanistic foundation for maternal influences on offspring phenotypes. In addition, to explore the potential links between gene expression and DNA methylation status in PGCs, we performed an integrative analysis of RNA‐seq and BS‐seq data. The differentially expressed genes (DEGs) and the genes with DMRs in gene body and promoter regions between ND and HFD PGCs were compared. Nonetheless, there were very few genes exhibiting the differential transcription and altered methylation simultaneously (Figure S8, Supporting Information). Likewise, such an uncoupling of the DNA methylome and transcriptional control has also been reported in normal mouse PGCs^[^ ^45^ ^]^ and human PGCs.^[^ ^46^, ^47^ ^]^ For example, during the 7 weeks of embryo development (4‐ to 11‐week), the transcriptomes of human PGCs were stable in general, with several hundreds of genes changing their expression significantly. By contrast, the global DNA methylation was drastically decreased to 6.0% in the 10‐week female PGCs during this period. How the PGCs maintain RNA expression pattern when the DNA methylation is globally removed warrants further analysis.

Numerous interventions have been proposed to rejuvenate cells and organs, delay the onset of diseases associated with obesity, and extend both healthspan and lifespan. Among these interventions, TRF has garnered recognition as one of the effective strategies. For instance, circadian rhythm disruption perturbs glucose homeostasis through the loss of circadian transcriptional and epigenetic identity in type 2 diabetes mellitus mouse.^[^ ^48^ ^]^ Reinforcement of circadian fasting/feeding patterns through TRF augments the magnitude of circadian gene expression and improves metabolic function in animal models^[^ ^31^ ^]^ and human clinical trials.^[^ ^49^ ^]^ Similarly, we found that TRF intervention not only effectively improves the metabolic status of obese mother, but also significantly ameliorates the meiotic defects of fetal oocytes (Figure 6). Nonetheless, we noticed that TRF promotes the recovery in the proportion of oocytes at pachytene stage, while has little effects on the proportion of diplotene oocytes (Figure 6d). During oogenesis, the first diplotene oocytes can be detected in E17.5 embryos, and less than 10% of oocytes are at diplotene stage in E18.5 embryos.^[^ ^50^, ^51^ ^]^ Thus, the above observation may be explained by the following reason: i) the majority of oocytes are at pachytene stage in E18.5 embryos that we evaluated in the present study; ii) the effects of TRF on diplotene oocytes in HFD fetus may be delayed. In addition, TRF application partially prevented the epigenetic deficits in HFD fetal oocytes (Figure 6). Nevertheless, certain genomic loci we examined were not restored to normal level (Figure S11c–e, Supporting Information). In conclusion, our results provide an integrative picture of the development, transcription, and epigenetics in fetal oocytes exposed to maternal obesity (Figure 7). We provide molecular insights into the beneficial effects of TRF on fetal germ cell development. TRF may represents a non‐pharmacological intervention to prevent reproductive defects associated with obesity.

## 4 Experimental Section

### Animal Breeding and Treatment

All animal experiments were conducted in strict compliance with the regulations and guidelines established by the local animal ethics committee and approved by the Animal Care and Use Committee of Nanjing Medical University (IACVC: 1 703 017). Female ICR mice aged 3 weeks were purchased from Charles River Laboratories, China Inc., and were accommodated in ventilated cages maintained under a 12‐h light: 12‐h dark cycle, at a constant temperature (24 ± 2 °C), and with controlled humidity levels. These mice were randomly allocated to two dietary groups: one group received a High‐Fat Diet (HFD; D12492, 60% kcal from fat; 5.21 kcal g^−1^, Research Diets, Inc.), while the other group received a Normal Diet (ND; D1415, 7% kcal from fat; 3.5 kcal g^−1^, Beijing HFK Bioscience Co. Ltd) for a duration of 16 weeks.

### Time‐Restricted Feeding

According to previous reports,^[^ ^24^ ^]^ a cohort of 40 ICR mice commenced a HFD regimen at the age of 3 weeks. Subsequently, their body weights and food intake were meticulously recorded on a weekly basis. Upon reaching an average weight of 50 g, which typically occurred after ≈4 months on the HFD, the cohort was partitioned into two groups: the HFD group (20 mice) and the TRF group (20 mice). For the TRF intervention study, custom‐designed fully automated feeder cages were utilized. In this setup, the HFD group had unrestricted access to the diet at all times, while the TRF group had access to the HFD only during the hours between 10 pm and 6 am every day. A separate group of ICR mice was concurrently maintained on a normal diet (ND) and served as the control group for this study.

### Fetus Collection and Oocyte Isolation

*Fetus collection*: Gestational age was determined by the presence of a vaginal plug, designating midnight on the day of mating as day 0. Plugged female mice were isolated from males and housed individually. On embryonic days 13.5 and 18.5, mice were humanely euthanized, and embryos were collected and assessed according to the published protocols.^[^ ^52^ ^]^

*Oocyte isolation*: Embryonic gonads were dissected from at E13.5 or E18.5 after euthanizing pregnant female mice by cervical dislocation. Male and female gonads were distinguished by their distinct morphology. Female gonads were dissected in sterile cold PBS and immediately subjected to hypotonic or dissociated treatment for further analysis. Embryonic ovaries were enzymatically digested in 0.5% trypsin and 0.8 mg mL^−1^ DNase I (Worthington) at 37 °C for 3–5 min, followed by manual dissociation through pipetting. PGCs and matched somatic cells were isolated by FACS using an AriaII instrument (BD Biosciences) based on SSEA‐1 expression. PGCs and somatic cells were sorted directly into PBS for further methylome sequencing, and immediately frozen on dry ice or stored at −80 °C.

### Glucoregulatory Assessments

For the glucose tolerance test (GTT), 5–10 mice per group were fasted for 16 h prior to the test. Blood glucose levels were measured by tail bleeding at 0 min, and then 2 g kg^−1^ of glucose was injected intraperitoneally. Blood glucose was monitored at 30‐min intervals up to 120 min. The tip of the tail was severed, and a blood drop was used to measure the baseline glycemia with a glucose meter (Accu‐CHEK active; Roche Diagnostic). For the insulin tolerance test (ITT), mice were fasted for 6 h, and blood glucose levels were measured at 0 min before intraperitoneal injection of 0.75 IU kg^−1^ of insulin. Blood glucose was measured at 30‐min intervals up to 120 min. Terminal fasting glucose was also measured following 6 h of fasting. The area under the curve (AUC) for glucose was then calculated for both tests as follows:

(1) $$AUC=\left(\left(C1+C2\right)/2\right)\times \left(t2-t1\right)$$

Here, C1 and C2 represent the concentrations of glucose at time points t1 and t2, respectively.^[^ ^24^ ^]^ This calculation was performed for each time frame (0–30 min, 30–60 min, etc.), and the total AUC was calculated as the sum of all AUC calculations. The same mice were used to perform both GTT and ITT with a 3‐day interval.

### Fetal Oocytes Methylome Profiling

The SSEA‐1‐positive female germ cells obtained through FACS were washed with PBS, and DNA was isolated from the cell pellets using DNeasy Blood & Tissue Kits (QIAGEN).^[^ ^7^, ^10^, ^46^ ^]^ Genomic DNAs, along with 1% unmethylated lambda DNA (Thermo Scientific), were subjected to bisulfite conversion using a MethylCode Bisulfite Conversion Kit (Invitrogen) following the manufacturer's instructions. The DNA methylome of fetal oocytes was analyzed as previously described.^[^ ^8^, ^53^ ^]^ SSEA‐1‐positive PGCs obtained through FACS were washed with DPBS, and DNA was isolated from the cell pellets using lysis buffer (10 mm Tris‐Cl pH 7.4 and 2% SDS) with 0.5 mL protease K for 1 h at 37 °C. Libraries for sequencing were prepared using 5–100 ng of genomic DNA, as previously described with minor modifications.^[^ ^8^ ^]^ The quality and quantity of the purified library were evaluated using the Agilent Bioanalyzer and StepOnePlus Real‐Time PCR System (Applied Biosystems). Libraries were prepared for 125‐bp paired‐end sequencing on a HiSeq2500.

### Analysis of Methylome Data

All paired‐end BS‐seq reads were first adapter‐trimmed and quality‐filtered using Trim Galore (v0.6.4). Then Bismark (v0.22.3)^[^ ^54^ ^]^ was used to align the cleaned data to the reference genome (mm10) with parameters “–bowtie2 –unmapped –non_directional”. Subsequently, the commands deduplicate_bismark (with default parameters) and bismark_methylation_extractor (with parameters “–gzip –bedGraph”) from the Bismark software were used to remove duplicated reads due to PCR amplification and to extract cytonsine methylation information for every CpG site, respectively.

Averaged methylation ratios of CpG sites within every 20 kb window were taken to assess the overall CpG methylation status across the genome. Meanwhile, mean methylation ratios were calculated in different genomic regions of specific genomic features, such as CpG islands, exons, introns, and repeat elements. The annotation of CpG islands (CGIs) was extracted from the UCSC Genome Browser,^[^ ^55^ ^]^ and CGI shores were the regions 2 kb upstream and downstream of the CGIs. Exons, introns, UTRs were defined according to the RefSeq annotation obtained from the UCSC Genome Browser, and promoters were defined as the regions 2 kb upstream of the transcriptional start sites (TSSs) of the RefSeq genes. Repeat elements were extracted from the RepeatMastker track of the UCSC Genome Browser. DeepTools (v3.5.1)^[^ ^56^ ^]^ was used to visualize averaged methylation ratios along the gene bodies of all RefSeq genes, comparing between different samples.

Differentially methylated regions (DMRs) were identified using Metilene (v0.2‐8)^[^ ^57^ ^]^ with default parameters and filtered by mean difference > 10% and q value < 0.05. Genes overlapped with DMRs were defined as differentially methylated genes, and GO enrichment analysis of these genes was assisted by DAVID bioinformatics resources (v2022q1).^[^ ^58^ ^]^ DMRs were also intersected with different genomic features; when a DMR overlapped with multiple features, the feature with the largest intersection was chosen. Enrichment analysis of DMRs on each genomics feature was conducted by Binomial tests. The methylation levels of DMRs were represented by the mean methylation levels in their respective regions.

### Bisulfite Pyrosequencing

The methylation profile of 8 DMR regions were detected by bisulfite mutagenesis and sequencing as previously described.^[^ ^59^ ^]^ Bisulfite conversion was performed on ten pooled oocytes derived from ten mice for each group using the EZ DNA Methylation kit (ZYMO Research). The converted DNA was then amplified by nested PCR. The second‐round PCR products were subcloned, and approximately ten clones for each sample were sequenced. The methylation level of each sample was calculated by online tool QUMA web server (http://quma.cdb.riken.jp/) as described.^[^ ^60^ ^]^ Primer sequence information is provided in Table S5 (Supporting Information). To determine the methylation state of each sequence, the experiment was repeated three times starting from oocyte collection. The percentage of methylation was calculated as 100× (number of methylated CpG dinucleotides) / (total number of CpGs).

### RNA‐Sequencing and Data Analysis

RNA isolated from fetal oocytes (300 oocytes from 10 fetuses per sample, 2 samples for each group) was quantified using an Agilent Bioanalyzer RNA Pico Kit. Amplification was carried out using the SMART‐Seq2 method. Successful libraries were assessed for quality and size distribution and quantified using Qubit 3.0 Fluorometer and Agilent 2100 Bioanalyzer. For library preparation, the pooled and purified cDNA was fragmented by sonication and then converted into sequencing libraries according to the standard Illumina library preparation protocol. Library preparation integrity was verified with PerkinElmer LabChip GX Touch and StepOnePlus Real‐Time PCR System. After library qualification, sequencing libraries were generated on the Illumina Hiseq platform for PE150 sequencing.

Raw reads underwent initial quality control with FastQC (v0.11.9). Subsequently, Trim Galore (v0.6.4) was used to trim sequencing adapters and filter out low‐quality reads based on the evaluation of the raw data. The clean data were then aligned to the mus musculus reference genome (mm10) using HISAT2 (v2.2.1),^[^ ^61^ ^]^ followed by gene expression quantification with feature Counts (v2.0.3)^[^ ^62^ ^]^ for counts. Differential expression analysis was performed on count data using the DESeq2 (v1.30.1) R package, with genes having an absolute fold change >1.5 and adjusted P‐value <0.05 considered as differentially expressed genes (DEGs). Enrichment analysis of DEGs for GO terms was conducted using DAVID (v2022q1), with significantly enriched GO terms defined by a p‐value < 0.05. FPKM values were utilized to create heatmaps in R (v4.0.4).

### Immunofluorescence

Chromosome spreads were prepared as previously described.^[^ ^63^ ^]^ Ovaries were dissected from embryos at embryonic day 13.5 and 18.5 and treated with a hypotonic buffer [17 mm trisodium citrate dihydrate, 50 mm sucrose, 5 mm EDTA, 0.5 mm DTT, 30 mm Tris· HCl (pH 8.2)] for 20 min at 37 °C. Ovaries were dispersed in a 100 mm sucrose solution using tweezers under a stereomicroscope. The cell suspension was applied to adhesive microscope slides and fixed in a solution of 1% paraformaldehyde and 0.2% Triton X‐100 for 4 h in humidified chambers. After drying, the slides were rinsed by immersing them in 0.1% Triton X‐100 for 10 min, followed by three washes, and then soaked in distilled water for an additional 10 min. The washed slides were dried at room temperature. Slides were blocked in a solution of 5% (wt/vol) BSA in PBS for 2 h at room temperature, stored at −80 °C, or stained with primary antibodies (SYCP1: Abcam, ab15090; SYCP3: Abcam, ab97672; γ.H2AX: Abcam, ab22551; RAD51: Abcam, 133534; RPA2: Abcam, ab76420; MLH1: Proteintech, 11697‐1‐AP; CREST: Antibodies Incorporated, 15‐234) at 4 °C overnight (for CREST, at least 48 h). Slides were incubated with FITC (ZSGB‐BIO, ZF0312), TRITC (ZSGB‐BIO, ZF0316), or Cy5 (Jackson, 709605149) secondary antibodies. After washing three times for 10 min in PBS, which were diluted in blocking buffer, they were incubated for 2 h at room temperature in a wet chamber under dark conditions and washed three times again. Slides were incubated with Hoechst 33 342 for 15 min and washed with PBS two times. Slides were mounted with Vectashield (Vector, H‐1000, Shanghai, China). Finally, slides were analyzed under a fluorescence microscope (ZEISS 700 and ZEISS 800, Germany). Super‐resolution images were obtained by SIM (Nikon Eclipse Ti‐E) equipped with CFI SR Apo TIRF×100 oil objective (NA1.49). Image stacks were reconstructed using Nikon NIS software. For each observed antibody, immunofluorescence was conducted on oocytes from ND or HFD fetuses in parallel and under identical conditions. Images were always acquired using the same confocal microscope settings.

### Statistical Analysis

All experiments were repeated three times with similar results, and data from one representative experiment are shown unless otherwise stated. All analyses were performed using SPSS software version 16.0 (Chicago, IL, USA) and GraphPad Prism (Version 8.0). Statistical comparisons were made using two‐tailed Student's *t*‐tests, one‐way analysis of variance (ANOVA), Fisher's exact tests, or bootstrap tests, as appropriate. The data are presented as the mean ± standard deviation. Changes were considered statistically significant when P < 0.05.

### Data Availability

The BS‐seq data and RNA‐Seq data have been deposited in the Gene Expression Omnibus (GEO) and are accessible through accession number GSE263551 and GSE263548.

## Conflict of Interest

The authors declare no conflict of interest.

## Author Contributions

S.T., H.W., Q.C., and T.T. contributed equally to this work. S.T. and Q.W. conceived the projects. S.T., X.W., and Q.C. contributed to the DNA methylome analysis; S.T. X.Q., and J.L. contributed to the transcriptomics profiling. S.T., H.H., T.T., S.Z., L.H., H.S., and J.G. performed the embryo experiments. S.T. wrote, and Q.W. revised the manuscript. All authors reviewed and approved the manuscript for publication.

## Supporting information

Supporting Information

## Data Availability

The data that support the findings of this study are available from the corresponding author upon reasonable request.
