# Supplementary material for: Maternal Obesity Induces the Meiotic Defects and Epigenetic Alterations During Fetal Oocyte Development
Source: Adv Sci (Weinh). 2024 Jun 13;11(30):2309184. doi: 10.1002/advs.202309184 (PMC11321662; doi:10.1002/advs.202309184)
Supplement: Supplementary file 1 — Supporting Information [file ADVS-11-2309184-s001.pdf]

ADVANCED SCIENCE Open Access

Supporting Information

for *Adv. Sci.*, DOI 10.1002/advs.202309184

Maternal Obesity Induces the Meiotic Defects and Epigenetic Alterations During Fetal Oocyte Development

Shoubin Tang, Huihua Wu, Qiuzhen Chen, Tao Tang, Jiashuo Li, Huiqing An, Shuai Zhu, Longsen Han, Hongzheng Sun, Juan Ge, Xu Qian, Xi Wang and Qiang Wang**

Maternal Obesity Induces the Meiotic Defects and Epigenetic Alterations during Fetal Oocyte Development

Shoubin Tang[1, 3, 4], Huihua Wu[2, 4], Qiuzhen Chen[1, 4], Tao Tang[1, 4], Jiashuo Li[1], Huiqing An[1], Shuai Zhu[1], Longsen Han[1], Hongzheng Sun[1], Juan Ge[1], Xu Qian[3], Xi Wang[1*], and Qiang Wang[1,5*]

1 State Key Laboratory of Reproductive Medicine and Offspring Health, Changzhou Maternity and Child Health Care Hospital, Changzhou Medical Center, Nanjing Medical University, Nanjing, 211166, China

2 Suzhou Municipal Hospital, Nanjing Medical University, Nanjing 211166, China

3 Department of Nutrition and Food Hygiene, School of Public Health, Nanjing Medical University, Nanjing, 211166, China

4 These authors contributed equally

5 Lead contact

*Correspondence should be addressed to:

Qiang Wang, Ph.D., Professor, Nanjing Medical University, 101 Longmian Rd, Nanjing, Jiangsu, 211166 China

E-mail: qwang2012@njmu.edu.cn, Phone: +86-25-86869511; Fax: +86-25-86869511;

OR

Xi Wang, Ph.D., E-mail: xiwang@njmu.edu.cn.

Table of contents

Supporting figures ········· 3

Table S1 ········· 14

Table S2 ········· 46

Table S3 ········· 47

Table S4 ········· 48

Table S5 ········· 101

Supporting figures

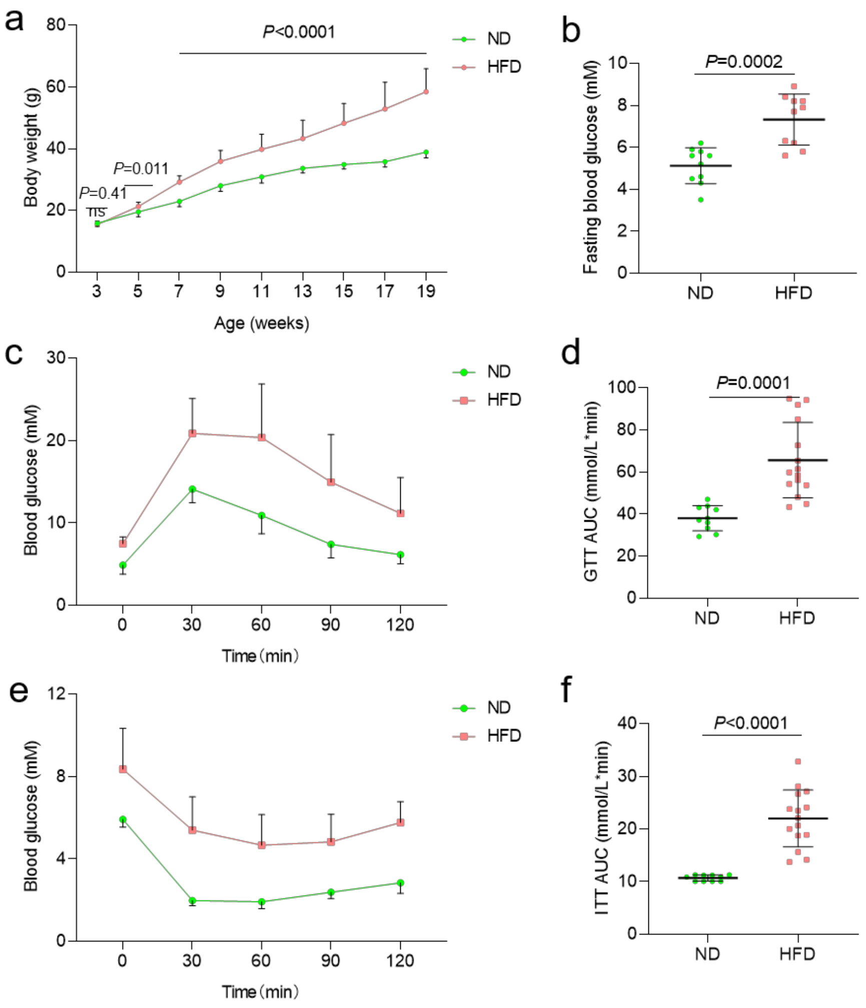

Figure S1. High-Fat diet induces obesity, glucose intolerance, and insulin resistance in female mice. Female mice were subjected to either a normal diet (ND) or a high-fat diet (HFD) for a duration of 16 weeks. The following parameters were assessed: **a,** Body weight of the mice (n = 10 for ND mice; n = 12 for HFD mice). **b,** Blood glucose levels after an 8-hour fasting period (n = 10 for ND mice; n = 10 for HFD mice). **c, d,** Blood glucose levels during the glucose tolerance test (GTT) and calculation of areas under the curve (AUC) (n = 10 for ND mice; n = 15 for HFD mice). **e, f,** Blood glucose levels during the insulin tolerance test (ITT) and calculation of areas under the curve (AUC) (n = 10 for ND mice; n = 15 for HFD mice). Data are presented as mean ± SD. Statistical analysis was performed using a two-tailed Student's *t*-test. Significance was set at P-value <0.05.

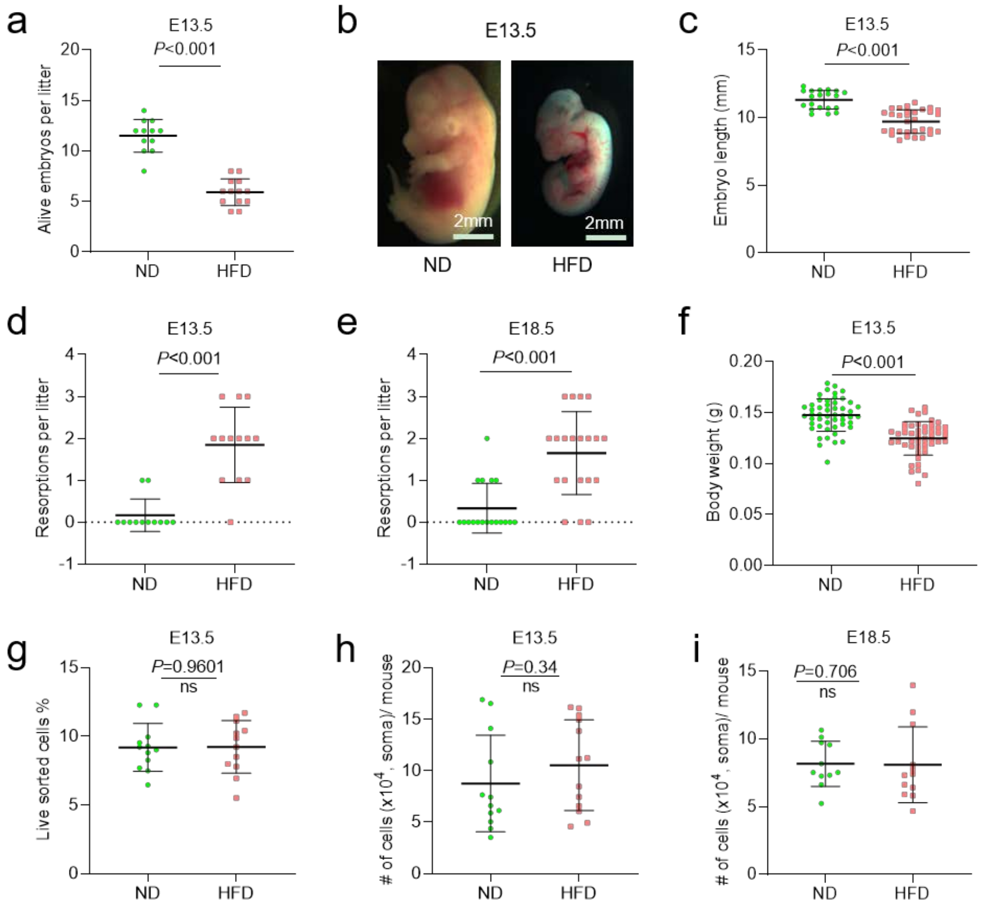

Figure S2. Maternal obesity impairs fetal development a, Fetal development was assessed by measuring the number of alive fetuses (**a,** ND = 12 and HFD = 13 litters at E13.5), crown–rump length (**b, c,** n = 20 for ND and n = 31 for HFD) in live embryos. **d, e,** Maternal obesity influences the resorption rate at E13.5 (n = 12 for ND and n = 13 for HFD) and at E18.5 (n = 18 for ND and n = 20 for HFD). **f,** Maternal obesity affects the body weight of fetuses at E13.5 (n = 50 for ND and n = 49 for HFD). **g,** Statistical analysis of the number of SSEA-1^{+} oocytes in each fetus at E13.5 (n = 12 for ND and n = 12 for HFD). **h, i,** Maternal obesity does not affect the average somatic cell number. At E13.5: n = 12 ND and n = 13 HFD biological replicates, at E18.5: n = 11 ND and n = 12 HFD biological replicates. Data are presented as mean ± SD. Statistical analysis was performed using a two-tailed Student's *t*-test. Significance was set at P-value <0.05.

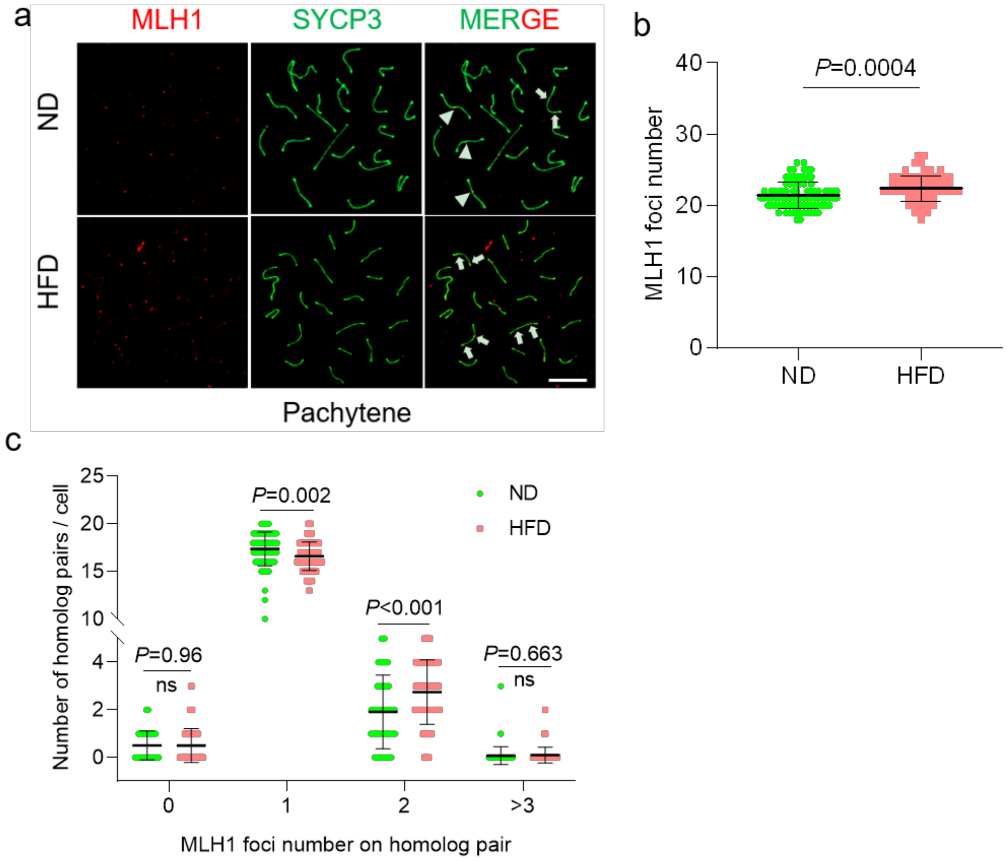

Figure S3. Obesity induces aberrant meiotic crossover formation in fetal oocytes. a, Chromosome spreads of fetal oocytes from E18.5 ND and HFD mice were immunostained for SYCP3 (green) and MLH1 (red) at the pachytene stage. While arrowheads indicate a single MLH1 signal on a homologous pair, arrows indicate multiple MLH1 signals on a homologous pair. **b,** Quantification of MLH1 foci number per cell at the pachytene stages. **c,** Distribution of MLH1 foci at different homologous pairs in ND and HFD fetal oocytes (n = 91 oocytes for ND and n = 90 oocytes for HFD, for each group, three mice were analyzed). Data are presented as means ± SD. Statistical analysis was performed using a two-tailed Student's *t*-test. Significance was set at P-value <0.05. "n.s." indicating no statistical significance.

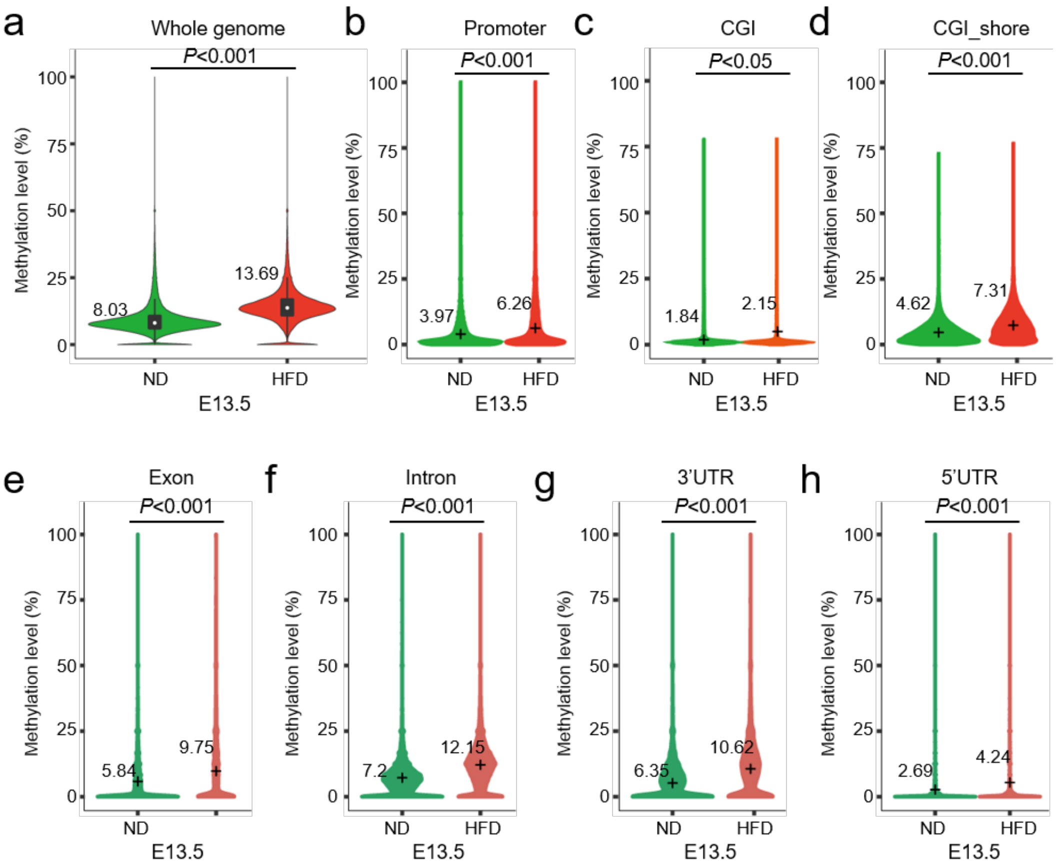

Figure S4. Global DNA hypermethylation across different genomic features in HFD fetal oocytes. a, Average methylation levels in the genome of fetal oocytes at E13.5. **b-h,** Violin plots displaying methylation levels for different genomic features, including promoters (**b**), CGI (**c**), CGI shore (**d**), exon (**e**), intron (**f**), 3'UTR (**g**) and 5'UTR (**h**) in E13.5 fetal oocytes. The mean methylation levels are denoted by numerical values and black crosses. Statistical analyses were conducted using a bootstrap test.

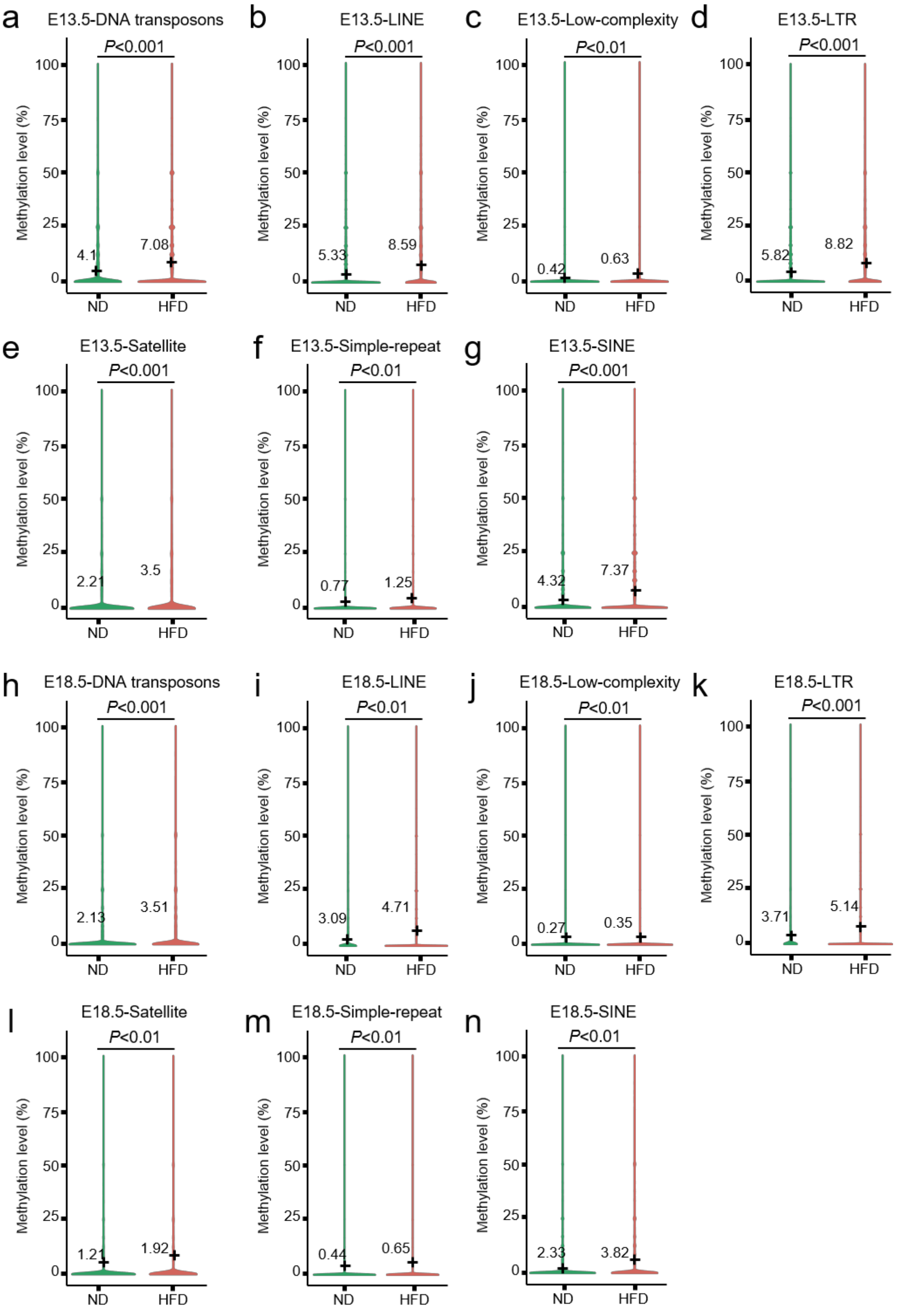

Figure S5. Global DNA hypermethylation across different repetitive elements in oocytes from HFD fetuses. Violin plots illustrating the methylation levels of major repetitive elements in fetal oocytes from ND and HFD mice at E13.5 (**a-g**) and E18.5 (**h-n**), respectively. Mean methylation levels are indicated by the numerical value and a black cross. Statistical analyses were performed using a bootstrap test.

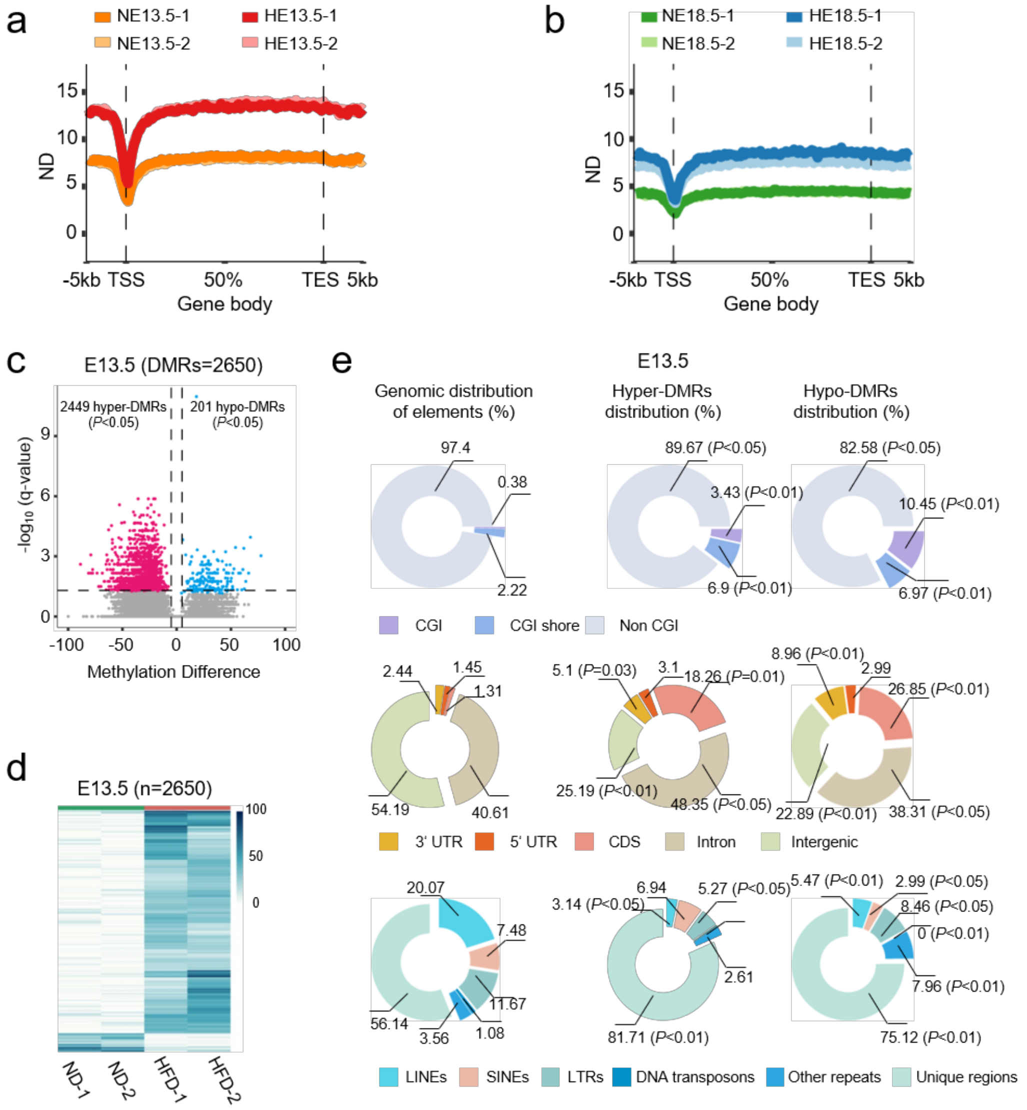

Figure S6. DNA methylation aberrations in fetal oocytes. a, b, Average DNA methylation levels (CpG sites) along gene bodies, 5 kb upstream of transcription start sites (TSSs), and 5 kb downstream of transcription end sites (TES) of all RefSeq genes across different samples. **c,** Volcano plot depicting the results of DNA methylation microarray analysis in fetal oocytes from ND and HFD backgrounds at E13.5. Dots positioned in the upper left corner represent significantly hypermethylated probes, while those in the upper right corner represent significantly hypomethylated probes with a statistical significance of P < 0.05 and a fold change >1.5. **d,** Heatmap visualization illustrating DMRs in ND and HFD fetal oocytes at E13.5, where each line represents a distinct DMR. High methylation levels are denoted in blue, while low methylation levels are depicted in white. **e,** The relative proportion of DMRs among

CpG Islands (CGIs) and CGI shores (top), the relative proportion of DMRs among coding and noncoding regions (middle), and the relative proportion of DMRs among distinct sequence and repetitive elements throughout the genome (bottom). Fisher's exact test was employed to assess potential enrichment or depletion of DMRs relative to the genomic distribution of these elements: significance was set at P-value <0.05.

a

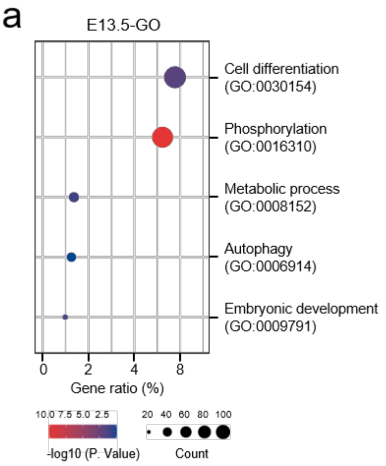

b

	Gene	Function involvement	Reference (PMID)
Cell differentiation	*Itsn2*	Embryonic proliferation and differentiation	34692701
	Mef2b	Cell differentiation, proliferation	30205047
Oocyte and embryonic development	*Ddx4*	Embryogenesis, spermatogenesis, and cellular growth and division	26444631
	Sohlh2	Oocyte development, early mouse folliculogenesis and oocyte-specific gene	28504655
Phosphorylation	*Dyrk3*	Catalyze autophosphorylation on serine/threonine and tyrosine residues	12356771
	Alk	Phosphorylation	36253486
Autophagy and apoptosis	*Uvrag*	Autophagy, apoptotic and inflammation	31905312
	Phf23		32380078
Metabolic process	*Pygb*	Glucose metabolism	31905009
	Camk1d	Type 2 diabetes	30054458

c

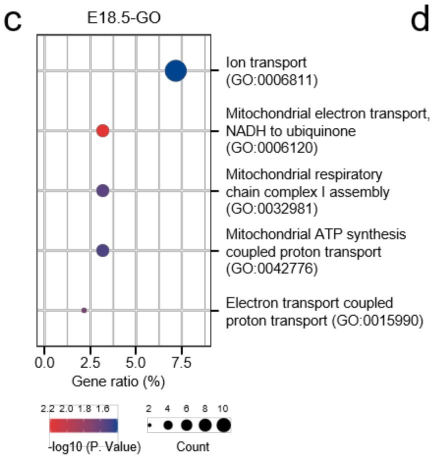

d

	Gene	Function involvement	Reference (PMID)
Ion transport	*Kcnc2*	Enables transmembrane transporter binding activity	31972370
	Slc12a3	Sodium ion transmembrane transporter	34607911
Mitochondrial function	*MT-ND5*	Mitochondrial function and endoplasmic reticulum stress	31176373
	MT-ND2	NADH dehydrogenase activity and protein kinase binding activity	22414913
	MT-ND2	Mitochondrial electron transport and mitochondrial respiratory chain complex I assembly	34382907
	MT-CO1	Mitochondrial electron transport and response to oxidative stress	35220898

Figure S7. GO enrichment analysis of DMRs. a, c, GO enrichment analysis results for DMRs in E13.5 and E18.5 fetal oocytes. **b, d,** Function and reference display of selected genes associated DMRs.

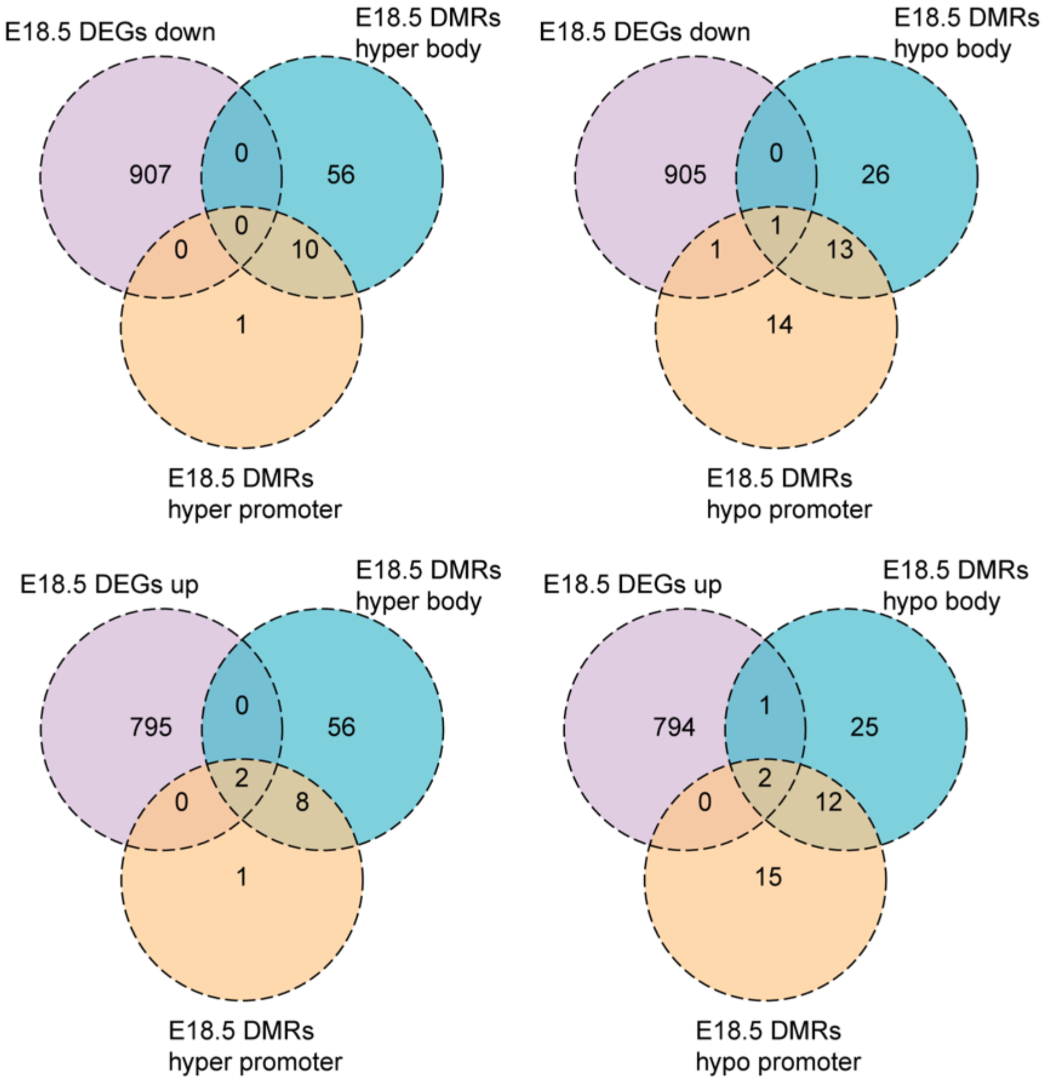

Figure S8. Venn diagrams showing the overlap between differentially expressed genes and the genes with hypo-/hyper-methylated regions in E18.5 PGCs.

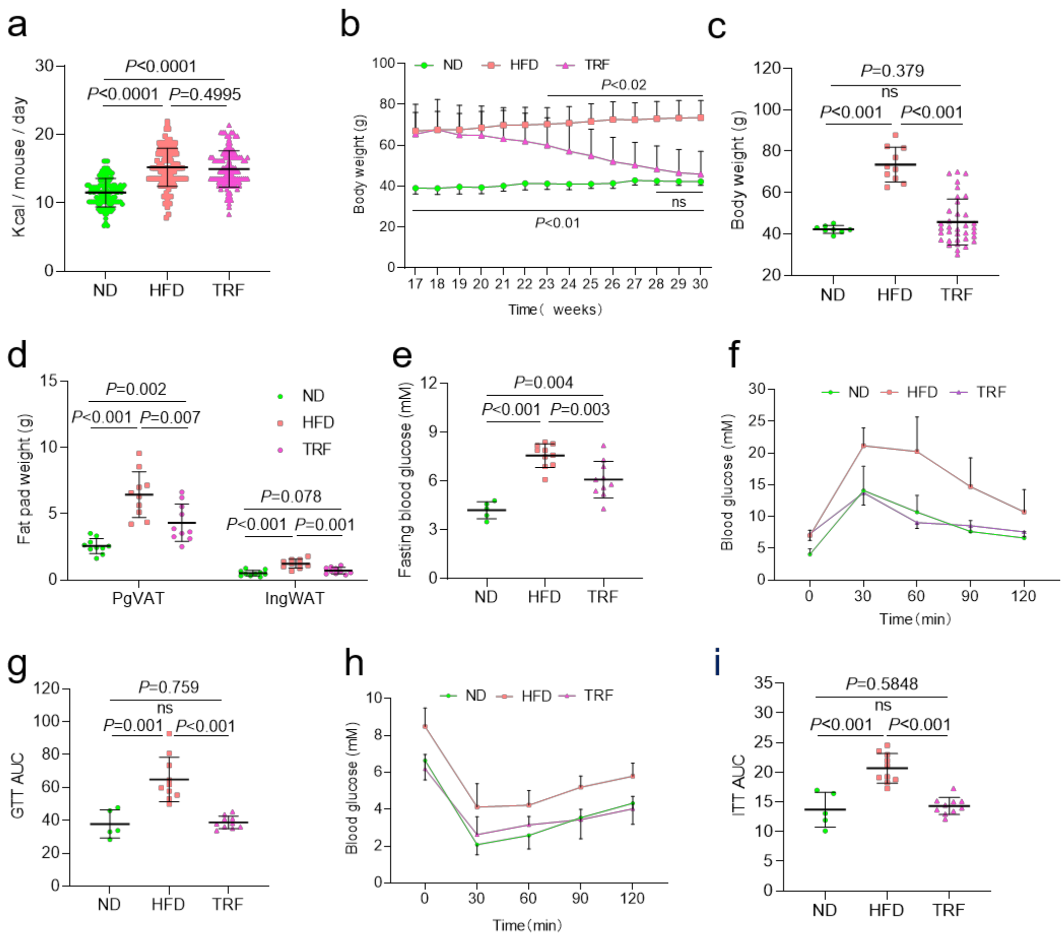

Figure S9. TRF enhances glucose metabolism in HFD-induced obese mice. a, Daily average food intake in mice under ND, HFD, and TRF conditions (n = 12 for each group). **b,** Weekly body weights of mice in ND, HFD, and TRF groups after initiating the TRF protocol (n = 8 for ND, n = 10 for HFD, n = 35 for TRF). **c,** The final weight of each group of mice. **d,** After TRF, measurements of pgVAT and ingWAT fat mass in each group (n = 10 in each group). **e-i**, Assessment of blood glucose levels in mice after an 8-hour fast (**e**) fasting blood glucose (n = 5 for ND mice, n = 10 for HFD mice, n = 10 for TRF), blood glucose levels during GTT and calculation of AUC (**f, g**), blood glucose levels during ITT and calculation of AUC (**h, i**) (n = 5 for ND, n = 10 for HFD, n = 10 for TRF). Data presented as mean ± SD. Student's *t*-test (two-tailed) was employed for statistical analysis. Significance was set at P-value <0.05. "n.s." indicating no statistical significance.

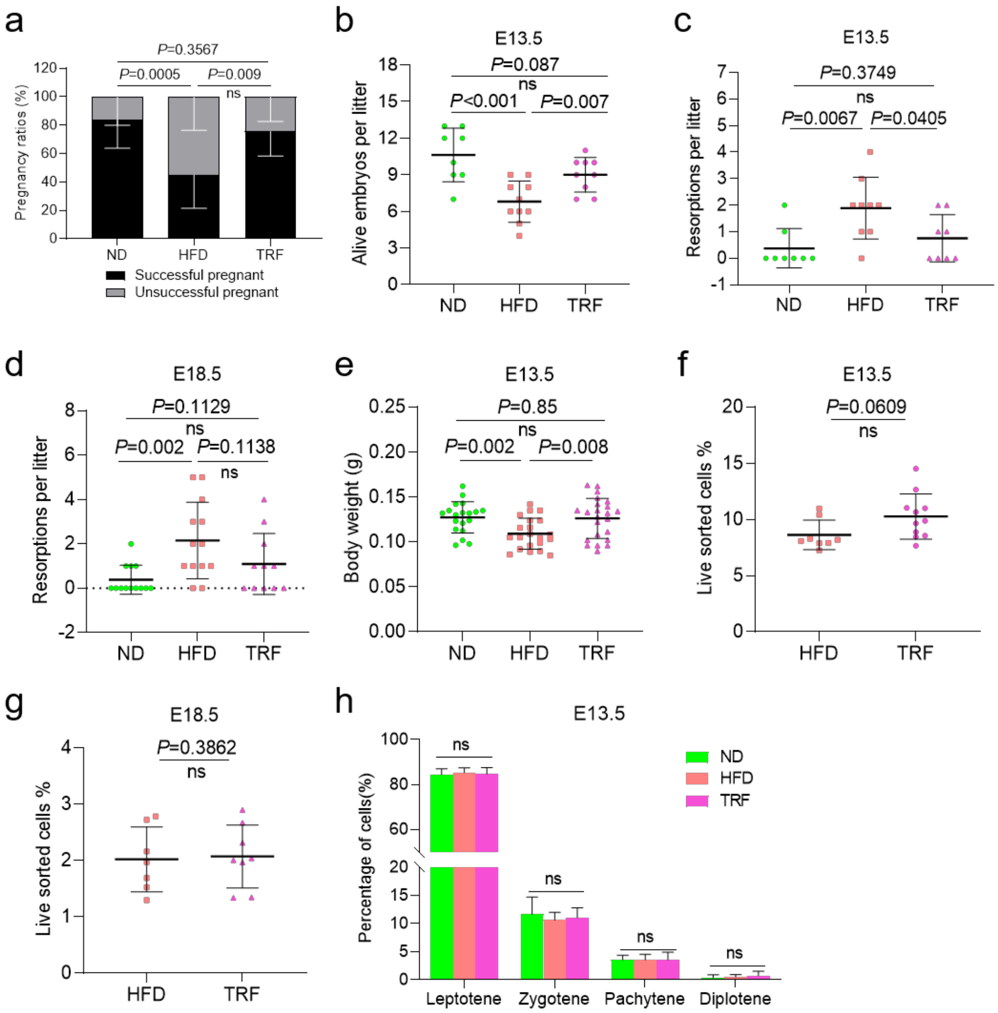

Figure S10. TRF enhances fetal development in HFD-Induced obese mice. a, Assessment of pregnancy rates in each group of mice (n = 13 for ND, n = 9 for HFD, n = 8 for TRF). **b,** Evaluation of fetal development in surviving embryos at E18.5. TRF improved the rate of viable fetuses in HFD mice (n = 8 for ND, n = 10 for HFD, n = 9 for TRF). **c, d,** Reduction in fetal absorption rate in HFD mice at E13.5 and E18.5 with TRF. **e,** Assessment of fetal body weight at E13.5. **f, g,** Quantification of the number of SSEA-1^+ oocytes in each fetus at E13.5 and E18.5 (n = 8 for HFD and n = 11 for TRF at E13.5, n = 7 for HFD and n = 8 for TRF at E18.5). **h,** Analysis of meiotic stages frequencies in ND (n=535), HFD (n=592) and TRF (n=534) fetus oocytes at E13.5. A minimum of three mice were analyzed for each group. Data represented as mean ± SD. Student's *t*-test (two-tailed) was applied for statistical analysis. Significance was set at P-value <0.05. "n.s." indicating no statistical significance.

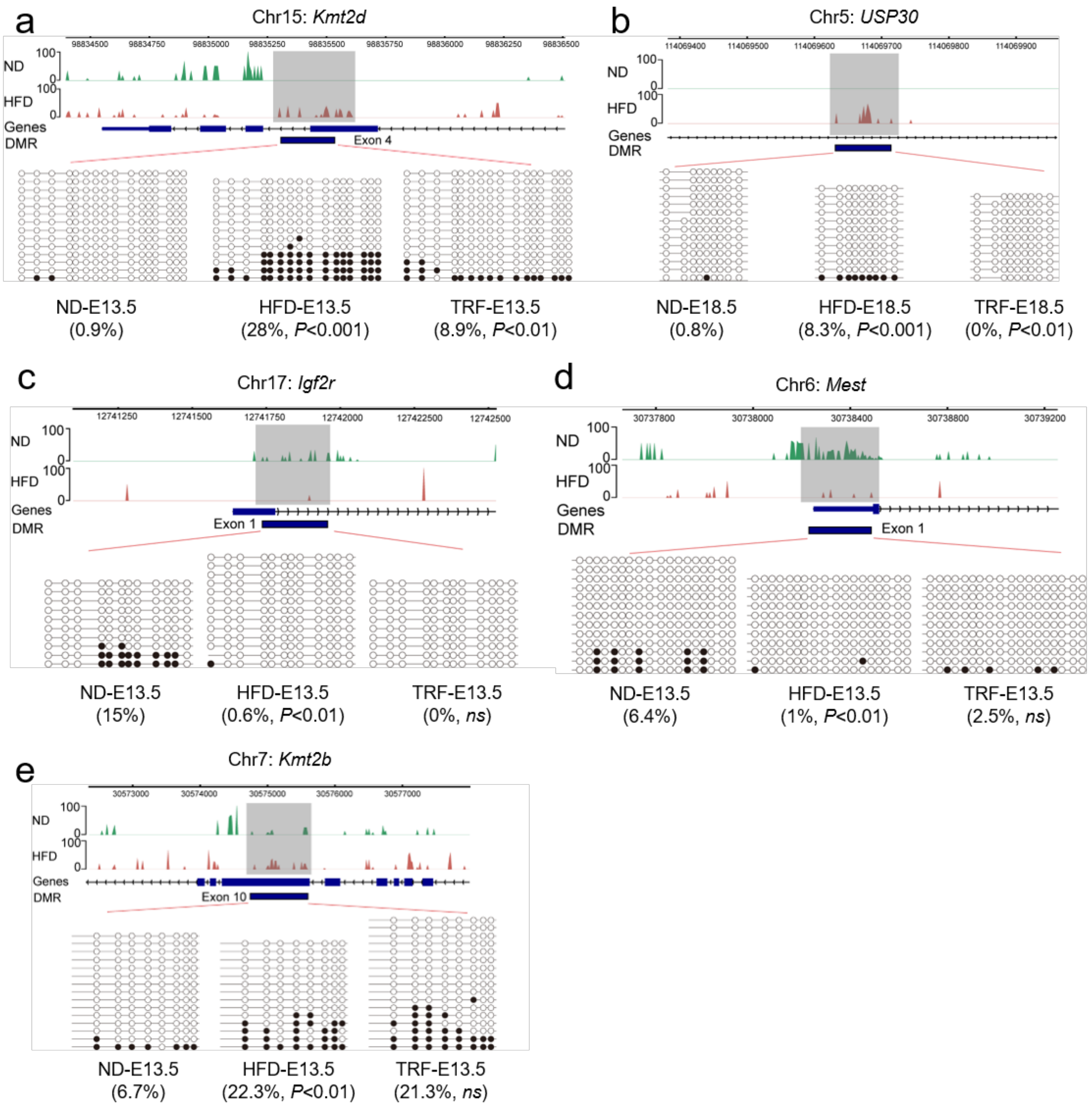

Figure S11. Impact of HFD and TRF on methylation patterns of fetal oocytes in HFD mice.

Graphical depiction of methylation patterns at different genomic loci in ND, HFD, and TRF fetal oocytes. The region enclosed by the gray box was selected for further validation. BS-seq analysis confirming the methylation status of *kmt2d*, *USP30*, *Mest*, *Igf2r*, and *Kmt2b* in ND, HFD, and TRF fetal oocytes. Open and filled circles represent unmethylated and methylated CpGs, respectively. Percentages of methylated CpGs are displayed beneath each panel. Significance was set at P-value <0.05. "n.s." indicating no statistical significance.

Table S1

Identified 1704 DEGs between ND and HFD fetal oocytes at E18.5.

baseMean	log2FoldC	lfcSE	stat	p.value	p.adj	gene name	significance
11687.34	-10.6939	1.376628	-7.76819	7.96E-15	1.25E-10	Mrpl42	DOWN
10185.49	-10.4221	1.429441	-7.29106	3.08E-13	2.42E-09	Nhp2	DOWN
4993.883	-12.6429	1.840483	-6.86934	6.45E-12	3.38E-08	Sar1b	DOWN
8886.196	-9.56562	1.451457	-6.59036	4.39E-11	1.67E-07	Fth1	DOWN
9697.495	-10.828	1.658239	-6.52984	6.58E-11	1.67E-07	Rnf167	DOWN
5627.827	-10.7028	1.643661	-6.51155	7.44E-11	1.67E-07	Selenoh	DOWN
6206.884	-13.4463	2.052205	-6.5521	5.67E-11	1.67E-07	Utf1	DOWN
9170.924	-10.7258	1.670041	-6.4225	1.34E-10	2.34E-07	Snx12	DOWN
7625.12	-9.71834	1.51136	-6.4302	1.27E-10	2.34E-07	Supt4a	DOWN
8461.513	-9.93515	1.550895	-6.40608	1.49E-10	2.35E-07	Cdkn2aipnl	DOWN
8551.411	-9.48743	1.528715	-6.20615	5.43E-10	7.37E-07	Lamtor5	DOWN
7858.909	-11.2074	1.807492	-6.2005	5.63E-10	7.37E-07	Ssna1	DOWN
6021.936	-9.76456	1.594515	-6.12384	9.13E-10	1.10E-06	Atp5md	DOWN
5318	-9.55343	1.632065	-5.85359	4.81E-09	5.40E-06	Ube2b	DOWN
3094.397	-10.133	1.766619	-5.73584	9.70E-09	1.02E-05	Polr2i	DOWN
44.24476	4.97079	0.871887	5.701183	1.19E-08	1.17E-05	Gm42900	UP
7018.786	-9.50866	1.685432	-5.64168	1.68E-08	1.56E-05	Rars2	DOWN
57.39002	6.028621	1.076203	5.601753	2.12E-08	1.85E-05	Gm43622	UP
5439.153	-12.1425	2.179416	-5.57147	2.53E-08	2.09E-05	Tmed3	DOWN
4065.641	-9.71523	1.762302	-5.51281	3.53E-08	2.78E-05	Scx	DOWN
5914.966	-8.44737	1.557241	-5.42457	5.81E-08	4.15E-05	Laptm4b	DOWN
7710.716	-8.68648	1.600942	-5.42586	5.77E-08	4.15E-05	Pak1ip1	DOWN
3272.963	-12.5121	2.323565	-5.38486	7.25E-08	4.96E-05	Rps41	DOWN
10272.4	-11.1126	2.082413	-5.33642	9.48E-08	6.21E-05	Smim12	DOWN
4589.221	-8.85991	1.671747	-5.29979	1.16E-07	7.01E-05	Hat1	DOWN
4867.687	-11.5574	2.178059	-5.30629	1.12E-07	7.01E-05	Lgals1	DOWN
104.382	-7.04884	1.337011	-5.27209	1.35E-07	7.57E-05	Gm7125	DOWN
5271.016	-9.74645	1.848302	-5.27319	1.34E-07	7.57E-05	Mrpl40	DOWN
4761.435	-8.56835	1.628068	-5.2629	1.42E-07	7.69E-05	Snrpd2	DOWN
42.36453	6.524284	1.261973	5.169908	2.34E-07	1.23E-04	Gm15758	UP
5586.672	-8.42635	1.64669	-5.11714	3.10E-07	1.52E-04	Asf1b	DOWN
5744.948	-8.42334	1.644784	-5.12125	3.04E-07	1.52E-04	Wdr75	DOWN
1468.898	-9.62559	1.893636	-5.08313	3.71E-07	1.72E-04	Pdss1	DOWN
4631.352	-8.50815	1.674095	-5.08224	3.73E-07	1.72E-04	Tecr	DOWN
90.93844	-6.8481	1.354122	-5.05723	4.25E-07	1.91E-04	Gm21399	DOWN
6413.628	-9.32182	1.861302	-5.00822	5.49E-07	2.37E-04	Golt1b	DOWN
4337.335	-13.2676	2.650742	-5.00525	5.58E-07	2.37E-04	Mettl18	DOWN
36.56927	-5.12084	1.034566	-4.94975	7.43E-07	3.07E-04	Rbx1-ps	DOWN
5035.418	8.017527	1.622776	4.940625	7.79E-07	3.14E-04	Sycp1	UP
2793.405	-8.5805	1.748935	-4.90613	9.29E-07	3.65E-04	Gstp1	DOWN
7533.926	8.904121	1.827079	4.873419	1.10E-06	4.21E-04	Uchl1	UP
54.34481	-5.60232	1.153352	-4.85743	1.19E-06	4.45E-04	Gm13604	DOWN
3088.074	8.505659	1.75359	4.850426	1.23E-06	4.50E-04	Zfp160	UP
66.64935	-5.4167	1.124668	-4.81627	1.46E-06	5.13E-04	Gm12669	DOWN
3523.688	-10.3698	2.15336	-4.81563	1.47E-06	5.13E-04	Pigp	DOWN
3350.107	-8.69719	1.80865	-4.80866	1.52E-06	5.19E-04	Mapk1	DOWN
3169.194	-8.59006	1.789491	-4.80028	1.58E-06	5.30E-04	Ubl4a	DOWN
3451.34	8.106463	1.697446	4.775683	1.79E-06	5.87E-04	AU015836	UP
31.19469	-4.41788	0.927917	-4.76107	1.93E-06	6.11E-04	Gm10095	DOWN

56.34131	-4.85255	1.019594	-4.7593	1.94E-06	6.11E-04	Ppp1r15a	DOWN
6465.006	-9.85437	2.079585	-4.73862	2.15E-06	6.63E-04	Mrpl47	DOWN
221.7098	-6.50462	1.384084	-4.69958	2.61E-06	7.88E-04	Gstp2	DOWN
6790.995	-9.44264	2.015091	-4.68596	2.79E-06	8.26E-04	2210016F16Rik	DOWN
2655.915	-9.33303	1.993453	-4.68184	2.84E-06	8.28E-04	Rps2-ps13	DOWN
5379.937	-9.75598	2.089575	-4.66888	3.03E-06	8.66E-04	Psma1	DOWN
2698.894	-11.1358	2.391975	-4.65547	3.23E-06	9.07E-04	Manba1	DOWN
5982.698	-10.0792	2.169232	-4.64646	3.38E-06	9.31E-04	Prpsap2	DOWN
2115.299	-8.43617	1.818981	-4.63786	3.52E-06	9.54E-04	Siah1b	DOWN
520.8539	-12.3296	2.662028	-4.63165	3.63E-06	9.62E-04	Gm44	DOWN
46.92309	4.673643	1.010344	4.625796	3.73E-06	9.62E-04	Gpr34	UP
657.7803	-8.53384	1.844591	-4.62641	3.72E-06	9.62E-04	Rtraf-ps	DOWN
5594.677	-10.152	2.197351	-4.62012	3.84E-06	9.72E-04	Cript	DOWN
5061.347	9.02794	1.9594	4.607502	4.08E-06	0.001017	Pfdn5	UP
996.3477	-9.96818	2.166607	-4.60082	4.21E-06	0.001034	Psenen	DOWN
8983.48	-9.30029	2.032293	-4.57625	4.73E-06	0.001145	Imp4	DOWN
3543.746	-9.86344	2.159214	-4.56807	4.92E-06	0.001172	Cox7b	DOWN
4883.249	7.441855	1.630705	4.563581	5.03E-06	0.00118	Elavl2	UP
2894.858	-10.3013	2.261485	-4.55512	5.24E-06	0.00121	Pdcd10	DOWN
6558.367	-9.69534	2.130496	-4.55074	5.35E-06	0.001218	Supt3	DOWN
6649.924	-9.36677	2.05987	-4.54726	5.43E-06	0.00122	Nol7	DOWN
4391.678	-8.68604	1.914024	-4.5381	5.68E-06	0.001239	Hint1	DOWN
7944.669	-9.56965	2.108223	-4.5392	5.65E-06	0.001239	Snap47	DOWN
36.51697	-3.46903	0.767352	-4.52078	6.16E-06	0.001313	Aurka	DOWN
4573.517	-7.31856	1.619114	-4.5201	6.18E-06	0.001313	Dock7	DOWN
3141.338	7.733295	1.714148	4.51145	6.44E-06	0.00135	Myl6	UP
8121.254	-9.26853	2.059529	-4.50032	6.79E-06	0.001403	Nfu1	DOWN
3752.636	-8.44301	1.880673	-4.48936	7.14E-06	0.001422	Atpif1	DOWN
5679.129	-8.93571	1.990225	-4.4898	7.13E-06	0.001422	Coprs	DOWN
5402.912	7.086092	1.578019	4.490499	7.11E-06	0.001422	Srp9	UP
2939.011	-11.0318	2.462594	-4.47973	7.47E-06	0.00145	Cox7a1	DOWN
197.4283	-7.61612	1.699182	-4.48223	7.39E-06	0.00145	Tagap	DOWN
2783.142	-7.8245	1.748725	-4.4744	7.66E-06	0.00146	Gpx4	DOWN
111.3295	-6.96803	1.557772	-4.47308	7.71E-06	0.00146	Mageb16	DOWN
58.72587	3.957734	0.888748	4.453154	8.46E-06	0.00152	Cox17	UP
2739.679	7.67165	1.720733	4.458361	8.26E-06	0.00152	Hspa4l	UP
3169.608	-7.60856	1.709027	-4.45198	8.51E-06	0.00152	Ipo9	DOWN
3417.75	-9.7408	2.187665	-4.4526	8.48E-06	0.00152	Rhox6	DOWN
6031.941	-8.16511	1.832113	-4.45667	8.32E-06	0.00152	Uqcrb	DOWN
121.5146	-5.59029	1.257954	-4.44395	8.83E-06	0.001543	Rhox9	DOWN
2942.467	7.614361	1.712717	4.445778	8.76E-06	0.001543	Trim33	UP
4174.907	-9.7603	2.201085	-4.43431	9.24E-06	0.001596	Rps19bp1	DOWN
145.4179	6.797471	1.53402	4.43115	9.37E-06	0.001602	Scml1	UP
3788.259	7.94192	1.795537	4.423144	9.73E-06	0.00161	Nobox	UP
978.0273	9.874734	2.232351	4.423468	9.71E-06	0.00161	Pet100	UP
6889.443	-9.05999	2.04809	-4.42363	9.71E-06	0.00161	Tiparp	DOWN
2461.478	7.667032	1.737903	4.411656	1.03E-05	0.001677	Ola1	UP
1145.487	-10.172	2.306652	-4.40983	1.03E-05	0.001677	Rgcc	DOWN
58.63184	3.346276	0.759234	4.407438	1.05E-05	0.001678	Coq8a	UP
5014.468	7.06995	1.606827	4.399944	1.08E-05	0.001719	Col4a1	UP
111.394	6.622138	1.510956	4.382748	1.17E-05	0.001789	Gm11739	UP
1511.181	11.23487	2.566019	4.378325	1.20E-05	0.001789	Gm43071	UP
2464.772	-7.82604	1.785039	-4.38424	1.16E-05	0.001789	Hmgn2	DOWN
2388.812	-7.68006	1.753313	-4.38031	1.19E-05	0.001789	Lgr4	DOWN

2307.314	-9.80058	2.238326	-4.37853	1.19E-05	0.001789	Ptrhd1	DOWN
3641.043	-7.95737	1.818235	-4.37643	1.21E-05	0.001789	Sf3a3	DOWN
76.40898	-5.29278	1.206572	-4.38663	1.15E-05	0.001789	Tuba3a	DOWN
3388.529	7.656881	1.752728	4.36855	1.25E-05	0.001838	Taf7l	UP
965.4805	-9.21982	2.113711	-4.36191	1.29E-05	0.00184	Acot9	DOWN
117.473	-6.10321	1.399754	-4.3602	1.30E-05	0.00184	Eif3s6-ps2	DOWN
6071.86	-9.09635	2.084406	-4.364	1.28E-05	0.00184	Mrpl52	DOWN
1454.336	-9.13163	2.091897	-4.36524	1.27E-05	0.00184	Tmx1	DOWN
138345.3	2.621308	0.601728	4.356304	1.32E-05	0.001857	mt-Rnr2	UP
2105.191	-9.94693	2.288064	-4.34731	1.38E-05	0.001904	Alg14	DOWN
43.08915	3.745788	0.861714	4.346907	1.38E-05	0.001904	Nudt16l1	UP
3065.207	-7.85149	1.81188	-4.33334	1.47E-05	0.002008	Eif4a1	DOWN
128.4436	-5.83867	1.349153	-4.32765	1.51E-05	0.002025	Tmed2	DOWN
7832.211	-8.52916	1.970166	-4.32916	1.50E-05	0.002025	Tpgs2	DOWN
1783.419	-10.0207	2.324588	-4.31073	1.63E-05	0.002168	Car13	DOWN
1160.182	9.971443	2.319701	4.29859	1.72E-05	0.002252	A230005M16Rik	UP
231.3569	-7.33336	1.705604	-4.29957	1.71E-05	0.002252	Fam216a	DOWN
998.0984	-8.10825	1.888482	-4.29353	1.76E-05	0.002282	Rab9	DOWN
84.41751	-4.09199	0.953528	-4.29143	1.78E-05	0.002282	Stk38l	DOWN
5433.329	-9.41349	2.194209	-4.29015	1.79E-05	0.002282	Surf1	DOWN
1383.959	-10.0009	2.333177	-4.28638	1.82E-05	0.002302	1190007I07Rik	DOWN
457.5148	10.07689	2.353967	4.280811	1.86E-05	0.002342	C230086J09Rik	UP
2027.019	-8.50822	1.990031	-4.27542	1.91E-05	0.00238	Plp2	DOWN
95.90659	-4.69376	1.098347	-4.27348	1.92E-05	0.002382	Gm9333	DOWN
154.7566	-6.42935	1.505156	-4.27155	1.94E-05	0.002384	4930549G23Rik	DOWN
43.3915	-3.38163	0.792567	-4.26668	1.98E-05	0.002418	Ube2d1	DOWN
1623.771	-9.46681	2.220087	-4.26416	2.01E-05	0.002426	1110038F14Rik	DOWN
5465.245	8.92727	2.096667	4.257839	2.06E-05	0.00244	Dmap1	UP
246.6807	-7.5679	1.77679	-4.25931	2.05E-05	0.00244	Phlda2	DOWN
1467.161	-7.55027	1.772183	-4.26044	2.04E-05	0.00244	Ss18l2	DOWN
7114.013	-8.75562	2.058967	-4.25243	2.11E-05	0.002481	Coq7	DOWN
299.7176	-6.99093	1.646223	-4.24665	2.17E-05	0.00252	Cox11	DOWN
3628	-7.19564	1.694862	-4.24556	2.18E-05	0.00252	Metap2	DOWN
123.57	-6.0994	1.438904	-4.23892	2.25E-05	0.002577	Nipsnap3b	DOWN
983.7321	9.494298	2.245141	4.228821	2.35E-05	0.002656	0610040J01Rik	UP
1215.845	-8.77052	2.074728	-4.22731	2.37E-05	0.002656	Gm37219	DOWN
2463.101	7.423187	1.755929	4.227499	2.36E-05	0.002656	Znrf1	UP
2185.556	-7.61762	1.805806	-4.2184	2.46E-05	0.002743	Vamp4	DOWN
5494.853	-7.47558	1.774159	-4.21359	2.51E-05	0.002782	Pole	DOWN
2479.558	-8.32192	1.977863	-4.20753	2.58E-05	0.002796	2310061I04Rik	DOWN
3046.423	-9.35705	2.223679	-4.20791	2.58E-05	0.002796	B230118H07Rik	DOWN
4899.953	-9.08689	2.160358	-4.2062	2.60E-05	0.002796	Dnd1	DOWN
2172.117	9.186711	2.182403	4.209447	2.56E-05	0.002796	Gtsf1	UP
2102.228	9.776662	2.326313	4.202644	2.64E-05	0.002821	Gm47918	UP
2776.582	9.01221	2.146324	4.198905	2.68E-05	0.002849	Thy1	UP
4268.307	-9.13959	2.179692	-4.19307	2.75E-05	0.002884	Hikeshi	DOWN
1860.197	8.726494	2.080607	4.194205	2.74E-05	0.002884	Sdf2	UP
3732.15	9.382063	2.245455	4.178245	2.94E-05	0.003058	Znrd1	UP
199.6428	-6.86599	1.645094	-4.17362	3.00E-05	0.00308	Ak6	DOWN
2216.998	7.313108	1.751792	4.174644	2.98E-05	0.00308	Dctn2	UP
165.4421	-6.03901	1.44897	-4.1678	3.08E-05	0.003139	Timm8b	DOWN
4694.542	9.263701	2.225108	4.163259	3.14E-05	0.003182	Pcbd2	UP
2429.732	-9.34685	2.249692	-4.15472	3.26E-05	0.003225	Arl6ip4	DOWN
2662.028	7.552257	1.817895	4.154396	3.26E-05	0.003225	Atg14	UP

3484.236	-9.35552	2.249719	-4.15853	3.20E-05	0.003225	Ndufa8	DOWN
938.4684	7.323846	1.762768	4.154743	3.26E-05	0.003225	Prps1	UP
4051.087	-8.88822	2.140288	-4.15282	3.28E-05	0.003226	Clip3	DOWN
1695.589	9.59603	2.311514	4.151405	3.30E-05	0.003226	Mrps18b	UP
3230.959	-9.12877	2.200124	-4.14921	3.34E-05	0.003237	Ensa	DOWN
1556.883	8.313227	2.004409	4.147471	3.36E-05	0.003239	Figla	UP
4521.843	-7.81529	1.885516	-4.14491	3.40E-05	0.003239	Mrpl23	DOWN
392.6043	-8.26239	1.993067	-4.14557	3.39E-05	0.003239	Prss23	DOWN
2472.846	-9.60589	2.321028	-4.13863	3.49E-05	0.003269	Eef1akmt1	DOWN
87.48093	-5.27002	1.27399	-4.13662	3.52E-05	0.003269	Gm15501	DOWN
40.6885	-3.70972	0.89724	-4.1346	3.56E-05	0.003269	Nit2	DOWN
1748.97	-9.75934	2.356947	-4.14067	3.46E-05	0.003269	Nop10	DOWN
254.5841	-6.57825	1.590808	-4.13516	3.55E-05	0.003269	Pcna	DOWN
2238.575	-8.12544	1.962829	-4.13966	3.48E-05	0.003269	Sh3gl3	DOWN
6412.634	-8.41536	2.037707	-4.12982	3.63E-05	0.003318	AU022252	DOWN
22.3266	4.216962	1.021843	4.126819	3.68E-05	0.003329	Gm9320	UP
1973.725	-9.36492	2.269521	-4.12639	3.69E-05	0.003329	Spag7	DOWN
58.73568	-2.99668	0.726497	-4.12483	3.71E-05	0.003333	Mcm4	DOWN
5093.221	-8.87592	2.15252	-4.1235	3.73E-05	0.003333	Ndufv3	DOWN
2546.102	7.178972	1.744337	4.115588	3.86E-05	0.00343	Kif5b	UP
2424.092	-8.46613	2.059945	-4.10988	3.96E-05	0.003441	C1qbp	DOWN
86.28574	5.482653	1.333447	4.111641	3.93E-05	0.003441	Gnpnat1	UP
89.73134	-4.98713	1.213504	-4.1097	3.96E-05	0.003441	Rpl15-ps6	DOWN
174.1989	7.524435	1.829185	4.113546	3.90E-05	0.003441	S100a1	UP
1021.64	9.506937	2.315213	4.10629	4.02E-05	0.003473	Gm9012	UP
1930.009	8.740753	2.130479	4.102717	4.08E-05	0.003508	Ntpcr	UP
50.08454	3.315686	0.809753	4.094688	4.23E-05	0.003612	Mbl2	UP
518.0572	-7.5489	1.844986	-4.09158	4.28E-05	0.003641	Pgam1	DOWN
2052.106	-9.00014	2.203513	-4.08445	4.42E-05	0.003734	Gpx1	DOWN
61.81807	-3.59194	0.879989	-4.0818	4.47E-05	0.00374	Pisd	DOWN
2415.258	9.037347	2.214184	4.08157	4.47E-05	0.00374	Tmem129	UP
3176.733	7.171055	1.757754	4.07967	4.51E-05	0.003751	Map4	UP
105.819	-5.64512	1.385689	-4.07388	4.62E-05	0.0038	Pcdhb17	DOWN
2024.77	-8.96813	2.201118	-4.07435	4.61E-05	0.0038	Psmg2	DOWN
4381.671	8.574487	2.106462	4.070564	4.69E-05	0.0038	Stat5a	UP
1683.622	-9.03992	2.220196	-4.07168	4.67E-05	0.0038	Txnl4a	DOWN
2623.163	-8.17362	2.007469	-4.07161	4.67E-05	0.0038	Uchl3	DOWN
366.6296	-8.16237	2.007466	-4.06601	4.78E-05	0.003805	Hspb6	DOWN
1949.887	-9.36648	2.303878	-4.06553	4.79E-05	0.003805	Immp1l	DOWN
3540.993	9.428649	2.319079	4.065686	4.79E-05	0.003805	Mrps11	UP
199.9188	7.56846	1.860861	4.067181	4.76E-05	0.003805	Selenbp1	UP
119.3603	-5.43809	1.339553	-4.05963	4.92E-05	0.003883	Med19	DOWN
2195.734	8.713996	2.147329	4.058062	4.95E-05	0.003889	Def8	UP
2832.609	-7.55978	1.86572	-4.05194	5.08E-05	0.003973	Lyrm4	DOWN
4033.361	7.003469	1.730148	4.047901	5.17E-05	0.004022	Arpc1a	UP
119.4122	-6.12158	1.512843	-4.04641	5.20E-05	0.004028	Xlr5c	DOWN
1678.378	-9.08908	2.247994	-4.0432	5.27E-05	0.004063	Emc4	DOWN
3588.968	7.325432	1.816442	4.032847	5.51E-05	0.004189	Atp9b	UP
45.04498	-3.60595	0.894027	-4.03338	5.50E-05	0.004189	Bet1	DOWN
2397.253	-8.70081	2.157606	-4.03262	5.52E-05	0.004189	Cited1	DOWN
3786.521	6.791705	1.686132	4.027978	5.63E-05	0.004231	Ccar1	UP
2161.144	-8.4451	2.096694	-4.02782	5.63E-05	0.004231	Dctpp1	DOWN
2441.336	7.020138	1.743312	4.026896	5.65E-05	0.004231	Sart1	UP
2495.127	-8.4553	2.101633	-4.02321	5.74E-05	0.004242	Ice2	DOWN

675.2201	-9.0328	2.245313	-4.02296	5.75E-05	0.004242	S100a10	DOWN
3731.183	-8.40444	2.088481	-4.02419	5.72E-05	0.004242	Tomm20	DOWN
31.24694	-3.11486	0.775424	-4.01698	5.89E-05	0.004251	Nme4	DOWN
2211.256	-7.10894	1.768764	-4.01916	5.84E-05	0.004251	Rbm15	DOWN
3071.615	-10.2086	2.541291	-4.01708	5.89E-05	0.004251	Rhox5	DOWN
1723.844	-9.70902	2.416729	-4.01742	5.88E-05	0.004251	Sh3bgrl3	DOWN
1694.857	7.076781	1.76113	4.018319	5.86E-05	0.004251	Smdt1	UP
44.70739	-2.97212	0.740618	-4.01303	5.99E-05	0.004303	Cers2	DOWN
216.6692	6.702451	1.670804	4.011512	6.03E-05	0.004311	Gm28370	UP
1009.202	-8.77975	2.190701	-4.00773	6.13E-05	0.004361	Riok2	DOWN
1203.74	-7.56311	1.888725	-4.00435	6.22E-05	0.004404	Rpl29	DOWN
623.1292	-8.33693	2.085522	-3.99752	6.40E-05	0.004492	Bex4	DOWN
2605.16	8.988782	2.248592	3.997516	6.40E-05	0.004492	Nat14	UP
992.4402	-7.14271	1.788575	-3.99352	6.51E-05	0.004548	Ddx18	DOWN
277.172	-6.09544	1.528665	-3.98742	6.68E-05	0.004646	Tceal9	DOWN
3419.867	-8.95794	2.248272	-3.98436	6.77E-05	0.004686	Gm11426	DOWN
3546.973	-8.77053	2.203262	-3.9807	6.87E-05	0.004738	Fam210b	DOWN
3908.704	-8.80794	2.215548	-3.97551	7.02E-05	0.004771	Dnajb1	DOWN
3610.939	7.356003	1.850619	3.974889	7.04E-05	0.004771	Fos	UP
41.828	-3.32699	0.836939	-3.97519	7.03E-05	0.004771	Polr2j	DOWN
1884.958	-8.61127	2.1662	-3.97529	7.03E-05	0.004771	Ppdpf	DOWN
103.4209	5.838779	1.469593	3.973059	7.10E-05	0.004787	Rnls	UP
2965.466	8.799101	2.216717	3.969429	7.20E-05	0.00484	Maoa	UP
3581.988	8.641752	2.181873	3.960704	7.47E-05	0.004863	Cnpy4	UP
285.1161	-8.69656	2.19628	-3.95968	7.51E-05	0.004863	Gm17251	DOWN
99.0383	-5.96451	1.50599	-3.96052	7.48E-05	0.004863	Gm8663	DOWN
938.2343	-8.61787	2.175147	-3.96197	7.43E-05	0.004863	Itm2a	DOWN
52.90709	-3.79166	0.955814	-3.96694	7.28E-05	0.004863	Pcdh7	DOWN
2162.646	7.167017	1.808874	3.962142	7.43E-05	0.004863	Pebp1	UP
1574.009	-9.52667	2.403622	-3.96347	7.39E-05	0.004863	Rbis	DOWN
3744.14	-8.74571	2.208914	-3.95928	7.52E-05	0.004863	Smoc1	DOWN
3342.894	-8.62975	2.177395	-3.96334	7.39E-05	0.004863	Snrpa1	DOWN
1217.495	-8.8108	2.228512	-3.95367	7.70E-05	0.004878	Cyct	DOWN
83.99803	-4.66868	1.180822	-3.95376	7.69E-05	0.004878	D17H6S53E	DOWN
2604.817	8.92488	2.256718	3.954806	7.66E-05	0.004878	Dctn3	UP
43.00467	-2.75252	0.69562	-3.95693	7.59E-05	0.004878	Lgr6	DOWN
2599.146	9.137114	2.310671	3.954313	7.68E-05	0.004878	Mrpl16	UP
697.353	-6.91074	1.748445	-3.95251	7.73E-05	0.004882	Psmb7	DOWN
2315.931	-6.93056	1.754991	-3.94906	7.85E-05	0.004934	Set	DOWN
155.9723	-5.82895	1.477826	-3.94427	8.00E-05	0.005013	Rps18-ps1	DOWN
1468.141	-8.43515	2.139313	-3.94293	8.05E-05	0.005021	Ostc	DOWN
860.3287	-6.88649	1.747454	-3.94087	8.12E-05	0.005037	Amt	DOWN
5711.198	-8.08162	2.051078	-3.94018	8.14E-05	0.005037	Kcp	DOWN
256.9778	7.675391	1.948392	3.939346	8.17E-05	0.005037	Map2k3os	UP
1554.98	-8.76766	2.226701	-3.93751	8.23E-05	0.005056	Pde6d	DOWN
2093.255	-8.57304	2.17955	-3.9334	8.38E-05	0.005123	Cwc15	DOWN
304.7484	-6.2897	1.599487	-3.93232	8.41E-05	0.005126	Ptpn2	DOWN
6537.484	6.66712	1.698275	3.925818	8.64E-05	0.005246	Ncl	UP
1792.083	-8.61144	2.194445	-3.9242	8.70E-05	0.005261	Mrps12	DOWN
739.5072	-7.50145	1.913926	-3.9194	8.88E-05	0.005319	Ifitm2	DOWN
157.6536	6.173954	1.574919	3.920173	8.85E-05	0.005319	Noxred1	UP
3210.445	-8.50827	2.17113	-3.91882	8.90E-05	0.005319	Tk2	DOWN
3900.485	-8.41345	2.150211	-3.91285	9.12E-05	0.005431	Psma6	DOWN
508.3948	9.249261	2.367682	3.906462	9.37E-05	0.005556	4833412C15Rik	UP

618.8374	9.215551	2.361521	3.902379	9.53E-05	0.005629	Glt28d2	UP
504.7177	-6.32224	1.621968	-3.89788	9.70E-05	0.005713	Scml2	DOWN
2803.894	8.692751	2.233605	3.891803	9.95E-05	0.005727	Ak3	UP
4287.178	8.453528	2.169618	3.896321	9.77E-05	0.005727	Cnpy1	UP
4844.072	-8.32426	2.140034	-3.88978	1.00E-04	0.005727	Mrpl58	DOWN
2874.707	-8.80776	2.264865	-3.88887	1.01E-04	0.005727	Naa38	DOWN
1651.712	8.487876	2.181693	3.890499	1.00E-04	0.005727	Nptx2	UP
1871.151	-8.27723	2.128705	-3.88839	1.01E-04	0.005727	Nsmce4a	DOWN
153.3534	-5.49523	1.413221	-3.88844	1.01E-04	0.005727	Plod2	DOWN
2806.839	6.727019	1.729761	3.888988	1.01E-04	0.005727	Rnf10	UP
48.2101	-3.19621	0.820794	-3.89405	9.86E-05	0.005727	Tmem147	DOWN
1718.389	7.174057	1.841804	3.895125	9.81E-05	0.005727	Tollip	UP
252.8607	7.913973	2.036013	3.886996	1.01E-04	0.00573	1810041H14Rik	UP
172.0819	-4.81169	1.238108	-3.88632	1.02E-04	0.00573	Epcam	DOWN
4944.266	8.080261	2.07951	3.885657	1.02E-04	0.00573	Osgep	UP
1527.66	7.29767	1.87901	3.883785	1.03E-04	0.005753	BC031181	UP
364.3918	-7.68008	1.97836	-3.88204	1.04E-04	0.005754	0610009B22Rik	DOWN
2939.686	8.721014	2.246253	3.882472	1.03E-04	0.005754	Tut1	UP
3129.685	-8.66346	2.232704	-3.88025	1.04E-04	0.005776	Tbata	DOWN
2937.068	-6.66264	1.718723	-3.87651	1.06E-04	0.005845	Gsdme	DOWN
4491.533	7.256895	1.87349	3.873463	1.07E-04	0.005877	Cdc37l1	UP
384.04	8.842589	2.282662	3.873806	1.07E-04	0.005877	Gm48742	UP
2215.292	-8.54474	2.209294	-3.86764	1.10E-04	0.005998	Porcn	DOWN
33.53983	-3.27887	0.848504	-3.86429	1.11E-04	0.00606	Dph3	DOWN
2559.402	8.487493	2.197491	3.862356	1.12E-04	0.006087	Rtf2	UP
2869.921	8.840769	2.290894	3.859091	1.14E-04	0.006089	Ccdc59	UP
2889.26	6.750867	1.748991	3.859864	1.13E-04	0.006089	Lig1	UP
3389.219	-8.41462	2.180551	-3.85894	1.14E-04	0.006089	Prdx6	DOWN
4093.403	8.106763	2.100722	3.859036	1.14E-04	0.006089	Rbak	UP
3259.567	-8.40198	2.178673	-3.85647	1.15E-04	0.006109	Cdc42ep2	DOWN
1450.53	8.469516	2.195737	3.857255	1.15E-04	0.006109	Ogfod1	UP
6323.999	-7.68291	1.993144	-3.85467	1.16E-04	0.006134	Topbp1	DOWN
4130.205	8.369469	2.175121	3.847817	1.19E-04	0.006287	Usp11	UP
117.9334	-5.45758	1.423365	-3.83428	1.26E-04	0.006621	Sdhaf4	DOWN
35.23978	3.230394	0.842721	3.833291	1.26E-04	0.006625	Mon1a	UP
779.4598	-7.23169	1.889705	-3.82689	1.30E-04	0.006755	Mrpl18	DOWN
916.485	-8.46581	2.211903	-3.82739	1.30E-04	0.006755	Zfp414	DOWN
35.78648	-3.63438	0.950345	-3.82427	1.31E-04	0.006805	Ndufa12-ps	DOWN
1744.8	-8.46987	2.217641	-3.81931	1.34E-04	0.006858	Apool	DOWN
2292.684	7.149358	1.870924	3.821297	1.33E-04	0.006858	Ate1	UP
2342.077	-8.59373	2.250197	-3.8191	1.34E-04	0.006858	Cmtm7	DOWN
2953.198	8.549772	2.238406	3.819581	1.34E-04	0.006858	Gm49839	UP
2400.085	-8.41591	2.205267	-3.81628	1.35E-04	0.006867	Aar2	DOWN
2456.217	-7.52955	1.972514	-3.81724	1.35E-04	0.006867	Azi2	DOWN
73.74918	-3.08047	0.807335	-3.8156	1.36E-04	0.006867	Pip4p2	DOWN
1280.44	8.389416	2.198241	3.816422	1.35E-04	0.006867	Thap3	UP
885.6287	9.253869	2.427194	3.812579	1.38E-04	0.006929	Coa3	UP
1835.958	7.161094	1.879119	3.810878	1.38E-04	0.006941	Thra	UP
814.1939	-6.98793	1.833817	-3.81059	1.39E-04	0.006941	Usp14	DOWN
1109.314	-8.36366	2.196208	-3.80823	1.40E-04	0.006969	Ccdc58	DOWN
252.4539	-6.74151	1.770343	-3.80803	1.40E-04	0.006969	Cxcr4	DOWN
4211.244	6.436858	1.690725	3.807158	1.41E-04	0.006971	Maged1	UP
3325.532	-8.04412	2.113717	-3.80568	1.41E-04	0.006991	Gpatch8	DOWN
1422.418	-7.45737	1.960401	-3.804	1.42E-04	0.007016	Tcea3	DOWN

136.6145	-4.46433	1.174429	-3.80128	1.44E-04	0.007068	Rer1	DOWN
1176.966	-8.89851	2.341318	-3.80064	1.44E-04	0.007068	Rfk	DOWN
3231.04	-7.77264	2.045552	-3.79978	1.45E-04	0.00707	Prdx4	DOWN
3061.925	6.504386	1.712586	3.797991	1.46E-04	0.007099	Mrfap1	UP
3307.514	8.174562	2.154811	3.793632	1.48E-04	0.007144	E2f2	UP
47.43233	-2.79634	0.737162	-3.79339	1.49E-04	0.007144	Gde1	DOWN
78.07012	-4.22124	1.112588	-3.79407	1.48E-04	0.007144	Sema3e	DOWN
4053.433	6.892286	1.81638	3.794519	1.48E-04	0.007144	Tax1bp1	UP
3397.836	-8.10387	2.138116	-3.79019	1.51E-04	0.007214	Gfer	DOWN
1990.802	8.395227	2.216521	3.787568	1.52E-04	0.007229	3110070M22Rik	UP
1006.368	-7.9474	2.098532	-3.78712	1.52E-04	0.007229	Enoph1	DOWN
482.2006	-6.95636	1.836766	-3.78729	1.52E-04	0.007229	Gabarapl2	DOWN
1832.233	-8.48233	2.240476	-3.78595	1.53E-04	0.007229	Krtcap2	DOWN
2991.221	-9.20587	2.431431	-3.78619	1.53E-04	0.007229	Rab4a	DOWN
184.0204	-5.47361	1.446813	-3.78322	1.55E-04	0.007286	Smndc1	DOWN
6781.474	-7.58326	2.006081	-3.78014	1.57E-04	0.007355	Tsc22d1	DOWN
691.9337	8.1941	2.168909	3.777983	1.58E-04	0.007397	Gm2479	UP
4298.974	8.055629	2.133414	3.775933	1.59E-04	0.007436	Rcbtb2	UP
782.1419	-8.43979	2.237723	-3.7716	1.62E-04	0.007455	Cox7a2l	DOWN
38.96228	-2.83425	0.752003	-3.76893	1.64E-04	0.007455	Ddx4	DOWN
2881.042	8.277948	2.197336	3.767266	1.65E-04	0.007455	Dhrs7b	UP
3621.388	-7.8073	2.070922	-3.76996	1.63E-04	0.007455	Ergic1	DOWN
2015.394	7.226169	1.916002	3.771482	1.62E-04	0.007455	Erlec1	UP
1672.395	8.722426	2.314603	3.768433	1.64E-04	0.007455	F2r	UP
248.3114	-7.38097	1.959154	-3.76743	1.65E-04	0.007455	Gm8615	DOWN
217.4591	-5.71882	1.517949	-3.76747	1.65E-04	0.007455	H2ax	DOWN
2212.072	8.218048	2.181209	3.767656	1.65E-04	0.007455	Sohlh1	UP
1895.354	-8.27341	2.194714	-3.7697	1.63E-04	0.007455	Tipin	DOWN
699.8269	-7.74919	2.05333	-3.77396	1.61E-04	0.007455	Tmed4	DOWN
196.3291	6.126357	1.626528	3.766524	1.66E-04	0.007456	Cyb561d2	UP
46.53918	-2.70571	0.71892	-3.76358	1.67E-04	0.007499	Ctdspl	DOWN
1893.256	-8.24303	2.190574	-3.76295	1.68E-04	0.007499	Dars	DOWN
3374.983	-8.29546	2.204087	-3.76367	1.67E-04	0.007499	Wdr6	DOWN
482.5971	-7.56024	2.011228	-3.75902	1.71E-04	0.007596	Spdya	DOWN
81.4568	-3.61481	0.962416	-3.75597	1.73E-04	0.007668	Slc25a5	DOWN
1524.69	7.194659	1.918492	3.750164	1.77E-04	0.007733	Atxn7l3b	UP
3946.057	6.233884	1.662795	3.74904	1.78E-04	0.007733	Cct2	UP
3859.567	6.558347	1.748844	3.750103	1.77E-04	0.007733	Dcp1a	UP
1660.345	8.551077	2.281747	3.747601	1.79E-04	0.007733	Ifi27	UP
654.6778	-7.72812	2.062031	-3.74782	1.78E-04	0.007733	Naa11	DOWN
1997.873	-7.93494	2.115685	-3.75053	1.76E-04	0.007733	Nup43	DOWN
1840.307	6.903073	1.84202	3.747557	1.79E-04	0.007733	Slc20a1	UP
2716.197	8.036472	2.142718	3.750597	1.76E-04	0.007733	Zfp346	UP
2679.128	8.427826	2.248673	3.747911	1.78E-04	0.007733	Zfp994	UP
2588.361	8.797277	2.349401	3.744476	1.81E-04	0.007804	Gm14137	UP
3700.365	-7.8845	2.105976	-3.74387	1.81E-04	0.007804	Htatsf1	DOWN
1822.985	-8.36111	2.234314	-3.74214	1.82E-04	0.007816	Fbxo22	DOWN
2491.973	-8.2505	2.204567	-3.74246	1.82E-04	0.007816	Nipal2	DOWN
1505.566	8.126824	2.173044	3.739834	1.84E-04	0.007866	Itm2c	UP
403.7931	7.24532	1.939238	3.736168	1.87E-04	0.007941	Ifngr1	UP
3163.043	-7.78328	2.083278	-3.73607	1.87E-04	0.007941	Mtx2	DOWN
38.22527	2.555483	0.684293	3.734485	1.88E-04	0.00797	Gm10390	UP
5181.032	-7.92547	2.126106	-3.72769	1.93E-04	0.008096	Alg5	DOWN
996.7216	8.214736	2.204365	3.726577	1.94E-04	0.008096	Fam110c	UP

1318.656	8.12502	2.180133	3.726846	1.94E-04	0.008096	Lsm7	UP
511.176	8.196809	2.1996	3.726499	1.94E-04	0.008096	Mcee	UP
177.9423	-6.23057	1.670662	-3.7294	0.000192	0.008096	Mettl15	DOWN
460.2378	7.578593	2.033648	3.7266	1.94E-04	0.008096	Mss51	UP
418.1758	-8.15034	2.188563	-3.72406	1.96E-04	0.008153	Snrpf	DOWN
85.64534	-4.418	1.186793	-3.72263	1.97E-04	0.008178	Tdh	DOWN
1109.469	8.279915	2.224807	3.721632	1.98E-04	0.008188	6430511E19Rik	UP
4003.105	7.722839	2.07674	3.718732	2.00E-04	0.008261	Hadha	UP
1198.964	8.252702	2.222149	3.713839	2.04E-04	0.008401	Gm15751	UP
1178.445	-8.45185	2.276291	-3.71299	2.05E-04	0.008407	Dynlrb1	DOWN
1092.555	8.756615	2.361991	3.707302	0.000209	0.008446	1700061I17Rik	UP
989.0954	-6.77516	1.82616	-3.71006	2.07E-04	0.008446	Arf4	DOWN
3411.018	-7.84089	2.115023	-3.70724	2.10E-04	0.008446	Klhl22	DOWN
394.7541	-6.79347	1.831284	-3.70968	2.08E-04	0.008446	Npat	DOWN
1803.956	-8.7665	2.363627	-3.70892	2.08E-04	0.008446	Rasl10a	DOWN
1965.334	6.771362	1.825332	3.709661	2.08E-04	0.008446	Rngtt	UP
313.8633	-5.384	1.452132	-3.70765	2.09E-04	0.008446	Snap23	DOWN
3120.804	7.913679	2.136254	3.704466	2.12E-04	0.008517	Srpr	UP
1457.257	-6.66292	1.79901	-3.70366	2.13E-04	0.008522	Skil	DOWN
354.617	-7.17114	1.938951	-3.69847	2.17E-04	0.008567	4933415A04Rik	DOWN
47.86852	3.218543	0.869716	3.700684	2.15E-04	0.008567	Cd200	UP
96.55654	-4.52454	1.222757	-3.70028	2.15E-04	0.008567	Lpar1	DOWN
2715.312	-8.27651	2.237485	-3.69902	2.16E-04	0.008567	Slc25a17	DOWN
1565.012	8.239408	2.22595	3.701525	2.14E-04	0.008567	Slc50a1	UP
463.4337	7.61316	2.057823	3.699619	2.16E-04	0.008567	Tssc4	UP
2463.249	8.42912	2.280623	3.695972	2.19E-04	0.008609	Cd320	UP
1663.349	7.003932	1.894759	3.696477	2.19E-04	0.008609	Pdcd7	UP
1820.5	-6.57607	1.781192	-3.69195	2.23E-04	0.008724	Mest	DOWN
2166.029	-7.78565	2.110421	-3.68914	2.25E-04	0.008777	Mettl9	DOWN
1146.647	-6.46224	1.751416	-3.68973	2.24E-04	0.008777	Zc3hav1	DOWN
5487.077	-7.66641	2.078468	-3.68849	2.26E-04	0.008778	Zfp292	DOWN
1542.803	8.293767	2.249846	3.686371	2.27E-04	0.008829	1700028K03Rik	UP
3352.93	-6.55693	1.779376	-3.68496	2.29E-04	0.008857	Gnb1	DOWN
2974.525	-7.93817	2.154985	-3.68363	2.30E-04	0.008865	Timm17b	DOWN
724.6549	-7.34454	1.993929	-3.68345	2.30E-04	0.008865	Tusc3	DOWN
4490.218	-6.89674	1.873481	-3.68124	2.32E-04	0.008901	Alg13	DOWN
369.0053	7.233299	1.964944	3.681173	2.32E-04	0.008901	Rnf113a2	UP
133.4964	-4.52546	1.230541	-3.67762	2.35E-04	0.009005	Snx4	DOWN
151.5769	5.980434	1.626745	3.67632	2.37E-04	0.009028	Mm2pr	UP
224.708	-4.87229	1.325589	-3.67557	2.37E-04	0.009033	Timmdc1	DOWN
1036.827	-7.9685	2.16867	-3.67437	2.38E-04	0.009054	Bub1	DOWN
4416.418	-6.66515	1.814926	-3.67241	2.40E-04	0.009101	Smc6	DOWN
5858.189	-6.70035	1.825082	-3.67126	2.41E-04	0.009121	Srsf5	DOWN
526.0238	7.458188	2.032029	3.670316	2.42E-04	0.009132	Olfr1383	UP
42726.79	-3.41525	0.930837	-3.66901	2.43E-04	0.009157	mt-Cytb	DOWN
1333.663	8.356257	2.280786	3.663762	2.49E-04	0.009236	Ccdc181	UP
2592.421	-7.89701	2.155137	-3.66427	2.48E-04	0.009236	Cfl2	DOWN
1231.228	-7.97487	2.175429	-3.66589	2.46E-04	0.009236	Cldn7	DOWN
98.10449	3.622863	0.988619	3.664569	2.48E-04	0.009236	Gm28439	UP
575.5296	-7.88314	2.150801	-3.66521	2.47E-04	0.009236	Tmem42	DOWN
1654.793	-7.71411	2.105979	-3.66296	2.49E-04	0.009244	Ifitm1	DOWN
1297.22	8.375475	2.286986	3.662233	2.50E-04	0.009248	Cdc42ep5	UP
2360.173	-7.54618	2.061536	-3.66046	2.52E-04	0.009289	Ddx39b	DOWN
970.6875	-6.39987	1.748648	-3.65989	2.52E-04	0.009289	Gsk3b	DOWN

2108.246	8.267895	2.260592	3.657402	2.55E-04	0.009358	Ifi35	UP
1900.059	6.448074	1.763637	3.656123	2.56E-04	0.009369	Ccdc88a	UP
21.82752	-3.95137	1.080819	-3.6559	2.56E-04	0.009369	Rpl21-ps7	DOWN
819.5441	7.717184	2.111264	3.655244	2.57E-04	0.009371	Gm44997	UP
2047.439	6.665339	1.824627	3.652988	2.59E-04	0.009432	Wasf2	UP
3919.986	7.457859	2.042688	3.651002	2.61E-04	0.009484	Hook1	UP
1043.037	-7.03456	1.927234	-3.65008	2.62E-04	0.009496	Sec61g	DOWN
801.243	-6.42966	1.761857	-3.64937	2.63E-04	0.0095	Cycs	DOWN
1296.75	6.563773	1.800959	3.644599	2.68E-04	0.009615	Chordc1	UP
28.90192	3.533086	0.969427	3.644511	2.68E-04	0.009615	Gm6222	UP
2211.7	6.750659	1.851712	3.645632	2.67E-04	0.009615	Tcf7l2	UP
2111.746	-8.31086	2.28137	-3.64292	2.70E-04	0.009653	Caap1	DOWN
1311.469	-8.12785	2.232141	-3.64128	2.71E-04	0.009668	Gm50322	DOWN
1012.418	-8.1092	2.227315	-3.6408	2.72E-04	0.009668	Lum	DOWN
311.3479	6.598081	1.812287	3.640747	2.72E-04	0.009668	Topaz1	UP
3739.679	6.811487	1.871949	3.638715	2.74E-04	0.009723	Polr2a	UP
3624.14	-8.25109	2.26955	-3.63556	2.77E-04	0.009821	Tmem50a	DOWN
2657.009	8.078167	2.222773	3.634274	2.79E-04	0.009848	Ndufaf1	UP
310.6396	-6.50198	1.79203	-3.62828	2.85E-04	0.010057	Cks1b	DOWN
667.6328	7.34191	2.023976	3.62747	2.86E-04	0.010065	Pycr1	UP
2855.928	-7.95677	2.196488	-3.6225	2.92E-04	0.010238	Ndc1	DOWN
1482.568	-7.47208	2.063261	-3.62149	2.93E-04	0.010247	Drg1	DOWN
65.00966	3.453389	0.953681	3.621115	2.93E-04	0.010247	Gps2	UP
1015.911	8.091051	2.236044	3.618467	2.96E-04	0.010307	Apold1	UP
1237.176	7.961226	2.200147	3.618497	2.96E-04	0.010307	Foxa2	UP
1144.315	-7.95145	2.197972	-3.61763	2.97E-04	0.010317	Irak1bp1	DOWN
20.0617	3.011261	0.832535	3.61698	2.98E-04	0.01032	Olfr1341	UP
80.34267	-3.97002	1.097887	-3.61605	2.99E-04	0.010335	Slc39a8	DOWN
1614.927	6.459446	1.787136	3.614413	3.01E-04	0.010355	Map7d2	UP
1391.007	-6.43899	1.781214	-3.61495	3.00E-04	0.010355	Spats2	DOWN
1329.3	7.880798	2.180844	3.613647	3.02E-04	0.010363	Slc25a24	UP
50.30435	-3.49693	0.967979	-3.6126	3.03E-04	0.010382	Zfp985	DOWN
2612.553	-7.87617	2.182946	-3.60804	3.09E-04	0.010499	1500011B03Rik	DOWN
2106.059	-7.86965	2.180716	-3.60875	3.08E-04	0.010499	Fbxo8	DOWN
985.5219	7.876371	2.183026	3.608006	3.09E-04	0.010499	Gm38126	UP
5165.157	7.82424	2.168949	3.607388	3.09E-04	0.010501	Gabarap	UP
644.3444	6.256538	1.735058	3.605954	3.11E-04	0.010532	Fkbp14	UP
266.6471	-6.75766	1.874556	-3.60494	3.12E-04	0.010532	Rab4b	DOWN
1916.686	7.669418	2.127744	3.604484	3.13E-04	0.010532	Susd6	UP
303.835	-6.41164	1.778836	-3.6044	3.13E-04	0.010532	Wdr74	DOWN
26.28849	3.289376	0.91339	3.601283	3.17E-04	0.010636	Spin4	UP
29.77909	3.189821	0.885903	3.600646	3.17E-04	0.01064	Gm47026	UP
1462.634	-8.04616	2.235783	-3.59881	3.20E-04	0.010692	Ccnh	DOWN
1330.046	-8.31493	2.313078	-3.59475	3.25E-04	0.010814	Cmbl	DOWN
1229.527	-7.98271	2.220651	-3.59476	3.25E-04	0.010814	Rogdi	DOWN
44506.25	-3.13062	0.871429	-3.59251	3.28E-04	0.010884	mt-Co1	DOWN
3136.326	-7.4045	2.062643	-3.58981	3.31E-04	0.010952	Dus1l	DOWN
92.43082	4.680669	1.303869	3.589832	3.31E-04	0.010952	Lpar4	UP
23.5794	-2.98669	0.832932	-3.58575	3.36E-04	0.0111	Gucy2f	DOWN
555.0161	-7.4314	2.073953	-3.58321	3.39E-04	0.011162	Aldh3a2	DOWN
983.1047	-7.86625	2.195003	-3.58371	3.39E-04	0.011162	Gm45501	DOWN
1748.345	-7.88751	2.202486	-3.58119	3.42E-04	0.01122	Ccdc66	DOWN
2006.205	-7.9102	2.20937	-3.5803	3.43E-04	0.01122	Ndufa9	DOWN
4030.087	-7.7459	2.163529	-3.58021	3.43E-04	0.01122	Tbc1d4	DOWN

819.6557	-8.01631	2.239723	-3.57915	3.45E-04	0.011242	Enpp4	DOWN
2910.503	7.867801	2.199561	3.576987	3.48E-04	0.011312	Tmem205	UP
1370.104	7.966961	2.228154	3.575589	3.49E-04	0.01135	Avpi1	UP
2574.886	-7.79567	2.181493	-3.57355	3.52E-04	0.011415	Sypl	DOWN
156.4561	-5.21503	1.461261	-3.56885	3.59E-04	0.011479	C1ra	DOWN
1932.528	7.739153	2.167903	3.56988	3.57E-04	0.011479	Cgnl1	UP
860.0061	7.44334	2.084117	3.57146	3.55E-04	0.011479	Dolk	UP
80.89367	3.683122	1.031437	3.570867	3.56E-04	0.011479	Mitf	UP
1999.558	-7.2462	2.030204	-3.5692	3.58E-04	0.011479	Mrps14	DOWN
3844.286	-7.46459	2.090761	-3.57027	3.57E-04	0.011479	Ttc14	DOWN
2503.225	-7.62352	2.136653	-3.56797	3.60E-04	0.011495	Spc24	DOWN
90.19128	-3.92607	1.100811	-3.56652	3.62E-04	0.011526	Eif4e3	DOWN
2910.167	-7.68354	2.154545	-3.5662	3.62E-04	0.011526	Oxa1l	DOWN
1328.051	8.495807	2.383734	3.564076	3.65E-04	0.011553	4930513D17Rik	UP
1073.522	-7.595	2.130663	-3.56462	3.64E-04	0.011553	Cnot6	DOWN
2231.546	7.985695	2.240654	3.564003	3.65E-04	0.011553	Ubl5	UP
1753.498	-7.45773	2.093218	-3.56281	3.67E-04	0.011582	Mob4	DOWN
138.857	5.996107	1.684266	3.560072	3.71E-04	0.011652	Gm9347	UP
2491.15	7.820629	2.197017	3.559657	3.71E-04	0.011652	Ift20	UP
1982.522	-8.05826	2.263199	-3.56056	3.70E-04	0.011652	Npl	DOWN
8688.706	-5.19316	1.459308	-3.55865	3.73E-04	0.011666	B4galt1	DOWN
2571.943	-7.79394	2.190367	-3.55828	3.73E-04	0.011666	Fkbp8	DOWN
568.5802	7.474122	2.101207	3.557061	3.75E-04	0.011697	Mgp	UP
197.4191	-6.01167	1.69134	-3.55438	3.79E-04	0.011763	Gypc	DOWN
1291.973	7.961791	2.240209	3.554039	3.79E-04	0.011763	Snapc2	UP
689.8411	-7.60576	2.139441	-3.55502	3.78E-04	0.011763	Thumpd2	DOWN
1718.764	-7.58962	2.136231	-3.55281	3.81E-04	0.011771	Ap3b1	DOWN
1387.271	-7.58079	2.133557	-3.55312	3.81E-04	0.011771	Chmp1a	DOWN
784.935	-7.3118	2.058836	-3.55142	3.83E-04	0.01181	Snx2	DOWN
1831.453	7.483442	2.108002	3.550016	3.85E-04	0.01185	Ctsd	UP
769.6933	6.302277	1.77556	3.549459	3.86E-04	0.011852	Ybx2	UP
4220.756	7.477615	2.107406	3.548255	3.88E-04	0.011883	Shroom4	UP
2681.456	7.840032	2.2105	3.546724	3.90E-04	0.011929	Rabggta	UP
1592.81	8.065935	2.274914	3.545599	3.92E-04	0.011957	Zfp940	UP
1752.589	6.488339	1.831507	3.542623	3.96E-04	0.011996	Calr	UP
2214.016	-7.2568	2.048523	-3.54245	3.96E-04	0.011996	Eif4e	DOWN
1516.324	-7.44564	2.101325	-3.5433	3.95E-04	0.011996	Ndufa13	DOWN
55.67023	-2.98937	0.84393	-3.5422	3.97E-04	0.011996	Plcl2	DOWN
386.8063	-7.23932	2.043488	-3.54263	3.96E-04	0.011996	Ska2	DOWN
1528.609	-6.40946	1.810678	-3.53981	4.00E-04	0.012082	Fubp3	DOWN
123.4141	-4.50929	1.275497	-3.53532	4.07E-04	0.012131	Atox1	DOWN
114.7018	5.301216	1.499416	3.53552	4.07E-04	0.012131	Cfap298	UP
71.18164	-3.62613	1.025155	-3.53715	4.04E-04	0.012131	Ctu1	DOWN
62.90046	-3.14933	0.890847	-3.53521	4.07E-04	0.012131	Sacm1l	DOWN
2362.71	-7.99747	2.262192	-3.53527	4.07E-04	0.012131	Tsen34	DOWN
51.13083	-2.46964	0.698507	-3.5356	4.07E-04	0.012131	Ube2l3	DOWN
35.87811	2.969218	0.839546	3.536695	4.05E-04	0.012131	Zfp771	UP
4199.886	7.614543	2.155084	3.533293	4.10E-04	0.012173	Maged2	UP
1293.107	7.906692	2.237718	3.533374	4.10E-04	0.012173	Necap2	UP
27.23702	-2.62727	0.744511	-3.52886	4.17E-04	0.012332	Entpd5	DOWN
904.5859	7.953092	2.253509	3.529203	4.17E-04	0.012332	Snhg12	UP
913.9041	-7.51267	2.1296	-3.52774	4.19E-04	0.012362	Rundc3b	DOWN
1759.093	-7.80826	2.213758	-3.52715	4.20E-04	0.012366	Rpain	DOWN
3404.114	6.596218	1.871005	3.525495	4.23E-04	0.012395	Enah	UP

33.39175	-2.46213	0.698279	-3.526	4.22E-04	0.012395	Fam120b	DOWN
581.4834	7.449776	2.113681	3.524551	4.24E-04	0.012395	Gm48890	UP
1054.512	7.751963	2.199282	3.52477	4.24E-04	0.012395	Rab24	UP
1330.452	-7.73206	2.195533	-3.52172	4.29E-04	0.012505	Slc35e3	DOWN
2622.845	7.751748	2.203142	3.518497	4.34E-04	0.012529	Sarm1	UP
2279.945	-7.31886	2.080008	-3.51867	4.34E-04	0.012529	Selenow	DOWN
2252.443	7.74442	2.200553	3.519306	4.33E-04	0.012529	Stk4	UP
3562.481	7.541523	2.142437	3.520067	4.31E-04	0.012529	Ttc19	UP
54.92975	-2.75177	0.782136	-3.51827	4.34E-04	0.012529	Unc50	DOWN
1701.4	-7.51502	2.135419	-3.51923	4.33E-04	0.012529	Vdac3	DOWN
1605.09	-7.62818	2.169111	-3.51673	4.37E-04	0.012575	Cdkn2aip	DOWN
2130.828	-6.67434	1.898103	-3.51632	4.38E-04	0.012575	Napa	DOWN
180.6066	-4.24991	1.208807	-3.51579	4.38E-04	0.012578	Capza2	DOWN
878.0054	7.628283	2.17012	3.515144	4.40E-04	0.012585	Hexim1	UP
936.5177	-6.34003	1.80455	-3.51336	4.42E-04	0.012646	Armc1	DOWN
149.6019	5.107165	1.453836	3.512889	4.43E-04	0.012646	Slc2a4rg-ps	UP
57.55586	-2.50114	0.712427	-3.51074	4.47E-04	0.012717	Atg12	DOWN
2656.752	7.70362	2.194489	3.510439	4.47E-04	0.012717	Marchf9	UP
2659.164	8.969844	2.557353	3.507471	4.52E-04	0.012837	Gm49745	UP
1785.998	6.274768	1.789804	3.505841	4.55E-04	0.012892	Mapk8	UP
3571.342	7.447173	2.128267	3.499172	4.67E-04	0.013031	Bgn	UP
2017.119	7.605285	2.172989	3.499919	4.65E-04	0.013031	Cpsf2	UP
759.3894	-7.22444	2.064082	-3.50007	4.65E-04	0.013031	Fst	DOWN
2359.864	7.741486	2.21127	3.500923	4.64E-04	0.013031	Gm10638	UP
1577.891	-7.61372	2.175677	-3.49947	4.66E-04	0.013031	Nudt13	DOWN
618.2236	-7.965	2.27616	-3.49931	4.66E-04	0.013031	Ormdl1	DOWN
315.862	7.192351	2.05354	3.502415	4.61E-04	0.013031	Polr3gl	UP
330.0637	-5.17886	1.47965	-3.50006	4.65E-04	0.013031	Serf2	DOWN
1983.547	8.185718	2.340709	3.497111	4.70E-04	0.013069	Dnajc24	UP
22.43816	-3.10634	0.88829	-3.49699	4.71E-04	0.013069	Gm7292	DOWN
3280.859	-7.06075	2.018787	-3.49752	4.70E-04	0.013069	Zcrb1	DOWN
1901.509	7.746528	2.21692	3.494275	4.75E-04	0.013179	Clpx	UP
1394.999	-7.69673	2.203787	-3.4925	4.79E-04	0.013233	Hormad2	DOWN
3095.032	7.802888	2.23435	3.49224	4.79E-04	0.013233	Phkb	UP
350.8473	6.849507	1.962438	3.490305	4.82E-04	0.013285	Enpp5	UP
1992.525	-7.50128	2.149211	-3.49025	4.83E-04	0.013285	Selenof	DOWN
1547.868	-7.86587	2.253991	-3.48975	4.83E-04	0.013287	Pbk	DOWN
3000.67	-6.75999	1.937408	-3.48919	4.84E-04	0.013292	Atp5c1	DOWN
142.7641	5.097302	1.462588	3.485126	4.92E-04	0.013425	Gm45205	UP
75.73623	-4.41143	1.265705	-3.48535	4.91E-04	0.013425	Pdgfrl	DOWN
854.424	-7.19732	2.06461	-3.48604	4.90E-04	0.013425	Ywhaq	DOWN
2581.213	-6.11591	1.755496	-3.48387	4.94E-04	0.013442	Brd4	DOWN
2872.378	-7.54811	2.166576	-3.48389	4.94E-04	0.013442	Dnajc5	DOWN
462.4133	6.931734	1.991059	3.48143	4.99E-04	0.013541	Ccdc105	UP
165.9629	-4.42424	1.271724	-3.47893	5.03E-04	0.013644	Ap3s1	DOWN
1327.04	-6.99221	2.011852	-3.47551	5.10E-04	0.013736	4833439L19Rik	DOWN
1001.287	7.756631	2.231456	3.476041	5.09E-04	0.013736	Ces2f	UP
2327.346	-7.35965	2.117709	-3.47529	5.10E-04	0.013736	Gpt2	DOWN
1263.343	7.766099	2.234466	3.475595	5.10E-04	0.013736	Gstm7	UP
2345.237	-7.54022	2.170891	-3.47333	5.14E-04	0.013813	Atf4	DOWN
5666.817	-5.96494	1.717637	-3.47276	5.15E-04	0.013819	Nop56	DOWN
1421.805	7.551892	2.175516	3.471311	5.18E-04	0.013823	Gtf2f1	UP
2670.633	-7.73116	2.227068	-3.47145	5.18E-04	0.013823	Ppih	DOWN
1234.445	7.517562	2.16538	3.471706	5.17E-04	0.013823	Snrnp25	UP

3594.071	7.183934	2.070109	3.470317	5.20E-04	0.013827	Plcg2	UP
200.4157	-5.70009	1.642319	-3.47076	5.19E-04	0.013827	Thoc1	DOWN
1951.07	7.93877	2.288448	3.469063	5.22E-04	0.013869	Ddx28	UP
2505.629	7.457768	2.150312	3.468226	5.24E-04	0.013888	Itga4	UP
209.4358	6.589984	1.900374	3.467731	5.25E-04	0.013891	Pctp	UP
2822.507	-7.4805	2.15776	-3.46679	5.27E-04	0.013916	Cpsf6	DOWN
2414.759	7.64383	2.205743	3.465421	5.29E-04	0.013963	Ttc27	UP
17.2719	-3.41908	0.987194	-3.46344	5.33E-04	0.013996	Gm8355	DOWN
24.17	2.67416	0.772088	3.463543	5.33E-04	0.013996	Olfr54	UP
511.7722	5.90177	1.703908	3.463667	5.33E-04	0.013996	Ppp2r3c	UP
25.35692	-3.54262	1.023033	-3.46286	5.34E-04	0.014001	Bnip3	DOWN
795.66	-7.35192	2.123327	-3.46245	5.35E-04	0.014001	Otud6b	DOWN
5817.728	-7.10845	2.0541	-3.46062	5.39E-04	0.014073	Eea1	DOWN
4855.223	-6.2195	1.797646	-3.4598	5.41E-04	0.014092	Psma3	DOWN
146.87	4.927529	1.424792	3.458419	5.43E-04	0.014142	Atxn3	UP
2372.585	7.832158	2.265628	3.456948	5.46E-04	0.014196	Crabp1	UP
1978.068	7.710277	2.232792	3.4532	5.54E-04	0.014371	Wwtr1	UP
329.0919	7.458067	2.160052	3.452726	5.55E-04	0.014371	Gm16573	UP
1078.055	-6.15728	1.783541	-3.45228	5.56E-04	0.014371	Lonrf2	DOWN
10194.61	4.895843	1.418319	3.451864	5.57E-04	0.014371	Qrich1	UP
52.72599	-2.81151	0.814809	-3.45052	5.60E-04	0.014419	Trnt1	DOWN
78.84614	-3.81004	1.104607	-3.44923	5.62E-04	0.01443	Gm15772	DOWN
317.8516	6.056357	1.755577	3.449782	5.61E-04	0.01443	Taf15	UP
1765.367	7.849078	2.275768	3.44898	5.63E-04	0.01443	Tmem39b	UP
1700.778	7.442468	2.158894	3.447352	5.66E-04	0.014444	Fntb	UP
783.4616	-7.55932	2.193604	-3.44608	5.69E-04	0.014444	Mkrn3	DOWN
2122.174	7.526606	2.184373	3.44566	5.70E-04	0.014444	Mtmr1	UP
1533.452	6.909617	2.005071	3.446071	5.69E-04	0.014444	Prpf4	UP
894.4468	6.836985	1.983264	3.44734	5.66E-04	0.014444	Rab12	UP
2778.072	-7.4481	2.161139	-3.44638	5.68E-04	0.014444	Stn1	DOWN
1424.304	-6.87256	1.993169	-3.44806	5.65E-04	0.014444	Zfand6	DOWN
3166.351	8.119206	2.357831	3.443506	5.74E-04	0.014513	Ctxn1	UP
885.1306	-7.25448	2.106448	-3.44394	5.73E-04	0.014513	Pdia4	DOWN
3534.176	7.111795	2.066645	3.441227	5.79E-04	0.014612	Dnajc21	UP
1698.611	7.382783	2.145804	3.440568	5.80E-04	0.014624	Foxk2	UP
2070.066	-7.70816	2.242434	-3.43741	5.87E-04	0.014749	Rps6kb1	DOWN
348.261	-5.13329	1.493364	-3.4374	5.87E-04	0.014749	Twf1	DOWN
39.06716	-2.30196	0.669845	-3.43655	5.89E-04	0.014772	Gemin7	DOWN
288.7102	-5.85056	1.703658	-3.43412	5.94E-04	0.014787	Abhd17b	DOWN
214.126	-5.86078	1.706354	-3.43468	5.93E-04	0.014787	Gm32123	DOWN
20.80126	-3.61474	1.052379	-3.43483	5.93E-04	0.014787	Gm9294	DOWN
431.1953	-6.39607	1.862482	-3.43417	5.94E-04	0.014787	Mpc1	DOWN
2707.125	7.29096	2.122362	3.435305	5.92E-04	0.014787	Scaper	UP
4278.789	-7.21094	2.100186	-3.43348	5.96E-04	0.014798	Pttg1	DOWN
2290.302	7.490353	2.182555	3.43192	5.99E-04	0.01486	Bag4	UP
51.44661	-2.81563	0.820637	-3.43103	6.01E-04	0.014885	Fam162a	DOWN
3094.461	7.510086	2.189251	3.430437	6.03E-04	0.014895	Eloa	UP
18.13727	-3.56825	1.040426	-3.4296	6.04E-04	0.014917	2700078F05Rik	DOWN
1380.905	-7.51173	2.190782	-3.42879	6.06E-04	0.014939	Gdap2	DOWN
77.76771	-3.82644	1.116544	-3.42704	6.10E-04	0.014988	Nek2	DOWN
1165.744	7.837515	2.28683	3.42724	6.10E-04	0.014988	Zfp830	UP
1600.853	7.429067	2.169545	3.424251	6.16E-04	0.015119	Dhx8	UP
1663.687	7.386499	2.157556	3.42355	6.18E-04	0.015135	Spcs1	UP
538.0639	-6.53164	1.908495	-3.4224	6.21E-04	0.015175	Nup37	DOWN

1010.238	7.645842	2.235382	3.420374	6.25E-04	0.015265	Prss35	UP
620.2775	-7.11821	2.082033	-3.41887	6.29E-04	0.015303	Cmc4	DOWN
1461.809	-7.651	2.23789	-3.41885	6.29E-04	0.015303	Mettl25	DOWN
1750.44	7.590695	2.22101	3.417677	6.32E-04	0.015345	Paf1	UP
852.6433	-7.18409	2.103766	-3.41487	6.38E-04	0.015456	Arrdc4	DOWN
1646.45	-7.86462	2.303027	-3.41491	6.38E-04	0.015456	Mis18a	DOWN
803.3813	-7.56012	2.215194	-3.41285	6.43E-04	0.015463	1700013H16Rik	DOWN
929.5963	-7.25237	2.125137	-3.41266	6.43E-04	0.015463	D130040H23Rik	DOWN
208.6745	-5.76036	1.68732	-3.41391	6.40E-04	0.015463	Il33	DOWN
13.40037	3.83728	1.123964	3.414059	6.40E-04	0.015463	Nfkbid	UP
506.5703	-7.0609	2.06892	-3.41284	6.43E-04	0.015463	Rpa3	DOWN
190.0913	-5.70401	1.671661	-3.41218	6.44E-04	0.015467	Mrpl22	DOWN
19.03108	2.943233	0.862722	3.411567	6.46E-04	0.015478	Urod	UP
6637.812	-5.56941	1.633564	-3.40936	6.51E-04	0.01558	Dynll1	DOWN
1834.363	-7.60408	2.230894	-3.40854	6.53E-04	0.015603	Stx1b	DOWN
1553.901	6.114183	1.794446	3.407282	6.56E-04	0.015628	Srrt	UP
3013.198	-7.27626	2.135314	-3.40758	6.55E-04	0.015628	Ube2o	DOWN
1840.074	-7.90729	2.322918	-3.40403	6.64E-04	0.015671	1500009L16Rik	DOWN
2327.779	5.949846	1.747399	3.404973	6.62E-04	0.015671	Arid4b	UP
28.47484	-2.42736	0.7131	-3.40396	6.64E-04	0.015671	Cc2d2a	DOWN
123.0268	-5.45937	1.603805	-3.40401	6.64E-04	0.015671	Gm4864	DOWN
1221.025	-7.52962	2.212222	-3.40364	6.65E-04	0.015671	Maip1	DOWN
1294.511	-7.32109	2.150036	-3.4051	6.61E-04	0.015671	Papss1	DOWN
27.76089	-2.754	0.808689	-3.40551	6.60E-04	0.015671	Tmem183a	DOWN
1718.703	7.978314	2.344766	3.402605	6.67E-04	0.015707	Arf2	UP
633.8511	-6.53291	1.922312	-3.39847	6.78E-04	0.015923	Gcsh	DOWN
1886.755	7.71155	2.269937	3.397253	6.81E-04	0.015946	Mrps7	UP
1432.849	6.05068	1.780842	3.397651	6.80E-04	0.015946	Neurl4	UP
327.0077	-5.01894	1.478191	-3.39533	6.85E-04	0.015998	Arhgap44	DOWN
3582.95	7.137543	2.10195	3.395677	6.85E-04	0.015998	Myo18b	UP
1233.923	-6.59073	1.94122	-3.39515	6.86E-04	0.015998	Pmf1	DOWN
1390.171	7.791776	2.295918	3.393751	6.89E-04	0.016054	Cryl1	UP
1231.396	-6.99275	2.060708	-3.39337	6.90E-04	0.016054	Ube2c	DOWN
2558.256	7.352181	2.167579	3.391887	6.94E-04	0.016118	Cachd1	UP
3914.045	-7.31519	2.157466	-3.39064	6.97E-04	0.016167	Sdhaf2	DOWN
2526.292	-7.24484	2.136979	-3.39023	6.98E-04	0.016168	Yipf1	DOWN
40.49297	-3.22597	0.951766	-3.38946	7.00E-04	0.016189	Ipp	DOWN
1363.034	7.574915	2.235594	3.388323	7.03E-04	0.016201	Nsg1	UP
56.08029	-2.70472	0.798293	-3.38812	7.04E-04	0.016201	Psmc6	DOWN
455.8977	-6.7268	1.985444	-3.38806	7.04E-04	0.016201	Rbmxl1	DOWN
2142.386	-7.43614	2.197082	-3.38455	7.13E-04	0.016243	Cdk9	DOWN
29.14089	-2.72879	0.80625	-3.38454	7.13E-04	0.016243	Gm10029	DOWN
19.60446	3.113443	0.919412	3.386341	7.08E-04	0.016243	Gm5069	UP
242.6991	5.765381	1.703022	3.385382	7.11E-04	0.016243	Myl12b	UP
854.4621	-7.14505	2.110603	-3.38531	7.11E-04	0.016243	Pdha1	DOWN
2311.562	7.366609	2.175621	3.38598	7.09E-04	0.016243	Sharpin	UP
338.3818	-5.41357	1.599087	-3.38541	7.11E-04	0.016243	Snx3	DOWN
324.8446	6.552805	1.937475	3.382136	7.19E-04	0.016273	Gm47208	UP
31.06462	-2.62556	0.77631	-3.38211	7.19E-04	0.016273	Msx1	DOWN
2942.252	6.463419	1.91078	3.382608	7.18E-04	0.016273	Pum3	UP
610.3271	-7.1062	2.101146	-3.38206	7.19E-04	0.016273	Tmem230	DOWN
137.5129	4.259376	1.258898	3.383416	7.16E-04	0.016273	Usf2	UP
83.83608	-3.5024	1.036097	-3.38038	7.24E-04	0.016349	Atp13a1	DOWN
761.709	-7.72559	2.28639	-3.37895	7.28E-04	0.016411	Lhfp	DOWN

1196.538	7.108823	2.104701	3.377593	7.31E-04	0.016435	Pcmtd1	UP
3370.649	7.062178	2.091034	3.377362	7.32E-04	0.016435	Psd3	UP
1483.576	-7.35712	2.178301	-3.37746	7.32E-04	0.016435	Tent2	DOWN
405.6204	-6.30111	1.865982	-3.37683	7.33E-04	0.016443	Ddc	DOWN
1748.971	-6.77965	2.009214	-3.37428	7.40E-04	0.01655	Topors	DOWN
2155.054	7.386833	2.189085	3.374393	7.40E-04	0.01655	Zcchc7	UP
105.3427	4.8178	1.428035	3.373726	7.42E-04	0.016559	Gm45885	UP
203.7095	-5.69046	1.688415	-3.3703	7.51E-04	0.01669	Actr5	DOWN
950.9445	-6.08837	1.806063	-3.37107	7.49E-04	0.01669	Klf13	DOWN
2782.862	-7.73703	2.295862	-3.36999	7.52E-04	0.01669	Mecr	DOWN
474.4131	6.97187	2.068521	3.370462	7.50E-04	0.01669	Star	UP
1458.392	7.128244	2.115461	3.369593	7.53E-04	0.016691	Adamtsl3	UP
2197.788	-7.42642	2.204486	-3.36878	7.55E-04	0.016717	Polr3b	DOWN
1447.442	7.985254	2.370839	3.368113	7.57E-04	0.016734	Casq2	UP
1956.81	7.231681	2.147642	3.367266	7.59E-04	0.016762	Tbcd	UP
2532.338	-7.3628	2.187647	-3.36562	7.64E-04	0.016823	Mrpl13	DOWN
3917.62	7.087461	2.105922	3.36549	7.64E-04	0.016823	Plaa	UP
307.3711	6.242703	1.855947	3.363621	7.69E-04	0.016913	Gtf3c6	UP
1680.168	7.534268	2.241023	3.361977	7.74E-04	0.01699	Exoc3	UP
197.4264	5.116325	1.522343	3.360823	7.77E-04	0.017016	Inf2	UP
2403.749	-6.98044	2.077021	-3.36079	7.77E-04	0.017016	Sccpdh	DOWN
2434.645	7.406976	2.204546	3.359865	7.80E-04	0.017034	Gtf3c5	UP
423.2613	-6.37364	1.897067	-3.35973	7.80E-04	0.017034	Nusap1	DOWN
981.9702	-7.00559	2.086582	-3.35745	7.87E-04	0.017151	Ywhah	DOWN
1444.271	7.207381	2.14741	3.356313	7.90E-04	0.017198	Pja2	UP
1303.433	7.338342	2.18692	3.35556	7.92E-04	0.017221	Snx24	UP
2397.686	6.469192	1.92818	3.355076	7.93E-04	0.017228	Sympk	UP
1399.283	5.920961	1.765412	3.353869	7.97E-04	0.017279	Socs6	UP
36.89421	2.766886	0.825448	3.351981	8.02E-04	0.017373	Rpsa-ps9	UP
1102.71	7.081993	2.113626	3.350636	8.06E-04	0.017425	Pard6b	UP
1972.147	7.13401	2.129647	3.349856	8.09E-04	0.017425	Psmd3	UP
268.499	-6.92781	2.067995	-3.35001	8.08E-04	0.017425	Rarres2	DOWN
350.1647	5.916379	1.766271	3.349644	8.09E-04	0.017425	Thsd1	UP
25.19458	-2.49525	0.745021	-3.34924	8.10E-04	0.017426	Dync1li1	DOWN
140.11	4.718397	1.409092	3.348537	8.12E-04	0.017447	AI837181	UP
4455.364	-6.68758	1.997898	-3.34731	8.16E-04	0.017486	Hectd4	DOWN
1507.24	7.224329	2.159022	3.346112	8.20E-04	0.017486	Map3k11	UP
2927.448	6.991456	2.089485	3.346019	8.20E-04	0.017486	Nphp4	UP
318.2173	6.899458	2.061878	3.3462	8.19E-04	0.017486	Tagln	UP
278.9267	5.982	1.787394	3.346772	8.18E-04	0.017486	Trp53rka	UP
1239.585	6.978671	2.086442	3.344771	8.24E-04	0.017541	Gabre	UP
1005.751	-7.10206	2.123849	-3.34396	8.26E-04	0.017558	Cept1	DOWN
21.56779	-3.47358	1.038839	-3.34371	8.27E-04	0.017558	Gm13680	DOWN
645.5994	6.913397	2.067787	3.343379	8.28E-04	0.017558	Gpr162	UP
1460.832	7.177138	2.147662	3.341837	8.32E-04	0.01757	Ano10	UP
2122.028	7.329301	2.19475	3.33947	8.39E-04	0.01757	Exoc7	UP
708.8416	-6.85804	2.052669	-3.34104	8.35E-04	0.01757	Fam107b	DOWN
2422.561	7.267971	2.176283	3.339625	8.39E-04	0.01757	Hmmr	UP
1830.631	-6.91315	2.069592	-3.34035	8.37E-04	0.01757	Larp4	DOWN
307.5949	-5.51114	1.650114	-3.33985	8.38E-04	0.01757	Mindy3	DOWN
1607.965	-7.2351	2.16515	-3.34162	8.33E-04	0.01757	Nfyc	DOWN
146.8096	-4.37316	1.308899	-3.3411	8.34E-04	0.01757	Sap30	DOWN
429.4739	6.760375	2.024227	3.339732	8.39E-04	0.01757	St3gal5	UP
605.0066	7.145318	2.137604	3.342676	8.30E-04	0.01757	Tmem11	UP

247.4752	-6.62441	1.985834	-3.33583	8.50E-04	0.017754	Gm26850	DOWN
2315.707	-5.98175	1.793101	-3.33598	8.50E-04	0.017754	Sall4	DOWN
1597.67	7.508671	2.251591	3.334829	8.54E-04	0.017795	C330018D20Rik	UP
96.00368	3.956778	1.186781	3.334043	8.56E-04	0.017822	1700008J07Rik	UP
628.4837	5.961436	1.788981	3.332308	8.61E-04	0.017909	Leo1	UP
1155.416	7.014007	2.105354	3.33151	8.64E-04	0.017937	Lmbr1	UP
35.45186	-2.99052	0.897894	-3.33059	8.67E-04	0.017973	Pradc1	DOWN
992.373	5.946865	1.78596	3.329786	8.69E-04	0.017986	Otub1	UP
29.85349	-2.94595	0.884763	-3.32965	8.70E-04	0.017986	Tmem256	DOWN
2449.695	7.261973	2.182027	3.328085	8.74E-04	0.018021	Cpeb3	UP
1768.587	7.171377	2.154851	3.328016	8.75E-04	0.018021	Elof1	UP
697.8558	-5.79904	1.742148	-3.32867	8.73E-04	0.018021	Scpep1	DOWN
3863.94	-7.15162	2.149533	-3.32706	8.78E-04	0.018059	Senp3	DOWN
112.5068	-3.97992	1.196622	-3.32596	8.81E-04	0.018083	Dars2	DOWN
97.87599	3.76476	1.131884	3.3261	8.81E-04	0.018083	Elk1	UP
383.3306	6.029415	1.81308	3.32551	8.83E-04	0.018089	Junb	UP
282.4039	-5.47045	1.645603	-3.32428	8.86E-04	0.018138	Cnot2	DOWN
2510.385	-7.30583	2.197901	-3.324	8.87E-04	0.018138	Impdh1	DOWN
1442.898	-7.22244	2.173651	-3.32272	8.91E-04	0.018138	Sae1	DOWN
685.6989	-7.18763	2.163092	-3.32285	8.91E-04	0.018138	Sdc2	DOWN
3528.458	7.084068	2.131872	3.322933	8.91E-04	0.018138	Usp16	UP
2504.472	7.06977	2.127794	3.322581	8.92E-04	0.018138	Usp32	UP
467.7402	6.351167	1.912428	3.320996	8.97E-04	0.018171	Cmss1	UP
872.7096	-7.23717	2.179196	-3.32103	8.97E-04	0.018171	Cnpy2	DOWN
912.5042	-6.73651	2.02818	-3.32146	8.95E-04	0.018171	Magoh	DOWN
1183.757	-6.97843	2.102703	-3.31879	9.04E-04	0.018223	Crbn	DOWN
4159.981	5.779675	1.741221	3.319323	9.02E-04	0.018223	Dnmt1	UP
13.59713	3.901971	1.175812	3.318534	9.05E-04	0.018223	Gm42717	UP
623.4303	-6.97746	2.102808	-3.31816	9.06E-04	0.018223	Gm42979	DOWN
41.5529	-2.62625	0.791508	-3.31803	9.07E-04	0.018223	Rpl22l1	DOWN
728.3615	7.277978	2.192648	3.319264	9.03E-04	0.018223	Tmem242	UP
1567.302	-7.10532	2.14247	-3.31642	9.12E-04	0.018306	Dusp11	DOWN
1459.334	6.798332	2.051428	3.31395	9.20E-04	0.018361	Ezh1	UP
266.5782	-5.87458	1.772762	-3.3138	9.20E-04	0.018361	Jam3	DOWN
380.4923	-5.99007	1.807324	-3.31433	9.19E-04	0.018361	Lars	DOWN
38.20121	-2.25168	0.67935	-3.31447	9.18E-04	0.018361	Orc1	DOWN
89.52356	-4.62817	1.396561	-3.31398	9.20E-04	0.018361	Rpl3-ps2	DOWN
1276.252	7.415111	2.238239	3.312922	9.23E-04	0.018395	3110009E18Rik	UP
131.9209	-4.75206	1.436169	-3.30884	0.000937	0.018642	Tuba1a	DOWN
2970.295	7.081566	2.141184	3.307313	9.42E-04	0.018696	Natd1	UP
2062.945	-7.32822	2.21555	-3.30763	9.41E-04	0.018696	Tchp	DOWN
36.95241	-3.15221	0.953256	-3.30678	9.44E-04	0.018708	Aasdhppt	DOWN
83.10984	-3.2953	0.997023	-3.30514	9.49E-04	0.018747	Pnrc1	DOWN
2853.139	-7.15115	2.16352	-3.30533	9.49E-04	0.018747	Tpm1	DOWN
307.1063	5.664996	1.713818	3.305482	9.48E-04	0.018747	Zswim5	UP
72.40291	3.02891	0.916938	3.303289	9.56E-04	0.018848	Pdp2	UP
697.2341	7.201137	2.180447	3.302596	9.58E-04	0.018871	Slc35d3	UP
1094.619	7.206832	2.182973	3.301384	9.62E-04	0.018881	Gm45555	UP
342.3564	6.367452	1.928659	3.301492	9.62E-04	0.018881	Nat9	UP
24.99956	2.652133	0.803309	3.30151	9.62E-04	0.018881	Zfp229	UP
1471.769	7.133218	2.161258	3.300494	9.65E-04	0.018918	Postn	UP
2464.465	-7.08609	2.149495	-3.29663	9.79E-04	0.019091	Fto	DOWN
15.49248	3.41414	1.035675	3.296535	9.79E-04	0.019091	Gm33677	UP
116.8407	-4.59922	1.394945	-3.29706	9.77E-04	0.019091	Rbbp9	DOWN

63.26989	3.196696	0.969512	3.297222	9.76E-04	0.019091	Slc6a20b	UP
4009.596	6.937979	2.105517	3.295143	9.84E-04	0.019109	Atp2b4	UP
1432.444	7.641133	2.319093	3.29488	9.85E-04	0.019109	Gm37183	UP
5389.131	6.915979	2.098485	3.295701	9.82E-04	0.019109	Larp4b	UP
2774.296	7.060901	2.142895	3.295029	9.84E-04	0.019109	Pik3r1	UP
1205.484	7.200947	2.187021	3.292582	9.93E-04	0.019237	4632427E13Rik	UP
559.2098	-6.42548	1.952273	-3.29128	9.97E-04	0.019237	Dcn	DOWN
805.0551	-7.40858	2.250453	-3.29204	9.95E-04	0.019237	Isca2	DOWN
507.468	6.344441	1.927586	3.291392	9.97E-04	0.019237	Laptm5	UP
901.6588	5.798003	1.761523	3.291472	9.97E-04	0.019237	Tnpo2	UP
1282.788	7.388688	2.247141	3.288039	0.001009	0.019388	Amhr2	UP
316.1655	6.409597	1.949157	3.288395	0.001008	0.019388	Gm10400	UP
1950.616	-7.92138	2.408919	-3.28835	0.001008	0.019388	Serpinb9	DOWN
1983.942	7.265873	2.211174	3.285979	0.001016	0.019483	Aprt	UP
110.6776	-4.27098	1.299752	-3.28599	0.001016	0.019483	Dnajb9	DOWN
1923.782	7.312802	2.22667	3.284187	0.001023	0.019569	Cdkn1a	UP
52.99213	-2.35599	0.717404	-3.28405	0.001023	0.019569	Rbm3	DOWN
457.4747	6.946801	2.115688	3.283472	0.001025	0.019585	Lsm5	UP
99.97337	-3.33538	1.016199	-3.28221	0.00103	0.019637	Gm6916	DOWN
694.352	-6.80254	2.072654	-3.28204	0.001031	0.019637	Peg12	DOWN
91.56453	4.263902	1.299307	3.281673	0.001032	0.019639	Etfbkmt	UP
638.3661	-6.27128	1.911458	-3.28089	0.001035	0.01967	Prmt3	DOWN
2421.93	7.331092	2.234726	3.280533	0.001036	0.019671	Capzb	UP
1120.212	7.762484	2.366529	3.280114	0.001038	0.019677	Dcaf8l	UP
981.6386	-7.08976	2.161675	-3.27975	0.001039	0.019678	Tspan31	DOWN
3020.805	7.234702	2.20622	3.279229	0.001041	0.019691	Rab2a	UP
1657.314	6.837175	2.086345	3.277106	0.001049	0.019816	Rtel1	UP
488.1295	-7.12545	2.174581	-3.2767	0.00105	0.01982	Nutf2	DOWN
5405.066	6.714208	2.050434	3.27453	0.001058	0.019854	Eef2	UP
339.3202	-5.91737	1.806705	-3.27523	0.001056	0.019854	Ier3ip1	DOWN
923.0239	6.910387	2.11013	3.274863	0.001057	0.019854	Ormdl3	UP
971.498	-6.32598	1.931368	-3.27539	0.001055	0.019854	Ranbp9	DOWN
1013.543	-6.7438	2.059355	-3.27471	0.001058	0.019854	Tram1	DOWN
776.986	5.869522	1.793356	3.272927	0.001064	0.019943	Btg1	UP
883.5223	7.419875	2.268532	3.270783	0.001073	0.020027	Asnsd1	UP
303.451	-6.08032	1.859005	-3.27074	0.001073	0.020027	Smim11	DOWN
180.5105	5.264018	1.609241	3.271119	0.001071	0.020027	Trap1a	UP
2172.485	-7.31738	2.237795	-3.26991	0.001076	0.020062	Plrg1	DOWN
1752.293	7.275044	2.226059	3.268127	0.001083	0.020164	Mex3c	UP
1490.051	-7.05105	2.157989	-3.26742	0.001085	0.020191	Idh3a	DOWN
1084.851	7.222789	2.211523	3.26598	0.001091	0.020244	4930563M21Rik	UP
36.29149	-2.87074	0.879439	-3.26429	0.001097	0.020244	C430049B03Rik	DOWN
785.832	6.893532	2.11097	3.265576	0.001092	0.020244	Cacng4	UP
873.1049	5.690871	1.743521	3.26401	0.001098	0.020244	Ddx19a	UP
428.9719	6.235905	1.909811	3.265194	0.001094	0.020244	Gatm	UP
110.0196	-4.0586	1.243225	-3.26458	0.001096	0.020244	Hnrnpf	DOWN
897.2429	7.15249	2.190248	3.265608	0.001092	0.020244	Qrsl1	UP
625.5088	-6.69392	2.050413	-3.26467	0.001096	0.020244	Tex12	DOWN
48.43803	-2.6626	0.815911	-3.26335	0.001101	0.020251	Ppib	DOWN
17634	-3.90994	1.198175	-3.26324	0.001101	0.020251	Prdx1	DOWN
344.5228	-5.76962	1.768674	-3.26211	0.001106	0.020285	Bcap31	DOWN
621.4808	-6.59402	2.021295	-3.26228	0.001105	0.020285	Saysd1	DOWN
700.6923	-6.47018	1.983836	-3.26145	0.001108	0.020301	Gpr160	DOWN
745.555	6.737352	2.065899	3.261221	0.001109	0.020301	Itpk1	UP

2034.426	-6.30709	1.934673	-3.26003	0.001114	0.020316	Brix1	DOWN
1344.862	-6.8038	2.086913	-3.26022	0.001113	0.020316	Psmd8	DOWN
97.98459	-3.68543	1.130376	-3.26036	0.001113	0.020316	Ssh3	DOWN
311.8225	-6.07924	1.865271	-3.25917	0.001117	0.020354	C230071H17Rik	DOWN
254.232	-5.5907	1.715633	-3.25868	0.001119	0.020365	BC065397	DOWN
201.4627	-5.52007	1.694903	-3.25687	0.001126	0.020435	Efcab7	DOWN
14.22118	3.493236	1.072443	3.25727	0.001125	0.020435	Rpl5-ps1	UP
3499.291	6.656617	2.043961	3.256725	0.001127	0.020435	Zmym5	UP
1587.034	7.018014	2.155217	3.256291	0.001129	0.020443	Per1	UP
1029.695	-6.90697	2.121786	-3.25526	0.001133	0.02047	Pabpc4l	DOWN
39.9676	2.747249	0.843889	3.25546	0.001132	0.02047	Tcf21	UP
2638.904	5.726534	1.759691	3.254284	0.001137	0.020517	Scaf11	UP
1378.949	-6.8415	2.102855	-3.25343	0.00114	0.020555	Fbxo38	DOWN
581.4874	6.758191	2.078209	3.25193	0.001146	0.020568	2310034G01Rik	UP
230.2556	5.146392	1.582872	3.2513	0.001149	0.020568	A730063M14Rik	UP
70.24443	3.319677	1.020679	3.252421	0.001144	0.020568	AC106834.1	UP
81.67695	3.668131	1.128317	3.250974	0.00115	0.020568	Klf6	UP
2423.37	6.825073	2.099209	3.25126	0.001149	0.020568	Ncapg2	UP
164.8315	-4.74155	1.457815	-3.2525	0.001144	0.020568	Ppat	DOWN
829.1118	6.61022	2.033192	3.251154	0.001149	0.020568	Wars2	UP
73.86465	-3.82843	1.177944	-3.25009	0.001154	0.020609	Mrpl44	DOWN
418.6433	-6.43624	1.982512	-3.24651	0.001168	0.020722	Chmp1b	DOWN
264.7338	5.840848	1.798463	3.247689	0.001163	0.020722	Gm42991	UP
761.6075	-6.64728	2.047568	-3.24643	0.001169	0.020722	Hint3	DOWN
1398.02	6.940179	2.137131	3.247429	0.001165	0.020722	Rangap1	UP
469.359	-6.33193	1.950506	-3.2463	0.001169	0.020722	Rnf7	DOWN
147.4846	-4.14164	1.275812	-3.24628	0.001169	0.020722	Thumpd3	DOWN
718.1402	-6.6238	2.039245	-3.24816	0.001162	0.020722	Ypel3	DOWN
23.06446	3.375492	1.040012	3.245629	0.001172	0.020746	Gm5100	UP
1273.87	-6.47318	1.995239	-3.24431	0.001177	0.020819	Klhl24	DOWN
1202.757	5.984709	1.845255	3.243297	0.001182	0.020851	Dnajc10	UP
631.893	-6.68313	2.061644	-3.24165	0.001188	0.020851	Elp5	DOWN
170.3332	-4.28331	1.321344	-3.24163	0.001188	0.020851	Gatad1	DOWN
296.665	-5.90305	1.820627	-3.24232	0.001186	0.020851	Gm9958	DOWN
816.2597	6.597401	2.035078	3.241842	0.001188	0.020851	Ifnar1	UP
2620.189	7.027436	2.167639	3.241978	0.001187	0.020851	Trir	UP
553.7139	7.066698	2.179098	3.242947	0.001183	0.020851	Uqcc3	UP
94321.18	6.383528	1.969723	3.240825	0.001192	0.020887	CT010467.1	UP
1219.279	-6.71774	2.073781	-3.23937	0.001198	0.020971	Fh1	DOWN
605.7253	-6.30757	1.947516	-3.23877	0.0012	0.020991	Erg28	DOWN
592.4647	6.830032	2.109365	3.237956	0.001204	0.021028	C1qtnf7	UP
1509.808	7.256207	2.241966	3.236538	0.00121	0.021052	Alg3	UP
457.5241	-5.31605	1.642597	-3.23637	0.001211	0.021052	Eif4a2	DOWN
359.8903	5.893862	1.820954	3.236688	0.001209	0.021052	Gm5112	UP
18.22427	2.818255	0.870689	3.23681	0.001209	0.021052	Olfr1349	UP
802.018	-6.39784	1.977268	-3.2357	0.001213	0.021078	Tfg	DOWN
312.9442	5.555038	1.717097	3.235134	0.001216	0.021096	Pdlim2	UP
2026.989	6.820829	2.108925	3.234268	0.00122	0.021137	Mapk10	UP
743.6997	-7.92088	2.449705	-3.2334	0.001223	0.021178	Snhg6	DOWN
1150.517	-6.91558	2.139314	-3.23262	0.001227	0.021213	Cnn2	DOWN
2299.623	6.82921	2.113373	3.231427	0.001232	0.021278	Kctd3	UP
297.5733	-5.32579	1.648779	-3.23014	0.001237	0.02135	Rps6ka6	DOWN
191.7682	-4.7911	1.48365	-3.22927	0.001241	0.021392	Serinc1	DOWN
1178.515	6.88605	2.136052	3.223728	0.001265	0.021613	Ccdc137	UP

35.47104	-2.9769	0.923806	-3.22242	0.001271	0.021613	Coro1b	DOWN
1696.418	6.900211	2.14128	3.22247	0.001271	0.021613	Csnk1d	UP
1703.799	6.820768	2.117006	3.221894	0.001273	0.021613	Csnk1g2	UP
1424.472	-6.53586	2.027404	-3.22376	0.001265	0.021613	Dab2	DOWN
49.43966	-2.42966	0.7533	-3.22536	0.001258	0.021613	Gm1965	DOWN
24.4446	2.443161	0.757703	3.224429	0.001262	0.021613	Gm53	UP
1444.173	7.016245	2.177824	3.221677	0.001274	0.021613	Gtf2h4	UP
1746.299	-6.90607	2.143638	-3.22166	0.001275	0.021613	Map1lc3b	DOWN
347.7235	-6.1356	1.902095	-3.22571	0.001257	0.021613	Meiob	DOWN
25.91463	-2.44286	0.757926	-3.22308	0.001268	0.021613	Pi16	DOWN
1420.69	7.045056	2.184179	3.225493	0.001258	0.021613	Sqstm1	UP
1217.927	-7.07196	2.193242	-3.22443	0.001262	0.021613	Tmem123	DOWN
3241.792	6.802382	2.109748	3.224263	0.001263	0.021613	Trim24	UP
1771.338	6.831244	2.12034	3.221769	0.001274	0.021613	Zp3	UP
2747.19	6.609047	2.051684	3.221279	0.001276	0.021618	Map1a	UP
2053.905	7.049847	2.189932	3.219208	0.001285	0.021752	Ccdc34	UP
59.38248	-7.59742	2.36057	-3.21847	0.001289	0.021784	6330403K07Rik	DOWN
1483.212	6.89344	2.142633	3.217275	0.001294	0.021852	Ivd	UP
2007.649	6.367089	1.980076	3.215577	0.001302	0.021958	Pcgf6	UP
2284.818	6.536333	2.033103	3.214955	0.001305	0.021982	Cdc34	UP
3283.888	-7.00311	2.178561	-3.21456	0.001306	0.021989	Zfp423	DOWN
1013.561	-7.06265	2.197976	-3.21325	0.001312	0.022012	Ccdc163	DOWN
1573.129	-6.82712	2.125162	-3.21252	0.001316	0.022012	Cog7	DOWN
1123.416	7.135621	2.221264	3.212414	0.001316	0.022012	Hacd4	UP
1214.757	-6.8019	2.117089	-3.21286	0.001314	0.022012	Lcmt1	DOWN
265.3641	5.716953	1.779224	3.213171	0.001313	0.022012	Mfap1b	UP
1220.652	-6.95734	2.164868	-3.21375	0.00131	0.022012	Srbd1	DOWN
1478.193	6.892809	2.146209	3.211621	0.00132	0.02205	4933427D06Rik	UP
3297.342	6.781455	2.111802	3.211218	0.001322	0.022057	Wdr36	UP
56.73962	-2.75064	0.856838	-3.21022	0.001326	0.02211	Txlng	DOWN
1981.184	-7.1308	2.221863	-3.20938	0.00133	0.022128	Zbtb45	DOWN
1796.017	-6.95709	2.167635	-3.20953	0.00133	0.022128	Zfp655	DOWN
174.0161	4.693235	1.46255	3.208941	0.001332	0.022138	Ube2g2	UP
405.8533	-5.92571	1.846885	-3.20849	0.001334	0.02215	Ift46	DOWN
1575.393	5.619747	1.751941	3.207727	0.001338	0.022185	Ablim1	UP
236.0677	5.262002	1.640718	3.207134	0.001341	0.022208	Dapk3	UP
1450.066	7.109158	2.218533	3.204441	0.001353	0.022374	Arhgap11a	UP
609.3233	6.867643	2.143207	3.204377	0.001354	0.022374	Gnb5	UP
476.135	-6.18892	1.933021	-3.20168	0.001366	0.022443	Cry1	DOWN
975.25	-6.6264	2.069352	-3.20216	0.001364	0.022443	Ddx51	DOWN
1213.426	-6.75566	2.109498	-3.2025	0.001362	0.022443	Eno1	DOWN
21.94385	-3.05767	0.95481	-3.20239	0.001363	0.022443	Nxt1	DOWN
1213.601	6.898288	2.153765	3.202897	0.001361	0.022443	Pam16	UP
1337.592	7.093629	2.215545	3.201754	0.001366	0.022443	Rhog	UP
561.6746	-6.63698	2.075184	-3.19826	0.001383	0.022687	Peg10	DOWN
1273.942	6.714836	2.100127	3.197347	0.001387	0.022735	Ell	UP
218.8042	5.483444	1.715173	3.19702	0.001389	0.022738	AA465934	UP
1429.138	-7.00552	2.191936	-3.19604	0.001393	0.022773	Cip2a	DOWN
332.2776	-5.5439	1.734657	-3.19597	0.001394	0.022773	Tmem106b	DOWN
214.0719	5.000129	1.564949	3.195074	0.001398	0.022813	Vps26c	UP
2000.2	7.32187	2.291763	3.194864	0.001399	0.022813	Zmat1	UP
1791.865	7.436709	2.328561	3.193693	0.001405	0.022864	Fam98a	UP
1648.934	9.231816	2.890881	3.193426	0.001406	0.022864	Gm37598	UP
1309.586	7.239785	2.267162	3.193325	0.001406	0.022864	Umps	UP

133.2744	-4.58936	1.437944	-3.19161	0.001415	0.022976	Colec12	DOWN
147.5843	4.61398	1.44598	3.190903	0.001418	0.023009	Vkorc1	UP
368.2691	5.93827	1.861266	3.190446	0.001421	0.023021	Gpr137	UP
9901.79	-4.46674	1.400183	-3.19011	0.001422	0.023024	Sarnp	DOWN
1898.321	-6.84035	2.144517	-3.18969	0.001424	0.023034	Rbm10	DOWN
209.0411	4.880555	1.530867	3.188098	0.001432	0.023095	Hacd3	UP
17.07337	-3.12734	0.98096	-3.18804	0.001432	0.023095	Numb	DOWN
1350.311	6.757883	2.119739	3.188073	0.001432	0.023095	Tdrd5	UP
1659.362	6.883929	2.159716	3.187423	0.001435	0.02312	9130401M01Rik	UP
1584.664	5.697686	1.787726	3.187113	0.001437	0.023122	Hdgfl2	UP
1470.632	6.889363	2.164665	3.182646	0.001459	0.023457	Arid4a	UP
1415.778	7.18016	2.257646	3.180374	0.001471	0.023618	Wdfy1	UP
2030.319	6.977031	2.194009	3.180037	0.001473	0.023621	Ckap4	UP
333.2041	6.02398	1.895145	3.178638	0.00148	0.023711	Polr2l	UP
851.2715	-6.59626	2.077002	-3.17586	0.001494	0.023876	Fbxl3	DOWN
165.8244	-4.7185	1.485899	-3.17552	0.001496	0.023876	Golga5	DOWN
2060.446	-6.71997	2.116229	-3.17545	0.001496	0.023876	Marchf8	DOWN
1522.738	-6.79891	2.140674	-3.17606	0.001493	0.023876	Prim1	DOWN
3775.494	6.751466	2.126396	3.175075	0.001498	0.023878	Bsg	UP
1276.477	7.172873	2.259287	3.17484	0.001499	0.023878	Oard1	UP
1063.282	-5.63552	1.775706	-3.17368	0.001505	0.023949	Ncoa3	DOWN
2013.37	7.02168	2.212816	3.173187	0.001508	0.023966	Apbb1	UP
65.95468	3.082094	0.971541	3.172375	0.001512	0.024008	Crabp2	UP
236.1257	-5.11145	1.61161	-3.17164	0.001516	0.02402	Calu	DOWN
1007.396	5.511248	1.737634	3.171697	0.001516	0.02402	Crls1	UP
577.4671	-7.00257	2.209272	-3.16963	0.001526	0.024033	AU020206	DOWN
1146.495	6.898472	2.175729	3.170649	0.001521	0.024033	Crk	UP
918.748	-6.66103	2.10157	-3.16955	0.001527	0.024033	Ddost	DOWN
213.5744	-5.07845	1.602315	-3.16944	0.001527	0.024033	Pwp1	DOWN
2490.444	7.088566	2.235819	3.170456	0.001522	0.024033	Slc27a4	UP
1739.076	7.342562	2.3165	3.169679	0.001526	0.024033	Zfp105	UP
775.6213	6.629168	2.09077	3.170682	0.001521	0.024033	Zfp91	UP
102.4716	-3.70717	1.169787	-3.1691	0.001529	0.024038	Rpl13a	DOWN
753.8649	-6.70661	2.116669	-3.16847	0.001532	0.024042	Asb8	DOWN
631.5614	-6.61012	2.086163	-3.16855	0.001532	0.024042	Ntf3	DOWN
154.9409	-4.68093	1.478053	-3.16695	0.00154	0.024139	Pla2g7	DOWN
2500.27	7.268195	2.295186	3.166713	0.001542	0.024139	Srp68	UP
1465.829	-6.91036	2.18283	-3.16578	0.001547	0.024193	Lsm6	DOWN
2747.417	-6.66912	2.107579	-3.16435	0.001554	0.02424	Cdk16	DOWN
2184.857	6.810698	2.151964	3.164875	0.001551	0.02424	Fign	UP
1531.524	6.861629	2.168398	3.164377	0.001554	0.02424	Sap30l	UP
580.6529	6.582504	2.080445	3.163988	0.001556	0.024246	7420426K07Rik	UP
1142.814	6.693225	2.116129	3.162957	0.001562	0.024308	Spout1	UP
37.52302	-2.95324	0.933833	-3.16249	0.001564	0.024322	Nap1l2	DOWN
980.2467	-6.46906	2.045749	-3.1622	0.001566	0.024323	Btbd10	DOWN
94.31185	3.433253	1.086077	3.16115	0.001571	0.024338	Icam1	UP
197.3333	4.352857	1.376834	3.161496	0.00157	0.024338	Tsga10	UP
746.9048	6.673733	2.111036	3.161354	0.00157	0.024338	Znhit1	UP
1984.819	5.93522	1.877935	3.160504	0.001575	0.024369	Lmo7	UP
2233.316	-6.65418	2.106475	-3.15892	0.001584	0.024471	Mff	DOWN
925.197	6.610949	2.09293	3.158706	0.001585	0.024471	Nop2	UP
2135.402	7.105685	2.250451	3.157449	0.001592	0.024553	Zfp395	UP
1116.025	-6.5404	2.072016	-3.15654	0.001597	0.024604	Spg21	DOWN
282.6805	-5.41041	1.71418	-3.15627	0.001598	0.024604	Tpi1	DOWN

1340.944	-6.81244	2.159842	-3.15414	0.00161	0.024712	Ercc8	DOWN
1693.41	-6.87946	2.180844	-3.1545	0.001608	0.024712	Tmeff2	DOWN
258.1054	5.184886	1.643749	3.154305	0.001609	0.024712	Zfp626	UP
40.00591	-2.58233	0.81886	-3.15357	0.001613	0.024736	Nudt4	DOWN
286.706	-5.42317	1.720471	-3.15214	0.001621	0.024833	Flrt2	DOWN
112.0453	4.133365	1.311496	3.151643	0.001624	0.024851	Pop7	UP
73.94836	3.740402	1.187625	3.14948	0.001636	0.024993	Gm47493	UP
814.8777	6.845722	2.17365	3.149412	0.001636	0.024993	Tktl1	UP
18.70332	-2.78648	0.884858	-3.14907	0.001638	0.024998	Rab5a	DOWN
291.1888	6.66201	2.116355	3.14787	0.001645	0.025052	1700007L15Rik	UP
1250	-5.68677	1.806813	-3.14741	0.001647	0.025052	Angel2	DOWN
932.4824	-6.57464	2.088973	-3.14731	0.001648	0.025052	Banf1	DOWN
1077.636	-6.77857	2.153608	-3.14754	0.001646	0.025052	Ptar1	DOWN
94.84109	-3.75392	1.193597	-3.14505	0.001661	0.025214	Cpxm1	DOWN
131.572	-4.66974	1.48488	-3.14486	0.001662	0.025214	Tex261	DOWN
469.3898	5.977671	1.901124	3.144282	0.001665	0.025239	Irgm1	UP
939.0085	6.845668	2.177458	3.14388	0.001667	0.025247	Leng1	UP
1012.737	7.028652	2.235842	3.143626	0.001669	0.025247	Tpk1	UP
112.5882	-4.52972	1.441578	-3.1422	0.001677	0.025261	Agtr2	DOWN
1971.482	6.876825	2.190719	3.139071	0.001695	0.025261	Anapc2	UP
2401.857	6.577204	2.094723	3.139892	0.00169	0.025261	Atad2b	UP
652.1798	-6.12734	1.951821	-3.13929	0.001694	0.025261	Cand1	DOWN
2077.115	6.71444	2.136395	3.142884	0.001673	0.025261	Cdip1	UP
1091.325	6.604779	2.104021	3.139122	0.001695	0.025261	Enpp2	UP
2019.703	-6.75876	2.152671	-3.13971	0.001691	0.025261	Farsa	DOWN
176.6196	4.540359	1.445784	3.140414	0.001687	0.025261	Gm9493	UP
892.3509	6.476106	2.060957	3.142282	0.001676	0.025261	Hgs	UP
1698.927	5.799216	1.846472	3.1407	0.001685	0.025261	Myo9a	UP
2410.028	6.740342	2.147181	3.139159	0.001694	0.025261	Prmt7	UP
391.3141	-6.19165	1.970857	-3.1416	0.00168	0.025261	Psmd10	DOWN
238.6417	5.203107	1.656869	3.140324	0.001688	0.025261	Rab5b	UP
1137.439	-6.14767	1.956415	-3.14231	0.001676	0.025261	Rbm38	DOWN
93.06562	-4.2091	1.340193	-3.14067	0.001686	0.025261	Rnf26	DOWN
1476.121	6.851021	2.182553	3.138995	0.001695	0.025261	Serhl	UP
1216.69	6.616787	2.108201	3.138595	0.001698	0.025271	Cd47	UP
1415.938	-7.22196	2.302681	-3.13633	0.001711	0.025443	Cmtm3	DOWN
2440.512	6.664055	2.125481	3.135316	0.001717	0.025459	Nhlrc2	UP
1854.079	6.94549	2.215011	3.135646	0.001715	0.025459	Rad23b	UP
774.4757	5.797124	1.84894	3.135378	0.001716	0.025459	Rnf170	UP
7963.68	-6.40552	2.044712	-3.13272	0.001732	0.02564	Eef1d	DOWN
853.9983	-5.62293	1.794969	-3.13261	0.001733	0.02564	Ssr1	DOWN
1549.442	-6.29692	2.010248	-3.13241	0.001734	0.02564	Zfp207	DOWN
24.91927	-2.5286	0.807617	-3.13094	0.001742	0.025696	Atp6v0d1	DOWN
26.76945	-2.4039	0.76776	-3.13105	0.001742	0.025696	Cep85	DOWN
47.97571	-2.22724	0.711276	-3.13133	0.00174	0.025696	Cxxc5	DOWN
1966.746	6.704129	2.141878	3.130025	0.001748	0.025704	Chpt1	UP
1788.306	-6.69043	2.137184	-3.13049	0.001745	0.025704	Cops8	DOWN
40.01872	-2.83479	0.905612	-3.13024	0.001747	0.025704	Zfand2b	DOWN
215.4818	-4.34802	1.389572	-3.12904	0.001754	0.025766	Phospho2	DOWN
950.0188	6.857415	2.191797	3.128672	0.001756	0.025774	Asrgl1	UP
299.7077	5.508798	1.761009	3.128206	0.001759	0.025791	Cdk5rap3	UP
17.29913	-3.14152	1.004401	-3.12775	0.001761	0.025807	Yars2	DOWN
1479.249	6.539513	2.091281	3.127037	0.001766	0.025829	1700025G04Rik	UP
128.4567	-4.14357	1.325116	-3.12695	0.001766	0.025829	Tbx3os1	DOWN

82.06398	3.228666	1.032703	3.126422	0.001769	0.025831	Slu7	UP
1778.631	6.505038	2.080692	3.126381	0.00177	0.025831	Tulp4	UP
2179.863	6.646371	2.126516	3.125475	0.001775	0.025887	Mta1	UP
1975.189	6.868031	2.197651	3.125169	0.001777	0.02589	Slc25a12	UP
354.6953	-5.18134	1.658409	-3.12428	0.001782	0.025896	Nt5dc2	DOWN
662.3936	6.404578	2.049597	3.124799	0.001779	0.025896	Pigs	UP
243.417	-5.01395	1.60473	-3.12448	0.001781	0.025896	Syap1	DOWN
1533.908	6.719188	2.151141	3.123546	0.001787	0.025937	Nsrp1	UP
706.4015	-6.52125	2.08856	-3.12237	0.001794	0.026017	Tmem237	DOWN
1344.578	-6.63292	2.124848	-3.1216	0.001799	0.026037	Cope	DOWN
1475.141	6.71378	2.150708	3.12166	0.001798	0.026037	Prr51	UP
2028.926	-6.63551	2.126134	-3.12093	0.001803	0.026048	Appbp2	DOWN
3486.559	6.578634	2.1078	3.121091	0.001802	0.026048	Safb2	UP
1527.995	-6.79284	2.177259	-3.11991	0.001809	0.026115	Mettl26	DOWN
1765.484	-7.0245	2.253122	-3.11767	0.001823	0.026265	Hspa13	DOWN
17.65029	2.845954	0.912786	3.117878	0.001822	0.026265	Slc2a4	UP
1179.054	-6.35421	2.038855	-3.11656	0.00183	0.026302	Dop1a	DOWN
1202.476	-6.56691	2.107175	-3.11645	0.00183	0.026302	Mfsd11	DOWN
1013.593	-6.65946	2.136682	-3.11673	0.001829	0.026302	Shld2	DOWN
1749.648	6.920592	2.22142	3.115391	0.001837	0.026319	Nfxl1	UP
622.3675	-6.27512	2.014368	-3.11518	0.001838	0.026319	Pdhb	DOWN
2047.836	6.696512	2.149319	3.115643	0.001835	0.026319	Phtf2	UP
317.0737	-5.41183	1.737036	-3.11555	0.001836	0.026319	Tmem41a	DOWN
126.5766	4.51887	1.45076	3.114829	0.001841	0.02632	Sntb1	UP
30.35919	2.268974	0.728487	3.114639	0.001842	0.02632	Uroc1	UP
898.7272	6.482314	2.081901	3.113651	0.001848	0.026384	Ccdc127	UP
1350.74	6.65386	2.13722	3.113326	0.00185	0.026389	Chaf1b	UP
140.1736	4.452166	1.430495	3.112326	0.001856	0.026428	Nfatc2ip	UP
680.3615	-6.08346	1.954786	-3.11209	0.001858	0.026428	Rac1	DOWN
1035.377	6.134817	1.9711	3.112382	0.001856	0.026428	St3gal2	UP
248.5723	-5.6205	1.806653	-3.111	0.001865	0.026501	Acot2	DOWN
26.69653	-2.26858	0.729787	-3.10855	0.00188	0.026698	D030056L22Rik	DOWN
3156.734	6.729329	2.165247	3.107881	0.001884	0.026734	Rad54l	UP
1870.287	6.616993	2.129376	3.107481	0.001887	0.026747	Gnl2	UP
222.3806	5.137279	1.653658	3.106614	0.001892	0.026801	Gm17767	UP
543.0018	-5.57107	1.793765	-3.1058	0.001898	0.026851	Psma5	DOWN
939.6179	-6.57913	2.118662	-3.10532	0.001901	0.026859	Ndel1	DOWN
26.3426	-2.47509	0.797085	-3.10517	0.001902	0.026859	Tcea2	DOWN
2457.176	6.549365	2.109749	3.104334	0.001907	0.026911	Nrbp1	UP
598.3162	-6.63424	2.137556	-3.10366	0.001911	0.026949	B2m	DOWN
1471.882	6.493268	2.092618	3.10294	0.001916	0.02699	Dis3l2	UP
26.04755	-2.45569	0.791641	-3.10203	0.001922	0.027049	Thap11	DOWN
82.08168	-3.56893	1.151431	-3.09956	0.001938	0.027251	Nectin2	DOWN
1083.95	5.581225	1.800886	3.099156	0.001941	0.02726	Ankhd1	UP
1449.09	-6.66002	2.149134	-3.09893	0.001942	0.02726	Trim26	DOWN
237.3263	-4.85652	1.567509	-3.09824	0.001947	0.0273	Dtymk	DOWN
1072.142	6.711495	2.166987	3.097155	0.001954	0.027375	Ppil3	UP
20.91959	-2.913	0.940806	-3.09628	0.00196	0.027432	Gm10224	DOWN
127.4078	3.82681	1.236144	3.095765	0.001963	0.027455	Capn10	UP
1723.777	6.636146	2.145813	3.092603	0.001984	0.027711	Efnb1	UP
3020.772	6.585133	2.129399	3.092484	0.001985	0.027711	Sf3b2	UP
2929.211	6.473169	2.093509	3.092019	0.001988	0.02773	Mga	UP
876.5711	-6.67375	2.158821	-3.09139	0.001992	0.027762	Ccnc	DOWN
209.9503	-5.16518	1.670957	-3.09115	0.001994	0.027762	Gnpda1	DOWN

629.8238	-6.13251	1.98451	-3.09019	0.002	0.027827	4930523C07Rik	DOWN
1268.933	7.00439	2.267215	3.089425	0.002005	0.027874	Il1rl1	UP
2176.715	6.713931	2.174191	3.088013	0.002015	0.027982	Map2k4	UP
179.9638	-4.667	1.51218	-3.08627	0.002027	0.028122	Zfp748	DOWN
417.1319	4.89061	1.585726	3.084145	0.002041	0.028137	2010001K21Rik	UP
18.86213	2.641923	0.856717	3.083775	0.002044	0.028137	9630002D21Rik	UP
132.6718	-3.58056	1.160564	-3.08519	0.002034	0.028137	Atp5mpl	DOWN
40.64329	2.171009	0.703695	3.085156	0.002034	0.028137	AW011738	UP
149.1633	4.793636	1.55474	3.083239	0.002048	0.028137	Clec14a	UP
117.6611	3.747982	1.214965	3.084849	0.002037	0.028137	Phldb3	UP
2284.687	6.67313	2.163881	3.083871	0.002043	0.028137	Selenoi	UP
1062.196	6.594075	2.137036	3.085618	0.002031	0.028137	Snf8	UP
2304.452	6.622213	2.147625	3.083505	0.002046	0.028137	Sycp2	UP
244.1978	-5.02377	1.629367	-3.08326	0.002047	0.028137	Ubxn8	DOWN
125.9486	-4.07621	1.321982	-3.0834	0.002046	0.028137	Zfp78	DOWN
3767.95	6.436847	2.088694	3.081757	0.002058	0.028252	Arl4a	UP
1278.321	-6.27956	2.038148	-3.08101	0.002063	0.028274	Rmc1	DOWN
404.0658	5.462404	1.772868	3.081111	0.002062	0.028274	Zp2	UP
13.72383	-3.51921	1.142353	-3.08067	0.002065	0.028281	Gm4742	DOWN
95.00597	-3.08593	1.002026	-3.07969	0.002072	0.028301	Ankle2	DOWN
1506.737	-6.92168	2.247402	-3.07986	0.002071	0.028301	Bloc1s5	DOWN
1492.242	6.927643	2.249155	3.080109	0.002069	0.028301	Pias2	UP
345.9889	-5.70719	1.854793	-3.07699	0.002091	0.028484	Cenpq	DOWN
1805.565	6.596554	2.143662	3.077236	0.002089	0.028484	Elac2	UP
44.48	-2.46537	0.801114	-3.07743	0.002088	0.028484	Rab5if	DOWN
999.1241	6.433733	2.092221	3.075073	0.002105	0.028569	Abcd3	UP
259.4251	-5.04093	1.639032	-3.07556	0.002101	0.028569	Casp6	DOWN
1012.653	-6.41837	2.087162	-3.07516	0.002104	0.028569	Cd9	DOWN
2415.474	6.849655	2.227255	3.07538	0.002102	0.028569	Stimate	UP
292.727	5.7096	1.857286	3.074163	0.002111	0.028632	Micos13	UP
1290.909	6.697588	2.179456	3.073055	0.002119	0.028713	Stx7	UP
501.8919	-6.44527	2.097828	-3.07235	0.002124	0.028756	Mospd2	DOWN
26.15527	-2.28556	0.744037	-3.07184	0.002127	0.028756	Hcfc2	DOWN
752.9486	-5.1948	1.690974	-3.07207	0.002126	0.028756	Ndufab1	DOWN
16.84583	-2.80337	0.91276	-3.07132	0.002131	0.028764	Myc	DOWN
1141.299	-6.50724	2.118763	-3.07124	0.002132	0.028764	Ywhag	DOWN
850.6928	-5.8051	1.890595	-3.07051	0.002137	0.028788	Ndufa6	DOWN
298.6913	-5.00155	1.628913	-3.07049	0.002137	0.028788	Vps36	DOWN
189.2157	-5.07251	1.65245	-3.06969	0.002143	0.028815	Bbof1	DOWN
5755.263	-6.86123	2.23504	-3.06985	0.002142	0.028815	Psmd6	DOWN
2164.095	-5.7041	1.858812	-3.06868	0.00215	0.028881	H3f3a	DOWN
692.2514	-5.99361	1.953334	-3.0684	0.002152	0.028881	Svbp	DOWN
1443.782	-5.99826	1.95495	-3.06824	0.002153	0.028881	Uba1	DOWN
354.0667	-5.3695	1.751015	-3.0665	0.002166	0.028981	Fam126a	DOWN
2062.201	6.748369	2.200511	3.066728	0.002164	0.028981	Fbxo33	UP
1633.754	6.489074	2.116153	3.066448	0.002166	0.028981	Numa1	UP
2211.849	6.475083	2.111812	3.066127	0.002169	0.028987	Usp2	UP
1235.789	6.620326	2.159677	3.065425	0.002174	0.028996	Nifk	UP
1063.077	6.885318	2.246233	3.065273	0.002175	0.028996	Rspo1	UP
57.9122	2.173962	0.709113	3.06575	0.002171	0.028996	Slc43a2	UP
28.91526	2.441845	0.797541	3.061716	0.002201	0.029318	Arl5c	UP
230.026	5.109204	1.669094	3.061064	0.002206	0.029356	Cir1	UP
67.07444	-3.30004	1.078332	-3.06032	0.002211	0.029356	Fra10ac1	DOWN
581.3852	7.471244	2.440945	3.0608	0.002207	0.029356	Gabarapl1	UP

2619.49	6.62092	2.163431	3.06038	0.002211	0.029356	Nes	UP
457.3599	6.277683	2.051936	3.059396	0.002218	0.029421	Sgpp2	UP
98.79047	-3.8646	1.263613	-3.05837	0.002225	0.029487	B930095G15Rik	DOWN
906.8108	-6.35754	2.079175	-3.05772	0.00223	0.029487	Rb1	DOWN
1077.241	-6.24304	2.041678	-3.0578	0.00223	0.029487	Rrm2	DOWN
1323.813	-6.37416	2.084387	-3.05805	0.002228	0.029487	Tmem167	DOWN
583.3047	5.891412	1.927347	3.056747	0.002238	0.029533	Dnajb2	UP
99.58579	3.772987	1.234304	3.056772	0.002237	0.029533	Psen2	UP
802.6492	-6.26633	2.05047	-3.05604	0.002243	0.029551	Cdca7	DOWN
446.9373	-5.80947	1.901119	-3.05581	0.002245	0.029551	Plpp3	DOWN
122.6894	3.97838	1.301693	3.056312	0.002241	0.029551	Poll	UP
1841.682	6.828483	2.235418	3.054679	0.002253	0.029588	Mvb12a	UP
623.4419	5.857141	1.917329	3.054844	0.002252	0.029588	Nub1	UP
28.33679	-2.54933	0.834497	-3.05493	0.002251	0.029588	Yeats4	DOWN
1727.01	-6.29577	2.061674	-3.05372	0.00226	0.029634	Klhdc2	DOWN
184.6153	-4.80066	1.571989	-3.05388	0.002259	0.029634	Rnd3	DOWN
1770.748	6.48046	2.122438	3.05331	0.002263	0.02965	Pa2g4	UP
64.94817	-2.75221	0.901468	-3.05303	0.002265	0.029652	Zbed5	DOWN
2478.566	-6.82706	2.236543	-3.0525	0.002269	0.02968	Obsl1	DOWN
1727.401	6.800601	2.229222	3.050662	0.002283	0.029838	4930453N24Rik	UP
49.34515	-2.51289	0.823846	-3.0502	0.002287	0.029859	Slf1	DOWN
117.9566	-3.49191	1.145308	-3.04889	0.002297	0.029965	Xrn2	DOWN
1319.547	6.58418	2.159872	3.048412	0.002301	0.029978	Crlf3	UP
2217.208	-6.39756	2.098847	-3.04813	0.002303	0.029978	Grpel1	DOWN
161.6176	-4.10066	1.34536	-3.048	0.002304	0.029978	Sos1	DOWN
979.4387	-6.66982	2.188692	-3.0474	0.002308	0.030014	Zfp422	DOWN
1034.544	6.524719	2.141586	3.046676	0.002314	0.030061	Pak1	UP
1653.293	-7.11621	2.336182	-3.04609	0.002318	0.030095	Zbtb17	DOWN
50.20082	-2.07085	0.679979	-3.04546	0.002323	0.03011	Mtch1	DOWN
2643.215	6.558389	2.153511	3.045441	0.002323	0.03011	U2surp	UP
298.0638	5.467514	1.795727	3.044735	0.002329	0.030121	Cd34	UP
1821.535	-5.5474	1.822048	-3.04459	0.00233	0.030121	Klhl9	DOWN
295.7737	5.400318	1.773659	3.044733	0.002329	0.030121	Xaf1	UP
397.476	-5.36711	1.763484	-3.04347	0.002339	0.030209	Gtf2h2	DOWN
1849.326	-6.58498	2.164025	-3.04293	0.002343	0.030238	Slc12a6	DOWN
32.8635	-2.39666	0.787822	-3.04214	0.002349	0.030293	Mcm5	DOWN
1006.462	6.126096	2.014956	3.040312	0.002363	0.030452	Epm2aip1	UP
951.8205	6.478483	2.131801	3.038972	0.002374	0.030563	Snrk	UP
191.4235	4.950551	1.629192	3.038654	0.002376	0.03057	S100a16	UP
758.6512	-6.28088	2.067173	-3.03839	0.002378	0.030572	Stard8	DOWN
602.6356	-6.22281	2.048313	-3.03802	0.002381	0.030585	Ldlrad3	DOWN
2508.713	6.570046	2.163435	3.036859	0.002391	0.030652	Setd7	UP
107.6912	3.947948	1.299997	3.036891	0.00239	0.030652	Trim30a	UP
1445.163	6.407232	2.110107	3.036449	0.002394	0.030669	Ptpn1	UP
260.3907	-6.10197	2.009761	-3.03617	0.002396	0.030673	Serp2	DOWN
2008.628	6.534945	2.153094	3.035142	0.002404	0.030752	Adsl	UP
96.19521	-3.07251	1.012407	-3.03485	0.002407	0.030757	Trappc2	DOWN
1480.178	5.317064	1.753237	3.032713	0.002424	0.030825	Bclaf1	UP
874.5355	6.790181	2.238534	3.033315	0.002419	0.030825	Endog	UP
171.9654	4.661328	1.536699	3.033339	0.002419	0.030825	Gm43775	UP
2206.017	6.55935	2.162731	3.032901	0.002422	0.030825	Gna11	UP
995.5865	6.219519	2.050323	3.033434	0.002418	0.030825	Ptpn12	UP
1954.598	-5.62832	1.855669	-3.03304	0.002421	0.030825	Rbbp7	DOWN
218.3155	4.523798	1.491813	3.032417	0.002426	0.03083	Mbnl3	UP

895.1841	-6.33687	2.090225	-3.03167	0.002432	0.030882	Lsm3	DOWN
223.7039	-4.93028	1.627434	-3.02948	0.00245	0.031075	Hnrnph3	DOWN
2865.93	6.377219	2.105181	3.029297	0.002451	0.031075	Itga6	UP
632.9068	-5.68792	1.877873	-3.02892	0.002454	0.031089	Mad2l1	DOWN
1152.295	-6.73416	2.223998	-3.02795	0.002462	0.031164	Faim	DOWN
338.4404	-5.35874	1.769996	-3.02755	0.002465	0.031181	Mettl4	DOWN
515.9972	-5.82128	1.923096	-3.02704	0.00247	0.031185	Dtwd2	DOWN
2158.258	6.762318	2.233984	3.027021	0.00247	0.031185	Smad3	UP
153.6214	-4.09593	1.35335	-3.02651	0.002474	0.031212	Eci2	DOWN
1389.612	6.670145	2.20455	3.025626	0.002481	0.031279	Ndufb6	UP
260.5322	-4.3029	1.423561	-3.02263	0.002506	0.031435	Ap3s2	DOWN
19.50749	-2.57707	0.852413	-3.02327	0.002501	0.031435	Crtap	DOWN
1189.734	-6.43058	2.127385	-3.02276	0.002505	0.031435	Fbxw11	DOWN
2983.285	6.294037	2.082447	3.022423	0.002508	0.031435	Jarid2	UP
1042.717	-6.39085	2.114465	-3.02244	0.002507	0.031435	Slc18b1	DOWN
590.4147	5.972849	1.975409	3.023602	0.002498	0.031435	Tcaim	UP
3525.626	5.534525	1.830421	3.023635	0.002498	0.031435	Usp22	UP
15.83507	-3.10285	1.026908	-3.02154	0.002515	0.031501	Gm14410	DOWN
1528.279	6.949647	2.300344	3.021134	0.002518	0.031519	Tmem250-ps	UP
1800.279	6.312283	2.0897	3.020665	0.002522	0.031543	Sart3	UP
339.2547	5.46878	1.810831	3.020039	0.002527	0.031558	Dnlz	UP
239.331	5.276009	1.746873	3.020259	0.002526	0.031558	Mutyh	UP
184.5357	4.231263	1.401412	3.019286	0.002534	0.031611	Nkapd1	UP
668.9303	-5.91229	1.958468	-3.01883	0.002538	0.031633	Mybl1	DOWN
2345.103	6.394277	2.118472	3.018344	0.002542	0.031659	Zfp217	UP
1327.169	-6.42277	2.128269	-3.01784	0.002546	0.03167	Plagl1	DOWN
1421.819	-6.71187	2.224124	-3.01776	0.002547	0.03167	Prkca	DOWN
46.64259	-2.3286	0.771755	-3.01728	0.002551	0.031695	Gli3	DOWN
812.0692	6.155807	2.040441	3.016901	0.002554	0.03171	Anxa2	UP
2890.47	6.249294	2.072229	3.015735	0.002564	0.031757	Cald1	UP
91.85265	3.35888	1.113725	3.015898	0.002562	0.031757	Prmt2	UP
2785.327	6.513711	2.15963	3.016123	0.00256	0.031757	Ssu72	UP
1796.875	6.49575	2.154124	3.015495	0.002566	0.031757	Rab3d	UP
3206.537	6.541149	2.169934	3.014447	0.002574	0.031811	Rae1	UP
1072.042	6.225573	2.06537	3.014264	0.002576	0.031811	Tmed9	UP
519.6982	6.135875	2.035503	3.014427	0.002575	0.031811	Tppp3	UP
17.72915	-2.72076	0.903122	-3.01262	0.00259	0.031909	Dimt1	DOWN
1464.206	5.964077	1.97963	3.012723	0.002589	0.031909	Hsph1	UP
1761.48	-6.35082	2.10776	-3.01307	0.002586	0.031909	Mthfsd	DOWN
381.6401	-5.83334	1.937281	-3.0111	0.002603	0.031916	Gatb	DOWN
57.98288	-2.76846	0.919293	-3.01151	0.0026	0.031916	Gm9616	DOWN
3089.973	-6.54116	2.172502	-3.01089	0.002605	0.031916	Itm2b	DOWN
983.1535	-6.40929	2.128583	-3.01106	0.002603	0.031916	Nup12	DOWN
1456.046	6.654089	2.209494	3.011589	0.002599	0.031916	Pcolce	UP
2245.839	6.914885	2.296089	3.011593	0.002599	0.031916	Samd10	UP
3084.317	6.158874	2.044961	3.011731	0.002598	0.031916	Tmcc1	UP
335.8121	-5.37688	1.785995	-3.01058	0.002607	0.031923	Ppme1	DOWN
320.8564	5.518269	1.833483	3.009719	0.002615	0.031967	Nxn	UP
628.1523	6.150019	2.043401	3.009697	0.002615	0.031967	Poc1b	UP
120.6085	3.205373	1.065695	3.007777	0.002632	0.032144	Cirbp	UP
1467.55	6.428852	2.138051	3.006875	0.002639	0.032215	Mrpl51	UP
130.783	3.91865	1.303832	3.005486	0.002652	0.032287	Khnyn	UP
3046.496	-5.49007	1.82665	-3.00554	0.002651	0.032287	Srsf11	DOWN
1243.675	-6.3791	2.122317	-3.00572	0.002649	0.032287	Thap1	DOWN

64.49876	-5.99424	1.995042	-3.00457	0.00266	0.032309	Gm48170	DOWN
1652.35	6.239012	2.076415	3.004704	0.002658	0.032309	Tbc1d10b	UP
208.8245	-3.93646	1.310093	-3.00472	0.002658	0.032309	Zfp367	DOWN
666.3862	-6.00208	1.998106	-3.00389	0.002666	0.032313	Pgghg	DOWN
1405.117	6.858419	2.283031	3.004085	0.002664	0.032313	Pygo2	UP
14.95797	3.16318	1.053047	3.003835	0.002666	0.032313	Vmn2r118	UP
1069.688	-6.4343	2.143266	-3.0021	0.002681	0.032427	Fhl4	DOWN
23.58024	-2.33268	0.776941	-3.00239	0.002679	0.032427	Mindy2	DOWN
1037.354	6.33988	2.111847	3.002054	0.002682	0.032427	Pex1	UP
51.24217	-2.37222	0.790432	-3.00117	0.002689	0.032496	Cdk2ap2	DOWN
524.5943	-5.93353	1.977259	-3.00088	0.002692	0.032502	Vstm4	DOWN
158.5579	-4.29037	1.429993	-3.00027	0.002697	0.032543	Rnf181	DOWN
1207.237	-6.41718	2.139389	-2.99954	0.002704	0.032571	A430035B10Rik	DOWN
1362.879	-6.41659	2.139081	-2.99969	0.002703	0.032571	Dyrk1a	DOWN
2379.098	-6.15378	2.052818	-2.99772	0.00272	0.03274	Camta1	DOWN
4531.298	-6.16633	2.058503	-2.99554	0.00274	0.032929	Psma7	DOWN
642.9439	-5.79996	1.936218	-2.99551	0.00274	0.032929	Vps37a	DOWN
3160.429	6.290882	2.100845	2.994453	0.002749	0.033018	Epb41l5	UP
1631.36	-6.17518	2.062531	-2.99398	0.002754	0.033043	Ncor1	DOWN
3743.274	-5.58275	1.86491	-2.99358	0.002757	0.033051	Fam168b	DOWN
14.22487	3.049991	1.018967	2.993218	0.002761	0.033051	Kcnj2	UP
252.0156	-4.87108	1.627292	-2.99337	0.002759	0.033051	Prpf38a	DOWN
1370.064	-5.36709	1.793587	-2.99238	0.002768	0.033083	Mphosph6	DOWN
1344.486	6.502032	2.172977	2.992223	0.00277	0.033083	Pcmt1	UP
2525.668	6.490019	2.168781	2.992473	0.002767	0.033083	Slc4a1ap	UP
22.98547	-3.06401	1.024177	-2.99168	0.002774	0.033096	Gm3333	DOWN
432.0391	-5.58498	1.866867	-2.99163	0.002775	0.033096	Txn1	DOWN
521.3097	6.051735	2.023128	2.991277	0.002778	0.03311	Pop4	UP
661.0032	6.072402	2.030297	2.990893	0.002782	0.033127	Rdh10	UP
1936.77	5.769103	1.929213	2.990392	0.002786	0.033131	Foxj3	UP
3325.897	6.162346	2.060628	2.990518	0.002785	0.033131	Usp34	UP
1412.27	-6.73272	2.253049	-2.98827	0.002806	0.033311	2210016L21Rik	DOWN
2088.993	6.328731	2.117763	2.988404	0.002804	0.033311	Zzz3	UP
145.93	-4.5903	1.536575	-2.98736	0.002814	0.033335	Atp6ap1	DOWN
1038.419	5.414936	1.812528	2.987505	0.002813	0.033335	Brd2	UP
32.17419	2.58443	0.865011	2.987743	0.00281	0.033335	Mfap4	UP
1294.696	6.197109	2.074638	2.98708	0.002817	0.033341	B230219D22Rik	UP
2192.857	6.36018	2.129986	2.98602	0.002826	0.03337	Itpkb	UP
2470.078	5.188051	1.737522	2.985892	0.002828	0.03337	Matr3	UP
22.78466	-2.42476	0.8119	-2.98652	0.002822	0.03337	Mrps15	DOWN
1777.638	6.300126	2.109878	2.986014	0.002826	0.03337	Ube2j2	UP
779.8689	-6.03821	2.022756	-2.98514	0.002834	0.033427	AW549877	DOWN
67.09608	3.155951	1.057908	2.983198	0.002853	0.033524	Dtd2	UP
2254.525	-6.58851	2.209115	-2.98242	0.00286	0.033524	Ei24	DOWN
959.2253	5.306654	1.778706	2.983436	0.00285	0.033524	Esrp1	UP
12.86389	3.731657	1.250654	2.983765	0.002847	0.033524	Gm11851	UP
103.9071	-3.8543	1.292088	-2.983	0.002854	0.033524	Gm715	DOWN
1240.885	6.132772	2.055293	2.983892	0.002846	0.033524	Micu1	UP
1440.94	6.217283	2.084643	2.982421	0.00286	0.033524	Ralgapa1	UP
299.9605	5.163865	1.731409	2.982463	0.002859	0.033524	Terb1	UP
1027.44	-6.11997	2.053383	-2.98043	0.002878	0.033717	Zfp444	DOWN
723.4299	5.900874	1.980322	2.979755	0.002885	0.033761	Lhx8	UP
1982.138	6.569805	2.20494	2.979584	0.002886	0.033761	Snx9	UP
13.47211	-3.37152	1.13173	-2.97908	0.002891	0.033791	Cnksr3	DOWN

20.34194	-2.87206	0.964251	-2.97854	0.002896	0.0338	Idi1	DOWN
2033.852	6.293355	2.112814	2.978661	0.002895	0.0338	Senp2	UP
22.9047	2.254541	0.757435	2.976546	0.002915	0.033971	Calhm4	UP
693.9723	6.12838	2.058772	2.976715	0.002914	0.033971	Kctd9	UP
409.6597	-5.9081	1.985541	-2.97556	0.002925	0.03403	Ccdc126	DOWN
29.06418	-2.60975	0.877062	-2.97556	0.002925	0.03403	Socs1	DOWN
1844.025	-6.56908	2.208796	-2.97405	0.002939	0.034172	Itgb3	DOWN
2538.705	6.333361	2.129828	2.973648	0.002943	0.034192	Acbd3	UP
240.5154	-5.05129	1.6994	-2.9724	0.002955	0.034306	Fam217a	DOWN
1842.115	-6.28699	2.116955	-2.96983	0.00298	0.034547	Bmp1	DOWN
1277.895	6.019774	2.027002	2.969792	0.00298	0.034547	Piwil2	UP
585.954	5.441605	1.832506	2.969489	0.002983	0.034556	Ociad1	UP
20.37534	2.681169	0.903464	2.967656	0.003001	0.034686	Ccno	UP
543.3693	-5.79258	1.951696	-2.96797	0.002998	0.034686	Nphp1	DOWN
4764.024	5.179539	1.745234	2.96782	0.002999	0.034686	Rest	UP
12.58881	-3.38462	1.140666	-2.96723	0.003005	0.034708	E430024P14Rik	DOWN
47.48567	1.933153	0.65176	2.966048	0.003017	0.034816	Nexmif	UP
2613.579	6.162799	2.078859	2.964511	0.003032	0.034965	Lrba	UP
355.6941	-5.43554	1.834084	-2.96362	0.00304	0.035015	Sema6d	DOWN
355.2874	-5.08016	1.714106	-2.96374	0.003039	0.035015	Slc44a1	DOWN
67.9199	-2.87182	0.969583	-2.96191	0.003057	0.035165	Prickle1	DOWN
2048.871	-6.19534	2.091706	-2.96186	0.003058	0.035165	Ralbp1	DOWN
1165.825	6.395002	2.159392	2.961483	0.003062	0.035182	Mrpl11	UP
1358.799	6.509494	2.198264	2.961198	0.003064	0.035189	Becn1	UP
868.8388	5.982242	2.020607	2.960615	0.00307	0.03521	Gramd4	UP
33.5862	-2.04504	0.690761	-2.96056	0.003071	0.03521	Pwwp2b	DOWN
525.6659	-5.58929	1.888458	-2.95971	0.003079	0.035258	Cyb5b	DOWN
22.07945	2.281011	0.770693	2.959689	0.003079	0.035258	Slc17a2	UP
316.2974	5.02143	1.696894	2.959189	0.003084	0.035269	Grb7	UP
740.8225	-5.95444	2.012393	-2.95889	0.003088	0.035269	Myl12a	DOWN
229.051	-4.80256	1.623201	-2.9587	0.003089	0.035269	Ppp2cb	DOWN
300.3973	5.452868	1.842835	2.958956	0.003087	0.035269	Rilpl1	UP
321.0421	-5.12585	1.732775	-2.95817	0.003095	0.035278	Cep41	DOWN
720.4486	-6.04836	2.044538	-2.9583	0.003093	0.035278	Wdr91	DOWN
2002.714	6.50831	2.200657	2.95744	0.003102	0.035336	Ndufs2	UP
378.7919	5.459999	1.846449	2.957026	0.003106	0.035346	Ctdp1	UP
978.3358	6.279408	2.123713	2.956806	0.003108	0.035346	Sgta	UP
185.3603	-3.92108	1.326174	-2.95669	0.00311	0.035346	Xrcc6	DOWN
821.2225	5.607993	1.897615	2.955285	0.003124	0.03543	Pik3r4	UP
86.18731	-3.32504	1.124969	-2.95568	0.00312	0.03543	Tiprl	DOWN
425.6908	5.578576	1.887588	2.955399	0.003123	0.03543	Zfp574	UP
1454.89	6.290444	2.128998	2.95465	0.00313	0.035452	Acsl1	UP
1944.674	-6.25378	2.116558	-2.95469	0.00313	0.035452	Rhbdf1	DOWN
629.1594	-5.99017	2.028036	-2.95368	0.00314	0.035538	Cdc123	DOWN
24.05456	-2.50119	0.847064	-2.95277	0.003149	0.035617	Tmem128	DOWN
1708.494	6.342556	2.148217	2.952475	0.003152	0.035626	Zbtb7a	UP
1380.583	6.468012	2.191081	2.951972	0.003158	0.035658	Chchd1	UP
935.7578	6.10926	2.069743	2.9517	0.00316	0.035664	Ppp2r3d	UP
872.4281	-5.96934	2.022692	-2.95119	0.003166	0.035698	Stau1	DOWN
1639.452	-6.31346	2.14028	-2.94983	0.003179	0.035787	Hsdl2	DOWN
6923.397	-5.23056	1.773355	-2.94953	0.003183	0.035787	Oip5os1	DOWN
1425.24	6.441316	2.183844	2.949531	0.003183	0.035787	Pacsin2	UP
200.1321	-4.17587	1.415681	-2.94973	0.003181	0.035787	Stt3b	DOWN
1800.27	6.462746	2.192537	2.947611	0.003202	0.035984	Eef2k	UP

1121.505	-6.1365	2.083173	-2.94575	0.003222	0.036176	Ndufb8	DOWN
732.6447	-6.46146	2.194522	-2.94436	0.003236	0.036313	Phf11d	DOWN
27.86376	2.396523	0.814114	2.943718	0.003243	0.036362	Ilvbl	UP
520.2357	-5.49968	1.868578	-2.94324	0.003248	0.036392	Zfp729a	DOWN
175.4212	-3.84153	1.305539	-2.94249	0.003256	0.036455	Gars	DOWN
1292.871	6.145722	2.088877	2.942117	0.00326	0.036472	Cramp1l	UP
145.6706	4.163192	1.41594	2.940233	0.00328	0.036539	Fam207a	UP
723.665	-6.10321	2.075742	-2.94025	0.003279	0.036539	Fbxo30	DOWN
1642.684	6.478194	2.203271	2.940262	0.003279	0.036539	Gm1673	UP
1876.558	6.330597	2.152746	2.940708	0.003275	0.036539	Kdm1b	UP
453.7966	-5.70539	1.940129	-2.94073	0.003274	0.036539	Klhl7	DOWN
1885.623	-6.28389	2.136873	-2.94069	0.003275	0.036539	Ulk4	DOWN
739.2967	5.946015	2.022675	2.939679	0.003286	0.036552	Pi4k2b	UP
521.6647	5.887308	2.002555	2.939898	0.003283	0.036552	Styxl1	UP
869.9079	-5.96904	2.030678	-2.93943	0.003288	0.036556	Slc4a2	DOWN
1236.867	5.145437	1.750821	2.938872	0.003294	0.036578	Fxr1	UP
712.1027	-6.068	2.064783	-2.93881	0.003295	0.036578	Ppid	DOWN
1132.802	6.635383	2.258443	2.938034	0.003303	0.036617	Crlf2	UP
360.136	5.536962	1.884575	2.938043	0.003303	0.036617	Slc47a2	UP
623.4251	5.871612	1.999631	2.936348	0.003321	0.036791	Lmbrd1	UP
535.7986	5.748975	1.958628	2.935204	0.003333	0.036901	Dsg2	UP
1234.818	6.175073	2.104179	2.934671	0.003339	0.036935	Eif3l	UP
26.62378	2.102245	0.716395	2.934477	0.003341	0.036935	Podxl2	UP
979.0997	6.23623	2.125615	2.933847	0.003348	0.036974	Eif3h	UP
187.9893	4.712388	1.606285	2.933719	0.003349	0.036974	Fhl2	UP
640.6466	-5.98251	2.039549	-2.93325	0.003354	0.037003	Nup62	DOWN
1903.271	-6.18378	2.108377	-2.93296	0.003358	0.037013	Atg4b	DOWN
3471.333	5.805715	1.979908	2.932316	0.003364	0.037063	Tbca	UP
780.1336	-6.24405	2.129602	-2.93203	0.003368	0.037072	Sec61a2	DOWN
207.3104	-4.65935	1.589786	-2.9308	0.003381	0.037127	Gna12	DOWN
52.24407	-2.01681	0.688096	-2.931	0.003379	0.037127	Kpna2	DOWN
16.11769	-2.99403	1.021497	-2.93102	0.003379	0.037127	Rps2-ps7	DOWN
2488.103	-6.09788	2.080698	-2.93069	0.003382	0.037127	Sipa1l1	DOWN
81.91892	-3.72699	1.271987	-2.93006	0.003389	0.037152	Fdps	DOWN
653.527	4.884365	1.66699	2.930051	0.003389	0.037152	Tnks	UP
616.2348	5.053064	1.724814	2.929628	0.003394	0.037177	Zcchc2	UP
4307.886	5.446545	1.859541	2.928973	0.003401	0.037229	Tex15	UP
1846.007	6.32498	2.159632	2.928731	0.003403	0.037232	Drosha	UP
43.48522	2.710711	0.925663	2.928398	0.003407	0.037246	Oaz2	UP
290.1878	5.067775	1.731291	2.927166	0.003421	0.037368	Chchd6	UP
1001.335	-6.11542	2.089522	-2.92671	0.003426	0.037381	Lancl1	DOWN
896.681	-5.20158	1.777331	-2.92662	0.003427	0.037381	Ube2i	DOWN
1464.048	6.312437	2.157069	2.926395	0.003429	0.037383	Mcph1	UP
454.4365	-5.68703	1.943519	-2.92615	0.003432	0.037386	Timm23	DOWN
220.8065	-4.33071	1.480273	-2.92562	0.003438	0.037425	Hmces	DOWN
285.8218	-5.05252	1.727294	-2.92511	0.003443	0.03746	Gas2	DOWN
6662.622	4.810269	1.644633	2.924829	0.003446	0.037468	H19	UP
53.36528	-2.38874	0.816782	-2.92457	0.003449	0.037473	Cyp4f17	DOWN
2979.764	6.355592	2.173372	2.9243	0.003452	0.03748	Ncapg	UP
471.3216	4.966485	1.698629	2.923819	0.003458	0.037486	Nme1	UP
1521.205	6.517911	2.229109	2.923999	0.003456	0.037486	Rad50	UP
1613.561	6.055749	2.07186	2.922856	0.003468	0.037576	Snrnp70	UP
925.0748	-5.85858	2.005183	-2.92172	0.003481	0.037688	Fkbp3	DOWN
2338.518	6.368617	2.180231	2.921075	0.003488	0.037737	Bmpr1a	UP

126.1837	-3.9439	1.350241	-2.92088	0.00349	0.037737	Dusp10	DOWN
1465.883	6.854472	2.346941	2.920598	0.003494	0.037745	Mfsd1	UP
23.93034	-2.21064	0.757147	-2.91971	0.003504	0.037828	Nup35	DOWN
1389.211	6.561417	2.24774	2.919117	0.00351	0.037873	Hacd1	UP
689.9554	-6.0411	2.069882	-2.91857	0.003516	0.037913	Cstf3	DOWN
733.2054	-5.74059	1.967083	-2.91833	0.003519	0.037917	Zfp704	DOWN
2452.231	6.412136	2.198124	2.917095	0.003533	0.038041	Nfrkb	UP
1068.932	-6.00437	2.058948	-2.91623	0.003543	0.038087	Polr3h	DOWN
1052.444	-5.62942	1.930552	-2.91596	0.003546	0.038087	Rheb	DOWN
1923.682	6.242304	2.140804	2.915869	0.003547	0.038087	St7	UP
474.5947	5.581527	1.913907	2.9163	0.003542	0.038087	Zfp280b	UP
203.9907	5.208337	1.786541	2.915318	0.003553	0.038128	1700007K13Rik	UP
476.1595	-5.75654	1.974947	-2.91478	0.003559	0.038168	Sdhaf3	DOWN
828.8904	-5.93424	2.037148	-2.91301	0.00358	0.038332	Ghitm	DOWN
65.1431	-2.66562	0.915046	-2.9131	0.003579	0.038332	Zfp9	DOWN
76.61662	-3.06788	1.053347	-2.91251	0.003585	0.038365	Atp6v1f	DOWN
790.1525	-5.23114	1.796212	-2.91232	0.003588	0.038365	Tex264	DOWN
621.9257	5.817649	1.998158	2.911506	0.003597	0.038439	C1d	UP
1018.743	-5.97032	2.051409	-2.91035	0.00361	0.038555	Abcg2	DOWN
234.7901	-5.03775	1.732219	-2.90826	0.003634	0.038787	Ppt1	DOWN
276.7123	-4.1979	1.444542	-2.90604	0.00366	0.039037	Hbp1	DOWN
44.27684	2.190066	0.753813	2.905319	0.003669	0.039066	Jun	UP
453.8431	5.437517	1.871483	2.905459	0.003667	0.039066	Rgl2	UP
2011.291	6.169558	2.123648	2.90517	0.003671	0.039066	Rpl22	UP
291.5635	-5.51768	1.900209	-2.90372	0.003688	0.039221	Slc35a1	DOWN
485.6642	-5.72739	1.973373	-2.90234	0.003704	0.039368	Snip1	DOWN
659.3879	6.084598	2.096607	2.902116	0.003707	0.039369	Otulin	UP
2892.544	6.064671	2.090256	2.901402	0.003715	0.039432	Ralgps2	UP
1312.254	6.420531	2.213244	2.900959	0.00372	0.039461	Sirpa	UP
1264.589	6.310676	2.176935	2.898881	0.003745	0.039644	Ctc1	UP
1085.787	5.976685	2.061677	2.898943	0.003744	0.039644	Rbm22	UP
789.3628	-6.36264	2.194662	-2.89914	0.003742	0.039644	Zfp760	DOWN
96.25297	3.317843	1.144671	2.898513	0.003749	0.039664	Ccdc149	UP
615.7417	-5.89616	2.034665	-2.89785	0.003757	0.03972	Zcchc10	DOWN
897.5165	5.919036	2.042735	2.897604	0.00376	0.039725	Mob1a	UP
1381.381	-5.98868	2.068886	-2.89464	0.003796	0.040075	Ptpra	DOWN
2417.038	-6.50441	2.247373	-2.89423	0.003801	0.040101	Intu	DOWN
250.2965	4.553747	1.573907	2.893277	0.003812	0.040196	Thop1	UP
2351.894	6.23708	2.156582	2.892113	0.003827	0.040318	Phf3	UP
1760.505	-6.25306	2.162318	-2.89183	0.00383	0.040327	Zfp821	DOWN
1633.794	6.217701	2.150581	2.891173	0.003838	0.040385	Cep290	UP
2025.403	6.091087	2.107067	2.890789	0.003843	0.040407	Fbn2	UP
843.6745	-5.68368	1.96653	-2.89021	0.00385	0.040455	Ndufb9	DOWN
73.09293	2.901762	1.004161	2.889737	0.003856	0.040488	Wdr89	UP
850.9765	6.156532	2.131181	2.888789	0.003867	0.040583	Mrpl12	UP
39.75048	2.512823	0.871131	2.884554	0.00392	0.041106	Plod3	UP
21.29872	2.45484	0.851254	2.883793	0.003929	0.041178	Tyrobp	UP
800.6271	5.776676	2.003339	2.883524	0.003933	0.041185	Fxr2	UP
1265.56	-6.06101	2.102847	-2.88229	0.003948	0.041237	Apc	DOWN
338.8549	5.33655	1.852008	2.881494	0.003958	0.041237	Dach1	UP
185.0593	-4.17314	1.44819	-2.88163	0.003956	0.041237	Eif2s1	DOWN
600.4248	6.020773	2.08867	2.882586	0.003944	0.041237	Mreg	UP
77.48524	-2.6288	0.912318	-2.88145	0.003958	0.041237	Naa25	DOWN
3680.498	6.16122	2.137764	2.882086	0.003951	0.041237	Nexn	UP

817.0375	-6.04999	2.098896	-2.88247	0.003946	0.041237	Pmp22	DOWN
99.47506	-3.25634	1.129874	-2.88204	0.003951	0.041237	Tex9	DOWN
489.3666	5.617873	1.94999	2.880975	0.003964	0.041272	Vat1	UP
1460.015	-5.5982	1.945277	-2.87784	0.004004	0.041657	S100pbp	DOWN
858.8938	-5.84246	2.031087	-2.87652	0.004021	0.041804	Hadhb	DOWN
794.1218	-5.84382	2.032599	-2.87505	0.00404	0.04194	Dpy30	DOWN
128.0361	-3.43705	1.19555	-2.87487	0.004042	0.04194	Mis18bp1	DOWN
127.4518	-3.8782	1.348963	-2.87495	0.004041	0.04194	Pipox	DOWN
1696.939	6.068465	2.111041	2.874632	0.004045	0.041944	Tom1l2	UP
25.05593	-2.66461	0.92733	-2.87342	0.004061	0.042078	Gm10699	DOWN
1727.049	6.405378	2.229441	2.873087	0.004065	0.042094	Pin1	UP
31.79485	-2.01775	0.702367	-2.87278	0.004069	0.042107	Srrm4	DOWN
14.87263	-2.78574	0.970255	-2.87114	0.00409	0.04227	Mtpn	DOWN
559.6135	-4.99597	1.740057	-2.87115	0.00409	0.04227	Zkscan3	DOWN
1269.601	6.165733	2.14805	2.870386	0.0041	0.042316	Gtf2ird2	UP
591.3383	-5.90252	2.056318	-2.87043	0.004099	0.042316	Stk17b	DOWN
196.6883	-4.65335	1.621339	-2.87006	0.004104	0.042331	Oser1	DOWN
316.6139	-4.97368	1.733264	-2.86955	0.004111	0.042373	Rnft1	DOWN
2818.684	6.179196	2.154077	2.868605	0.004123	0.042444	Serpine2	UP
1508.65	5.943583	2.071923	2.868631	0.004123	0.042444	Supt6	UP
1398.641	6.04102	2.10668	2.867555	0.004137	0.042484	Bfar	UP
2005.562	6.225136	2.170946	2.867477	0.004138	0.042484	Dvl3	UP
591.5784	6.275868	2.18855	2.867592	0.004136	0.042484	Gpx7	UP
1330.639	5.051546	1.761527	2.867708	0.004135	0.042484	Ptprf	UP
1745.83	-5.91972	2.064981	-2.86672	0.004148	0.042558	Esco2	DOWN
157.3305	4.068536	1.419443	2.866292	0.004153	0.042588	Hdac8	UP
250.8762	-4.26251	1.487437	-2.86568	0.004161	0.042643	Ctnnb1	DOWN
125.8465	-4.11032	1.434474	-2.86539	0.004165	0.042654	8430429K09Rik	DOWN
678.4268	6.041809	2.110091	2.863294	0.004193	0.042909	Stt3a	UP
187.0922	-3.73251	1.303786	-2.86282	0.004199	0.042945	Ube2e3	DOWN
1113.736	6.132412	2.14283	2.861829	0.004212	0.043024	Ganab	UP
13.73507	7.360718	2.571913	2.861962	0.00421	0.043024	Gm22003	UP
212.9123	4.654457	1.626877	2.860976	0.004223	0.043111	1700022I11Rik	UP
1818.384	6.289828	2.198801	2.860572	0.004229	0.043138	Vegfb	UP
118.9892	3.759831	1.314474	2.860331	0.004232	0.043143	Shcbp1l	UP
1577.925	6.121696	2.141228	2.858965	0.00425	0.043301	Banp	UP
33.49278	-2.0401	0.713705	-2.85846	0.004257	0.043342	Alpl	DOWN
772.047	4.927463	1.724352	2.857574	0.004269	0.043379	Abl2	UP
1165.045	-5.78792	2.025195	-2.85796	0.004264	0.043379	Gapdh	DOWN
20.31295	-2.33652	0.817604	-2.85776	0.004266	0.043379	Gm32486	DOWN
515.6791	5.538081	1.938505	2.856883	0.004278	0.043409	Atl3	UP
495.7255	-5.68845	1.99129	-2.85667	0.004281	0.043409	Ccdc25	DOWN
299.4282	4.935726	1.72754	2.857084	0.004276	0.043409	Tbrg1	UP
1044.636	-6.06808	2.124281	-2.85653	0.004283	0.043409	Zscan29	DOWN
148.2422	-3.6762	1.287238	-2.85588	0.004292	0.043443	Ppp1r14b	DOWN
440.3101	-4.47694	1.567613	-2.85589	0.004292	0.043443	Znrf3	DOWN
1343.812	6.157277	2.156889	2.854702	0.004308	0.043562	Arvcf	UP
460.4138	5.363264	1.878815	2.854599	0.004309	0.043562	Mocs2	UP
974.4963	-5.94472	2.082827	-2.85416	0.004315	0.043594	Smyd3	DOWN
930.1417	6.102316	2.138964	2.852931	0.004332	0.043707	Cuta	UP
1695.721	6.118828	2.144682	2.853023	0.004331	0.043707	Tnpo3	UP
1222.127	6.213478	2.178557	2.852108	0.004343	0.043793	Stx17	UP
1168.139	6.169255	2.164069	2.850767	0.004361	0.043929	Jcad	UP
29.91509	-1.94926	0.683781	-2.85071	0.004362	0.043929	Xrcc5	DOWN

2354.856	6.27856	2.202815	2.850243	0.004369	0.043957	Atad1	UP
948.9923	-5.95392	2.089067	-2.85004	0.004371	0.043957	Atp5g1	DOWN
508.0834	5.570466	1.954664	2.849833	0.004374	0.043957	Blzf1	UP
324.9249	-4.86015	1.705497	-2.8497	0.004376	0.043957	Tpst1	DOWN
1384.345	-5.75908	2.021303	-2.84919	0.004383	0.043996	Gng5	DOWN
369.487	-5.02833	1.76494	-2.84901	0.004386	0.043996	Snapin	DOWN
918.1772	-5.9865	2.101694	-2.84842	0.004394	0.044049	Rad51	DOWN
408.7957	-4.87895	1.713271	-2.84774	0.004403	0.044115	Ugcg	DOWN
663.3365	5.867299	2.061575	2.846027	0.004427	0.044325	Sirt2	UP
345.2223	-4.72229	1.659618	-2.84541	0.004435	0.044382	Pnisr	DOWN
35.17461	-2.12528	0.74741	-2.84353	0.004462	0.044561	Fam104a	DOWN
15.41135	-2.83204	0.99594	-2.84359	0.004461	0.044561	Fam122a	DOWN
296.1939	4.686279	1.648032	2.843562	0.004461	0.044561	Mark1	UP
2398.573	6.022259	2.118615	2.842545	0.004475	0.04467	Txnl1	UP
28.27423	-2.73286	0.96153	-2.8422	0.00448	0.04469	Cib2	DOWN
1284.137	5.995506	2.110025	2.841439	0.004491	0.04474	Atp6v0b	UP
273.9292	-4.76781	1.677899	-2.84154	0.00449	0.04474	G2e3	DOWN
1517.952	6.34157	2.232355	2.840754	0.004501	0.044785	Eed	UP
175.4462	4.397511	1.548033	2.840709	0.004501	0.044785	Gm16083	UP
932.103	5.663573	1.994524	2.839561	0.004518	0.044918	Zfp777	UP
1415.893	-6.04024	2.127586	-2.83901	0.004525	0.044939	Adss	DOWN
220.1279	-4.43824	1.563221	-2.83916	0.004523	0.044939	Susd4	DOWN
22.30583	2.099213	0.739831	2.837423	0.004548	0.045135	Olfr714	UP
1577.566	6.182946	2.179457	2.83692	0.004555	0.045177	Phka2	UP
1916.345	5.850366	2.062422	2.836649	0.004559	0.045187	Rsf1	UP
342.3369	4.928756	1.737754	2.83628	0.004564	0.045211	Ptms	UP
96.33461	-3.32029	1.170781	-2.83596	0.004569	0.045223	B3galnt2	DOWN
70.31461	-2.59395	0.914717	-2.8358	0.004571	0.045223	Cops7a	DOWN
1974.541	6.114732	2.156765	2.835141	0.004581	0.045258	Col20a1	UP
445.9208	-5.48848	1.935818	-2.83522	0.004579	0.045258	Cyb5a	DOWN
646.6612	-5.63248	1.98805	-2.83317	0.004609	0.045482	Cdca4	DOWN
596.5599	4.995815	1.763278	2.833255	0.004608	0.045482	Cdh2	UP
1121.299	-6.09927	2.153811	-2.83185	0.004628	0.045614	Ehd3	DOWN
13.47085	-3.29352	1.163035	-2.83183	0.004628	0.045614	Mtfr2	DOWN
2487.499	6.219834	2.196656	2.831501	0.004633	0.045633	Glyr1	UP
124.1646	-3.79597	1.340837	-2.83104	0.00464	0.04567	Ppp1cc	DOWN
238.2886	4.417502	1.56128	2.829411	0.004663	0.045875	Ddx20	UP
89.45688	-3.4045	1.203512	-2.8288	0.004672	0.045903	Nbn	DOWN
1241.827	5.084009	1.797347	2.828618	0.004675	0.045903	Ppip5k2	UP
393.4689	4.688811	1.657608	2.828661	0.004674	0.045903	Scaf8	UP
15.6187	2.801367	0.990881	2.827149	0.004696	0.046085	Barhl1	UP
1371.047	6.237366	2.206733	2.826516	0.004706	0.046129	BC024978	UP
567.2643	5.772509	2.04232	2.826447	0.004707	0.046129	Sparcl1	UP
545.3958	5.392121	1.908494	2.825327	0.004723	0.046168	Dipk2a	UP
570.2351	5.846791	2.069071	2.825805	0.004716	0.046168	Itpripl1	UP
909.0869	-6.15773	2.179512	-2.82528	0.004724	0.046168	Polr1b	DOWN
984.3406	-5.79602	2.051561	-2.82518	0.004725	0.046168	Smarcb1	DOWN
478.2541	-4.92716	1.743953	-2.82528	0.004724	0.046168	Stmn1	DOWN
588.3334	-5.13692	1.818425	-2.82493	0.004729	0.046175	Orc4	DOWN
887.7878	5.443299	1.927162	2.824515	0.004735	0.046206	Ptn	UP
480.1448	-5.27372	1.867505	-2.82394	0.004744	0.046233	Ints11	DOWN
14682.01	-5.36251	1.898957	-2.82393	0.004744	0.046233	Ndufs6	DOWN
15.09841	-2.703	0.957509	-2.82295	0.004758	0.046345	Tmem229b	DOWN
601.3937	-5.62801	1.993859	-2.82267	0.004763	0.046357	Blvrb	DOWN

52.5248	-2.1931	0.777049	-2.82234	0.004767	0.046377	Sec23a	DOWN
50.83265	-2.38464	0.845097	-2.82173	0.004776	0.046435	Pdcd2l	DOWN
250.91	4.195627	1.487001	2.821536	0.004779	0.046435	Slc5a3	UP
1003.25	-5.84113	2.071494	-2.81977	0.004806	0.046635	Map3k20	DOWN
1378.111	6.19247	2.195994	2.819893	0.004804	0.046635	Msn	UP
641.8682	-5.90531	2.095312	-2.81834	0.004827	0.046813	Rida	DOWN
352.8496	4.37036	1.551186	2.817432	0.004841	0.046888	Kdm6a	UP
546.1563	5.65652	2.007569	2.817597	0.004838	0.046888	Piga	UP
171.6463	4.013594	1.425335	2.815895	0.004864	0.047055	Fgd1	UP
171.1436	-4.35822	1.547695	-2.81595	0.004863	0.047055	Ruvbl1	DOWN
110.017	3.611257	1.282613	2.815547	0.004869	0.047077	Lcp1	UP
1237.064	-5.8502	2.078644	-2.81443	0.004886	0.047212	Csnk2b	DOWN
1788.186	-6.03883	2.146065	-2.81391	0.004894	0.04726	Ccdc88c	DOWN
4225.923	-5.81221	2.066018	-2.81324	0.004904	0.047329	Ahctf1	DOWN
869.5914	-5.80844	2.065011	-2.81279	0.004911	0.047366	Glmp	DOWN
359.8094	-5.38964	1.917409	-2.8109	0.00494	0.047579	Arc	DOWN
62.35402	2.752298	0.979202	2.810758	0.004943	0.047579	Ythdf3	UP
68.47326	2.279682	0.810957	2.811102	0.004937	0.047579	Zfp827	UP
1392.904	6.027182	2.144792	2.810147	0.004952	0.04764	Xist	UP
1061.917	6.187211	2.20192	2.809916	0.004955	0.047645	Rexo2	UP
4079.612	4.835571	1.721083	2.809609	0.00496	0.047661	Pafah1b1	UP
358.8253	-5.176	1.842699	-2.80892	0.004971	0.047711	Mettl5	DOWN
2486.625	6.108899	2.174852	2.808881	0.004971	0.047711	Setd2	UP
53.18757	2.218196	0.789806	2.808533	0.004977	0.047733	Lsm14b	UP
1466.924	-5.94786	2.118056	-2.80817	0.004982	0.047748	Ctnna1	DOWN
1464.037	6.012925	2.141322	2.808043	0.004984	0.047748	Hltf	UP
74.98336	2.65399	0.945396	2.807279	0.004996	0.047822	Hif1an	UP
1407.847	-5.92588	2.110994	-2.80715	0.004998	0.047822	Med23	DOWN
3257.277	5.725956	2.040407	2.806281	0.005012	0.047922	Ankrd17	UP
63.9074	-2.4194	0.862204	-2.80607	0.005015	0.047925	Casp9	DOWN
307.1968	4.85261	1.729896	2.805145	0.005029	0.048033	Gab3	UP
549.6386	5.592916	1.994286	2.804471	0.00504	0.048104	Inpp5a	UP
1249.254	6.128357	2.185395	2.804233	0.005044	0.048105	Fdft1	UP
363.7554	5.957724	2.124669	2.804071	0.005046	0.048105	Ldhb-ps	UP
3078.101	6.030121	2.15134	2.802961	0.005064	0.048219	Rars	UP
1296.598	6.105947	2.178421	2.802923	0.005064	0.048219	Zbtb11	UP
22.71049	-2.27411	0.81181	-2.80129	0.00509	0.048434	2410004B18Rik	DOWN
510.846	5.081585	1.814569	2.800436	0.005103	0.048533	Strn3	UP
258.2935	4.514664	1.612256	2.800215	0.005107	0.048537	Fam129a	UP
2178.29	-6.32724	2.259775	-2.79994	0.005111	0.048548	Timm10	DOWN
104.7776	3.201727	1.143594	2.799705	0.005115	0.048555	Snx19	UP
316.8517	5.266736	1.881308	2.799508	0.005118	0.048555	Rsbn1	UP
13.29501	2.874822	1.0271	2.798971	0.005127	0.048607	Gm44017	UP
1575.646	5.036243	1.800142	2.797692	0.005147	0.04877	Txndc17	UP
1144.174	6.007165	2.148312	2.796226	0.00517	0.048941	1810013L24Rik	UP
216.8051	4.21082	1.505924	2.796171	0.005171	0.048941	Lrrc28	UP
212.5772	4.085504	1.461978	2.794504	0.005198	0.049165	Khdc4	UP
1402.285	5.767401	2.064227	2.793976	0.005206	0.049187	Echs1	UP
482.1191	5.468451	1.957236	2.793966	0.005207	0.049187	Mepce	UP
1163.651	-5.86265	2.098848	-2.79327	0.005218	0.049264	Kmt2e	DOWN
1470.718	5.823647	2.085382	2.792605	0.005229	0.049335	Emsy	UP
319.2995	4.997289	1.79039	2.791174	0.005252	0.049481	Arpc3	UP
283.0294	-3.85383	1.38064	-2.79134	0.005249	0.049481	Clk1	DOWN
1006.453	-6.01838	2.156297	-2.79107	0.005253	0.049481	Gdf11	DOWN

59.36217	2.433537	0.871987	2.790795	0.005258	0.049493	Slc12a4	UP
225.4423	-4.62048	1.656442	-2.7894	0.005281	0.049528	Cyp20a1	DOWN
2052.566	5.96378	2.137854	2.78961	0.005277	0.049528	Ewsr1	UP
14.60468	2.628807	0.942217	2.790024	0.00527	0.049528	Gm50268	UP
153.6136	3.693929	1.324361	2.789217	0.005284	0.049528	Pdk1	UP
1536.83	-5.94041	2.129095	-2.79011	0.005269	0.049528	Tars2	DOWN
434.48	-5.60203	2.008376	-2.78933	0.005282	0.049528	Tax1bp3	DOWN
1284.112	5.668418	2.0321	2.789438	0.00528	0.049528	Tsc22d2	UP
232.1771	-4.60573	1.651554	-2.78872	0.005292	0.049573	Lamtor2	DOWN
13.39976	-3.01777	1.082251	-2.78842	0.005297	0.049591	Gm45407	DOWN
22.02956	-2.12145	0.761008	-2.78769	0.005309	0.049601	Hmbs	DOWN
1695.233	6.090532	2.18488	2.787582	0.00531	0.049601	Kdr	UP
77.12432	-3.02787	1.086095	-2.78785	0.005306	0.049601	Lancl2	DOWN
2309.343	5.827718	2.090429	2.78781	0.005307	0.049601	Nbea	UP
141.915	3.797584	1.36271	2.786787	0.005323	0.049605	D2hgdh	UP
492.4138	5.483488	1.967364	2.787226	0.005316	0.049605	Fmnl2	UP
377.3914	4.502908	1.61576	2.786867	0.005322	0.049605	Kidins220	UP
33.15406	1.970349	0.706993	2.786945	0.005321	0.049605	Lats1	UP
189.2801	4.28302	1.537142	2.786352	0.00533	0.049612	Ccdc102a	UP
933.7497	5.657463	2.030366	2.786425	0.005329	0.049612	Sltm	UP
2007.884	6.083152	2.183427	2.786057	0.005335	0.049624	Dusp9	UP
55.8658	2.654576	0.952864	2.785893	0.005338	0.049624	Nostrin	UP
1212.89	5.971751	2.144304	2.784937	0.005354	0.049696	Dip2c	UP
840.0886	5.736521	2.059903	2.78485	0.005355	0.049696	Irs1	UP
174.1777	3.842562	1.379661	2.785149	0.00535	0.049696	Reep3	UP
422.4582	-5.27104	1.893101	-2.78434	0.005364	0.049739	Sec11a	DOWN
99.92843	-3.31825	1.191821	-2.78418	0.005366	0.049739	Tspyl1	DOWN
293.4002	4.935338	1.772813	2.783902	0.005371	0.049753	Ss18l1	UP
117.7972	3.949731	1.419059	2.783345	0.00538	0.049809	Cwc27	UP
376.2928	-5.13561	1.845533	-2.78272	0.00539	0.04987	Mfap2	DOWN
468.4452	5.186337	1.863866	2.782569	0.005393	0.04987	Slc35g1	UP
1066.137	5.776426	2.076662	2.781592	0.005409	0.04998	Ddx6	UP
288.5557	4.779155	1.718286	2.78135	0.005413	0.04998	Sdcbp	UP
20.00232	-2.6134	0.939703	-2.78109	0.005418	0.04998	Zfp866	DOWN
899.9979	5.965969	2.145125	2.781176	0.005416	0.04998	Zfp871	UP

Table S2

Methylation levels of genomic elements in ND and HFD fetal oocytes （E13.5）

Elements	ND		HFD		P value
	**Mean**	**Median**	**Mean**	**Median**	
Promoter	3.97	1.72	6.26	2.58	9.65E-248
3'UTR	6.35	4.47	10.62	9.5	0
5'UTR	2.69	0	4.24	0.29	2.48E-91
Exon	5.84	1.25	9.75	5	0
Intron	7.2	6.32	12.15	11.81	0
CGI	1.84	0.99	2.15	0.96	0.046346128
CGI shore	4.62	3.63	7.31	6.25	0
LINE	5.33	0	8.59	0	0
SINE	4.32	0	7.37	0	0
LTR	5.82	0	8.82	0	0
DNA	4.1	0	7.08	0	0
Low complexity	0.42	0	0.63	0	5.06E-57
Satellite	2.21	0	3.5	0	9.01E-60
Simple repeat	0.77	0	1.25	0	0

Table S3

Methylation levels of genomic elements in ND and HFD fetal oocytes （E18.5）

Elements	ND		HFD		P value
	Mean	Median	Mean	Median	
Promoter	2.38	1.13	3.78	1.44	8.66E-138
3'UTR	3.43	1.29	6.1	2.9	7.15E-196
5'UTR	1.66	0	2.46	0	3.15E-05
Exon	3.19	0	5.45	0	0
Intron	3.95	2.86	7.04	5.42	0
CGI	1.46	0.96	1.71	0.98	0.000101915
CGI shore	2.66	1.9	4.45	3.16	0
LINE	3.09	0	4.71	0	0
SINE	2.33	0	3.82	0	0
LTR	3.71	0	5.14	0	0
DNA	2.13	0	3.51	0	7.78E-182
Low complexity	0.27	0	0.35	0	0.566769174
Satellite	1.21	0	1.92	0	1.17E-25
Simple repeat	0.44	0	0.65	0	6.49E-134

Table S4

Identified 2449 hyper-DMRs between ND and HFD fetal oocyte at E13.5.

dmr_ chrom	dmr_ start	dmr_ end	qval	diff	cpgs	mean_ ND	mean_ HFD	genes_name
chr8	83923164	83923526	1.3488e-0	-35.56563	19	2.4027	37.968	Adgrl1
chr14	52006790	52007004	1.35E-06	-26.1044	28	2.0536	28.158	Zfp219
chr12	107916703	10791692	1.3488e-0	-21.29121	32	2.3611	23.652	Bcl11b
chr15	76889779	76890331	1.3488e-0	-19.91505	32	1.224	21.139	Zfp7 & Gm49527
chr4	133860134	13386023	2.6719e-0	-52.76909	16	1.5625	54.332	Rps6ka1
chr15	98456535	98456726	2.6719e-0	-23.66086	24	0.86806	24.529	Olfr281
chr14	60691187	60691659	3.88E-06	-26.6861	44	5.5677	32.254	Spata13
chr7	5029668	5029973	6.1054e-0	-20.88292	33	3.1692	24.052	Gm44973 & zinc finger protein 865 & coiled-coil domain containing 106
chr6	48064244	48064499	6.1329e-0	-39.86037	27	1.1111	40.971	Zfp746
chr4	141475312	14147545	8.8077e-0	-38.53165	18	0.39683	38.928	Spen
chr17	31844782	31845127	8.8077e-0	-32.68518	27	2.4868	35.172	Sik1
chr17	56434447	56434582	8.8077e-0	-30.75906	17	0.91036	31.669	Ptprs
chr7	43938329	43938453	8.8077e-0	-27.11425	21	0.29762	27.412	Klk15 & Gm44756
chr1	36408477	36408881	8.8077e-0	-16.25843	40	3.5193	19.778	Fer1l5
chr5	123029317	12302960	1.1113e-0	-34.05919	24	0.82672	34.886	Orai1
chr8	120568290	12056855	1.1113e-0	-24.25754	24	1.4955	25.753	Gse1 & Gm20388
chr7	25342514	25342600	2.024e-05	-48.21428	14	0.89286	49.107	Megf8
chr3	89379776	89380099	2.024e-05	-21.39624	23	0.85404	22.25	Zbtb7b
chr4	1.52E+08	1.52E+08	2.16E-05	-50.7579	15	1.9444	52.702	Dnajc11
chr15	98597366	98597872	2.161e-05	-38.96275	21	2.0238	40.987	Adcy6
chr14	70139219	70139361	2.161e-05	-37.80228	17	0.78782	38.59	Ccar2
chr7	45232209	45232693	2.161e-05	-35.74404	16	1.1458	36.89	Tead2
chr7	144791226	14479216	2.161e-05	-33.31517	44	13.651	46.966	Gm34964 & Gm9711
chr11	1.01E+08	1.01E+08	2.16E-05	-22.7737	16	0.5208	23.295	Zfp385c
chr7	1.28E+08	1.28E+08	2.29E-05	-39.0789	19	1.3158	40.395	Cox6a2 & Itgad
chr8	85044161	85045109	2.309e-05	-33.22971	27	2.3148	35.545	Tnpo2
chr2	92953424	92953744	2.4695e-0	-28.61552	27	0.37037	28.986	Syt13
chr13	55156186	55156467	2.4695e-0	-21.75946	29	1.7241	23.484	Fgfr4
chr10	79733803	79734333	2.6547e-0	-27.59653	34	7.3023	34.899	Hcn2
chr18	84209396	84209946	2.6547e-0	-19.17568	38	1.7701	20.946	Zfp407
chr7	127811104	12781139	2.7338e-0	-11.58996	24	0.17361	11.764	Stx1b
chr19	55908376	55908524	2.9311e-0	-60.06746	15	1.7262	61.794	Tcf7l2
chr15	75980948	75981164	3.981e-05	-21.46141	29	3.7541	25.216	Ccdc166
chr5	140759877	14076015	4.2225e-0	-28.33512	23	1.1775	29.513	Amz1 & Gna12
chr11	1.12E+08	1.12E+08	4.76E-05	-62.7244	13	16.122	78.846	
chr19	29116403	29117353	4.9849e-0	-31.43140	21	1.5873	33.019	Rcl1
chr10	44058350	44058713	5.13E-05	-38.7698	27	0.6173	39.387	Crybg1
chr10	81498773	81498997	5.22E-05	-43.1889	16	1.0417	44.231	S1pr4
chr17	6492743	6492796	5.22E-05	-40.0484	16	0.8371	40.885	Gm26848
chr12	81690106	81690364	5.22E-05	-34.2183	20	3.75	37.968	
chr17	56069209	56069389	5.2157e-0	-32.33543	17	2.6961	35.032	Ubxn6 & Chaf1a
chr8	108949279	10894976	5.2157e-0	-28.96176	30	5.8333	34.795	Zfhx3
chr7	19388197	19388383	5.2157e-0	-27.11863	15	4.5029	31.622	Ercc2 & Gm26852
chr9	119974073	11997430	5.2157e-0	-25.95463	24	2.5647	28.519	Csrnp1 & Cx3cr1
chr1	91308762	91309144	5.2157e-0	-23.21387	31	0.40963	23.624	Scly & Gm17090
chr4	43538167	43538283	5.22E-05	-23.2	18	1.8519	25.052	Tln1
chr15	79620760	79621028	5.22E-05	-22.6518	20	0.8333	23.485	Fam227a
chr15	76435805	76436420	5.2157e-0	-22.09181	27	3.6075	25.699	Mroh1

chr17	56894968	56895139	5.2157e-0	-22.04280	17	1.0084	23.051	Mllt1
chr11	70725905	70726325	5.2157e-0	-18.80120	36	4.8457	23.647	Kif1c
chr7	1.45E+08	1.45E+08	5.68E-05	-31.4162	30	3.869	35.285	Fgf3
chr5	134522864	13452308(	7.8409e-0	-17.54951	29	4.2352	21.785	Clip2
chr19	25410071	25410184	8.4179e-0	-37.75344	15	0.30303	38.056	Kank1
chr9	75388828	75389141	8.4469e-0	-35.54533	23	5.1449	40.69	Mapk6
chr9	53884029	53884139	9.3075e-0	-20.20661	22	0.89286	21.099	Gm16380
chr4	1.34E+08	1.34E+08	9.59E-05	-30.3262	17	1.9608	32.287	Crybg2
chr8	45794962	45795178	9.6478e-0	-33.60644	17	1.1555	34.762	Sorbs2 & Sorbs2os
chr6	1.4E+08	1.4E+08	9.65E-05	-32.7418	16	0.625	33.367	Plekha5
chr2	1.63E+08	1.63E+08	0.000104	-26.8795	22	0.9091	27.789	Tox2
chr3	1.47E+08	1.47E+08	0.000112	-25.3671	12	0.8333	26.2	Ttll7
chr19	42263199	42263305	0.000119	-53.3482	12	2.0833	55.432	Golga7b
chr1	162544609	16254475	0.0001189	-43.75000	12	1.3889	45.139	Mettl13
chr4	56740548	56740769	0.0001189	-30.98415	24	7.9861	38.97	Actl7b & Gm26657
chr15	78820730	78820836	0.00013	-21.9782	30	0.5159	22.494	Gm7318
chr2	27859799	27860175	0.00013	-19.881	14	1.25	21.131	
chr4	43422069	43422254	0.0001308	-41.99175	13	1.25	43.242	Rusc2
chr4	137546355	13754666	0.0001308	-38.57586	13	2.5991	41.175	Hspg2
chr19	5685902	5686024	0.0001347	-31.33394	13	0.42735	31.761	Pcnx3
chr8	70650570	70650718	0.0001347	-21.86612	19	1.3158	23.182	Pgpep1
chr1	13113635	13114462	0.000136	-22.8801	19	4.8246	27.705	Prdm14
chr1	1.34E+08	1.34E+08	0.000143	-51.1054	14	1.1905	52.296	Fmod
chr12	81937072	81937727	0.0001430	-43.57936	15	0.47619	44.056	Pcnx
chrX	1.65E+08	1.65E+08	0.000143	-39.7098	16	3.2812	42.991	
chr5	1.19E+08	1.19E+08	0.000143	-35.501	17	0.7353	36.236	Med13l
chr17	25859134	25859216	0.0001430	-34.78836	18	1.9444	36.733	Wdr90 & Gm26694
chrX	73794920	73795103	0.000143	-25.9567	17	1.0989	27.056	Pdzd4
chr5	124214778	12421492	0.0001430	-21.32812	16	1.0417	22.37	Pitpnm2
chr13	52967605	52968036	0.0001430	-17.22011	18	0.46296	17.683	Nfil3
chr13	1.13E+08	1.13E+08	0.000143	-17.2044	29	0.2155	17.42	Ddx4
chr14	60040129	60040423	0.000145	-32.1212	22	10.72	42.841	Gm9013 & Atp8a2
chr7	144500283	14450037	0.0001456	-59.32291	16	4.4271	63.75	Ppfia1
chr2	165893015	16589359	0.0001456	-29.36233	16	2.0833	31.446	Zmynd8
chr7	25046229	25046359	0.0001533	-32.02381	24	3.8947	35.918	Grik5
chr8	71584254	71584422	0.0001533	-20.17068	32	4.5752	24.746	Slc27a1
chr15	86086587	86086974	0.0001533	-19.04017	24	4.3601	23.4	Gramd4
chr11	106017416	10601784	0.0001573	-20.04157	32	3.6272	23.669	Kcnh6
chr7	105480384	10548119	0.0001595	-22.01078	32	3.3817	25.392	Cavin3
chr13	54996164	54996506	0.0001679	-22.10427	28	2.381	24.485	Unc5a
chr7	131183620	13118394	0.0001862	-36.83035	12	0.52083	37.351	5430419D17Rik & Fgfr2
chr17	56427377	56427584	0.0002186	-59.22222	15	0.55556	59.778	Ptprs
chr17	25377550	25377668	0.000228	-32.5649	22	3.6364	36.201	Cacna1h
chr7	29529075	29529351	0.0002280	-29.84349	13	0.48077	30.324	4932431P20Rik
chr10	80000900	80001139	0.0002318	-42.71214	13	1.9231	44.635	Abca7
chr2	167128158	16712847	0.0002318	-30.59805	13	0.27473	30.873	Kcnb1
chr2	31893857	31893882	0.0002407	-38.27381	10	7	45.274	Lamc3
chr13	34746792	34746867	0.0002407	-28.54166	10	0.35714	28.899	Fam50b
chr4	1.31E+08	1.31E+08	0.000241	-38.4734	14	6.5564	45.03	
chr7	126373960	12637404	0.0002409	-33.32539	15	7.619	40.944	Spns1
chr1	36103925	36103988	0.000241	-28.5714	10	3.5714	32.143	Hs6st1
chr4	118541902	11854205	0.0002439	-28.17163	26	7.5345	35.706	Tmem125
chr16	32679789	32679873	0.0002468	-33.68254	15	6.2222	39.905	Tnk2
chr15	76174792	76174945	0.0002488	-27.69001	26	4.7024	32.392	Plec

chr10	81077975	81078516	0.000251	-19.6562	26	5.69	25.346	Thop1
chr8	122269164	12226930	0.0002514	-17.92852	21	0.34014	18.269	Gm20388
chr10	120093527	12009406	0.0002797	-24.08088	25	4.4913	28.572	Helb
chr2	1.37E+08	1.37E+08	0.000295	-25.9853	20	6.1012	32.086	Jag1
chr4	56740241	56740523	0.0002956	-21.85793	25	3.127	24.985	Actl7b & Gm26657
chr19	53009756	53010275	0.0003387	-26.87457	19	0.87719	27.752	Xpnpep1
chr18	24958885	24959308	0.0003469	-56.75595	12	2.0833	58.839	Fhod3
chr14	118597724	11859789	0.0003507	-26.13562	17	6.8371	32.973	Abcc4
chr11	116599251	11659942	0.0003507	-24.63564	22	0.5772	25.213	Rhbdf2
chr7	29008448	29009261	0.0003507	-22.99759	22	4.1288	27.126	Ryr1
chr4	62498743	62498871	0.000351	-19.2329	12	0.2604	19.493	
chr15	98835052	98835354	0.0003507	-14.33261	28	1.1905	15.523	Kmt2d
chr9	67092699	67092731	0.000352	-51.2897	12	0.6944	51.984	
chr8	119951949	11995206	0.0003590	-36.75925	18	0.79365	37.553	Usp10 &
chr16	4370323	4370507	0.0003618	-31.79157	12	2.0833	33.875	Adcy9
chr15	80235812	80235943	0.0003618	-52.36111	12	2.0833	54.444	AC140267.1 & mitochondrial elongation factor 1
chr7	128242504	12824254	0.0003618	-45.19292	12	3.266	48.459	Armc5
chr11	61313496	61313770	0.0003618	-21.91876	17	2.1569	24.076	Slc47a2
chr7	99425964	99426651	0.0003618	-20.90580	28	1.9069	22.813	Gdpd5
chr5	31059668	31059878	0.0003670	-30.08870	17	0.98039	31.069	Cad
chr15	98846705	98846861	0.000384	-28.7957	17	0.7353	29.531	Kmt2d
chr2	167106448	16710669	0.0003843	-42.18750	16	1.5625	43.75	Kcnb1
chr11	79027230	79027399	0.000384	-39.2485	16	1.5625	40.811	Gm11201 & Ksr1
chr17	47391867	47391990	0.000384	-32.6501	16	0.7813	33.431	Guca1b
chr4	1.36E+08	1.36E+08	0.000384	-29.0464	16	1.8229	30.869	
chr15	1.01E+08	1.01E+08	0.000384	-25.7626	16	4.6875	30.45	Scn8a
chr1	56796839	56797037	3.84E-04	-25.5105	15	1.6667	27.177	Satb2
chr1	1.8E+08	1.8E+08	0.000384	-25.4502	13	0.641	26.091	
chr9	121909188	12190942	0.0003843	-21.58482	16	2.567	24.152	Ackr2
chr15	89457436	89457631	0.0003843	-16.17497	23	1.5064	17.681	Mapk8ip2
chrX	52898664	52898773	0.0003843	-11.11957	13	0.96154	12.081	Rps2-ps13
chr8	1.17E+08	1.17E+08	0.000386	-40.4167	16	4.1146	44.531	
chr17	25383462	25383616	0.000394	-33.3056	15	1.75	35.056	Cacna1h
chr13	1.01E+08	1.01E+08	0.000412	-29.3513	23	1.8892	31.241	
chr7	109727837	10972811	0.0004115	-28.32913	20	2.1071	30.436	Ascl3
chr11	51644281	51644486	0.0004115	-21.01034	25	2.9841	23.994	N4bp3
chr5	137571640	13757192	0.000418	-25.53024	27	0.61728	26.148	Tfr2
chr14	1.18E+08	1.18E+08	0.00042	-36.8034	27	0.3704	37.174	
chr13	48805817	48805897	0.0004303	-44.79761	10	7.881	52.679	Phf2
chr18	75521600	75521711	0.0004333	-59.33333	10	1	60.333	Gm10532 & Ctif
chr6	1.36E+08	1.36E+08	0.000486	-23.1313	22	2.2727	25.404	Grin2b
chr19	4801604	4801748	0.000488	-17.209	33	0.3788	17.588	Rbm14 & Gm21844 & Gm21992
chr10	77041835	77042100	0.0005122	-24.91206	28	3.8479	28.76	Slc19a1
chr7	46059799	46060036	0.0005474	-35.17306	18	2.3258	37.499	Nomo1
chr3	89380429	89380914	0.0005748	-31.19161	35	4.966	36.158	Zbtb7b
chr10	3956728	3956995	0.0005748	-23.89367	29	2.0567	25.95	Plekhg1
chr14	72978515	72978841	0.0005755	-29.61666	21	1.1905	30.807	Gm9198
chr12	1.08E+08	1.08E+08	0.000591	-19.7686	32	1.3542	21.123	Ccdc85c
chr5	36204872	36205044	0.0006043	-26.27855	12	0.34722	26.626	Psapl1 & Sorcs2
chr11	54336949	54337274	0.0006043	-17.07049	29	3.9819	21.052	Acsl6
chr2	32685722	32685864	0.0006171	-13.78235	16	1.25	15.032	Fpgs
chr8	108557200	10855770	0.0006392	-56.66666	12	0.69444	57.361	Lncbate1 & Zfhx3

chr12	117184595	117184702	0.0006392	-47.77777	15	4.4444	52.222	Ptprn2
chr2	153231511	153231828	0.0006392	-41.84812	15	8.6219	50.47	Plagl2
chr8	124194593	124194873	0.0006392	-36.85267	16	3.0506	39.903	Gm20388
chr2	158829442	158829582	0.0006449	-42.84127	15	3.4444	46.286	Dhx35
chr7	16837900	16838216	0.0006673	-38.38003	14	3.2738	41.654	Strn4
chr12	84650146	84650254	0.0006673	-33.79220	15	3.1522	36.944	Vrtn
chr1	131847175	131847585	0.0006673	-32.61607	20	1.6667	34.283	Slc41a1
chr11	60480529	60480750	0.0006673	-26.12049	15	1.6364	27.757	Myo15
chr7	141191394	141191967	0.0006673	-25.87244	19	7.9323	33.805	Hras
chr11	113562573	113562783	0.0006792	-43.10515	14	1.7432	44.848	Slc39a11
chr8	70420819	70421588	0.0007136	-28.52652	19	5.6798	34.206	Crtc1
chr6	124749607	124749984	0.0007258	-30.49242	22	1.5152	32.008	Gm45234 & atrophin 1
chr7	118984026	118984209	0.0007258	-24.45792	19	3.6614	28.119	Gprc5b
chr3	100472316	100472665	0.0007869	-26.93922	19	0.65789	27.597	Tent5c
chr11	114226361	114226951	0.0007953	-36.80555	24	0.52083	37.326	Gm11690
chr10	80569007	80569097	0.0007953	-35.92857	25	6.0048	41.933	Klf16
chr11	70189607	70189731	0.000834	-55.7222	10	1.25	56.972	
chr4	1.36E+08	1.36E+08	0.000845	-79.1667	10	5.1667	84.333	
chr18	79112198	79112405	0.000845	-64.5	10	1.6667	66.167	
chr7	4146988	4147112	0.0008451	-43.50000	10	0.83333	44.333	Leng8
chr11	101458450	101458691	0.0008451	-40.76587	10	0.55556	41.321	Ifi35
chr2	1.68E+08	1.68E+08	0.000845	-39.7619	10	1.6667	41.429	Ripor3
chrX	8938117	8938164	0.000845	-38.9167	10	5	43.917	
chr1	1.82E+08	1.82E+08	0.000845	-28.9372	10	2.5	31.437	Fbxo28
chr1	83004535	83004735	0.000845	-26.6638	21	2.3044	28.968	Gm7553
chr3	90300015	90300457	0.000845	-24.1389	10	1	25.139	Gatad2b
chr4	1.35E+08	1.35E+08	0.000845	-23.4458	18	1.1243	24.57	
chr19	25409980	25410071	0.0008451	-15.03014	18	1.2346	16.265	Kank1
chr13	38022582	38022811	0.0009240	-37.52801	17	1.2745	38.803	Cage1
chr11	77471616	77471748	0.000952	-33.5449	23	1.5942	35.139	Ankrd13b
chr4	47244518	47245480	0.0009524	-25.11785	17	1.4706	26.588	Col15a1
chr19	46307057	46307915	0.0010179	-30.82695	28	3.1193	33.946	Nfkb2
chr8	71290363	71290513	0.0010365	-18.28249	16	2.2917	20.574	Myo9b
chr5	125483088	125483428	0.0010366	-54.28210	15	3.3333	57.615	Aacs
chr4	63416429	63416498	0.0010366	-50.71759	12	0.46296	51.181	Whrn
chr2	167992589	167992689	0.0010366	-49.57864	15	4.4444	54.023	Ripor3
chr13	104036843	104036921	0.0010366	-46.50000	15	1.6667	48.167	Nln
chr7	126373600	126373911	0.0010366	-44.25891	14	3.4524	47.711	Spns1
chr6	134953910	134954094	0.0010366	-44.09722	12	2.0833	46.181	Lockd
chr17	27441897	27441992	0.001037	-41.8394	12	3.7554	45.595	Grm4
chr12	110618148	110618409	0.0010366	-40.15692	22	1.8939	42.051	Dync1h1
chr2	92415922	92416266	0.0010366	-40.04273	13	2.1795	42.222	D930015M05Rik & Cry2
chr2	25625331	25625878	0.0010366	-39.09013	14	0.59524	39.685	Fcna & Fcnaos
chr7	36770411	36770571	0.0010366	-37.95222	12	1.7113	39.664	Tshz3
chr4	1.49E+08	1.49E+08	0.001037	-36.776	12	0.8333	37.609	Mtor
chr17	30527548	30527736	0.0010366	-35.72089	15	3.3333	39.054	Btbd9
chr13	51698589	51698787	0.0010366	-35.55555	15	3.3333	38.889	Sema4d
chr7	132568424	132568566	0.0010366	-34.72222	15	5.8333	40.556	Oat & Fgfr2
chr10	31248449	31248900	0.0010366	-34.22348	22	4.5455	38.769	Gm5422
chr15	74447564	74447689	0.001037	-33.4524	14	1.7857	35.238	
chr5	1.16E+08	1.16E+08	0.001037	-32.3462	12	1.0417	33.388	Gcn1l1
chr5	121372894	121373313	0.0010366	-32.30459	15	1.2222	33.527	Trafd1
chr11	98764360	98764446	0.0010366	-31.63946	12	0.83333	32.473	Thra
chr17	23834689	23834857	0.0010366	-30.97222	15	0.55556	31.528	Prss33
chr5	124114371	124114564	0.0010366	-30.92261	16	2.0833	33.006	Ogfod2
chr15	78940486	78940594	0.0010366	-30.83333	13	2.5641	33.397	Nol12

chr18	66061411	66061624	0.0010366	-30.36061	13	0.21368	30.574	Ccbe1
chr8	117078620	117079165	0.0010366	-30.18849	12	2.0833	32.272	Pkd1l2
chr4	154152384	154152587	0.0010366	-29.32720	14	1.7857	31.113	Wrap73
chr3	88368576	88369010	0.0010366	-29.09139	12	2.0833	31.175	Bglap3
chr9	122166673	122166801	0.0010366	-28.72288	15	0.37037	29.093	Snrk
chr4	43535806	43536043	0.0010366	-28.62313	15	0.66667	29.29	Tln1
chr2	168148139	168148246	0.0010366	-28.08035	16	1.0714	29.152	Gm14235
chr5	120963606	120964052	0.0010366	-27.38802	15	2.9167	30.305	Rph3a
chr12	8790788	8791050	0.001037	-27.0513	13	2.5641	29.615	Sdc1
chr12	112610650	112610915	0.0010366	-26.75602	13	0.8547	27.611	Inf2
chr4	137548364	137548456	0.0010366	-26.54331	13	2.1795	28.723	Hspg2
chr14	63746841	63747275	0.0010366	-26.19047	14	0.39683	26.587	Xkr6
chr8	120475367	120475763	0.0010366	-26.16496	14	0.89286	27.058	Gse1 & Gm20388
chr7	98064104	98064194	0.0010366	-26.01615	14	7.6233	33.639	Myo7a
chr6	90413525	90413825	0.0010366	-25.85497	15	0.55556	26.411	Cfap100
chr5	115594633	115595015	0.0010366	-25.41666	14	5.3571	30.774	Gcn1l1
chr12	112739100	112739345	0.0010366	-23.74510	14	3.5714	27.317	Cep170b
chr4	148537587	148538511	0.0010366	-23.53667	26	0.96154	24.498	Mtor
chr11	4213298	4214003	0.0010366	-20.60041	31	4.4938	25.094	Tbc1d10a
chr17	46572565	46572747	0.0010366	-19.96713	27	6.9949	26.962	Ptk7 & Gm26904
chr7	25272195	25272305	0.0010366	-19.74969	15	4.1111	23.861	Cic
chr5	92952602	92952768	0.0010537	-35.28936	19	0.98684	36.276	Shroom3
chr4	118224742	118225451	0.0010638	-16.68363	42	1.636	18.32	Ptprf
chr5	114406327	114406602	0.0010812	-25.62358	21	1.9841	27.608	Ube3b
chr9	44433852	44434615	0.001122	-24.77311	23	5.766	30.539	Foxr1
chr3	1.54E+08	1.54E+08	0.001143	-37.7532	21	6.2434	43.997	
chr4	1.56E+08	1.56E+08	0.001143	-26.7857	27	6.1728	32.959	Atad3a
chr3	55181929	55182101	0.0011465	-33.99054	25	6.7978	40.788	Sohlh2
chr8	1.25E+08	1.25E+08	0.001178	-30.1587	18	0.3968	30.556	Disc1
chr19	7281803	7282128	0.001178	-18.7638	25	1	19.764	Mark2
chr11	78695816	78696102	0.001181	-38.938	21	1.8896	40.828	Nlk
chr18	65084221	65084377	0.0012214	-37.89281	18	13.009	50.902	Nedd4l
chr6	1.27E+08	1.27E+08	0.001221	-32.3927	25	3.8502	36.243	
chr10	127540617	127540892	0.0012994	-24.07643	20	5.4643	29.541	Lrp1
chr8	124292150	124292877	0.0013312	-18.60544	28	0.44643	19.052	Galnt2 & Gm20388
chr10	75333531	75334042	0.0013766	-20.12826	28	1.3889	21.517	Adora2a
chrX	99072631	99072893	0.0014134	-32.96568	17	0.98039	33.946	Stard8
chr2	1.53E+08	1.53E+08	0.001447	-29.4954	22	1.5828	31.078	Nol4l
chr11	106690960	106691051	0.0014756	-25.52074	10	1.5476	27.068	Pecam1
chr12	117875609	117875742	0.0014779	-37.19642	10	9.6369	46.833	Cdca7l
chr17	65867618	65867910	0.0014779	-21.07638	24	3.8194	24.896	Ralbp1
chr7	34175411	34175523	0.00148	-53.3889	10	1.25	54.639	
chr18	12732594	12732707	0.0014798	-44.16025	10	12.167	56.327	Ttc39c
chr8	94673029	94673137	0.0014798	-39.12878	10	0.95455	40.083	Arl2bp
chr15	81682005	81682070	0.0014858	-36.60714	10	1	37.607	L3mbtl2 & Chadl
chr17	31447298	31447417	0.0014883	-40.61904	10	7.131	47.75	Pde9a
chr2	120494693	120494860	0.0014883	-33.54978	10	0.71429	34.264	Capn3
chr7	82771134	82771203	0.0014938	-63.45833	10	19.042	82.5	Efl1
chr17	46684210	46684477	0.001505	-21.5924	22	7.4648	29.057	Ppp2r5d & Gm26904
chr4	41119703	41120167	0.0015239	-25.08192	24	1.0417	26.124	Nol6
chr15	74556758	74557444	0.0015543	-25.50236	16	2.2197	27.722	Adgrb1
chr8	121977101	121977455	0.0016101	-33.58187	19	0.87719	34.459	Banp & Gm20388
chr11	1.19E+08	1.19E+08	0.001647	-59.5635	15	0.4762	60.04	
chr5	120600475	120600665	0.0016579	-21.18734	19	1.5602	22.747	Iqcd & Rita1

chr9	44108561	44108872	0.0016708	-23.00562	19	2.4123	25.418	C1qtnf5 & Gm20444 & Gm49380 & Rnf26 & Mfrp
chr13	9223457	9223521	0.001682	-34.5714	15	0.5556	35.127	
chr6	91783592	91783699	0.001682	-27.9221	11	1.4069	29.329	Grip2
chr10	80905133	80905407	0.001682	-13.9994	19	0.2924	14.292	Lmnb2
chr10	127714866	127715048	0.0016859	-26.19841	15	0.33333	26.532	Myo1a
chr17	47935617	47935751	0.001706	-36.2184	12	0.3788	36.597	
chr4	134666588	134666644	0.0017252	-64.16666	12	2.9167	67.083	Man1c1
chr9	46241087	46241266	0.0017707	-37.64249	13	2.1795	39.822	Gm47528 & apolipoprotein A-IV
chr12	1.01E+08	1.01E+08	0.001771	-30.7937	21	1.1905	31.984	Ccdc88c
chr16	90737433	90737564	0.00178	-31.4103	13	0.9615	32.372	
chr4	118223272	118223377	0.0017837	-35.25510	14	3.3418	38.597	Ptprf
chr17	24641854	24642060	0.001784	-26.167	13	4.8916	31.059	Slc9a3r2
chr11	120260534	120260690	0.0017837	-25.30453	12	0.59524	25.9	Bahcc1
chr8	85518019	85518147	0.0017837	-24.46428	14	2.1429	26.607	Gpt2
chr10	81269004	81269474	0.001784	-18.4167	25	1.2667	19.683	Apba3
chr17	28533500	28533633	0.0017994	-46.31944	12	0.69444	47.014	Armc12
chr1	87145976	87146156	0.001799	-35.9028	12	0.6944	36.597	Gm29374
chr10	14127512	14127989	0.001799	-29.3023	23	1.5217	30.824	Hivep2
chr17	46726289	46726360	0.0017994	-25.86288	13	3.4615	29.324	Gnmt & Gm26904
chr3	107342948	107343126	0.0017994	-15.82160	21	1.9707	17.792	Gm5279
chr13	102738042	102738474	0.0018208	-23.75000	23	4.8792	28.629	Cd180 & Mast4
chr2	166131951	166132610	0.0018375	-17.94852	18	7.6995	25.648	Sulf2
chr2	92980406	92980695	0.0018375	-17.85201	31	3.3603	21.212	Prdm11
chr11	97059880	97060085	0.0018408	-30.29100	18	0.92593	31.217	Osbpl7
chr9	44647174	44647738	0.001904	-30.7353	17	0.7353	31.471	
chr5	136836132	136836672	0.0019092	-33.98258	18	1.8519	35.834	Col26a1
chr4	1.35E+08	1.35E+08	0.001944	-25.7326	26	2.4679	28.201	
chr7	99557476	99558113	0.0020745	-20.94336	17	5.0223	25.966	Arrb1
chr12	77507767	77508091	0.00212	-54.0972	20	0.6944	54.792	
chr4	1.29E+08	1.29E+08	0.00212	-16.5848	26	3.6195	20.204	
chr5	125277363	125277939	0.002149	-34.75256	17	2.3284	37.081	Scarb1
chr10	44598982	44599340	0.002149	-26.46358	17	2.9412	29.405	Gm48065 & predicted gene, 35154
chr10	80410042	80410238	0.002149	-21.64215	17	15.571	37.213	Tcf3
chr4	1.38E+08	1.38E+08	0.002202	-29.6669	22	0.7792	30.446	Hspg2
chr6	124859016	124859208	0.0022063	-23.80880	17	4.8529	28.662	Gpr162
chr15	78676218	78676755	0.0022063	-18.32198	17	2.2059	20.528	Elfn2
chr4	41121458	41121905	0.0022207	-23.63710	22	1.9148	25.552	Nol6
chr14	63726874	63727248	0.0022207	-22.26890	17	0.58824	22.857	Mir598 & Xkr6
chr15	86194633	86194823	0.002268	-26.2343	22	7.0184	33.253	
chr4	138967161	138967584	0.0023227	-19.72883	30	1.9901	21.719	Rnf186
chr11	117140869	117141483	0.0023912	-25.64484	16	0.78125	26.426	Sec14l1
chr7	25715815	25716054	0.0024274	-23.32927	16	4.6949	28.024	Ccdc97
chr7	24557411	24557969	0.0024497	-31.64434	16	3.6012	35.246	Xrcc1
chr15	1.01E+08	1.01E+08	0.00245	-28.187	16	1.5625	29.75	
chr5	134502648	134502770	0.0024812	-30.62208	16	9.9508	40.573	Clip2
chr17	67552329	67552701	0.002497	-88.75	10	5	93.75	
chr7	114034203	114034320	0.0024971	-65.16666	10	1.6667	66.833	Spon1
chr17	26280027	26280112	0.002497	-62	10	2.5	64.5	Luc7l
chr14	122009267	122009596	0.0024971	-57.58333	10	6.25	63.833	Ubac2
chr17	34735793	34735852	0.0024971	-54.87878	10	8.1212	63	C4b
chr8	1.2E+08	1.2E+08	0.002497	-51.425	10	2.5	53.925	Adad2

chr18	80057289	80057477	0.0024971	-49.25000	10	1.25	50.5	Pard6g
chr11	119778324	119778588	0.0024971	-49.09523	10	3.3333	52.429	Rptor
chr18	67315359	67315747	0.0024971	-47.82312	21	2.5794	50.402	Impa2
chr9	121192595	121192725	0.0024971	-45.68777	10	0.83333	46.521	Ulk4
chr9	22005072	22005173	0.002497	-45.6429	10	1.6667	47.31	Prkcsh
chr13	49061397	49062059	0.002497	-45.6295	15	0.6364	46.266	Wnk2
chr4	137716557	137716871	0.0024971	-45.58928	10	0.5	46.089	Rap1gap
chr9	107193666	107193808	0.0024971	-44.95039	10	0.71429	45.665	AA543401 & Dock3
chr11	35703120	35703176	0.0024971	-44.76028	10	1.25	46.01	Slit3
chr11	1.2E+08	1.2E+08	0.002497	-42.8333	10	2.5	45.333	Slc38a10
chr8	8480645	8481057	0.002497	-42.3095	10	9.1905	51.5	Gm31135
chr1	192544870	192544952	0.0024971	-40.66666	10	2.5	43.167	Hhat
chr17	6019297	6019617	0.0024971	-40.26190	15	0.83333	41.095	Synj2
chr2	181240931	181241136	0.0024971	-40.25661	15	9.8175	50.074	Helz2
chr6	83304683	83304989	0.0024971	-39.93650	10	1	40.937	Slc4a5
chr15	8896267	8897105	0.002497	-38.4615	13	1.9231	40.385	
chr7	125555945	125556186	0.0024971	-37.29761	10	0.41667	37.714	Il4ra
chr4	152433436	152433638	0.0024971	-37.00000	15	1.1111	38.111	Kcnab2
chr2	126755007	126755149	0.0024971	-36.59523	10	4.1667	40.762	Usp8 & Usp50
chr4	58486797	58486929	0.0024971	-35.53968	10	0.55556	36.095	Lpar1
chr17	48081993	48082069	0.0024971	-35.41666	10	3.3333	38.75	CAAA01189291.2
chr7	140869064	140869729	0.0024971	-35.19190	15	2.2906	37.482	Sirt3
chr19	61257919	61258016	0.002497	-35.165	10	0.625	35.79	
chr9	56898035	56898222	0.0024971	-34.34647	16	2.0089	36.355	Cspg4
chr4	134812587	134812768	0.0024971	-33.46428	10	7.5833	41.048	Maco1
chr15	79725152	79725330	0.0024971	-33.26785	10	0.71429	33.982	Sun2
chr6	1.23E+08	1.23E+08	0.002497	-31.6641	10	1	32.664	
chr9	65556250	65556392	0.002497	-31.5259	10	0.625	32.151	Plekho2
chr3	1.01E+08	1.01E+08	0.002497	-30.9167	10	1.25	32.167	
chr8	122433834	122433996	0.0024971	-30.84680	15	0.83333	31.68	Mvd & Gm20388
chr1	34586980	34587083	0.002497	-30.6786	10	1.25	31.929	Amer3
chr4	124913950	124914102	0.0024971	-30.22023	10	0.83333	31.054	Epha10
chr12	1.12E+08	1.12E+08	0.002497	-29.1706	15	1.6667	30.837	
chr7	126961751	126962068	0.0024971	-28.42099	10	1.25	29.671	Sez6l2
chr11	106321850	106322291	0.0024971	-28.10990	23	2.3188	30.429	Scn4a
chr3	95167043	95167139	0.0024971	-27.94713	10	3.0952	31.042	Sema6c
chr8	123915140	123915194	0.0024971	-27.88690	10	2.25	30.137	Nup133 & Gm20388
chr7	68184747	68184812	0.0024971	-27.78343	15	6.6667	34.45	Igf1r
chr11	1.17E+08	1.17E+08	0.002497	-27.6327	23	3.0247	30.657	Sept9
chr9	15464463	15464643	0.002497	-27.3056	10	5	32.306	
chr7	100470416	100470801	0.0024971	-27.18698	15	1.3333	28.52	Gm10603
chr19	6070665	6070762	0.0024971	-26.60801	10	6.4127	33.021	Tm7sf2 & Vps51
chr17	33649980	33650119	0.002497	-26.2099	10	0.625	26.835	Hnrnpm
chr12	21199753	21200008	0.0024971	-26.03769	10	0.71429	26.752	Asap2
chr4	118223874	118224463	0.0024971	-24.61743	21	3.7302	28.348	Ptprf
chr7	24670099	24670261	0.0024971	-23.51340	10	3.2751	26.788	Tex101
chr15	98839390	98839581	0.0024971	-23.45514	15	3.5556	27.011	Kmt2d
chr10	96054120	96054648	0.0024971	-22.74494	10	1.6667	24.412	AC115970.1
chr4	141000708	141000757	0.0024971	-22.68813	10	2.6452	25.333	Atp13a2
chr11	61336726	61337441	0.002497	-22.1398	23	4.6964	26.836	Slc47a2
chr16	89955536	89955602	0.0024971	-21.99007	10	1.4286	23.419	Tiam1
chr19	3845555	3845656	0.002497	-21.3452	10	5	26.345	Gm16066
chr5	125050721	125050972	0.0024971	-20.53199	16	3.9062	24.438	Ncor2
chr2	156069256	156069485	0.0024971	-20.35466	16	0.78125	21.136	Spag4
chr17	24157278	24157387	0.0024971	-18.03571	10	1.6667	19.702	Amdhd2
chr5	111277366	111277531	0.0024971	-17.44576	15	2.0833	19.529	Ttc28

chr19	4154989	4156314	0.002497	-14.2485	43	1.4083	15.657	Ptprcap
chr5	1.28E+08	1.28E+08	0.002581	-40.4834	14	1.1905	41.674	Tmem132d
chr5	1.37E+08	1.37E+08	0.002581	-36.6752	14	4.4643	41.139	
chr2	119293266	119293430	0.0025823	-28.93121	14	6.6667	35.598	Vps18
chr11	1.18E+08	1.18E+08	0.00263	-24.931	33	3.453	28.384	Engase
chr19	5546361	5546798	0.002639	-31.5051	14	2.381	33.886	Gm31166
chr7	73032277	73032436	0.002639	-26.7361	12	4.1667	30.903	
chr10	127007690	127008168	0.002639	-26.40022	14	5.0028	31.403	Avil
chr12	54817989	54818400	0.00264	-25.469	11	2.2727	27.742	
chr12	99349388	99349891	0.002642	-44.8214	14	5.3571	50.179	Foxn3 & 3300002A11Rik
chr1	89713045	89713295	0.0026416	-41.00481	14	1.3393	42.344	Agap1
chr11	120067863	120068021	0.0026416	-31.46422	14	2.0604	33.525	Cep131
chr11	69448115	69448260	0.0026416	-30.79506	14	2.415	33.21	Dnah2
chr9	57796555	57796623	0.0026416	-23.67394	12	10.417	34.091	Gm17231 & AT rich interactive domain 3B (BRIGHT-like)
chr17	25384790	25385613	0.0026416	-22.37268	29	7.4035	29.776	Cacna1h
chr5	123614199	123614318	0.0026493	-26.27902	11	0.64935	26.928	Clip1
chr4	140823579	140823719	0.0026512	-35.96681	11	4.4877	40.455	Padi1
chr19	6993591	6994171	0.0026574	-39.54545	11	1.1364	40.682	Nudt22
chr19	40454466	40454867	0.0026574	-26.56450	11	2.4242	28.989	Sorbs1
chr15	99409233	99409493	0.0026574	-13.20332	13	3.8462	17.049	Tmbim6
chr3	132828724	132828846	0.0026789	-36.68831	11	14.848	51.537	Tbck
chr16	3974216	3974662	0.0026789	-28.79120	13	0.96154	29.753	Nlrc3
chr11	102001287	102001724	0.0026814	-54.12698	12	2.0833	56.21	Mpp3
chr18	81458374	81459379	0.002681	-41.8956	13	1.5385	43.434	
chr5	1.18E+08	1.18E+08	0.002681	-40.7688	12	1.3889	42.158	
chr7	105389575	105389655	0.0026814	-39.32539	12	4.1667	43.492	Fam160a2
chr9	68927590	68927658	0.0026814	-38.64748	12	3.7269	42.374	Rora
chr11	1.16E+08	1.16E+08	0.002681	-36.4927	13	0.7692	37.262	
chr2	28549377	28549470	0.0026814	-33.41922	13	4.5529	37.972	Ralgds
chr7	127343512	127343607	0.0026814	-30.40246	13	3.2051	33.608	Zfp768
chr4	125650471	125650568	0.0026814	-29.59096	13	8.4615	38.053	Grik3
chr11	68990906	68992501	0.0026814	-23.20151	40	4.6646	27.866	Pfas
chr15	85038658	85039111	0.0026814	-22.62000	13	0.29586	22.916	Fam118a
chr17	34853826	34853990	0.0026814	-20.51136	22	2.5216	23.033	Nelfe
chr1	34819608	34819962	0.002681	-19.281	13	1.2821	20.563	Fam168b
chr10	94578975	94579138	0.002687	-51.7741	12	3.836	55.61	Tmcc3os & transmembrane and coiled coil domains 3
chr1	38884371	38884850	0.0026872	-38.44471	12	1.3889	39.834	Gm37707 & carbohydrate sulfotransferase
chr11	1E+08	1E+08	0.002687	-28.8798	24	4.89	33.77	
chr6	125467328	125467573	0.0026872	-28.25595	12	3.1415	31.397	Cd9
chr10	81594367	81594433	0.002687	-25.2187	12	2.3611	27.58	Tle6
chr4	1.25E+08	1.25E+08	0.002687	-21.0681	12	0.6944	21.763	Epha10
chr2	26400118	26400913	0.0027392	-32.05357	20	1.4583	33.512	Inpp5e
chr4	85558544	85558973	0.0028483	-44.99299	17	2.9412	47.934	Sh3gl2 & Adamtsl1
chr16	18501007	18501304	0.002865	-23.0258	17	2.4664	25.492	Rtl10 & guanine nucleotide binding protein (G protein), beta polypeptide 1-like

chr10	81311574	81312011	0.0029176	-20.51628	27	9.8856	30.402	Pip5k1c
chr11	113523363	11352350[illegible]	0.0029207	-26.76937	17	2.7241	29.493	Slc39a11
chr10	43511284	43511605	0.002933	-9.71671	52	2.1337	11.85	Bend3
chr4	1.07E+08	1.07E+08	0.00296	-29.6166	17	3.8025	33.419	Ttc22
chr7	24433217	24433370	0.0032074	-18.51663	19	3.8482	22.365	Irgc1
chr10	99759096	99759302	0.003262	-26.6667	19	1.5351	28.202	Csl & Gm47578
chr15	76911339	76911609	0.0032827	-10.35499	37	1.1712	11.526	Zfp647
chr5	4825684	4825894	0.0033057	-31.03801	19	3.9474	34.985	Cdk14
chr19	5918005	5918164	0.00333	-28.4638	16	2.0833	30.547	Cdc42ep2
chr4	130926979	13092758[illegible]	0.0033355	-22.20270	19	2.6292	24.832	Laptm5
chr7	133765813	13376600[illegible]	0.0033355	-19.76190	19	3.4868	23.249	Dhx32
chr15	58028864	58029624	0.0033523	-29.69298	19	1.3158	31.009	9130401M01Rik & Gm29394
chr7	1.11E+08	1.11E+08	0.003371	-20.9286	19	1.7544	22.683	
chr5	1.24E+08	1.24E+08	0.003411	-22.3417	19	3.7531	26.095	Pitpnm2
chr11	120479590	12047983[illegible]	0.0034439	-23.78832	19	2.1961	25.984	Hgs & Hgs
chr9	51157783	51158265	0.003447	-26.2669	19	1.3158	27.583	Gm32742
chr10	29196635	29196779	0.0034737	-25.57256	21	2.466	28.039	Soga3
chr6	1.16E+08	1.16E+08	0.00351	-22.9464	16	1.0417	23.988	Gm44180
chr5	1.21E+08	1.21E+08	0.003522	-31.1083	21	4.1752	35.283	Hectd4
chr10	21304220	21305069	0.003526	-31.10750	21	1.9841	33.092	Hbs1l
chr14	31176825	31177069	0.0036114	-18.84620	21	3.7501	22.596	Nisch
chr5	67479162	67479575	0.003877	-21.0773	27	1.8695	22.947	
chr12	112176115	11217630[illegible]	0.003999	-14.34105	22	6.0606	20.402	Kif26a
chr15	80358583	80358909	0.0040315	-19.37839	15	1.5412	20.92	Cacna1i
chr5	143717025	14371727[illegible]	0.0040409	-28.15945	15	1	29.159	Usp42
chr4	149815118	14981536[illegible]	0.0040431	-28.05776	18	1.5653	29.623	Gm13064
chr2	166583138	16658328[illegible]	0.0040732	-24.13764	15	4.3957	28.533	Prex1
chr11	82901682	82901932	0.004112	-25.49713	18	1.5079	27.005	Nle1
chr6	1.12E+08	1.12E+08	0.004152	-28.7831	18	4.6627	33.446	Oxtr
chr1	72863855	72864157	0.004186	-15.9348	18	0.6944	16.629	Igfbp5
chr8	125087244	12508764[illegible]	0.004218	-20.66468	18	5.0926	25.757	Disc1
chr15	100782971	10078317[illegible]	0.0042263	-26.17504	18	0.61728	26.792	Slc4a8
chr4	56915585	56916309	0.0043743	-32.47294	22	1.1364	33.609	Tmem245
chr18	38198716	38199003	0.0043743	-26.31887	22	1.9372	28.256	Pcdh1
chr5	144877203	14487748[illegible]	0.004401	-23.87188	14	2.4008	26.273	Smurf1
chr1	36312291	36312582	0.0044706	-34.36649	14	7.1429	41.509	Arid5a
chr18	63658032	63658545	0.004483	-27.6801	14	2.9762	30.656	
chr2	24386598	24386737	0.0045015	-34.22619	14	3.869	38.095	Psd4
chr10	81048659	81048898	0.004522	-22.9208	14	1.7361	24.657	Sgta
chr5	1.14E+08	1.14E+08	0.004532	-36.7143	10	1.25	37.964	
chr16	19348129	19348218	0.004549	-77.5	10	10	87.5	
chr12	111217149	11121723[illegible]	0.0045488	-64.16666	10	11.667	75.833	Traf3
chrX	11899122	11899395	0.004549	-61.2024	10	3.2143	64.417	
chr6	128083558	12808370[illegible]	0.0045488	-58.45238	10	4.0476	62.5	Tspan9
chr5	115588826	11558905[illegible]	0.0045488	-38.75198	10	5.623	44.375	Gcn1l1
chr2	14945783	14945890	0.0045488	-38.52381	10	8.1429	46.667	Gm37437 & calcium channel, voltage-dependent, beta 2 subunit
chr18	13933755	13934267	0.004549	-35.6587	10	2.1429	37.802	Zfp521
chr7	126235782	12623592[illegible]	0.0045488	-35.45238	10	0.71429	36.167	1700123J17Rik
chr17	56767149	56767262	0.0045488	-30.87469	10	1.25	32.125	Dus3l
chr17	49926614	49927213	0.004549	-30.7464	10	1.4286	32.175	
chr6	86695983	86696180	0.0045488	-29.54040	10	2.7222	32.263	Gmcl1
chr14	51434538	51434858	0.0045488	-27.58333	10	0.83333	28.417	Gm7247
chr18	56761590	56761896	0.0045488	-27.27200	10	1.25	28.522	March3

chr7	4920445	4920513	0.0045488	-27.19642	10	1.6667	28.863	Zfp628
chr18	80418379	80418648	0.0045488	-25.92796	10	3.0556	28.984	Ctdp1
chr17	45696813	45696943	0.0045488	-24.94101	10	1.4583	26.399	Mrpl14
chr13	52968594	52968690	0.0045488	-24.82720	10	1.25	26.077	Nfil3
chr5	143555775	143555895	0.0045488	-24.77453	10	1	25.775	Fam220a
chr14	64803404	64803449	0.0045488	-23.19642	10	1.6667	24.863	Kif13b
chr11	1.03E+08	1.03E+08	0.004549	-23.1162	17	4.3056	27.422	Plcd3 & Gm26668
chr5	1.4E+08	1.4E+08	0.004549	-20.7905	10	5.8234	26.614	Ints1
chr13	54719030	54719262	0.0045488	-19.88257	10	2.2917	22.174	Cdhr2
chr8	120739841	120739889	0.0045488	-19.01388	10	5	24.014	Irf8 & Gm20388
chr19	4279825	4281467	0.0045488	-18.79984	38	4.4956	23.295	Ankrd13d
chr3	82046485	82046723	0.004549	-40.69597	13	0.54945	41.245	Gucy1b1
chr2	1.67E+08	1.67E+08	0.004549	-27.7273	25	1.5857	29.313	Ptgis
chr10	60379225	60379510	0.004581	-36.7811	13	1.6026	38.384	Cdh23
chr13	37930883	37931068	0.0045813	-21.67619	25	2.7333	24.41	Rreb1
chr11	74437783	74438175	0.004591	-41.12637	13	0.96154	42.088	Rap1gap2
chr5	121852544	121852655	0.004591	-18.11202	13	2.2436	20.356	Pheta1
chr15	89336341	89336587	0.0045977	-38.50588	13	4.1026	42.608	Miox
chr15	83909009	83909316	0.004627	-39.9374	13	3.2036	43.141	Efcab6
chr11	69430831	69430906	0.004627	-25.42582	13	4.0522	29.478	Dnah2
chr4	118623289	118623433	0.004627	-25.16435	17	7.5912	32.756	Ebna1bp2
chr11	79686308	79686511	0.0046358	-30.78435	12	0.32051	31.105	Rab11fip4
chr15	78911509	78911684	0.0046358	-26.09623	12	5.3819	31.478	Gm49510 & SH3-domain binding protein 1
chr8	70452566	70452667	0.0046358	-23.45386	17	1.208	24.662	Klhl26
chr10	69372691	69373524	0.004636	-22.0816	23	3.5826	25.664	Gm47211
chrX	99487358	99487487	0.004659	-49.4312	12	2.748	52.179	
chr17	6193518	6193762	0.0046666	-33.33333	17	7.3529	40.686	Tulp4
chr13	1.12E+08	1.12E+08	0.004667	-33.1513	17	7.8992	41.05	
chr8	11516363	11516472	0.0046666	-31.52928	12	1.6056	33.135	Cars2
chr5	1.01E+08	1.01E+08	0.004667	-29.0377	12	0.6944	29.732	
chr5	114394476	114394737	0.0046666	-28.97058	17	10.294	39.265	Ube3b
chr4	140824612	140824949	0.0046666	-26.02067	17	6.1331	32.154	Padi1
chr4	128589112	128589368	0.0046666	-22.82252	17	1.4706	24.293	Zscan20
chr2	93428871	93429669	0.0046666	-21.63377	23	0.59006	22.224	Cd82
chr2	25295818	25296291	0.004667	-20.1155	17	0.5882	20.704	Grin1 & Grin1os
chr8	35383992	35384204	0.0046666	-19.37441	17	2.9412	22.316	Ppp1r3b
chr5	33214360	33214640	0.004676	-20.0289	12	1.5046	21.533	Spon2
chr17	68150087	68150744	0.0046833	-48.24570	12	1.3889	49.635	AC151280.3
chr11	100802468	100802767	0.004686	-59.17658	12	7.1925	66.369	Stat5b
chr14	27091977	27092214	0.0046938	-45.39682	12	2.9167	48.313	Il17rd
chr7	19646751	19646908	0.0046938	-38.66402	12	1.3889	40.053	Clptm1
chr8	85096635	85096719	0.0046972	-32.75463	12	0.52083	33.275	Man2b1
chr17	34594790	34595332	0.0047147	-29.86542	23	4.9482	34.814	Pbx2
chr4	45043349	45043856	0.00479	-37.5896	23	5.723	43.313	Fbxo10
chr11	36024260	36024421	0.0053119	-40.00000	16	1.6667	41.667	Tenm2
chr9	122183381	122183949	0.0053499	-34.60869	16	4.8556	39.464	Ano10
chr8	120611243	120611832	0.0053499	-23.62283	22	2.5758	26.199	1190005I06Rik & Gm27021 & Gm20388
chr5	115602485	115602586	0.0053674	-19.29935	16	3.3854	22.685	Gcn1l1
chr4	1.36E+08	1.36E+08	0.005368	-21.9204	16	0.4808	22.401	Il22ra1
chr11	1.04E+08	1.04E+08	0.00541	-24.3417	16	1.1084	25.45	Wnt3
chr13	1.04E+08	1.04E+08	0.00543	-49.9826	16	3.6632	53.646	
chr6	1.14E+08	1.14E+08	0.00543	-26.6781	16	0.7813	27.459	
chr2	85041124	85041601	0.0054773	-33.94593	16	1.0417	34.988	Ssrp1
chr13	56422956	56423341	0.005477	-33.4673	16	1.5625	35.03	

chr1	152833745	152834353	0.0054773	-26.15530	16	3.0114	29.167	Ncf2
chr7	27580355	27580717	0.0054773	-24.75125	16	4.3837	29.135	2310022A10Rik
chr3	41596456	41596784	0.005477	-14.5275	16	1.3021	15.83	Jade1
chr16	11828126	11828380	0.0055082	-20.85813	16	5.0087	25.867	Cpped1
chr15	76174652	76174764	0.0057737	-15.27874	15	2.3165	17.595	Plec
chr4	117864722	117865528	0.0058374	-26.68335	24	4.7768	31.46	Slc6a9 & Gm17114
chr6	83770784	83771498	0.0059475	-20.12258	24	0.69444	20.817	Tex261
chr9	119694225	119694304	0.0059545	-26.41827	15	6.2576	32.676	Scn10a
chr19	6314116	6314523	0.0061898	-17.02008	15	1.6667	18.687	Cdc42bpg
chr3	152619994	152620137	0.0062138	-23.86348	15	1.4601	25.324	Ak5
chr5	1.06E+08	1.06E+08	0.006266	-35.4924	22	2.1266	37.619	Gm5987
chr17	25608255	25608447	0.0062754	-25.55250	15	2.0635	27.616	Lmf1
chr8	72268484	72268689	0.006337	-16.165	20	6.1667	22.332	
chr4	149066543	149066675	0.0063421	-40.01587	15	2.5	42.516	Pex14
chr8	22917314	22918159	0.006342	-37.0675	15	3.3056	40.373	Kat6a
chr3	67053319	67054085	0.0063421	-34.09447	18	2.6885	36.783	Rsrc1
chr5	31260711	31261469	0.0063421	-24.02290	20	1.7143	25.737	Ift172
chr6	86298692	86298956	0.006382	-40.8399	15	1.9444	42.784	
chr5	131190148	131190998	0.0063822	-37.84920	15	1.6667	39.516	Galnt17
chr4	152540146	152540322	0.0063822	-27.01455	15	1.6667	28.681	Nphp4
chr17	31215153	31215222	0.0063847	-26.34920	15	3.873	30.222	Ubash3a
chr8	126024765	126025167	0.0064069	-21.07440	20	9.8214	30.896	Kcnk1
chr19	43656988	43657161	0.006415	-24.373	15	1.1111	25.484	
chr5	145207315	145207722	0.0064199	-25.98630	14	7.1429	33.129	Zkscan5
chr2	1.74E+08	1.74E+08	0.006505	-28.9951	20	4.5833	33.578	
chr11	100565711	100565963	0.0065536	-38.38457	14	1.8323	40.217	Ttc25
chr15	83651719	83652061	0.006797	-20.8323	34	3.474	24.306	Scube1
chr12	111322782	111322997	0.0068476	-29.98120	19	8.4211	38.402	Cdc42bpb
chr5	1.01E+08	1.01E+08	0.006893	-81.25	10	3.75	85	
chr5	1.2E+08	1.2E+08	0.006893	-79.0714	10	4.4286	83.5	
chr11	74534738	74535050	0.0068931	-77.50000	10	2.5	80	Rap1gap2
chr5	67112986	67113395	0.006893	-73.4524	10	0.7143	74.167	
chr13	1.02E+08	1.02E+08	0.006893	-68.5595	10	5	73.56	
chr4	1.53E+08	1.53E+08	0.006893	-59.3777	10	1.25	60.628	
chr11	5835506	5835765	0.0068931	-58.33333	10	5	63.333	Polm
chr11	97747197	97747467	0.0068931	-58.03373	10	1.1806	59.214	Cwc25
chr1	12976903	12977238	0.006893	-56	10	2.5	58.5	Slco5a1
chr8	1.15E+08	1.15E+08	0.006893	-55.6667	10	2.5	58.167	Wwox
chr3	5826952	5827186	0.006893	-54.3849	12	1.875	56.26	
chr7	25301359	25301616	0.0068931	-52.59523	10	1.6667	54.262	Prr19
chr3	1.08E+08	1.08E+08	0.006893	-52.0635	10	2.5198	54.583	
chr11	77293458	77293495	0.0068931	-51.04166	12	2.0833	53.125	Gm12346 & Ssh2
chr15	24358219	24358594	0.0068931	-50.97222	10	1.1111	52.083	Gm36147 & AC131068.1
chr8	12872614	12872903	0.0068931	-49.94841	10	0.35714	50.306	Gm15347
chr7	141644608	141644989	0.0068931	-49.58333	10	1.25	50.833	Muc6
chr12	55501063	55501321	0.006893	-49.1667	10	3.75	52.917	
chr17	6069170	6069528	0.0068931	-48.81944	12	1.3889	50.208	Serac1
chr12	70729452	70729624	0.0068931	-48.18452	10	1.875	50.06	Gm40437
chr11	19145159	19145392	0.006893	-47.8929	10	1.6667	49.56	
chr11	78894832	78895038	0.006893	-47.7045	10	1.25	48.955	
chr6	87634170	87634293	0.0068931	-47.25000	10	2.5	49.75	Aplf
chr18	75126609	75126853	0.0068931	-46.74768	12	1.1905	47.938	Dym
chr16	13521659	13521981	0.006893	-45.0298	12	0.5952	45.625	
chr11	21976680	21976994	0.006893	-44.9735	12	1.713	46.687	
chr3	131180433	131180666	0.0068931	-44.66666	10	1.6667	46.333	Lef1
chr8	93860436	93860562	0.0068931	-44.55952	10	5.8333	50.393	Gnao1

chr18	80971404	80971506	0.0068931	-44.28736	12	0.92593	45.213	Sall3
chr13	1.01E+08	1.01E+08	0.006893	-43.75	12	1.0417	44.792	
chr4	1.18E+08	1.18E+08	0.006893	-43.6109	10	5.3295	48.94	Szt2
chr12	106508215	106508468	0.0068931	-43.14285	10	3.25	46.393	Gm17031
chr5	137637393	137637603	0.0068931	-42.66666	10	0.83333	43.5	Lrch4 & Gm20605
chr8	94517150	94517400	0.0068931	-42.50000	10	2.5	45	Nlrc5
chr18	36682757	36682988	0.0068931	-42.34693	14	1.1905	43.537	Slc35a4
chr3	1.6E+08	1.6E+08	0.006893	-42.125	10	3.9583	46.083	
chr10	60392477	60393006	0.0068931	-41.66813	27	3.2481	44.916	Cdh23
chr6	137797938	137798083	0.0068931	-41.27958	10	3.6667	44.946	Dera
chr8	95248783	95248981	0.0068931	-40.92803	12	0.46296	41.391	Cngb1
chr16	11828414	11828586	0.006893	-40.9099	14	3.4354	44.345	Cpped1
chr14	45175462	45175607	0.006893	-40.8333	10	1.6667	42.5	Txndc16
chr5	1.29E+08	1.29E+08	0.006893	-40.75	10	1	41.75	
chr19	10393912	10394169	0.0068931	-40.38311	10	0.41667	40.8	Syt7
chr17	31849641	31849795	0.0068931	-40.08333	10	1	41.083	Sik1
chr5	67398311	67398628	0.006893	-39.8309	23	1.1111	40.942	Bend4
chr4	109427725	109428158	0.0068931	-39.78571	10	2	41.786	Ttc39a
chr15	73942563	73942894	0.006893	-39.7406	14	2.6786	42.419	
chr13	93503030	93503087	0.006893	-39.6528	12	4.1667	43.819	
chr1	139470199	139470684	0.0068931	-39.50549	13	5	44.505	Aspm
chr8	94860645	94860721	0.0068931	-39.50469	10	2.5	42.005	Polr2c
chr10	75895093	75895167	0.006893	-39.4702	10	0.8333	40.304	Derl3
chr16	46897755	46898270	0.006893	-39.388	13	2.5321	41.92	
chr5	86792969	86793453	0.0068931	-39.06547	10	3.8095	42.875	Gm43055
chr3	108077682	108077855	0.0068931	-38.78968	10	3.5	42.29	Ampd2
chr15	1.03E+08	1.03E+08	0.006893	-38.7401	12	3.4127	42.153	
chrX	7939754	7939842	0.006893	-38.7222	10	1.25	39.972	Hdac6
chr1	1.36E+08	1.36E+08	0.006893	-38.6905	10	1.6667	40.357	
chr19	3339863	3340098	0.0068931	-38.64718	11	1.1364	39.784	Cpt1a
chr8	31367859	31368229	0.006893	-38.619	10	1	39.619	
chr3	1.46E+08	1.46E+08	0.006893	-38.3333	13	1.9231	40.256	
chr5	101981331	101981610	0.0068931	-38.25000	10	1.6667	39.917	Wdfy3
chr19	6911292	6911872	0.006893	-38.1819	19	2.2974	40.479	Esrra
chr4	101575110	101575312	0.0068931	-37.81250	12	5.5556	43.368	Dnajc6
chr7	24978619	24978960	0.006893	-37.6786	12	0.8333	38.512	Atp1a3
chr5	73317412	73317694	0.006893	-37.0879	13	1.5385	38.626	
chr2	30452679	30452809	0.006893	-37.004	12	2.6786	39.683	
chr8	91519328	91519396	0.0068931	-36.91666	10	3.25	40.167	Fto
chr4	139212563	139212622	0.0068931	-36.83333	10	1	37.833	Capzb
chr15	102334266	102334403	0.0068931	-36.40542	12	2.5694	38.975	Myg1
chr7	126370257	126370362	0.0068931	-36.30557	17	7.0472	43.353	Spns1
chr5	66546703	66547249	0.0068931	-36.09953	12	0.69444	36.794	Apbb2
chr15	100922241	100922470	0.0068931	-35.92857	10	1	36.929	Scn8a
chr5	38310850	38311335	0.006893	-35.7691	10	2.4306	38.2	
chr2	181649039	181649205	0.0068931	-35.47023	10	0.71429	36.185	Prpf6
chr7	68193350	68193418	0.0068931	-35.41666	10	1.25	36.667	Igf1r
chr9	62842017	62842239	0.0068931	-35.33333	10	5	40.333	Cln6 & Gm10653
chr7	58836972	58837062	0.0068931	-35.23223	19	4.0925	39.325	Gm6226
chr6	87209796	87210416	0.0068931	-34.96240	19	2.6316	37.594	Antxr1
chr7	131825767	131825895	0.0068931	-34.76785	10	2.5	37.268	Gm44546 & fibroblast growth factor receptor 2
chr15	76305675	76305793	0.006893	-34.6232	13	1.511	36.134	Oplah
chr9	119138162	119138246	0.0068931	-34.37301	10	1.6667	36.04	Dlec1
chr11	99034080	99034234	0.006893	-34.3367	14	7.7381	42.075	
chr4	45273996	45274360	0.0068931	-34.23611	12	5.2083	39.444	Frmpd1
chr16	11492154	11492402	0.006893	-34.0417	10	1.6667	35.708	Snx29

chr9	25688298	25688845	0.006893	-33.9286	14	5.9524	39.881	
chr15	80967120	80967499	0.0068931	-33.72023	14	0.89286	34.613	Adsl
chr19	6062718	6063045	0.0068931	-33.57142	14	2.381	35.952	Tm7sf2
chr11	5717929	5718088	0.006893	-33.0495	13	2.5641	35.614	Urgcp
chr7	25082087	25082304	0.0068931	-32.80555	10	1.1111	33.917	Zfp574
chr15	8269674	8269813	0.006893	-32.7202	10	0.625	33.345	Cplane1
chr11	97978523	97978714	0.006893	-32.6938	13	0.641	33.335	Plxdc1
chr5	144209536	14420984	0.0068931	-32.47023	12	4.8611	37.331	Tecpr1
chr9	111391476	11139168	0.0068931	-32.42857	10	0.83333	33.262	Trank1
chr15	38663625	38664045	0.0068931	-32.36706	10	1.8056	34.173	Atp6v1c1
chr8	122891727	12289187	0.0068931	-32.34523	10	4.1071	36.452	Gm45894 & ankyrin repeat domain 11 & Gm20388
chr4	74864461	74864606	0.006893	-32.2917	12	10.972	43.264	
chr6	71964875	71965058	0.0068931	-32.28840	13	5.0366	37.325	Polr1a
chr19	25579953	25580353	0.0068931	-32.27891	14	5.2296	37.509	Dmrt1
chr8	119749795	11975021	0.0068931	-32.27678	12	0.59524	32.872	Atp2c2
chr4	118237905	11823795	0.0068931	-32.10714	10	3.0952	35.202	Ptprf
chr13	53417981	53418380	0.0068931	-32.10353	10	2.7778	34.881	Gm2762
chr9	119379660	11937994	0.0068931	-31.86535	13	0.96154	32.827	Xylb
chr4	1.55E+08	1.55E+08	0.006893	-31.587	10	1.25	32.837	
chr12	111192047	11119293	0.0068931	-31.58333	10	1.6667	33.25	Traf3
chr4	1.17E+08	1.17E+08	0.006893	-31.0785	14	4.4643	35.543	Ptch2
chr7	100938291	10093845	0.0068931	-30.41514	13	3.2479	33.663	P2ry6
chr4	123189186	12318926	0.0068931	-30.33163	14	4.2857	34.617	Hpcal4
chr5	65300627	65301109	0.0068931	-30.05311	10	3.8526	33.906	Rfc1
chr2	92377518	92377701	0.0068931	-29.97889	10	2.381	32.36	Pex16
chr14	57699764	57699829	0.0068931	-29.96159	17	7.6857	37.647	Lats2 & Gm49361
chr15	28123174	28123479	0.0068931	-29.94709	12	3.5417	33.489	Gm8472
chr17	30874936	30875065	0.0068931	-29.60151	13	3.6769	33.278	Dnah8
chr14	55862298	55862519	0.0068931	-29.57936	10	1.6667	31.246	Nynrin
chr1	86427767	86428040	0.0068931	-29.43027	14	5.7143	35.145	Tex44
chr4	47256583	47257884	0.0068931	-29.30021	26	3.2051	32.505	Col15a1
chr11	1.17E+08	1.17E+08	0.006893	-28.994	10	1.3393	30.333	
chr4	1.31E+08	1.31E+08	0.006893	-28.9286	12	0.8333	29.762	
chr17	41195846	41196424	0.006893	-28.7454	13	3.5256	32.271	
chr5	146210498	14621081	0.0068931	-28.73051	10	1.8056	30.536	Rnf6
chr2	167977212	16797752	0.0068931	-28.71794	13	4.6154	33.333	Ptpn1
chr2	170775688	17077591	0.0068931	-28.63591	12	2.0238	30.66	Dok5
chr14	30031561	30031684	0.0068931	-28.62044	17	1.9608	30.581	Chdh
chr13	114007294	11400753	0.0068931	-28.57142	12	1.4286	30	Arl15
chr10	75739363	75739845	0.006893	-28.3318	13	3.2051	31.537	Cabin1
chrX	38456303	38456412	0.0068931	-28.26087	23	0.7971	29.058	Lamp2
chr5	125034344	12503448	0.0068931	-28.15800	10	6.0152	34.173	Ncor2
chr17	56196540	56197383	0.0068931	-28.14881	10	1.6667	29.815	Dpp9
chr6	87581612	87581737	0.0068931	-27.84748	14	3.2738	31.121	Prokr1
chr11	3997374	3997468	0.0068931	-27.77381	10	1.6667	29.44	Gal3st1
chrX	1.58E+08	1.58E+08	0.006893	-27.114	11	4.5455	31.659	
chr17	25397298	25397418	0.0068931	-26.86904	10	1.5476	28.417	Cacna1h
chr19	4731837	4731990	0.0068931	-26.81405	14	0.89286	27.707	Sptbn2
chr18	80652727	80653223	0.0068931	-26.72161	13	1.2821	28.004	Nfatc1
chr5	113751499	11375189	0.0068931	-26.41963	12	2.1465	28.566	Sart3
chr16	33123593	33123826	0.0068931	-25.60119	10	2.0833	27.685	Lmln
chr2	29404969	29405022	0.0068931	-25.47619	10	2.5	27.976	Med27
chr1	134224985	13422545	0.0068931	-25.46627	12	2.381	27.847	Adora1
chr5	122104992	12210533	0.0068931	-25.45860	12	4.7851	30.244	Myl2
chr14	66301194	66301426	0.0068931	-25.38551	10	1.6667	27.052	Trim35

chr13	49078491	49079016	0.0068931	-25.38095	10	7.5952	32.976	Wnk2
chr11	87617162	87617319	0.006893	-25.0157	19	3.5088	28.524	Hsf5
chr5	124719056	12471919[illegible]	0.0068931	-24.99956	12	1.7361	26.736	Atp6v0a2
chr17	35015743	35015857	0.0068931	-24.93882	12	1.0582	25.997	Vars
chr19	3815142	3815359	0.0068931	-24.89545	10	1.6783	26.574	Kmt5b & Gm16066
chr11	69404711	69405016	0.0068931	-24.66673	23	5.7189	30.386	Kdm6b
chr4	45772813	45773133	0.006893	-24.5252	10	3.75	28.275	
chr4	46576579	46576686	0.006893	-24.4194	12	4.9242	29.344	Coro2a
chr17	34921586	34921830	0.0068931	-24.36520	14	1.9674	26.333	Slc44a4
chr15	76180291	76180342	0.0068931	-24.27910	12	5.9441	30.223	Plec
chr14	34146544	34146623	0.006893	-24.2222	10	1	25.222	Npy4r
chr11	55381204	55381907	0.006893	-24.1494	23	4.4462	28.596	
chr15	1.01E+08	1.01E+08	0.006893	-23.4396	14	3.4226	26.862	Gm35853
chr11	5144321	5144495	0.006893	-23.2352	13	3.9194	27.155	Emid1
chr4	1.15E+08	1.15E+08	0.006893	-23.1981	14	1.7857	24.984	Gm12833
chr10	60406226	60406410	0.0068931	-23.19480	10	3.3333	26.528	Cdh23
chr12	4210002	4210305	0.0068931	-22.96131	12	4.1667	27.128	Cenpo & Adcy3
chr5	75655718	75655939	0.0068931	-22.94455	13	2.8846	25.829	Kit
chr8	110223241	11022356[illegible]	0.0068931	-22.79419	10	2.5	25.294	Cmtr2
chr4	153987815	15398802[illegible]	0.0068931	-22.20238	13	3.869	26.071	Cep104
chr11	82440462	82440587	0.0068931	-21.70653	10	2.5	24.207	Tmem132e
chr11	100746174	10074627[illegible]	0.0068931	-21.46428	10	2.5	23.964	Kcnh4
chr2	1.68E+08	1.68E+08	0.006893	-21.2628	14	3.5714	24.834	Tmem189 & Gm20431
chr8	70220717	70220939	0.006893	-20.5571	13	4.6154	25.172	Armc6
chr18	56761009	56761272	0.006893	-20.013	13	0.7692	20.782	
chr5	1.41E+08	1.41E+08	0.006893	-19.8898	27	3.6596	23.549	
chr15	76890817	76891191	0.0068931	-19.80347	21	4.8507	24.654	Zfp7 & Gm49527
chr3	51001770	51002081	0.006893	-19.7066	19	2.5877	22.294	
chr17	87781845	87782408	0.006893	-18.7286	13	3.1136	21.842	Kcnk12
chr3	1.23E+08	1.23E+08	0.006893	-18.4112	13	3.8462	22.257	Bcar3
chr7	122595026	12259542[illegible]	0.0068931	-18.30357	12	9.7222	28.026	Prkcb
chr6	112564632	11256495[illegible]	0.0068931	-17.99843	12	1.9097	19.908	Gm15519
chr5	130000875	13000106[illegible]	0.0068931	-17.54578	13	0.76923	18.315	Gusb
chr14	24477886	24478308	0.006893	-17.4675	11	4.5455	22.013	Polr3a
chr8	70771938	70772135	0.0068931	-16.64381	12	0.69444	17.338	Pik3r2
chr5	24325689	24325810	0.0068931	-16.48562	14	0.29762	16.783	Kcnh2
chr11	5874179	5874526	0.0068931	-16.30952	17	1.6457	17.955	Pold2
chr8	77447674	77447826	0.0068931	-14.77514	12	2.3752	17.15	Arhgap10
chr17	25842377	25842462	0.0068931	-14.47773	13	1.5797	16.057	Rhot2 & Gm26694
chr11	66217661	66217757	0.0068931	-9.035534	10	2.9545	11.99	Gm12296 &
chr17	89091733	89091812	0.0069	-40.7771	12	3.9335	44.711	Fshr
chr4	1.56E+08	1.56E+08	0.0069	-16.3872	12	1.5278	17.915	Agrn
chr14	32836198	32836302	0.0069248	-25.83874	11	0.90909	26.748	Fam170b
chr7	27529120	27529205	0.0069334	-30.45454	11	1.2175	31.672	Hipk4
chr9	1.19E+08	1.19E+08	6.97E-03	-33.7879	11	1.1364	34.924	Itga9
chr1	132360537	13236079[illegible]	0.0069746	-19.72234	24	4.2905	24.013	Tmcc2
chr1	1.87E+08	1.87E+08	0.00701	-51.9426	11	2.5379	54.481	Gpatch2
chr12	1.03E+08	1.03E+08	0.007027	-19.6429	11	5.4545	25.097	Itpk1
chr14	73025695	73025849	0.0070768	-41.14583	16	0.52083	41.667	Cysltr2
chr8	121551897	12155215[illegible]	0.0072987	-27.14436	16	0.625	27.769	Fbxo31 & Gm20388
chr4	1.45E+08	1.45E+08	0.007393	-23.802	28	1.8197	25.622	Dhrs3
chr7	56690776	56691404	0.007488	-48.7917	20	8.9167	57.708	

chr4	127048471	127049109	0.0076594	-27.77116	18	2.6455	30.417	Gm12940 & zinc finger, MYM domain containing 1
chr18	61206381	61207894	0.007736	-26.6261	22	6.25	32.876	Slc26a2
chr11	1.16E+08	1.16E+08	0.007787	-21.7673	18	0.9259	22.693	Unk
chr1	33681090	33681470	0.00796	-24.6848	25	8.7819	33.467	
chr2	125740809	125740940	0.0079597	-23.83157	18	0.46296	24.295	Secisbp2l
chr15	75710920	75711582	0.007997	-22.59523	15	1.3095	23.905	Rhpn1
chr9	110627492	110627755	0.0080103	-20.38690	18	1.3889	21.776	Nbeal2
chr4	1.46E+08	1.46E+08	0.00804	-22.7636	18	0.6944	23.458	Gm13226
chr2	181236058	181236415	0.0080479	-19.70189	23	2.6475	22.349	Helz2
chr11	67355948	67356088	0.008051	-44.375	20	1	45.375	Myh13
chr2	1.75E+08	1.75E+08	0.008051	-39.7244	18	1.8519	41.576	
chr17	25937051	25937415	0.008051	-24.2293	18	5.2745	29.504	Pigq & Gm26694
chr11	109558764	109559603	0.0080506	-22.83439	18	0.69444	23.529	Arsg
chr7	46544402	46544808	0.008051	-22.8042	18	0.9259	23.73	Sergef
chr5	113753221	113753705	0.0080506	-20.50721	18	1.5046	22.012	Sart3
chr1	1.36E+08	1.36E+08	0.008062	-18.9291	23	10.87	29.799	Inava
chr11	69998394	69998737	0.0080677	-25.78042	18	0.92593	26.706	Phf23
chr9	20928735	20929658	0.008526	-25.2556	17	3.2633	28.519	Dnmt1
chr2	103691802	103692493	0.0093986	-32.08916	17	0.74697	32.836	Abtb2
chr9	22129936	22130226	0.0095946	-23.27964	17	1.9608	25.24	6530413G14Rik & Acp5
chr1	1.67E+08	1.67E+08	0.009618	-31.2605	17	1.8908	33.151	Fam78b
chr2	32303651	32303839	0.0096481	-23.69056	21	1.4021	25.093	Golga2
chr5	35129262	35129517	0.009762	-22.8792	24	2.1712	25.05	
chr7	133020081	133020573	0.0097805	-29.32773	17	0.58824	29.916	Ctbp2 & Fgfr2
chr17	46685623	46686054	0.00989	-30.7384	17	3.9706	34.709	Ppp2r5d & Gm26904
chr16	11029430	11030090	0.0099047	-27.45639	17	2.556	30.012	Litaf
chr4	1.3E+08	1.3E+08	0.009937	-21.7489	21	2.3413	24.09	Col16a1
chr19	43663691	43664197	0.010015	-37.2024	14	1.9048	39.107	Slc25a28
chr2	29730581	29730887	0.010015	-27.7991	19	2.7471	30.546	Rapgef1
chr6	48975493	48976334	0.010015	-20.1853	21	6.1508	26.336	Doxl2
chr2	173033589	173033813	0.010015	-15.20647	17	4.1575	19.364	Rbm38 & Gm14453
chr4	99986684	99986868	0.010017	-34.91496	14	3.4864	38.401	Pgm1
chr10	19818653	19819034	0.010017	-30.3979	19	2.4635	32.861	
chr2	167978203	167978403	0.010017	-24.64466	14	2.7056	27.35	Ptpn1
chr11	35888799	35889378	0.010017	-23.76700	21	3.5544	27.321	Wwc1
chr12	76564317	76564540	0.010017	-21.50178	19	3.3835	24.885	Plekhg3
chr17	15401717	15402090	0.01003	-33.64229	21	7.1825	40.825	Fam120b
chr11	120009788	120010031	0.01003	-18.85822	21	3.7103	22.569	Aatk
chr7	46285125	46285466	0.010087	-31.53508	19	4.8246	36.36	Otog
chr11	65187444	65187531	0.010087	-26.24578	17	5.4669	31.713	Myocd
chr15	76454060	76454200	0.010087	-23.1271	17	4.0966	27.224	Bop1
chr8	1.07E+08	1.07E+08	0.010087	-20.3245	17	1.4706	21.795	
chr16	34054688	34055075	0.010338	-32.52377	19	3.1738	35.698	Kalrn
chr7	68165016	68165289	0.010459	-13.12876	29	2.5287	15.657	Igf1r
chr16	25018505	25018662	0.010817	-51.1992	16	20.312	71.512	
chr5	125052518	125052979	0.01092	-24.12408	13	1.0029	25.127	Ncor2
chr6	84107315	84107618	0.010952	-36.48663	13	2.1902	38.677	Dysf
chr11	118065658	118065911	0.011034	-27.71329	16	6.9792	34.692	Dnah17
chr6	115980666	115980925	0.011284	-19.37500	16	1.25	20.625	Plxnd1
chr8	71717171	71717272	0.011462	-21.23321	13	3.3333	24.567	Fcho1
chr15	99243343	99243402	0.01152	-29.71153	13	0.64103	30.353	Mcrs1
chr14	20712237	20712576	0.011798	-17.22222	18	0.90278	18.125	Zswim8

chr12	107917090	107917242	0.011801	-22.95544	22	1.8669	24.822	Bcl11b
chr8	80771107	80771615	0.011813	-27.73809	18	3.0093	30.747	Gm7997 & Gab1
chr4	136007717	136007847	0.011881	-46.71875	16	1.4062	48.125	Eloa & Gm13008
chr8	1.1E+08	1.1E+08	0.011881	-18.5392	22	7.4838	26.023	Hydin
chr13	51723074	51723839	0.011907	-24.5313	16	0.4464	24.978	Sema4d
chr2	30256020	30256123	0.011928	-35.96974	16	3.0729	39.043	Lrrc8a &
chr4	141871730	141872021	0.012111	-20.06200	16	1.0417	21.104	Efhd2
chr5	1.4E+08	1.4E+08	0.012128	-35.2046	16	1.4621	36.667	Micall2
chr3	1.42E+08	1.42E+08	0.01214	-58.6111	12	1.3889	60	Bmpr1b
chr7	98092513	98092679	0.012184	-24.41592	16	1.6667	26.083	Myo7a
chr11	6676510	6677015	0.012189	-30.65152	16	3.8802	34.532	Ramp3
chr2	1.06E+08	1.06E+08	0.012254	-39.375	16	0.7813	40.156	Pax6os1
chr7	111747478	111748530	0.012254	-29.53373	16	0.78125	30.315	Galnt18
chr2	168668936	168669518	0.012254	-29.17162	16	2.691	31.863	Atp9a
chr2	169720658	169721091	0.012254	-28.37651	16	1.4052	29.782	Tshz2 & Gm26883
chr8	1.2E+08	1.2E+08	0.012254	-28.3382	16	2.3935	30.732	Adad2
chr11	1.01E+08	1.01E+08	0.012254	-21.8447	20	5.5016	27.346	Kat2a
chr12	1.09E+08	1.09E+08	0.012254	-17.4869	16	0.3472	17.834	
chr7	85285355	85285500	0.012259	-36.7336	16	2.3438	39.077	
chr15	83114761	83114992	0.012259	-34.21255	16	5.0087	39.221	Rrp7a & Serhl
chr17	66432663	66433423	0.012261	-30.94678	20	3.125	34.072	Mtcl1
chr4	1.54E+08	1.54E+08	0.012261	-26.5983	18	1.3845	27.983	Wrap73
chr4	132127358	132127586	0.012261	-21.90972	16	1.6667	23.576	Oprd1
chr15	27748242	27748359	0.012287	-31.131	12	5.625	36.756	Trio
chr15	76012131	76012334	0.012342	-30.41140	12	3.287	33.698	Fam83h & K230010J24Rik
chr7	6440142	6440204	0.012368	-21.8634	12	0.9259	22.789	Olfr1344 & Gm20715
chr7	7288895	7289397	0.012369	-19.8468	20	4.8933	24.74	Clcn4
chr10	61657243	61657992	0.012468	-60.69444	15	5.8056	66.5	Ppa1
chr4	94996762	94996937	0.012468	-60.20238	10	8.75	68.952	Gm12694
chr11	79191619	79192100	0.012468	-59.0595	10	2.9643	62.024	
chr15	100182461	100182596	0.012468	-54.33605	10	2.5806	56.917	Dip2b
chr7	49453152	49453430	0.012468	-54.23611	10	9.1667	63.403	Nav2
chr19	57639951	57640253	0.012468	-52.58333	10	5.8333	58.417	Atrnl1
chr19	24351413	24351734	0.012468	-52.50595	10	1.3333	53.839	Pip5k1b
chr4	120133517	120133677	0.012468	-51.90488	10	4.8451	56.75	Hivep3
chr3	91607029	91607209	0.012468	-51.25	10	14.583	65.833	
chr4	60183221	60183454	0.012468	-49.7579	15	24.032	73.79	
chr5	124794378	124794430	0.012468	-46.91666	10	1.6667	48.583	Dnah10
chr8	75001401	75001459	0.012468	-45.01587	10	0.71429	45.73	Hmgxb4
chr3	14098242	14098814	0.012468	-44.43091	10	2.5	46.931	Ralyl
chr2	166077531	166077631	0.012468	-43.91269	10	2	45.913	Sulf2
chr4	1.35E+08	1.35E+08	0.012468	-43.7864	18	8.6574	52.444	
chr12	16730165	16730495	0.012468	-43.4524	10	3.2143	46.667	Greb1
chr12	91333967	91334056	0.012468	-42.58712	10	0.45455	43.042	Cep128
chr16	62317343	62317458	0.012468	-42.0982	12	3.6111	45.709	
chr5	112744491	112744699	0.012468	-40.54166	10	1.875	42.417	Myo18b
chr11	87871443	87871525	0.012468	-40.16666	10	2.6667	42.833	Epx
chr2	165796157	165796240	0.012468	-39.72222	10	2.625	42.347	Zmynd8
chr13	98239027	98239474	0.012468	-39.5417	10	2.875	42.417	
chr4	125698249	125698470	0.012468	-39.27381	10	6.25	45.524	Grik3
chr17	65548009	65548170	0.012468	-37.75	10	1.6667	39.417	
chr1	180403373	180403682	0.012468	-37.24206	10	1.0556	38.298	Itpkb
chr13	111756133	111756239	0.012468	-37.21825	10	2.0556	39.274	Map3k1
chr10	93846081	93846178	0.012468	-37.09505	10	0.95455	38.05	Usp44
chr14	72441256	72441514	0.012468	-36.84325	10	2.5	39.343	Gm9195
chr4	84275935	84276135	0.012468	-36.75000	10	1.6667	38.417	Bnc2

chr7	144508144	144508216	0.012468	-36.73611	12	10.417	47.153	Ppfia1
chr11	116973356	116973475	0.012468	-36.14352	10	1.7143	37.858	Mgat5b
chr10	81308485	81308635	0.012468	-35.7401	10	9.875	45.615	Pip5k1c
chr4	139444528	139444671	0.012468	-35.40476	10	0.71429	36.119	Ubr4
chr6	1.14E+08	1.14E+08	0.012468	-35.0813	10	0.7143	35.796	
chrX	1.6E+08	1.6E+08	0.012468	-34.8333	10	4.0833	38.917	
chr15	82005451	82005827	0.012468	-34.79166	10	3	37.792	Xrcc6 & Desi1
chr2	32677898	32678429	0.012468	-34.74206	18	2.3413	37.083	Eng
chr15	99626443	99626540	0.012468	-34.59490	12	5.0463	39.641	Racgap1
chr18	64505174	64505294	0.012468	-34.36904	10	5.5833	39.952	Nars
chr2	118696631	118696739	0.012468	-34.30952	10	2.381	36.69	Pak6
chr15	89100657	89100903	0.012468	-33.47222	15	1.5595	35.032	Tubgcp6
chr7	67669631	67669831	0.012468	-32.9524	10	5	37.952	Ttc23
chr5	32143811	32144155	0.012468	-32.67361	12	8.8542	41.528	Fosl2
chr4	129992669	129992828	0.012468	-32.18055	10	3.6667	35.847	Adgrb2
chr8	1.18E+08	1.18E+08	0.012468	-31.5987	20	8.1483	39.747	Mphosph6
chr6	15527568	15527759	0.012468	-31.5913	10	5.3452	36.937	
chr4	1.32E+08	1.32E+08	0.012468	-31.3095	10	1.6667	32.976	
chr2	1.04E+08	1.04E+08	0.012468	-31.2626	10	1.0714	32.334	D430041D05Rik
chr13	60667743	60668353	0.012468	-30.84505	12	3.3333	34.178	Dapk1
chr2	1.19E+08	1.19E+08	0.012468	-30.6944	10	2.5	33.194	Ankrd63
chr7	16134113	16134288	0.012468	-30.6131	10	4.9702	35.583	Slc8a2
chr1	135847795	135847950	0.012468	-30.56222	10	3.7679	34.33	Tnnt2
chr1	36520009	36520107	0.012468	-29.75	10	10.583	40.333	Cnnm3
chr13	30827839	30828301	0.012468	-29.62500	10	1.6667	31.292	Exoc2
chr8	1.24E+08	1.24E+08	0.012468	-29.0833	10	2.4286	31.512	
chr17	83795268	83795365	0.012468	-29.03846	26	1.2821	30.321	Mta3
chr9	18427337	18427535	0.012468	-28.9286	10	3.5	32.429	
chr5	105479987	105480120	0.012468	-28.81710	10	7.6234	36.44	Lrrc8b
chr15	97869584	97869679	0.012468	-28.76426	11	9.0929	37.857	Vdr
chr7	132788913	132789066	0.012468	-28.55158	10	2.6667	31.218	Fam53b & Fgfr2
chr4	1.55E+08	1.55E+08	0.012468	-28.3623	12	2.2321	30.594	Ski
chr17	56396511	56396917	0.012468	-28.01713	20	5.0487	33.066	Kdm4b
chr4	125055141	125055340	0.012468	-27.87301	10	0.71429	28.587	Dnali1 & Gnl2
chr7	12971414	12971464	0.012468	-27.52420	12	4.5718	32.096	Zfp324
chr5	120553676	120553909	0.012468	-27.36488	11	0.75758	28.122	Tpcn1
chr9	1.09E+08	1.09E+08	0.012468	-27.3552	10	2.4583	29.813	Atrip
chr17	56421952	56422239	0.012468	-27.0618	15	4.4726	31.534	Ptprs
chr11	116037322	116037931	0.012468	-26.99062	10	2.6667	29.657	Unk
chr2	29650907	29651466	0.012468	-26.87301	10	0.55556	27.429	Rapgef1
chr1	1.81E+08	1.81E+08	0.012468	-26.8611	10	1.6667	28.528	
chr7	1.41E+08	1.41E+08	0.012468	-26.6984	10	1.9643	28.663	Pkp3
chr17	32133419	32133562	0.012468	-26.5642	23	2.4094	28.974	Gm17276 & Notch3
chr7	44899866	44900201	0.012468	-26.54777	10	1.1806	27.728	Fuz
chr12	7977337	7977699	0.012468	-26.02381	15	2.2619	28.286	Apob
chr13	55967408	55968183	0.012468	-25.7402	23	1.8116	27.552	
chr1	90275454	90276062	0.012468	-25.631	10	1.6667	27.298	
chr19	5869512	5869623	0.012468	-24.7976	10	0.625	25.423	Frmd8
chr17	29339397	29339970	0.012468	-24.32181	23	0.36232	24.684	Mtch1
chr19	23975507	23975574	0.012468	-23.92124	10	2.4359	26.357	Fam189a2
chr19	6577441	6577956	0.012468	-23.5948	10	5.1774	28.772	
chr5	1.41E+08	1.41E+08	0.012468	-23.5819	10	5.7143	29.296	Ttyh3
chr5	37104663	37104819	0.012468	-23.44282	10	2	25.443	Jakmip1 & Gm1043
chr4	148886632	148887011	0.012468	-22.89881	10	0.5	23.399	Casz1
chr5	30593462	30593816	0.012468	-21.2719	20	1.25	22.522	Kcnk3
chr17	64137557	64138239	0.012468	-21.24084	26	9.2949	30.536	Fer

chr3	1.21E+08	1.21E+08	0.012468	-21.0532	12	0.463	21.516	Tmem56
chr9	106237980	106238207	0.012468	-20.82720	10	6.4585	27.286	Alas1
chr4	148051994	148052367	0.012468	-20.21031	10	6.5833	26.794	Mthfr
chr15	76059285	76059575	0.012468	-19.23214	10	3.3333	22.565	Scrib
chr11	103799816	103800014	0.012468	-18.82280	10	2.0556	20.878	Wnt3
chr7	44268329	44268409	0.012468	-18.78445	10	3	21.784	Gm18905
chr2	119293747	119293845	0.012468	-17.85218	12	2.3282	20.18	Vps18
chr5	124121392	124121535	0.012468	-17.75649	26	3.0357	20.792	Pitpnm2
chr1	85906439	85906563	0.012468	-17.3373	10	1.5556	18.893	Itm2c
chr11	1.03E+08	1.03E+08	0.012468	-15.8304	26	1.6026	17.433	Plcd3
chr14	52006572	52006769	0.012468	-13.87509	20	6.4444	20.32	Zfp219
chr16	33158019	33158472	0.012517	-31.6779	23	6.2319	37.91	
chr2	1.65E+08	1.65E+08	0.012547	-27.9862	15	8.3333	36.32	Elmo2
chr2	1.05E+08	1.05E+08	0.012547	-23.2685	15	1.6667	24.935	
chr6	125599894	125600116	0.012701	-23.05158	15	1.9167	24.968	Vwf
chr19	43795712	43796286	0.012814	-29.9246	15	2.2222	32.147	Abcc2
chr4	134906266	134906721	0.012848	-48.02777	15	5	53.028	Tmem50a
chr2	17722831	17723553	0.012848	-38.29365	15	3.9444	42.238	Nebl
chr5	102040156	102040348	0.012848	-23.66426	15	1.6667	25.331	Wdfy3
chr10	75352677	75352785	0.013078	-40.40476	15	2.2222	42.627	Gm5779 & Gucd1
chr13	99228898	99229041	0.013078	-15.91783	15	3.8014	19.719	Zfp366
chr18	52796483	52797363	0.013173	-29.67460	15	1.9444	31.619	Sncaip
chr8	70770809	70771132	0.013173	-22.6349	15	5.8333	28.468	Pik3r2
chr1	1.8E+08	1.8E+08	0.013189	-20.9352	15	0.6667	21.602	Stum
chr11	1.21E+08	1.21E+08	0.013236	-35.3333	15	1.6667	37	Gcgr
chr17	46070927	46071454	0.013236	-34.97474	15	2.5253	37.5	AC110562.2
chr8	119601583	119601661	0.013236	-30.61111	15	1.0833	31.694	Taf1c
chr11	79045015	79045285	0.013236	-26.96222	21	3.514	30.476	Ksr1
chr1	1.83E+08	1.83E+08	0.013237	-20.5257	15	3.3333	23.859	Disp1
chr5	111183053	111183140	0.013242	-20.26972	15	0.55556	20.825	Ttc28
chr7	28023154	28023620	0.013267	-49.3056	15	15.833	65.139	
chr17	75737885	75738412	0.013267	-27.8059	15	1.9444	29.75	
chr7	35612411	35612680	0.013267	-20.88912	15	3.4444	24.334	Ankrd27
chr2	1.59E+08	1.59E+08	0.013267	-18.8333	15	2.7778	21.611	
chr8	8711586	8711682	0.013481	-27.10084	17	3.5784	30.679	Gm18393
chr7	44884548	44884886	0.013483	-49.44727	14	3.1888	52.636	Med25
chr4	1.36E+08	1.36E+08	0.01401	-29.8479	24	7.662	37.51	
chr15	81783073	81783308	0.01401	-18.4215	19	4.1145	22.536	Zc3h7b
chr8	123407927	123408023	0.014077	-25.08053	17	1.7157	26.796	Mc1r & Gm20388
chr3	121354323	121354962	0.014155	-26.86716	19	0.96491	27.832	Alg14
chr6	86085957	86086431	0.014254	-28.3473	17	4.3417	32.689	Gm44089 & adducin 2 (beta)
chr16	35651568	35651669	0.014299	-20.74859	19	1.8203	22.569	Sema5b
chr17	34718663	34719234	0.01433	-18.2784	35	2.424	20.702	Tnxb
chr8	122747338	122747587	0.014363	-27.52976	14	2.0833	29.613	C230057M02Rik & Gm20388
chr11	69650268	69650780	0.014478	-39.80310	19	1.7544	41.557	Fxr2
chr8	83934397	83934792	0.014504	-28.46938	14	4.3155	32.785	Adgrl1 & Gm10644
chr7	24912751	24913490	0.01454	-28.76096	19	3.7385	32.499	Arhgef1
chr5	36909560	36909770	0.01454	-18.22896	19	2.0175	20.247	Ppp2r2c
chr10	43576338	43576699	0.014562	-32.675	19	0.8772	33.552	
chr15	94324456	94324846	0.014562	-29.78075	17	10.839	40.62	Adamts20
chr4	62498911	62499418	0.014562	-19.0764	19	4.7382	23.815	Hdhd3
chr10	77937723	77937867	0.014657	-31.5056	17	5.5182	37.024	Trpm2
chr17	48786326	48786453	0.014663	-30.2089	17	3.0042	33.213	
chr7	24994592	24994716	0.014663	-25.77066	17	4.7335	30.504	Atp1a3
chr10	59894334	59894406	0.014674	-28.5598	17	10.131	38.69	Dnajb12

chr9	21832031	21832650	0.014754	-38.4792	17	3.9216	42.401	Dock6
chr7	35658186	35658703	0.014777	-22.3016	14	1.3039	23.605	
chr9	113981405	113981696	0.014786	-26.41812	19	3.1433	29.561	Fbxl2
chr7	112353888	112354117	0.014786	-20.91184	19	0.87719	21.789	Mical2
chr2	1.58E+08	1.58E+08	0.014786	-20.1422	19	8.4503	28.593	Gm20412 & transglutaminase 2, C polypeptide & regulation of nuclear pre-mRNA domain containing 1B
chr16	18223665	18224286	0.014786	-19.32036	19	6.5205	25.841	Zdhhc8
chr1	89624674	89625170	0.015207	-22.79942	14	0.99206	23.791	Agap1
chr4	56856695	56857191	0.015305	-34.91431	14	3.5714	38.486	Ctnnal1
chr3	97591430	97592269	0.015348	-43.86904	14	0.89286	44.762	Chd1l
chr2	4891692	4891799	0.015355	-40.77381	14	3.5714	44.345	Sephs1
chr13	52757027	52757704	0.015355	-39.1667	14	4.5833	43.75	BB123696
chr16	31892513	31892632	0.015355	-36.59863	14	5.4762	42.075	Meltf
chr6	122696063	122696202	0.015355	-17.32142	14	1.7857	19.107	Gm10420
chr12	33249224	33249311	0.015361	-30.2126	14	1.7857	31.998	Atxn7l1
chr7	45817874	45818209	0.015361	-28.35884	14	3.5714	31.93	Kcnj14
chr4	1.37E+08	1.37E+08	0.015361	-26.0649	14	0.8929	26.958	
chr7	30584067	30584523	0.015361	-23.72449	14	3.5714	27.296	Kmt2b
chr17	28857384	28857656	0.015367	-22.93650	14	2.4235	25.36	Pnpla1os
chr15	102188210	102188359	0.01537	-17.68617	14	0.71429	18.4	Csad
chr7	135269481	135270076	0.015431	-44.70238	14	1.7857	46.488	Nps
chr2	140073375	140073832	0.015431	-19.49263	14	0.59524	20.088	Rps19-ps7
chr18	65991550	65991851	0.015434	-33.47222	14	2.7126	36.185	Lman1
chr3	1.33E+08	1.33E+08	0.015456	-35.7924	14	1.4286	37.221	Tet2
chr12	112600026	112600102	0.015488	-31.17205	14	0.5102	31.682	Inf2
chr18	36028601	36028824	0.015488	-25.51767	14	1.9048	27.422	Nrg2
chr17	25719195	25719375	0.015492	-34.07467	14	1.1905	35.265	Gng13 & Chtf18
chr7	126160487	126160965	0.015502	-36.32227	14	2.619	38.941	Xpo6
chr11	28339621	28339867	0.015563	-55.4762	14	8.8095	64.286	Gm6685
chr8	95842385	95842873	0.015576	-23.79960	14	1.1905	24.99	Slc38a7
chr1	34200903	34201148	0.015624	-27.69444	25	4.6548	32.349	Dst
chr17	14680962	14681412	0.016149	-28.8766	22	3.1025	31.979	Thbs2
chr11	61384138	61384615	0.0167	-28.2051	13	1.0989	29.304	
chr11	57709535	57709783	0.016706	-25.76007	13	2.8388	28.599	Galnt10
chr15	74370013	74370465	0.01676	-25.3938	13	2.8846	28.278	
chr15	76174498	76174593	0.016865	-31.22767	16	1.9531	33.181	Plec
chr10	67571500	67571610	0.017171	-45.641	13	1.9231	47.564	4930563J15Rik
chr15	83867038	83867208	0.01737	-32.1474	13	1.2821	33.429	Efcab6
chr1	43553923	43554145	0.017383	-21.73000	26	1.3462	23.076	Nck2
chr6	83299335	83299961	0.017393	-25.4711	27	1.7996	27.271	Slc4a5
chr10	76436508	76436623	0.017411	-14.56101	16	0.39062	14.952	Pcnt
chr2	150815318	150815687	0.017477	-32.88461	13	1.9231	34.808	Pygb
chr16	93754117	93754619	0.017477	-25.89	20	2.2778	28.168	Dop1b
chr14	54664389	54664529	0.017563	-44.08068	18	0.69444	44.775	Acin1
chr13	48928436	48928825	0.017565	-52.2329	13	2.0604	54.293	Fam120a
chr11	99348872	99348980	0.017605	-47.96703	13	3.8462	51.813	Krt27
chr6	34755641	34755881	0.017605	-23.7197	16	5.625	29.345	Cald1
chr15	81851410	81851629	0.017605	-20.76637	13	3.5343	24.301	Tob2
chr6	29456324	29456376	0.017605	-20.3939	13	2.9487	23.343	Flnc
chr12	73351791	73351867	0.017608	-48.30586	13	1.2821	49.588	Slc38a6
chr9	99067661	99068084	0.017608	-44.70696	13	1.9231	46.63	Pik3cb
chr2	31598430	31598726	0.017608	-44.09340	13	2.8846	46.978	Fubp3
chr8	125238171	125238986	0.017608	-41.70787	13	0.76923	42.477	Disc1

chr10	68000221	68000412	0.017608	-37.50915	13	1.7308	39.24	Rtkn2
chr5	30893006	30893107	0.017608	-36.67665	13	5.2506	41.927	Agbl5
chr17	35141519	35141895	0.017608	-33.92747	16	4.367	38.294	Bag6
chr10	38918657	38919392	0.017608	-32.6978	18	6.9444	39.642	
chr11	87771357	87771768	0.017608	-32.44352	13	0.76923	33.213	Tspoap1
chr5	121426139	12142691(	0.017608	-30.63864	16	3.4375	34.076	Naa25
chr6	5423649	5423848	0.017608	-30.21062	13	1.2821	31.493	Asb4
chr10	117215180	11721557	0.017608	-30.12820	13	0.96154	31.09	Yeats4
chr15	1.02E+08	1.02E+08	0.017608	-28.9713	13	0.9615	29.933	Krt1
chr4	142104779	14210490(	0.017608	-28.27075	13	1.8681	30.139	Kazn
chrX	1.02E+08	1.02E+08	0.017608	-28.1512	13	0.9615	29.113	
chr19	6504939	6505009	0.017608	-27.92124	13	8.4066	36.328	Nrxn2
chr2	31800232	31800359	0.017608	-27.79068	13	2.3077	30.098	Abl1
chr18	4672924	4673968	0.017608	-27.74206	20	5.5278	33.27	Jcad
chr15	36491420	36491685	0.017608	-26.859	13	3.4615	30.321	Ankrd46
chr12	91675176	91675400	0.017608	-26.0043	13	1.3355	27.34	Gm8378 & Ston2
chr5	144877877	14487834	0.017608	-25.90201	13	3.8462	29.748	Smurf1
chr5	74959230	74959614	0.017608	-24.93589	13	1.2821	26.218	Gm6116
chr11	97695544	97695581	0.017608	-24.65812	13	3.8462	28.504	Pcgf2
chr15	84288276	84288827	0.017608	-24.45054	13	0.6993	25.15	Parvb
chr15	11387655	11387810	0.017608	-24.20787	13	1.9231	26.131	Tars
chr4	1.29E+08	1.29E+08	0.017608	-23.931	18	1.702	25.633	A3galt2
chr11	53535776	53535929	0.017608	-23.50274	13	2.5641	26.067	Sept8
chr5	112290120	11229060	0.017608	-23.43025	13	0.96154	24.392	Tpst2
chr8	3458863	3459644	0.017608	-23.05860	13	3.2051	26.264	Pex11g & Gm44775
chr8	1.24E+08	1.24E+08	0.017608	-22.9088	13	3.9044	26.813	Gm20388
chr4	141474570	14147472	0.017608	-22.81746	13	2.5641	25.382	Spen
chr14	25746241	25746773	0.017608	-22.27075	13	1.511	23.782	Zcchc24
chr8	1.2E+08	1.2E+08	0.017608	-22.1245	13	0.8547	22.979	Gm20388
chr9	121470974	12147155(	0.017608	-21.44230	13	0.96154	22.404	Trak1
chr19	4108767	4109119	0.017608	-21.0943	13	2.381	23.475	Pitpnm1
chr2	1.57E+08	1.57E+08	0.017608	-20.0366	13	0.641	20.678	Epb41l1
chr4	156217488	15621758	0.017608	-18.76526	13	0.76923	19.534	Perm1
chr14	55098978	55099226	0.017608	-18.70659	16	1.4757	20.182	Thtpa & Gm20687 & Ap1g2
chr7	24922872	24923006	0.017608	-18.24505	16	0.97222	19.217	Arhgef1
chr2	167105590	16710572(	0.017608	-17.68467	13	1.2821	18.967	Kcnb1
chr19	6320358	6320447	0.017608	-16.5526	13	2.0513	18.604	Cdc42bpg
chr7	73310612	73310925	0.017608	-16.1645	13	0.9615	17.126	4930429H19Rik
chr15	96733105	96734194	0.017648	-20.9067	23	5.3442	26.251	
chr4	11491900	11492622	0.017708	-27.48536	18	1.7361	29.221	Virma
chr7	1.27E+08	1.27E+08	0.017709	-35.2778	18	4.8611	40.139	
chr13	47471727	47472074	0.017709	-24.9074	18	3.287	28.194	
chr4	1.23E+08	1.23E+08	0.017719	-41.5278	18	2.7778	44.306	
chr1	91353906	91354044	0.017763	-16.95071	23	7.9296	24.88	Klhl30
chr8	121357855	12135845(	0.017837	-34.32429	18	6.4815	40.806	Gm20388
chr8	94889284	94889530	0.017837	-32.6918	18	3.502	36.194	
chr5	73403388	73403671	0.01791	-30.1499	18	3.2407	33.391	
chr2	1.3E+08	1.3E+08	0.01792	-66.9444	10	15.556	82.5	Ebf4
chr1	82496968	82497220	0.01792	-66.38888	15	4.7222	71.111	Col4a4
chr7	10471936	10472054	0.01792	-63.25	10	12.167	75.417	
chr13	25036538	25036638	0.01792	-62.0417	10	10.542	72.583	
chr6	86274608	86274795	0.01792	-62.00000	10	4.1667	66.167	Tgfa
chr4	140132551	14013317(	0.01792	-58.13317	10	5.3248	63.458	Gm13027 & immunoglobulin superfamily, member 21

chr16	38028393	38028568	0.01792	-57.119	10	1.5476	58.667	
chr11	51992117	51992350	0.01792	-56.8869	10	8.6667	65.554	Ube2b & Gm26551
chr2	163740267	163740533	0.01792	-56.54761	10	5	61.548	Ada
chr1	1.9E+08	1.9E+08	0.01792	-56.5	10	3.5	60	Cenpf
chr2	76919411	76919763	0.01792	-56.25	10	4.0833	60.333	Ttn
chr8	22625863	22626305	0.01792	-55.78571	10	1.7143	57.5	Dkk4
chr3	51252804	51253152	0.01792	-54.50000	10	6.8333	61.333	Elf2
chr13	43782780	43783081	0.01792	-54.5	10	2.5	57	
chr8	1.04E+08	1.04E+08	0.01792	-54.1667	10	30.833	85	
chr8	77382363	77382741	0.01792	-54.05555	15	1.5	55.556	Arhgap10
chr1	89408314	89408606	0.01792	-53.5	10	4.1667	57.667	
chr15	1.02E+08	1.02E+08	0.01792	-53.4726	10	4.3056	57.778	
chr13	85103287	85103368	0.01792	-53.4028	12	10.417	63.819	
chr7	24709020	24709084	0.01792	-53.2222	15	10.111	63.333	
chr4	140576648	140577122	0.01792	-53.06349	10	2.0833	55.147	Arhgef10l
chr12	104688366	104688597	0.01792	-52.83333	10	5	57.833	Dicer1
chr7	30721485	30721705	0.01792	-52.19444	10	4.5833	56.778	Atp4a
chr8	26386507	26387247	0.01792	-51.1548	10	1.4286	52.583	
chr8	13930710	13930822	0.01792	-51.10912	10	3.5417	54.651	Fbxo25
chr1	1.77E+08	1.77E+08	0.01792	-51	10	6.6667	57.667	
chr10	40570346	40570483	0.01792	-50.9796	10	5.4192	56.399	Slc22a16
chr15	82010590	82010958	0.01792	-50.58333	10	16.667	67.25	Xrcc6 & Desi1
chr10	75105956	75106512	0.01792	-50.25000	10	1.25	51.5	Bcr
chr12	72994817	72994885	0.01792	-50.1984	12	6.0516	56.25	
chr4	151753020	151753351	0.01792	-50.05952	10	5	55.06	Camta1
chr5	122940755	122940973	0.01792	-49.68254	10	4.0675	53.75	Kdm2b
chr2	125200794	125200981	0.01792	-49.58333	10	5	54.583	Slc12a1
chr3	116304918	116305005	0.01792	-49.30952	10	4.6905	54	Cdc14a
chr14	104487331	104487442	0.01792	-49.25378	10	8.6667	57.92	Rnf219
chr2	1.72E+08	1.72E+08	0.01792	-48.0704	12	2.0833	50.154	
chr5	1.06E+08	1.06E+08	0.01792	-47.869	10	9.631	57.5	
chr9	101923275	101923333	0.01792	-47.83333	10	7.6667	55.5	9630041A04Rik & Ephb1
chr11	74749315	74749550	0.01792	-47.4226	10	1	48.423	
chr12	8396289	8396407	0.01792	-47.34127	12	4.1667	51.508	Gm48075
chr18	90430443	90430613	0.01792	-47.2792	12	10.625	57.904	
chr6	83627347	83627776	0.01792	-47.2735	12	2.1875	49.461	
chr4	131909310	131909530	0.01792	-47.10317	10	2.5	49.603	Gm12992
chr8	1.21E+08	1.21E+08	0.01792	-46.5476	10	1.25	47.798	Gm20388
chr18	12660106	12660429	0.01792	-46.42261	10	6.4226	52.845	Ttc39c
chr2	135967918	135968173	0.01792	-46.41203	12	1.6667	48.079	Plcb4
chr3	87529842	87530213	0.01792	-45.9848	11	1.5152	47.5	Etv3
chr8	31315505	31315969	0.01792	-45.869	10	0.7143	46.583	
chr7	25724472	25724835	0.01792	-45.57870	12	1.6667	47.245	Hnrnpul1
chr6	24572496	24572655	0.01792	-45.50000	10	4.5	50	Asb15
chr11	116343365	116343600	0.01792	-45.40476	10	1.4286	46.833	Rnf157
chr11	61507779	61508070	0.01792	-45.14285	10	2.6667	47.81	B9d1
chr2	132850047	132850358	0.01792	-45.08928	10	2.9167	48.006	Crls1
chr11	65848302	65848624	0.01792	-45.03968	12	5.625	50.665	Dnah9
chr3	1.52E+08	1.52E+08	0.01792	-44.9405	10	1.6667	46.607	Dnajb4
chr5	26929802	26929933	0.01792	-44.84920	10	2.2222	47.071	Dpp6
chr14	75931167	75931363	0.01792	-44.35941	21	0.68027	45.04	Gtf2f2
chr19	3265512	3265635	0.01792	-44.19047	10	1	45.19	Ighmbp2
chr11	49943126	49943751	0.01792	-44.00000	10	1.6667	45.667	Rasgef1c
chr17	87573548	87574138	0.01792	-43.75	10	8.75	52.5	
chr5	77001486	77001632	0.01792	-43.6615	11	8.9991	52.661	
chr5	1.22E+08	1.22E+08	0.01792	-43.2624	10	2.44	45.702	

chr8	94086137	94086167	0.01792	-43.11309	10	9.375	52.488	Bbs2
chr4	106665938	106666205	0.01792	-42.89881	10	4.1538	47.053	Ttc4
chr17	34678682	34678945	0.01792	-42.86111	10	1.25	44.111	Tnxb
chr2	84876816	84876990	0.01792	-42.7083	10	3.5417	46.25	Rtn4rl2
chr10	13855882	13856216	0.01792	-42.62500	10	2.5	45.125	Aig1
chr3	105545910	105546048	0.01792	-42.06746	10	1.25	43.317	Kcnd3
chr17	25777482	25778026	0.01792	-41.95833	10	2	43.958	Narfl
chr15	12886992	12887303	0.01792	-41.91666	10	5	46.917	Drosha
chr3	142184601	142184728	0.01792	-41.91666	10	7.6667	49.583	Gm6059
chr3	51227548	51227793	0.01792	-41.69191	11	0.90909	42.601	Noct
chr16	95987406	95987603	0.01792	-41.68154	12	3.5069	45.188	Psmg1
chr17	48325516	48325744	0.01792	-41.5119	10	1.6667	43.179	B430306N03Rik
chr4	136770957	136770994	0.01792	-41.50000	10	3.75	45.25	Ephb2
chr7	29355749	29356271	0.01792	-41.4821	10	1.6667	43.149	Sipa1l3
chr8	11050411	11050794	0.01792	-41.4762	10	0.7143	42.19	9530052E02Rik
chr6	73087521	73087666	0.01792	-41.13095	10	1.25	42.381	Dnah6
chr3	78880162	78880291	0.01792	-40.4167	10	3.3333	43.75	
chr2	1.69E+08	1.69E+08	0.01792	-39.9825	17	6.6176	46.6	
chr5	20600542	20600945	0.01792	-39.9702	12	2.0833	42.054	Magi2
chr12	103171387	103171607	0.01792	-39.93975	10	3.4432	43.383	Unc79
chr8	47326804	47327030	0.01792	-39.67234	10	2.5	42.172	Stox2
chr4	8189320	8189605	0.01792	-39.5833	10	1.25	40.833	Car8
chr7	98711766	98712439	0.01792	-39.48214	10	4.375	43.857	Thap12
chr4	1.52E+08	1.52E+08	0.01792	-39.2738	10	6.25	45.524	Dnajc11
chr5	140360264	140360441	0.01792	-39.18055	10	2	41.181	Snx8
chr12	4797277	4797477	0.01792	-39.1667	10	5	44.167	
chr4	1.18E+08	1.18E+08	0.01792	-38.9287	17	5.4902	44.419	Slc6a9 & Gm17114
chr3	1.44E+08	1.44E+08	0.01792	-38.9286	12	1.0417	39.97	
chr2	28009626	28010195	0.01792	-38.64661	19	2.8352	41.482	Col5a1
chr11	70514485	70514524	0.01792	-38.63095	10	5	43.631	Vmo1
chr1	1.9E+08	1.9E+08	0.01792	-38.5595	10	2.5	41.06	Ptpn14
chr14	79143160	79143542	0.01792	-38.416	12	2.2222	40.638	Vwa8
chr4	133420611	133420709	0.01792	-38.31547	10	2.5	40.815	Slc9a1
chr3	55117449	55117663	0.01792	-38.14682	10	0.71429	38.861	Spg20
chr11	52478752	52479180	0.01792	-38.08928	10	0.625	38.714	Gm12209
chr5	112464594	112464878	0.01792	-37.83201	15	4.8929	42.725	Sez6l
chr4	1.48E+08	1.48E+08	0.01792	-37.6667	10	1	38.667	Clcn6
chr5	88112668	88112879	0.01792	-37.4365	15	5.3333	42.77	
chr11	53544431	53544915	0.01792	-37.3039	10	5	42.304	Sept8
chr19	4226729	4227034	0.01792	-37.1255	10	1.6667	38.792	
chr5	135386726	135386931	0.01792	-37.08333	10	1	38.083	Pom121
chr7	25664414	25664506	0.01792	-36.7478	10	3	39.748	Exosc5
chr5	113846157	113846664	0.01792	-36.55799	15	2.3333	38.891	Coro1c
chr4	33251799	33252048	0.01792	-36.52381	10	2.5	39.024	Pnrc1
chr8	70149144	70149746	0.01792	-36.51893	10	1	37.519	Mef2b
chr15	81705998	81706435	0.01792	-36.50000	10	1.25	37.75	Rangap1
chr6	91334612	91335023	0.01792	-36.5	10	2.4286	38.929	
chr17	70882915	70883266	0.01792	-36.39285	10	0.83333	37.226	Gm26510 & Gm26561
chr7	127023536	127023673	0.01792	-36.38888	12	1.0417	37.431	Maz
chr8	1.04E+08	1.04E+08	0.01792	-36.375	10	2.2917	38.667	Gm29682
chr12	17380333	17380693	0.01792	-36.33333	10	1	37.333	Nol10
chr3	57942944	57942988	0.01792	-36.3194	12	8.3333	44.653	Gm42511
chr7	19053966	19054099	0.01792	-36.25649	10	1.6667	37.923	Sympk
chr7	4666497	4666998	0.01792	-36.22041	10	1.5	37.72	Hspbp1
chr4	56743895	56744083	0.01792	-36.05357	10	4.0179	40.071	Actl7a
chr16	94768950	94769218	0.01792	-36.00595	10	0.71429	36.72	Kcnj6

chr1	88261410	88261949	0.01792	-35.98265	10	1.7143	37.697	Mroh2a
chr14	63168694	63168999	0.01792	-35.94534	10	5	40.945	Fdft1
chr2	170517958	17051832[illegible]	0.01792	-35.83333	10	2.5	38.333	Pfdn4
chr11	118272070	11827222[illegible]	0.01792	-35.35353	10	2.0556	37.409	Usp36
chrX	140432131	14043225[illegible]	0.01792	-35.25000	10	1.25	36.5	Frmpd3
chr5	25163452	25163688	0.01792	-34.9028	10	4.1667	39.069	
chr13	51750444	51750799	0.01792	-34.89087	12	3.373	38.264	Sema4d
chr17	33948484	33948649	0.01792	-34.72619	10	2.0833	36.81	Wdr46
chr5	113192790	11319287[illegible]	0.01792	-34.42956	12	1.9097	36.339	2900026A02Rik
chr4	139425253	13942578[illegible]	0.01792	-34.38095	10	5.8333	40.214	Ubr4
chrX	87838526	87838615	0.01792	-34.38095	10	4.1667	38.548	Il1rapl1
chr6	113517916	11351826[illegible]	0.01792	-34.32702	10	1.9091	36.236	Emc3
chr1	33643884	33644350	0.01792	-34.25	10	5	39.25	Prim2
chr4	45399435	45399579	0.01792	-34.25000	10	2.5	36.75	Slc25a51
chr9	120052153	12005222[illegible]	0.01792	-34.18921	12	2.9735	37.163	Cx3cr1
chr4	117595678	11759620[illegible]	0.01792	-34.16666	12	8.3333	42.5	Eri3
chr9	22014495	22014723	0.01792	-34.0952	10	2.8333	36.929	
chr17	27560839	27561045	0.01792	-34.06862	17	3.7115	37.78	Hmga1
chr1	159858197	15985865[illegible]	0.01792	-33.77451	17	0.98039	34.755	Tnr
chr1	55279301	55279695	0.01792	-33.2619	10	6.5952	39.857	Boll
chr4	104507795	10450829[illegible]	0.01792	-33.00000	10	1.25	34.25	Dab1
chr7	30023217	30023423	0.01792	-32.9206	10	2.5	35.421	Zfp568
chr2	166589566	16658962[illegible]	0.01792	-32.67023	12	1.6369	34.307	Prex1
chr4	136680869	13668107[illegible]	0.01792	-32.52381	10	2.2619	34.786	Ephb2
chrX	89488425	89488561	0.01792	-32.504	15	12.712	45.216	
chr18	23931730	23932200	0.01792	-32.5	12	2.0833	34.583	
chr17	29345292	29345617	0.01792	-32.4412	10	5.625	38.066	Mtch1
chr11	55221849	55222414	0.01792	-32.27182	12	0.83333	33.105	Slc36a1
chr4	89115333	89115539	0.01792	-32.19697	11	1.5152	33.712	Gm12602
chr4	107137579	10713763[illegible]	0.01792	-32.19444	10	3.8889	36.083	Tceanc2
chr14	65782809	65782971	0.01792	-32.1667	10	1	33.167	
chr8	117612850	11761300[illegible]	0.01792	-32.08333	10	3.6667	35.75	Plcg2
chr1	192668596	19266880[illegible]	0.01792	-32.02381	10	2	34.024	Hhat
chr15	31411229	31411438	0.01792	-32.0119	10	1.25	33.262	
chr5	130212006	13021209[illegible]	0.01792	-32.00000	10	4.1667	36.167	Rabgef1
chr1	180169286	18016947[illegible]	0.01792	-31.88461	10	2.25	34.135	Coq8a
chr10	81530791	81530958	0.01792	-31.86670	10	0.90909	32.776	Gna11
chr14	31282137	31282417	0.01792	-31.81944	10	3.2143	35.034	Dnah1 & Gm35823
chr4	1.5E+08	1.5E+08	0.01792	-31.8158	10	5	36.816	Ctnnbip1
chr4	1.31E+08	1.31E+08	0.01792	-31.7798	10	5	36.78	
chr18	35980010	35980073	0.01792	-31.50000	10	8.3333	39.833	Psd2
chr2	28208160	28208236	0.01792	-31.43055	10	0.625	32.056	Olfm1
chr9	1E+08	1E+08	0.01792	-31.3373	10	1	32.337	
chr13	1.07E+08	1.07E+08	0.01792	-31.1924	21	2.4698	33.662	Ipo11
chr11	117271630	11727182[illegible]	0.01792	-31.17460	10	2.1111	33.286	Sept9
chr8	13613096	13613265	0.01792	-31.16071	12	4.5139	35.675	Rasa3
chr2	25680656	25680905	0.01792	-31.1553	10	2.5	33.655	Lcn6
chr13	19531027	19531567	0.01792	-30.9821	10	4.1667	35.149	
chr5	142494870	14249495[illegible]	0.01792	-30.84845	10	2.7917	33.64	Radil
chr12	83612060	83612342	0.01792	-30.7262	10	2.6786	33.405	
chr8	12508936	12509253	0.01792	-30.5952	11	1.1364	31.732	
chr2	1.68E+08	1.68E+08	0.01792	-30.2798	10	10	40.28	
chr5	1.06E+08	1.06E+08	0.01792	-30.25	10	1.8333	32.083	
chr9	63815333	63815938	0.01792	-30.248	12	8.7121	38.96	
chr17	27147787	27148129	0.01792	-30.15377	12	4.1667	34.32	Ip6k3
chr8	47866556	47867173	0.01792	-30.00000	12	0.83333	30.833	Wwc2
chr17	87041605	87041856	0.01792	-29.7897	10	0.5556	30.345	

chr2	32647698	32648137	0.01792	-29.76851	12	1.3889	31.157	Eng
chr11	83864101	83864323	0.01792	-29.69246	12	5.5556	35.248	Hnf1b
chr2	174454113	17445435	0.01792	-29.69047	10	1.25	30.94	Tubb1
chr17	33935645	33935857	0.01792	-29.67322	12	0.69444	30.368	Rgl2
chr4	136441598	13644191	0.01792	-29.57539	10	2.2222	31.798	Htr1d
chr4	47536764	47537008	0.01792	-29.5337	12	2.7778	32.312	
chr7	13052728	13052943	0.01792	-29.49150	10	1.3393	30.831	Mzf1
chr11	118333541	11833368	0.01792	-29.48677	15	1.0317	30.519	Timp2 &
chr10	79860558	79860872	0.01792	-29.4643	10	2.5	31.964	Ptbp1 & Plppr3
chr18	35686686	35687187	0.01792	-29.36111	10	1.6667	31.028	Dnajc18
chr9	1.1E+08	1.1E+08	0.01792	-29.3361	12	0.6944	30.031	Ptpn23
chr11	16514387	16514497	0.01792	-29.3333	10	5.8333	35.167	
chr4	143117255	14311744	0.01792	-29.30555	10	3.7778	33.083	Prdm2
chr11	97448180	97448451	0.01792	-29.21131	12	2.5	31.711	Arhgap23
chr2	138441299	13844170	0.01792	-29.19047	10	2.5	31.69	Btbd3
chr2	34857364	34858007	0.01792	-29.05627	10	1.9091	30.965	Psmd5
chr10	67909337	67909515	0.01792	-28.96660	24	0.9375	29.904	Zfp365
chr5	146288073	14628852	0.01792	-28.93249	17	1.8382	30.771	Cdk8
chr14	54934675	54935252	0.01792	-28.85000	25	2.769	31.619	Il25
chr4	1.52E+08	1.52E+08	0.01792	-28.7143	15	1.9444	30.659	Acot7
chr13	47355992	47356251	0.01792	-28.6429	10	5.8333	34.476	
chr11	86921009	86921647	0.01792	-28.5422	10	1.8333	30.376	
chr2	104241060	10424131	0.01792	-28.37872	10	2.0856	30.464	D430041D05Rik
chr17	25248349	25248546	0.01792	-28.34722	10	1.875	30.222	Baiap3
chr8	117113948	11711417	0.01792	-28.25000	10	0.83333	29.083	Bco1
chr12	69453211	69453403	0.01792	-28.25	10	1.7143	29.964	
chr3	31224143	31224549	0.01792	-28.18181	12	6.25	34.432	Slc7a14
chr8	1.2E+08	1.2E+08	0.01792	-28.1167	12	1.875	29.992	Gse1 & Gm20388
chr11	115845446	11584565	0.01792	-28.03221	17	8.0392	36.071	Llgl2
chr12	1.11E+08	1.11E+08	0.01792	-27.9825	17	6.3725	34.355	Dync1h1
chr14	1.18E+08	1.18E+08	0.01792	-27.9643	10	2.5	30.464	Abcc4
chr16	35267198	35267342	0.01792	-27.96428	10	1	28.964	Adcy5
chr15	82372784	82373271	0.01792	-27.94913	11	7.1699	35.119	Cyp2d22
chr1	59099585	59099705	0.01792	-27.9167	10	1.6667	29.583	
chr6	113694168	11369438	0.01792	-27.85714	10	2.5	30.357	Irak2
chr11	72448745	72448940	0.01792	-27.75985	10	1.25	29.01	Mybbp1a
chr8	1.22E+08	1.22E+08	0.01792	-27.4967	12	2.2222	29.719	Fbxo31 & Gm20388
chr17	25391000	25391263	0.01792	-27.45238	10	3	30.452	Cacna1h
chrX	95426562	95426733	0.01792	-27.4504	12	1.0417	28.492	Amer1
chr9	108070616	10807075	0.01792	-27.33766	11	1.5152	28.853	Rnf123
chr7	44900617	44900838	0.01792	-27.25790	15	7.4617	34.72	Fuz & Ap2a1
chr8	122437438	12243758	0.01792	-27.19749	10	2.25	29.447	Mvd & Gm20388
chr5	24435255	24435349	0.01792	-26.8234	10	1.9643	28.788	Slc4a2
chr12	103333429	10333393	0.01792	-26.77862	28	1.2571	28.036	Asb2
chr10	77170506	77170865	0.01792	-26.71	11	2.2727	28.983	
chr2	1.79E+08	1.79E+08	0.01792	-26.6127	12	2.9514	29.564	Cdh4
chr12	110662936	11066301	0.01792	-26.59556	12	7.2735	33.869	Dync1h1
chr8	105702950	10570315	0.01792	-26.59043	19	3.0107	29.601	Pard6a
chr15	37040369	37041050	0.01792	-26.52381	10	2.5	29.024	Gm26766
chr7	1.4E+08	1.4E+08	0.01792	-26.4758	10	5.4226	31.898	Olfr524
chr7	1.27E+08	1.27E+08	0.01792	-26.2262	10	3	29.226	Coro1a
chr6	71946965	71947341	0.01792	-26.15079	12	0.46296	26.614	Polr1a
chr19	45001605	45001754	0.01792	-26.07539	10	1.6667	27.742	Sema4g
chr1	89772260	89772392	0.01792	-26.0298	10	7	33.03	Agap1
chr17	66148170	66148670	0.01792	-25.96428	10	2	27.964	Ddx11
chr15	99814901	99815110	0.01792	-25.94047	10	3.3333	29.274	Lima1
chr6	54499909	54500589	0.01792	-25.92691	17	0.98039	26.907	Wipf3

chr4	132753850	132754058	0.01792	-25.92261	10	2.5	28.423	Smpdl3b
chr8	20267667	20268044	0.01792	-25.9073	12	3.9836	29.891	6820431F20Rik
chr11	98155632	98155972	0.01792	-25.89285	10	1.25	27.143	Med1
chr10	62839366	62839731	0.01792	-25.86904	10	0.83333	26.702	Tet1
chr15	45692260	45692555	0.01792	-25.7558	10	2.7917	28.547	
chr9	22511863	22512351	0.01792	-25.67640	11	3.9394	29.616	Bbs9
chr13	51725244	51725510	0.01792	-25.6262	10	2.8571	28.483	Sema4d
chr2	130593421	130593985	0.01792	-25.43154	12	2.2222	27.654	Ubox5
chr5	113309530	113310100	0.01792	-25.34313	17	19.118	44.461	Sgsm1
chr5	112999854	113000047	0.01792	-25.23053	21	2.74	27.971	Grk3
chr11	1.13E+08	1.13E+08	0.01792	-25.1471	10	0.3846	25.532	
chr4	1.07E+08	1.07E+08	0.01792	-25.0062	10	1.25	26.256	
chr14	30559669	30559721	0.01792	-24.89826	10	0.625	25.523	Tkt
chr18	83001317	83001523	0.01792	-24.8862	15	2.9074	27.794	Zfp516
chr5	138983860	138984407	0.01792	-24.78192	10	3.9286	28.71	Pdgfa
chr19	42022910	42022999	0.01792	-24.72222	10	2.5	27.222	Ubtd1
chr9	58516863	58517249	0.01792	-24.6726	12	3.8194	28.492	
chr7	19387870	19387969	0.01792	-24.5425	17	3.8177	28.36	Ercc2 & Gm26852
chr7	64662077	64662458	0.01792	-24.52947	21	1.0714	25.601	Apba2
chr3	1.17E+08	1.17E+08	0.01792	-24.5205	12	0.5952	25.116	Mfsd14a & Gm43191
chr9	110506667	110506805	0.01792	-24.50000	10	9.6667	34.167	Kif9
chr11	106398438	106398730	0.01792	-24.48618	10	8.2995	32.786	Ern1
chr16	18795743	18795909	0.01792	-24.44480	10	4.2143	28.659	Cdc45
chr6	83439362	83439896	0.01792	-24.38	12	2.0833	26.463	Tet3
chr5	134137468	134138119	0.01792	-24.36409	11	2.2727	26.637	Castor2
chr1	1.63E+08	1.63E+08	0.01792	-24.3333	10	2.0833	26.417	
chr15	99248467	99248873	0.01792	-24.32265	15	1.0833	25.406	Mcrs1
chr9	119797284	119797616	0.01792	-24.28246	10	1	25.282	Scn11a
chr2	29667607	29668108	0.01792	-24.10714	10	3.9087	28.016	Rapgef1
chr4	150618121	150618481	0.01792	-24.06150	10	1.6667	25.728	Rere
chr5	1.18E+08	1.18E+08	0.01792	-23.9286	10	2.5	26.429	Ksr2
chr15	73765258	73765329	0.01792	-23.9167	10	3.3333	27.25	Mroh5
chr10	61985193	61986102	0.01792	-23.7738	10	8.381	32.155	
chr15	72742913	72743016	0.01792	-23.56451	10	1.8727	25.437	Trappc9
chr10	62950935	62951406	0.01792	-23.47718	12	1.3889	24.866	Dna2
chr2	1.67E+08	1.67E+08	0.01792	-23.2996	10	4.6667	27.966	Prex1
chr8	46111740	46112184	0.01792	-23.05224	12	2.9167	25.969	Snx25
chr17	24679689	24679919	1.79E-02	-22.9708	10	3.381	26.352	Zfp598
chr15	76181046	76181164	0.01792	-22.8258	10	3.6971	26.523	Plec
chr17	32333543	32333799	0.01792	-22.80952	10	1	23.81	Akap8l
chr8	36584134	36584273	0.01792	-22.75649	10	6.5476	29.304	Dlc1
chr7	1.45E+08	1.45E+08	0.01792	-22.7343	10	1.9643	24.699	
chr1	1.89E+08	1.89E+08	0.01792	-22.7341	10	0.8333	23.567	
chr10	76503956	76504439	0.01792	-22.6578	12	1.0417	23.699	Mcm3ap
chr14	54961056	54961209	0.01792	-22.62770	10	1.1111	23.739	Myh6
chr11	113810688	113810917	0.01792	-22.48015	12	4.375	26.855	Sdk2
chr9	8751494	8752069	0.01792	-22.4405	10	10	32.44	
chr2	166625109	166625356	0.01792	-22.42296	17	8.8796	31.303	Prex1
chr7	65072367	65073124	0.01792	-22.38678	17	1.4706	23.857	Fam189a1
chr4	125540871	125540966	0.01792	-22.35539	10	3.2143	25.57	Grik3
chr18	64386353	64386555	0.01792	-22.13056	12	0.83333	22.964	Onecut2
chr11	75621012	75621247	0.01792	-21.95271	12	1.3889	23.342	Pitpna
chr4	150616899	150617044	0.01792	-21.92460	15	9.1616	31.086	Rere
chr7	75757417	75757646	0.01792	-21.9167	10	12.5	34.417	
chrX	36415162	36415541	0.01792	-21.8601	12	2.4802	24.34	
chr2	156043137	156043374	0.01792	-21.74007	10	1.875	23.615	Fer1l4
chrX	73421810	73421968	0.01792	-21.7083	10	0.8333	22.542	Zfp92

chr5	31070315	31070376	0.01792	-21.59920	10	1.3889	22.988	Cad
chr16	32777537	32777718	0.01792	-21.5972	10	2.5	24.097	Muc20 & Muc4
chr11	68783152	68783247	0.01792	-21.55591	10	0.55556	22.111	Myh10
chr9	43312437	43312767	0.01792	-21.53210	10	1.25	22.782	Trim29
chr11	72977650	72978358	0.01792	-21.49626	17	0.88235	22.379	Atp2a3
chr4	1.52E+08	1.52E+08	0.01792	-21.317	17	5.6919	27.009	Zbtb48
chr12	1.13E+08	1.13E+08	0.01792	-21.1583	11	1.1364	22.295	
chr5	112475221	11247532	0.01792	-21.13940	10	1.6667	22.806	Sez6l
chr14	63373538	63373687	0.01792	-21.12408	10	1.7361	22.86	Blk
chr17	55950542	55950888	0.01792	-20.9337	10	0.7738	21.708	
chr7	19593120	19593302	0.01792	-20.91468	10	1.6667	22.581	Clasrp
chr10	76549780	76549931	0.01792	-20.9094	12	8.3333	29.243	Lss & 4930483K19Rik
chr19	6219359	6219746	0.01792	-20.78571	10	7.4643	28.25	Majin
chr17	6739640	6739723	0.01792	-20.76190	10	7.5	28.262	Ezr
chr1	136148648	13614883	0.01792	-20.73898	10	1.2879	22.027	Kif21b
chr15	98851488	98851635	0.01792	-20.69047	10	1	21.69	Kmt2d
chr11	79605542	79605795	0.01792	-20.58231	12	2.5	23.082	Rab11fip4
chr11	5716394	5716554	0.01792	-20.5688	12	1.875	22.444	Urgcp
chr4	1.27E+08	1.27E+08	0.01792	-20.4353	10	1.1111	21.546	Tfap2e
chr9	45097180	45097432	0.01792	-20.42063	10	1.25	21.671	Jaml
chr7	45722395	45722686	0.01792	-20.38525	24	1.7063	22.092	Fam83e
chr17	24606175	24606595	0.01792	-20.07558	21	6.4683	26.544	Tsc2
chr5	35080191	35080370	0.01792	-20.00775	10	5.2363	25.244	Dok7
chr15	76172657	76172976	0.01792	-19.72703	21	6.1999	25.927	Plec
chr7	142306048	14230631	0.01792	-19.51177	17	3.4314	22.943	Gm5054
chr16	11727377	11728023	0.01792	-19.46358	17	2.3958	21.859	Snx29
chr5	63918718	63919018	0.01792	-19.41666	10	5	24.417	Rell1
chr10	59905446	59905567	0.01792	-19.0615	10	3.25	22.312	
chr8	110894625	11089470	0.01792	-18.91865	12	4.8611	23.78	Gm26816 & fucose kinase
chr11	60200193	60200922	0.01792	-18.88201	29	4.3747	23.257	Mir6921 & Srebf1
chr17	46305389	46305555	0.01792	-18.51145	17	4.6674	23.179	Abcc10
chr7	79722643	79723140	0.01792	-18.41666	15	1.6984	20.115	Plin1
chr12	107967552	10796773	0.01792	-18.22835	10	1.3889	19.617	Bcl11b
chr13	43200119	43200412	0.01792	-18.07959	12	1.189	19.269	Gfod1
chr8	70786454	70786698	0.01792	-18.04603	17	6.9118	24.958	Mast3
chr10	1.21E+08	1.21E+08	0.01792	-17.8311	17	1.4706	19.302	Gns
chr9	21587905	21587968	0.01792	-17.78708	10	3.1448	20.932	Carm1
chr5	36294374	36295213	0.01792	-17.76574	21	0.39683	18.163	Sorcs2
chr12	100941572	10094200	0.01792	-17.67081	10	2.7143	20.385	A630072L19Rik & Ccdc88c
chr11	1.21E+08	1.21E+08	0.01792	-17.4588	27	8.4524	25.911	Fasn
chr7	45047059	45047241	0.01792	-17.45375	10	3.0952	20.549	Prr12
chr9	68845962	68846227	0.01792	-17.39102	10	7.9167	25.308	Rora
chr19	5454163	5454520	0.01792	-17.1786	10	2.5	19.679	Fosl1
chr2	29996500	29996924	0.01792	-17.05357	12	3.3333	20.387	Sptan1
chr17	56127365	56127603	0.01792	-16.92595	10	0.45455	17.381	Sema6b
chr15	88004302	88004634	0.01792	-16.841	17	2.451	19.292	
chr17	14196872	14197088	0.01792	-15.71278	12	2.0833	17.796	Dact2
chr6	47905158	47905205	0.01792	-15.18971	12	5.8502	21.04	Zfp282
chr7	43442135	43442493	0.01792	-14.56142	12	1.0417	15.603	Cldnd2
chr11	74840329	74840697	0.01792	-14.2879	10	1.1111	15.399	Mnt
chr5	38200955	38201134	0.01792	-13.7464	10	4.1667	17.913	Zbtb49
chr6	1.18E+08	1.18E+08	0.01792	-13.746	10	7.5	21.246	
chr12	84890630	84890766	0.01792	-13.574	10	0.7692	14.343	
chr12	83157996	83158040	0.01792	-12.25432	10	3.6111	15.865	Rgs6
chr7	6677544	6678749	0.019333	-18.01968	26	2.755	20.775	Zim1

chr4	48552522	48552606	0.019334	-25.84523	20	5.8333	31.679	Msantd3
chr15	98604585	98604773	0.01971	-36.60714	14	3.784	40.391	Adcy6
chr10	43511743	43512191	0.020113	-14.52020	43	4.4326	18.953	Bend3
chr13	55476542	55477087	0.020136	-17.07057	14	4.9249	21.995	Dbn1
chr9	54750418	54751192	0.020158	-24.1783	14	0.5952	24.774	
chr1	135255033	135255298	0.020163	-32.40118	14	0.44643	32.848	Elf3 & Gm26642
chr11	115243230	11524355(	0.02024	-37.15986	14	1.1905	38.35	Tmem104
chr11	1.13E+08	1.13E+08	0.020276	-15.1006	14	5.0765	20.177	Slc39a11
chr8	14777981	14778370	0.020366	-38.5119	14	5.4762	43.988	Dlgap2
chr17	34781581	34782081	0.020384	-40.60387	14	14.396	55	Tnxa
chr15	100482551	10048268	0.020384	-19.13385	14	2.2643	21.398	Csrnp2
chr10	62289937	62290305	0.020416	-18.68990	35	2.2302	20.92	Hk1
chr2	29217662	29218192	0.020486	-14.2746	23	1.498	15.773	Ntng2
chr6	87580668	87581411	0.020637	-23.2475	23	6.8302	30.078	Prokr1
chr15	95948459	95948656	0.020652	-29.53674	23	1.9798	31.517	Ano6
chr13	92789620	92790083	0.020853	-51.8787	16	21.075	72.954	Thbs4
chr18	77356039	77357012	0.020853	-23.50405	20	2.5	26.004	Loxhd1
chrX	1.52E+08	1.52E+08	0.020958	-16.0198	20	3.9167	19.937	
chr8	106026016	10602702(	0.021035	-31.20701	18	4.0542	35.261	Dus2
chr15	86117874	86118622	0.021035	-19.8865	20	1.5417	21.428	Gramd4
chr10	80586666	80586790	0.021035	-19.4405	20	0.8333	20.274	Abhd17a
chr4	150678487	15067887	0.021035	-19.29464	20	7.0833	26.378	Gm16079
chr3	88112723	88113262	0.021043	-24.77795	20	2.7719	27.55	Iqgap3
chr17	26275732	26276534	0.021043	-19.29647	23	2.1676	21.464	Luc7l
chr18	82729752	82729958	0.021043	-12.0841	28	1.79	13.874	
chrX	141874092	14187456	0.021057	-25.92563	23	1.9082	27.834	Gm15294
chr2	166451427	16645272	0.021064	-25.07575	20	3.2986	28.374	5031425F14Rik
chr7	30574772	30575578	0.021156	-15.92563	23	3.3514	19.277	Kmt2b
chr14	117652385	11765297	0.021532	-47.59672	16	1.8229	49.42	Gpc6
chr16	89867498	89867642	0.021562	-29.84668	18	0.5303	30.377	Tiam1
chr8	12178348	12178531	0.021884	-22.4764	16	2.2917	24.768	
chr11	97835901	97836188	0.0219	-19.33248	28	1.1905	20.523	Lasp1
chr13	48832015	48832301	0.022212	-23.7443	18	2.7372	26.481	Phf2
chr11	67099623	67099772	0.022212	-20.08680	16	2.1875	22.274	Myh3
chr4	139417209	13941734	0.022249	-43.89012	16	12.481	56.372	Ubr4
chr3	1.43E+08	1.43E+08	0.022249	-37.2396	16	0.625	37.865	Kyat3
chr18	10647606	10647829	0.022249	-36.64806	16	2.5347	39.183	Abhd3
chr10	1.28E+08	1.28E+08	0.022284	-27.1652	16	2.5	29.665	Zbtb39
chr5	38673685	38674190	0.022365	-22.40465	28	1.7503	24.155	Zfp518b
chr2	25898426	25898781	0.022393	-26.21775	16	5.2083	31.426	Kcnt1
chr5	1.15E+08	1.15E+08	2.24E-02	-33.065	16	6.25	39.315	Hnf1a
chr6	1E+08	1E+08	0.022457	-29.1592	16	2.0089	31.168	
chr5	1.16E+08	1.16E+08	0.022457	-28.3705	16	5.1562	33.527	Ccdc60
chr11	104290316	10429063	0.022457	-26.46205	16	1.5625	28.025	Mapt
chr2	25055734	25055947	0.022467	-11.50297	16	4.1667	15.67	Nsmf
chr9	1.18E+08	1.18E+08	0.022503	-47.7083	16	3.2292	50.938	
chr3	89181738	89181927	0.022503	-38.43006	16	7.5893	46.019	Gm16069 & Scamp3
chr19	7095133	7095281	0.022503	-37.58522	16	8.6979	46.283	Flrt1 & Macrod1
chr5	66890305	66890847	0.022503	-35.17485	16	1.5625	36.737	Gm6517 & Limch1
chr17	31591182	31591760	0.022503	-35.05494	13	4.8077	39.863	Pknox1
chr1	168109482	16811014	0.022503	-32.67237	16	1.5625	34.235	Gm20711
chr19	3268437	3268839	0.022503	-30.23897	16	2.7344	32.973	Ighmbp2
chr17	25035555	25035915	0.022503	-28.34821	16	0.78125	29.129	Ift140
chr1	82287697	82287981	0.022503	-26.59188	18	3.6065	30.198	Irs1
chr6	121220463	12122067	0.022503	-26.46722	18	3.1668	29.634	Tuba8
chrX	37210831	37211365	0.022503	-24.6993	16	0.625	25.324	Gm9
chr2	1.2E+08	1.2E+08	0.022503	-23.8251	16	4.3056	28.131	Mapkbp1

chr4	139666906	139667256	0.022503	-23.06592	16	2.1875	25.253	Tas1r2
chr17	66344170	66344284	0.022503	-18.67086	16	0.39062	19.061	Mtcl1
chr2	166567207	166567827	0.022619	-28.44899	24	2.7266	31.176	Prex1
chr10	69096613	69096793	0.023293	-29.59124	13	1.9231	31.514	Gm47107
chr5	134990654	134990810	0.023379	-28.16239	13	0.64103	28.803	Wbscr25
chr2	1.63E+08	1.63E+08	0.023397	-34.1026	13	3.8462	37.949	Jph2
chr5	35701771	35702046	0.023451	-32.76848	13	8.4266	41.195	Sh3tc1
chr10	63116885	63116980	0.02353	-31.95054	13	0.54945	32.5	Mypn
chr15	78828086	78828214	0.023603	-32.002	13	2.4946	34.497	
chr8	123193935	123194208	0.023744	-31.30341	13	0.64103	31.944	Dpep1 & Gm20388
chr3	1.08E+08	1.08E+08	0.02381	-39.7299	13	2.5641	42.294	Sars
chr4	149649791	149649939	0.02381	-15.99640	13	3.4615	19.458	Pik3cd
chr2	122075226	122075716	0.023911	-21.82997	13	4.3956	26.226	Spg11
chr10	79912411	79912458	0.023932	-29.02319	13	6.9658	35.989	R3hdm4
chr9	107288117	107288445	0.024035	-27.35357	13	3.0112	30.365	Mapkapk3
chr7	24698979	24699280	0.024037	-33.6538	13	0.9615	34.615	
chr1	136219809	136219984	0.024037	-30.70818	13	1.3355	32.044	Inava & Gm26568
chr2	30330714	30331488	0.02405	-16.5917	13	1.9231	18.515	Nup188
chr2	93875837	93876241	0.024138	-37.9258	13	2.5641	40.49	
chr6	39769485	39770158	0.024138	-28.4585	13	1.9643	30.423	
chr6	121654417	121654597	0.024141	-49.20940	13	8.6538	57.863	A2m
chr4	128998271	128998337	0.024141	-42.14285	13	0.54945	42.692	Ak2
chr6	89323118	89323671	0.024466	-32.53590	21	1.3983	33.934	Gm26811 & plexin A1
chr4	118142204	118142605	0.024772	-19.74927	22	8.1494	27.899	Kdm4a
chr11	95023322	95023864	0.024931	-18.88650	25	2.7333	21.62	Samd14
chr5	1.39E+08	1.39E+08	0.025052	-16.3889	15	4.1111	20.5	
chr7	37346697	37347718	0.025269	-31.5836	25	2.3056	33.889	
chr10	1.17E+08	1.17E+08	0.025369	-31.8333	15	3.3333	35.167	
chr15	98378627	98378957	2.62E-02	-21.105	15	1.1111	22.216	Olfr283
chr15	52125402	52125480	0.02675	-52.7778	12	28.819	81.597	
chr4	130774438	130774585	0.02675	-38.05916	15	1.6984	39.758	Pum1
chr15	1.01E+08	1.01E+08	0.026908	-25.3677	15	3.8333	29.201	Acvr1b
chr15	74898116	74898349	0.026996	-27.5731	19	0.8772	28.45	
chr7	44597827	44598570	0.026996	-17.20214	15	4.3704	21.573	Kcnc3
chr11	59557738	59558670	0.027012	-27.1329	12	0.9375	28.07	Nlrp3
chr7	127489801	127489928	0.027059	-45.55555	15	6.5	52.056	Fbrs
chr11	117818848	117819150	0.027108	-26.16137	15	2.254	28.415	Tk1 & Gm20708
chr2	1.73E+08	1.73E+08	0.027327	-29.4707	12	4.9555	34.426	
chr8	84211956	84212128	0.027327	-25.60185	15	0.95238	26.554	Zswim4
chr4	1.33E+08	1.33E+08	0.027437	-37.2407	15	2.8889	40.13	Themis2
chr1	184912574	184912773	0.027451	-25.95238	15	1.3889	27.341	Mark1
chr7	49362105	49362433	0.027543	-21.33068	15	1.6667	22.997	Nav2
chr8	114781305	114781899	0.027582	-24.77777	15	1.1111	25.889	Wwox
chr13	57787077	57787551	0.027622	-34.373	15	3.3333	37.706	Spock1
chr12	1.07E+08	1.07E+08	0.027622	-33.8591	12	4.7917	38.651	
chr7	45532707	45533216	0.027622	-31.30952	15	6.6111	37.921	Plekha4
chr8	95577696	95577929	0.027622	-30.8218	12	7.6042	38.426	Gm5912
chr6	124728748	124729201	0.027622	-29.08465	15	4.6667	33.751	Ptpn6
chr4	63136916	63137242	0.027622	-28.08441	22	2.5433	30.628	Zfp618
chr8	110731603	110732115	0.027622	-23.31210	22	6.1387	29.451	Mtss1l
chr11	1.15E+08	1.15E+08	0.027622	-20.9209	15	1.3889	22.31	Rab37 & Cd300lf
chr5	134503197	134503316	0.027636	-19.48687	15	2.8889	22.376	Clip2
chr1	36795052	36795365	0.027648	-50.34722	12	4.2063	54.554	Tmem131
chr17	26213286	26213526	0.027723	-22.84547	15	1.5278	24.373	Fam234a
chr15	98843957	98844571	0.027723	-17.35269	15	0.89286	18.246	Kmt2d
chr1	7178210	7178917	0.027754	-54	15	2.7778	56.778	Gm9826

chr17	7255299	7255623	0.027754	-48.4028	12	5.8333	54.236	Rps6ka2
chr5	1.43E+08	1.43E+08	0.027754	-37.5694	12	10.764	48.333	Zfp12
chr6	121040440	12104055[illegible]	0.027754	-35.45634	12	1.6369	37.093	Mical3
chr3	89359164	89359651	0.027754	-35.41997	12	1.8254	37.245	Dcst1
chr2	181207664	18120824[illegible]	0.027754	-35.24603	15	1.8095	37.056	Srms
chr4	1.49E+08	1.49E+08	0.027754	-33.1944	12	3.4722	36.667	Kif1b
chr5	34001056	34001216	0.027754	-33.16919	22	3.355	36.524	Nat8l
chr8	8520580	8520717	0.027754	-30.8333	15	1.6667	32.5	
chr2	29179697	29180215	0.027754	-30.7976	15	4.119	34.917	Setx
chr12	81718208	81718607	0.027754	-30.53968	15	10.667	41.206	AC125351.1
chr7	25157346	25157502	0.027754	-30.41666	12	3.6111	34.028	Pou2f2
chr17	65679895	65680391	0.027754	-28.2143	12	2.0833	30.298	Rab31
chr17	30803342	30803526	2.78E-02	-27.5907	22	5.6331	33.224	Dnah8
chr17	71675109	71675192	0.027754	-27.35389	15	6.9444	34.298	Togaram2
chr19	5761448	5761721	0.027754	-23.4937	12	3.6111	27.105	Scyl1
chr6	91056805	91057463	0.027754	-22.40608	15	6.5317	28.938	Nup210
chr3	152555289	15255542[illegible]	0.027754	-22.19576	12	3.3598	25.556	Ak5
chr11	18603753	18603950	0.027754	-16.10101	15	3.3333	19.434	Gm12023
chr8	1.23E+08	1.23E+08	0.027754	-15.6641	15	5.2778	20.942	Galns & Gm20388
chr7	38188683	38188971	0.027754	-14.99518	12	7.4097	22.405	D530033B14Rik & 1600014C10Rik
chr12	111810827	11181095[illegible]	0.027754	-10.02381	15	2.4444	12.468	Xrcc3
chr6	125758294	12575872[illegible]	0.027758	-28.11904	15	2.1429	30.262	Ano2
chr19	47267631	47267797	0.028396	-28.35901	17	8.6928	37.052	Sh3pxd2a
chr12	59491748	59492092	0.029823	-31.5584	11	0.7576	32.316	
chr3	52600880	52601422	0.031001	-28.1042	20	8.0923	36.196	
chr1	87210276	87210674	0.031087	-20.0516	20	2.375	22.427	Chrng
chr11	1.18E+08	1.18E+08	0.031229	-48.9177	11	3.3333	52.251	Timp2
chr1	1.82E+08	1.82E+08	0.031236	-23.5479	11	0.5051	24.053	
chr19	6481461	6481541	0.031738	-35.06673	11	1.5152	36.582	Nrxn2
chr12	8952352	8952667	0.031738	-28.24288	14	6.746	34.989	Matn3
chr3	65613596	65614047	0.031738	-27.1645	11	1.4069	28.571	Gm5847
chr7	44844083	44844335	0.031797	-18.57599	24	2.2859	20.862	Tbc1d17
chr2	25441404	25441448	0.031827	-23.34901	11	10.813	34.163	Abca2
chr9	47268908	47268995	0.031941	-71.90476	10	8.0952	80	Gm31816
chr19	23675705	23675915	0.031941	-71.25000	10	6.25	77.5	Gm6563
chr14	17884818	17885026	0.031941	-69.83333	10	1	70.833	Gm24140 & thyroid hormone receptor beta
chr14	121336343	12133699[illegible]	0.031941	-62.52381	10	1.6667	64.19	Stk24
chr18	90294692	90294986	0.031941	-60.5032	10	10.512	71.015	
chr1	161241585	16124181[illegible]	0.031941	-56.91666	10	11.417	68.333	Prdx6
chrX	103551402	10355162[illegible]	0.031941	-54.66666	10	3.3333	58	Jpx
chr7	27537505	27537659	0.031941	-53.25595	10	4.9583	58.214	Pld3
chr12	1.11E+08	1.11E+08	0.031941	-50.9762	10	3.3333	54.31	Cdc42bpb
chr3	83304888	83305050	0.031941	-49.95833	10	0.625	50.583	Dchs2
chr16	13424509	13424751	0.031941	-49.59523	10	1	50.595	Gm15738
chr18	78069752	78069907	0.031941	-49.1667	10	2.5	51.667	
chr5	130906106	13090624[illegible]	0.031941	-49.04166	10	2.2917	51.333	Galnt17
chr6	85219929	85220077	0.031941	-48.31313	10	0.90909	49.222	Sfxn5
chr11	98579195	98579405	0.031941	-47.9048	10	3.6667	51.571	
chr17	46980600	46980708	0.031941	-47.41666	10	11.75	59.167	Ubr2
chr13	45593629	45593767	0.031941	-46.50000	10	4.3333	50.833	Atxn1
chr2	127755337	12775566[illegible]	0.031941	-45.88095	10	5.7143	51.595	Nphp1
chr5	112862377	11286248[illegible]	0.031941	-45.81349	10	6.2698	52.083	Myo18b
chr6	37610507	37610658	0.031941	-45.8078	14	5.2976	51.105	
chr5	148028376	14802866[illegible]	0.031941	-45.75000	10	5	50.75	Mtus2
chr17	7655553	7655861	0.031941	-45.69642	10	2.6667	48.363	AC119998.5

chrUn_	7617	7728	0.031941	-45.6667	10	1.8333	47.5	
chr11	1.04E+08	1.04E+08	0.031941	-45.3651	10	1	46.365	Crhr1
chr10	78284916	78285131	0.031941	-45.10714	10	9.2262	54.333	Agpat3
chr7	98098110	98098252	0.031941	-44.5357	10	5	49.536	Myo7a
chr19	46887207	46887520	0.031941	-44.4306	10	1	45.431	Nt5c2
chr10	75927350	75927450	0.031941	-43.51190	10	3.75	47.262	Mmp11
chr7	1.18E+08	1.18E+08	0.031941	-42.6964	10	9.4702	52.167	
chr16	19036087	19036313	0.031941	-42.5556	10	2.9861	45.542	
chr11	1.2E+08	1.2E+08	0.031941	-41.4762	10	3.3929	44.869	
chr3	94453585	94453940	0.031941	-41.2381	10	1.4286	42.667	Gm30023
chr2	173008121	17300857	0.031941	-40.72023	10	0.625	41.345	Rae1
chr15	72590736	72590784	0.031941	-40	10	3.75	43.75	Gm49460 & trafficking protein particle complex 9
chr10	121868761	12186902	0.031941	-39.08333	10	1.25	40.333	Srgap1
chr13	96748460	96748575	0.031941	-38.88095	10	2.7143	41.595	Ankrd31
chr16	20529118	20529297	0.031941	-38.75	10	1.8333	40.583	Dvl3
chr5	1.14E+08	1.14E+08	0.031941	-38.6429	10	1.6667	40.31	
chr16	52102259	52102708	0.031941	-38.25	10	8.4167	46.667	Cblb
chr12	1.07E+08	1.07E+08	0.031941	-38.1548	10	6.8452	45	
chr6	85933227	85933390	0.031941	-37.80952	10	1.6667	39.476	Nat8b-ps
chr2	5897917	5898047	0.031941	-37.6151	10	3.875	41.49	Gm13267 & Dhtkd1
chr17	28902980	28903296	0.031941	-37.31547	10	2.7917	40.107	4930539E08Rik
chr4	131849157	13184926	0.031941	-37.21428	10	4.3333	41.548	Mecr
chr5	115894399	11589495	0.031941	-37.05952	10	7.8333	44.893	Cit
chr3	98826433	98826852	0.031941	-35.9867	14	0.7143	36.701	
chr15	96381874	96382342	0.031941	-35.87963	18	6.0185	41.898	Arid2
chr2	1.58E+08	1.58E+08	0.031941	-35.7483	14	4.1667	39.915	
chr7	34284856	34285023	0.031941	-35.62139	10	3.9583	39.58	4931406P16Rik
chr17	78333872	78334533	0.031941	-35.58333	10	0.83333	36.417	Crim1
chr1	77975884	77976060	0.031941	-35.3038	10	5.6627	40.966	
chr8	1.18E+08	1.18E+08	0.031941	-35.0595	14	10.714	45.774	
chr9	1.16E+08	1.16E+08	0.031941	-35.0468	14	1.2245	36.271	
chr6	137513901	13751404	0.031941	-34.42857	10	4.9286	39.357	Eps8
chr19	5763431	5763698	0.031941	-34.23015	10	2.4444	36.675	Scyl1
chr3	95320697	95321014	0.031941	-34.20400	10	2.4286	36.633	Cers2
chr2	172912217	17291243	0.031941	-33.65705	10	4.4918	38.149	Bmp7
chr1	1.8E+08	1.8E+08	0.031941	-33.6513	10	2.6591	36.31	Cdc42bpa
chr14	51255405	51255677	0.031941	-33.4087	10	3	36.409	Rnase2a
chr7	1.28E+08	1.28E+08	0.031941	-32.7785	10	6.5952	39.374	Zfp646
chr7	141634001	14163447	0.031941	-32.58503	14	5.0595	37.645	Muc6
chr11	1.01E+08	1.01E+08	0.031941	-32.3452	10	8.4167	40.762	Ghdc
chr14	25806369	25806631	0.031941	-32.1429	10	0.8333	32.976	
chr11	50156967	50157020	0.031941	-32.04960	10	3.6667	35.716	Tbc1d9b
chr4	44984351	44984451	0.031941	-31.73809	10	4.5	36.238	Grhpr
chr6	97647780	97647858	0.031941	-31.1411	10	10.526	41.667	
chr9	108045579	10804570	0.031941	-31.08333	10	2	33.083	Ip6k1
chr11	1.03E+08	1.03E+08	0.031941	-31.0099	10	1.1806	32.19	Plekhm1
chr10	128949890	12895006	0.031941	-31.00058	10	3.8474	34.848	Itga7
chr4	148932824	14893297	0.031941	-30.93614	14	1.3095	32.246	Casz1
chr7	30765796	30766135	0.031941	-30.91666	10	2.1032	33.02	Dmkn
chr17	31348107	31348458	0.031941	-30.88888	10	0.625	31.514	Slc37a1
chr14	1.22E+08	1.22E+08	0.031941	-30.7473	14	3.9626	34.71	Dock9
chr15	37346655	37346948	0.031941	-30.70238	10	1.25	31.952	Grhl2
chr5	115545958	11554613	0.031941	-30.67261	10	0.625	31.298	Pxn
chr17	45539396	45539757	0.031941	-30.00948	14	4.1618	34.171	Tcte1

chr3	1.54E+08	1.54E+08	0.031941	-30	10	3.25	33.25	Gm22136 & ST6 (alpha-N-acetyl-neuraminyl-2,3-beta-galactosyl-1,3)-N-acetylgalactosaminide alpha-2,6-sialyltransferase 3
chr2	103722622	10372322	0.031941	-29.84127	10	8.5278	38.369	Nat10
chr5	1.4E+08	1.4E+08	0.031941	-29.4464	10	3	32.446	
chr5	140628256	14062869	0.031941	-29.37500	10	8.6667	38.042	Ttyh3
chr10	80520400	80520522	0.031941	-29.0136	14	0.8929	29.906	Atp8b3
chr6	1.25E+08	1.25E+08	0.031941	-28.9711	14	0.7143	29.685	Ptpn6
chr6	1.16E+08	1.16E+08	0.031941	-28.2882	21	6.7442	35.032	Rho
chr6	91679216	91679515	0.031941	-28.16774	10	2.5	30.668	Gm45217
chr8	1.17E+08	1.17E+08	0.031941	-28.1593	10	4.6032	32.762	Cmip
chr10	79829807	79830151	0.031941	-28.11111	10	6.7698	34.881	Misp
chr2	168098684	16809876	0.031941	-27.87554	10	1.5556	29.431	Pard6b
chr2	181411932	18141214	0.031941	-27.58531	10	2.2917	29.877	Zbtb46
chr4	116050900	11605142	0.031941	-27.45634	10	0.55556	28.012	Nsun4
chr1	163939764	16394009	0.031941	-27.36819	14	5.6122	32.98	Scyl3
chr5	35948111	35948194	0.031941	-27.3633	10	2.5	29.863	Afap1
chr12	91538044	91538203	0.031941	-27.30158	10	4.3651	31.667	Tshr
chr17	46721795	46721868	0.031941	-27.12159	14	2.5	29.622	Pex6 & Gm26904
chr15	101736430	10173674	0.031941	-26.66062	20	2.8959	29.557	Krt71
chr7	80312948	80313264	0.031941	-26.6369	10	5.625	32.262	Rccd1 & Prc1
chr14	30307593	30307988	0.031941	-26.54478	14	7.1429	33.688	Cacna1d
chr6	1.28E+08	1.28E+08	0.031941	-26.5357	10	3.6786	30.214	Gm26770 & RAD9-HUS1-RAD1 interacting nuclear orphan 1
chr7	46166949	46167115	0.031941	-26.39087	10	4.4821	30.873	Abcc8
chr9	119348211	11934870	0.031941	-26.39030	14	0.71429	27.105	Acaa1a & Slc22a14
chr8	121564770	12156507	0.031941	-26.37755	14	0.89286	27.27	Fbxo31 & Gm20388
chr13	54733556	54733831	0.031941	-26.36904	14	5.9666	32.336	Cdhr2
chr1	1.29E+08	1.29E+08	0.031941	-26.3571	10	9	35.357	
chr12	107949686	10795003	0.031941	-26.35317	10	2.2222	28.575	Bcl11b
chr4	130011549	13001189	0.031941	-25.38770	14	1.4286	26.816	Adgrb2
chr5	35627594	35627762	0.031941	-25.2262	10	0.625	25.851	
chr12	1.13E+08	1.13E+08	0.031941	-25.1392	10	5	30.139	Inf2
chr2	160908220	16090835	0.031941	-25.03282	10	5.6905	30.723	Emilin3
chr10	75976437	75976601	0.031941	-25.0278	10	1.2698	26.298	Gm5134
chr9	98437509	98437834	0.031941	-24.91071	10	3.25	28.161	Rbp1
chr10	127705345	12770575	0.031941	-24.86111	10	1.2698	26.131	Myo1a
chr9	65556618	65557099	0.031941	-24.83080	10	0.83333	25.664	Plekho2
chr6	90671975	90672109	0.031941	-24.80952	20	0.35714	25.167	Iqsec1
chr15	84693679	84693914	0.031941	-24.51677	10	4.2123	28.729	Prr5
chr5	123816815	12381705	0.031941	-24.25000	10	8	32.25	Kntc1
chr11	77505444	77505590	0.031941	-24.1752	14	1.7857	25.961	Git1
chr16	13611978	13613113	0.031941	-24.1355	14	2.9266	27.062	Parn
chr10	81080578	81080727	0.031941	-23.65955	14	4.5238	28.183	Thop1
chrX	94150197	94150689	0.031941	-23.5714	14	7.1429	30.714	
chr7	1.18E+08	1.18E+08	0.031941	-23.4638	14	0.8929	24.357	Xylt1
chr5	120128271	12012857	0.031941	-23.03246	10	4.5833	27.616	Gm10390 & Rbm19
chr8	14761337	14761571	0.031941	-23.01190	10	0.625	23.637	Dlgap2

chr2	174644962	174645169	0.031941	-22.66156	14	0.71429	23.376	Zfp831
chr17	25825667	25825765	0.031941	-22.33333	10	1.3333	23.667	Wdr24 & Gm26694
chr6	83296342	83296654	0.031941	-22.1429	10	4.3333	26.476	Slc4a5
chr5	142884752	142885473	0.031941	-21.68792	14	2.877	24.565	Fbxl18
chr10	62271534	62271708	0.031941	-21.68367	14	3.869	25.553	Hk1
chr3	59430075	59430694	0.031941	-21.6	14	1.1905	22.79	
chr9	121110321	121110551	0.031941	-21.20021	10	6.2266	27.427	Ulk4
chr5	24648373	24648612	0.031941	-20.85546	14	1.8132	22.669	1700022A21Rik
chr5	122247512	122247737	0.031941	-20.39357	10	4.3333	24.727	Tctn1
chr11	1.19E+08	1.19E+08	0.031941	-20.375	10	1.625	22	Rbfox3
chr7	1.24E+08	1.24E+08	0.031941	-19.9982	10	11.269	31.267	
chr7	44209507	44209714	0.031941	-19.85191	10	3.6806	23.532	Klk1b4
chr2	1.59E+08	1.59E+08	0.031941	-19.5629	18	2.4109	21.974	Ppp1r16b
chr17	85065691	85065768	0.031941	-19.55573	10	6.1448	25.701	Prepl
chr12	90332161	90332240	0.031941	-18.53968	10	0.71429	19.254	Nrxn3
chr3	68787770	68788069	0.031941	-18.0782	14	0.5952	18.673	
chr2	168754592	168754742	0.031941	-17.94501	14	1.4881	19.433	Sall4
chr14	57036550	57036725	0.031941	-17.89614	24	1.3021	19.198	Gja3
chr19	8549543	8549917	0.031941	-17.5099	10	9.4048	26.915	
chr17	24595221	24595664	0.031941	-17.38780	10	6.4683	23.856	Pkd1
chr5	125496511	125497133	0.031941	-17.10446	14	0.32468	17.429	Aacs
chr11	99145583	99145634	0.031941	-16.9351	10	4.1071	21.042	Ccr7
chr8	125807984	125808204	0.031941	-16.10119	14	3.5714	19.673	Pcnx2
chr13	48806699	48808039	0.031941	-16.0995	24	7.0817	23.181	Phf2
chr15	98650807	98651022	0.031941	-14.17225	10	1.9091	16.081	Ddx23
chr19	56847614	56848047	0.031941	-12.91591	10	3.5	16.416	Tdrd1
chr8	121554255	121554299	0.031941	-12.68011	14	2.0833	14.763	Fbxo31 & Gm20388
chr15	79034791	79035834	0.031941	-12.37381	24	1.5418	13.916	Gcat & Gcat
chr5	125149139	125149294	0.031941	-12.27782	14	1.7857	14.064	Ncor2
chr4	1.19E+08	1.19E+08	0.031941	-7.68948	14	6.5795	14.269	
chr13	37510492	37511199	0.031952	-28.2596	14	1.7857	30.045	Gm29590
chr8	1.23E+08	1.23E+08	0.031956	-31.8027	14	3.7925	35.595	Gm20388 & CBFA2/RUNX1 translocation partner 3
chr7	110802533	110802957	0.031976	-34.71119	18	5.0132	39.724	Gm47282 & adenosine monophosphate deaminase 3 & ring finger protein 141
chr8	121510380	121510575	0.031979	-22.99358	14	0.59524	23.589	Gm20388
chr10	80787119	80787981	0.031993	-40.18319	14	1.3095	41.493	Dot1l
chr18	46932357	46932452	0.031993	-23.5884	14	7.7041	31.293	Arl14epl
chr4	134898448	134898692	0.031993	-21.29251	14	0.89286	22.185	Tmem50a
chr2	31816539	31816804	0.032142	-17.83559	18	2.1605	19.996	Fibcd1
chr1	1.54E+08	1.54E+08	0.032186	-26.9279	18	4.6373	31.565	Npl
chr15	86108113	86108601	0.032308	-32.93049	18	4.1777	37.108	Gramd4
chr7	99344050	99344712	0.032372	-22.672	18	1.8783	24.55	
chr1	86305101	86305374	0.032626	-17.58597	18	2.9541	20.54	B3gnt7
chr7	105758741	105759202	0.032695	-19.14021	18	0.39683	19.537	Dchs1
chr12	102345472	102346395	0.032771	-36.38888	18	5.787	42.176	Rin3
chr10	75001660	75001928	0.03278	-13.72354	18	0.27778	14.001	Rsph14 & Gnaz
chr18	75426108	75426690	0.032803	-19.427	18	1.5542	20.981	
chr9	57615768	57616448	0.032847	-19.81764	21	6.2302	26.048	Lman1l
chr7	1.39E+08	1.39E+08	0.032987	-38.7202	16	3.6756	42.396	

chr11	3924650	3925248	0.033079	-35.5506	16	3.125	38.676	Tcn2
chrX	37827065	37827460	0.033139	-19.36533	21	1.7857	21.151	Rhox6
chr4	154268589	154268685	0.033273	-20.28138	16	2.3153	22.597	Megf6
chr5	123996871	123997100	0.03329	-17.80328	21	0.19841	18.002	Mir7032 & Hip1r
chr7	144607864	144608044	0.033384	-28.31632	21	5.5556	33.872	Ano1
chr10	9683649	9684132	0.033536	-32.89115	21	5.7143	38.605	Gm6150
chr10	1.28E+08	1.28E+08	0.033601	-16.0364	21	4.1213	20.158	Rnf41
chr5	52538046	52538473	0.033871	-26.76711	16	2.5521	29.319	Lgi2
chr11	1.19E+08	1.19E+08	0.033871	-20.1146	21	1.0204	21.135	Ccdc40
chr7	79569590	79570700	0.034138	-21.4943	21	3.6574	25.152	
chr3	89941555	89941773	0.034205	-25.2537	21	10.556	35.809	Atp8b2
chr15	102452381	102452638	0.034205	-16.75504	21	2.3413	19.096	Amhr2
chr5	1.43E+08	1.43E+08	0.034659	-9.88585	13	6.4103	16.296	Tnrc18
chr4	152012293	152012445	0.035369	-22.21611	19	1.372	23.588	Klhl21
chr11	120070318	120070379	0.036134	-20.21395	13	7.8846	28.099	Cep131
chr17	10400814	10401293	0.036538	-30.2549	26	5.8059	36.061	
chr9	69636774	69637016	0.036678	-53.8462	13	5.1282	58.974	
chr12	1.06E+08	1.06E+08	0.036678	-18.8233	13	7.6923	26.516	
chr13	3221721	3221928	0.036682	-51.4103	13	1.2821	52.692	
chr6	98958126	98958675	0.036764	-33.26312	13	2.0513	35.314	Foxp1
chr11	68505009	68505228	0.036858	-24.2247	13	5.8974	30.122	Pik3r6
chr10	62062248	62063400	0.037038	-23.518	26	4.9679	28.486	
chr8	72757609	72758281	0.037155	-21.35531	13	6.4103	27.766	Sin3b
chr6	1.38E+08	1.38E+08	0.037182	-20.9959	26	7.9327	28.929	Dera
chr7	44552189	44552339	0.037262	-22.37072	19	8.2655	30.636	Nr1h2
chr4	103563384	103563560	0.037575	-22.96139	13	1.9745	24.936	Gm12715
chr4	122948802	122949217	0.037724	-31.46270	13	4.1667	35.629	Mfsd2a
chr9	46297686	46298024	0.037724	-21.32020	13	0.38462	21.705	Bud13
chr5	1.29E+08	1.29E+08	0.037751	-40.1923	13	3.4615	43.654	
chr17	56168699	56168891	0.037757	-31.29537	13	1.69	32.985	Tnfaip8l1
chr16	92842525	92843569	0.037781	-37.4451	13	1.9231	39.368	
chr9	1.03E+08	1.03E+08	0.037936	-24.7573	13	3.5839	28.341	
chr3	58340964	58341590	0.037936	-23.5027	13	9.6154	33.118	
chr6	1.15E+08	1.15E+08	0.037936	-20.409	13	1.4957	21.905	Atg7
chr9	56892376	56892463	0.037937	-21.47435	13	1.9231	23.397	Cspg4
chr9	56888234	56888472	0.037937	-20.31898	13	1.2821	21.601	Cspg4
chr8	124683700	124683942	0.03794	-31.53846	13	1.0989	32.637	Ttc13
chr19	27323829	27323903	0.037959	-44.04761	13	1.9231	45.971	Kcnv2
chr14	63148950	63149249	0.037959	-24.76856	13	0.96154	25.73	Fdft1
chr4	155454490	155454881	0.037959	-23.42185	13	3.0769	26.499	Cfap74
chr10	75173512	75173780	0.037959	-18.31807	13	0.81197	19.13	Bcr
chr4	56018110	56018470	0.038005	-56.4103	13	5.7692	62.179	
chr7	25081492	25081534	0.038005	-46.03590	13	3.022	49.058	Zfp574
chr11	102306750	102307079	0.038005	-24.42182	13	1.6712	26.093	Ubtf
chr14	57699839	57699955	0.038005	-21.92363	13	6.0806	28.004	Lats2 & Gm49361
chr8	121556456	121556729	0.038005	-21.26068	13	0.96154	22.222	Fbxo31 & Gm20388
chr17	25171261	25171602	0.038005	-16.0445	13	1.0302	17.075	Ccdc154
chr12	1.11E+08	1.11E+08	0.038011	-37.096	13	1.6026	38.699	Cdc42bpb
chr15	97051954	97052376	0.038011	-33.71794	13	1.7308	35.449	Slc38a4
chr19	60079880	60080048	0.038011	-32.2985	13	4.7527	37.051	
chr1	89852504	89852969	0.038011	-31.73076	13	4.8077	36.538	Agap1
chr2	91497000	91497125	0.038011	-29.41391	13	1.0989	30.513	Lrp4
chr11	115957555	115957863	0.038011	-27.94871	13	4.4872	32.436	Sap30bp
chr8	1.18E+08	1.18E+08	0.038011	-26.2821	13	2.793	29.075	
chr12	85467618	85467989	0.038011	-24.5421	13	4.533	29.075	
chr7	141360283	141360461	0.038011	-23.17765	13	1.9231	25.101	Eps8l2 & B230206H07Rik

chr18	16316499	16317412	0.038011	-20.9982	13	5.1282	26.126	
chr14	69801393	69801684	0.038011	-17.79609	13	2.5641	20.36	Rhobtb2
chr5	123531704	12353190	0.038011	-16.28205	13	9.6154	25.897	Vps33a
chr5	37130443	37130852	0.038011	-15.4536	13	7.1795	22.633	Jakmip1 & Gm1043
chr9	121788891	12178896	0.038033	-31.13553	13	8.9744	40.11	Hhatl
chr1	1.83E+08	1.83E+08	0.038037	-35.9295	13	0.7692	36.699	
chr1	188944744	18894488	0.038037	-26.50641	13	2.5641	29.071	Ush2a
chr11	89391915	89392006	0.038038	-31.52014	13	1.9231	33.443	Ankfn1
chr10	40051802	40052040	0.038038	-28.7485	13	3.8462	32.595	Slc16a10
chr5	31077530	31077627	0.038038	-23.76956	13	4.9575	28.727	Cad
chr12	1.09E+08	1.09E+08	0.038049	-34.0378	13	1.6667	35.704	Wdr25
chr15	80388408	80388497	0.038049	-29.06232	13	8.7821	37.844	Cacna1i
chr2	165681403	16568160	0.038049	-21.64835	13	5.7692	27.418	Eya2
chr2	27352772	27353039	0.038049	-18.00241	13	3.4615	21.464	Vav2
chr8	45939658	45939803	0.038058	-33.58974	13	1.9231	35.513	Ccdc110
chr4	63297825	63297987	0.038058	-32.41453	13	6.7308	39.145	Col27a1
chr4	40327793	40328407	0.038058	-30.5769	13	0.7692	31.346	
chr18	82598749	82599071	0.038058	-30.2701	13	4.5879	34.858	Zfp236
chr7	65701616	65702024	0.038058	-29.37479	13	0.48077	29.856	Tm2d3
chr16	4633304	4633763	0.038058	-28.25854	13	2.3504	30.609	Coro7
chr2	29714012	29714567	0.038058	-21.74062	13	0.96154	22.702	Rapgef1
chr13	37919227	37919520	0.038058	-20.4762	13	2.4725	22.949	Rreb1
chr4	134673182	13467357	0.038058	-20.42735	13	2.5641	22.991	Man1c1
chr7	1.37E+08	1.37E+08	0.038058	-20.3846	13	0.9615	21.346	
chr8	122833416	12283366	0.038058	-19.36956	13	1.2821	20.652	Gm20388
chr1	127573159	12757415	0.038058	-18.64427	13	3.8462	22.49	Tmem163
chr8	1.2E+08	1.2E+08	0.038058	-16.6758	13	0.9615	17.637	
chr14	20032410	20032787	0.038058	-16.047	13	2.5641	18.611	
chr16	5278674	5279222	0.038347	-36.66040	19	1.9361	38.596	Gm15983
chr17	27434612	27434801	0.038739	-21.7373	17	4.7037	26.441	Grm4
chr8	9624971	9625716	0.039474	-37.8472	12	1.7361	39.583	Fam155a
chr7	144611644	14461187	0.039646	-20.92132	23	1.087	22.008	Ano1
chr10	79821676	79822061	0.040256	-25.9629	17	1.6667	27.63	Misp
chr10	80901885	80902301	0.040319	-26.5079	17	0.4202	26.928	Lmnb2
chr17	30729560	30729957	0.040513	-43.00000	15	0.83333	43.833	Dnah8
chr8	110738351	11073849	0.04058	-30.88235	17	0.73529	31.618	Mtss1l
chr8	79149121	79150099	0.040805	-16.61764	17	3.0392	19.657	Zfp827
chr4	63079844	63079967	0.040982	-36.53703	15	6.4021	42.939	Zfp618
chr18	61753061	61753380	0.041015	-16.23110	17	2.8011	19.032	Afap1l1
chr5	147874697	14787487	0.041168	-30.76587	20	8.5833	39.349	Pomp
chr1	186967225	18696741	0.041349	-31.13119	15	7.4471	38.578	Gm37912 & DNA segment, Chr 1, Pasteur Institute 1
chr15	99627126	99627556	0.041378	-29.04061	17	0.73529	29.776	Racgap1
chr6	33044402	33044666	0.041463	-31.46825	12	0.83333	32.302	Chchd3
chr15	7885332	7886058	0.041463	-21.46307	23	2.8261	24.289	Wdr70
chr16	52822152	52822296	0.041521	-29.902	17	15.686	45.588	
chr13	34746910	34747036	0.041521	-13.79785	17	2.0289	15.827	Fam50b
chr19	58977116	58977517	0.041603	-29.36041	17	0.4902	29.851	Shtn1
chr2	135703400	13570378	0.041634	-22.11017	17	9.1503	31.261	Plcb4
chr3	55203785	55204355	0.041656	-38.05641	23	28.072	66.128	Sohlh2
chr4	117894581	11789501	0.041656	-32.29575	17	1.2255	33.521	Ipo13
chr9	118095785	11809632	0.041666	-28.03125	17	5.7353	33.767	Cmc1
chr1	1.31E+08	1.31E+08	0.041666	-27.9972	17	5.3922	33.389	Dyrk3
chr17	66135531	66136147	0.041666	-26.6387	17	2.9412	29.58	Ddx11
chr4	136936094	13693625	0.041666	-20.70961	17	10.889	31.599	Epha8
chr11	53548033	53548261	0.041669	-18.5352	17	5.1261	23.661	Sept8

chr11	55628793	55629191	0.04167	-30.1471	17	4.6078	34.755	
chr17	25391933	25392301	0.04167	-17.1948	17	3.3403	20.535	Cacna1h
chr12	76606415	76606496	0.04167	-14.33862	15	4.2063	18.545	Sptb
chr11	51486435	51487031	0.041687	-37.25490	17	2.549	39.804	Col23a1
chr9	56663472	56663851	0.041771	-22.22486	15	4.5	26.725	Lingo1
chr19	53053638	53053771	0.041819	-17.4444	15	2.6111	20.056	
chr8	4801108	4802118	0.041849	-45.8333	15	12.222	58.056	
chr8	1.23E+08	1.23E+08	0.041849	-19.8913	23	2.8675	22.759	Gm45894 & ankyrin repeat domain 11 & Gm20388
chr9	1.16E+08	1.16E+08	0.042359	-26.8056	12	2.0833	28.889	
chr12	1.07E+08	1.07E+08	0.042409	-38.1034	12	0.7955	38.899	
chr13	20016420	20016586	0.042443	-45.6944	12	0.8333	46.528	
chr2	93194885	93195284	0.042564	-38.67063	12	5.1587	43.829	Trp53i11
chr6	28418729	28418917	0.042768	-20.19716	12	0.84135	21.039	Gcc1
chr15	78454026	78454536	0.042926	-26.55754	12	9.375	35.933	Tmprss6
chr9	73035556	73036149	0.042953	-51.04166	12	3.4722	54.514	Pigb
chr17	23283090	23283427	0.042953	-48.3929	12	9.1766	57.569	
chr4	152506499	15250667[illegible]	0.042953	-46.04888	12	11.47	57.519	Nphp4
chr5	67402668	67403081	0.042953	-45.97222	12	1.3889	47.361	Bend4
chr10	99029781	99030155	0.042953	-45.4861	12	1.5278	47.014	
chr13	1.14E+08	1.14E+08	0.042953	-43.75	12	5.8333	49.583	
chr7	29825979	29826485	0.042953	-43.5795	12	3.851	47.431	Zfp790
chr11	96565007	96565387	0.042953	-43.1944	12	4.1667	47.361	Skap1
chr1	134322295	13432260[illegible]	0.042953	-42.79761	12	4.3981	47.196	Ppfia4
chr5	99395951	99396522	0.042953	-42.63888	12	4.1667	46.806	Rasgef1b
chr1	1.81E+08	1.81E+08	0.042953	-42.5099	12	0.8333	43.343	Pycr2
chr11	1.04E+08	1.04E+08	0.042953	-42.4239	12	5.7407	48.165	Wnt9b
chr18	53984269	53984491	0.042953	-41.56746	12	4.1667	45.734	Gm5507
chr6	137458566	13745932[illegible]	0.042953	-40.94907	12	0.92593	41.875	Ptpro
chr13	56699580	56699972	0.042953	-40.6019	12	0.8333	41.435	Gm27698
chr9	72431695	72431778	0.042953	-39.375	12	1.0417	40.417	
chr4	139440420	13944048[illegible]	0.042953	-39.27774	12	1.6534	40.931	Ubr4
chr10	1.17E+08	1.17E+08	0.042953	-39.2427	12	11.111	50.354	
chr17	7755223	7755411	0.042953	-37.88194	12	3.869	41.751	Fndc1
chr6	124665233	12466534[illegible]	0.042953	-37.84060	12	3.4127	41.253	Lpcat3
chr1	1.81E+08	1.81E+08	0.042953	-37.7778	12	0.6944	38.472	Parp1
chr2	179926404	17992727[illegible]	0.042953	-37.46527	12	2.2222	39.688	Taf4
chr11	72447573	72447842	0.042953	-37.03373	12	0.83333	37.867	Mybbp1a
chr4	1.35E+08	1.35E+08	0.042953	-36.7956	12	3.9683	40.764	Man1c1
chr17	34577049	34577558	0.042953	-36.64051	12	1.0417	37.682	Notch4
chr15	78433600	78433762	0.042953	-36.40046	12	9.375	45.775	Kctd17
chr1	1.8E+08	1.8E+08	0.042953	-36.25	12	6.25	42.5	
chr12	1.08E+08	1.08E+08	0.042953	-35.9358	12	1.875	37.811	
chr17	71806569	71806700	0.042953	-35.70868	12	2.5	38.209	Clip4
chr8	22703412	22703910	0.042953	-35.16188	12	0.37879	35.541	Ikbkb
chr12	84923536	84924177	0.042953	-35.06944	12	1.3889	36.458	Arel1
chr14	121690759	12169168[illegible]	0.042953	-35.03336	12	2.0833	37.117	Dock9
chr8	116624027	11662411[illegible]	0.042953	-34.94047	12	0.34722	35.288	Cdyl2
chr12	84744300	84745277	0.042953	-34.7222	12	4.8611	39.583	
chr6	1.09E+08	1.09E+08	0.042953	-34.6329	12	6.6667	41.3	
chr19	6318156	6318327	0.042953	-34.4771	12	4.4399	38.917	Cdc42bpg
chr4	1.28E+08	1.28E+08	0.042953	-34.3254	12	5.5804	39.906	
chr13	51482110	51482630	0.042953	-33.87896	12	1.0417	34.921	Shc3
chrX	69002873	69003087	0.042953	-33.8128	12	3.75	37.563	
chr5	23816762	23817198	0.042953	-33.43750	12	0.69444	34.132	Rint1 & 4933427G23Rik

chr12	82362949	82363073	0.042953	-33.3829	12	3.3333	36.716	Sipa1l1
chr4	135930304	13593081	0.042953	-33.22330	12	10.417	43.64	Fuca1
chr5	1.45E+08	1.45E+08	0.042953	-33.0258	12	2.6786	35.704	
chr1	1.62E+08	1.62E+08	0.042953	-32.6109	12	1.875	34.486	
chr4	106572249	10657234	0.042953	-32.46527	12	3.0903	35.556	Dhcr24
chr1	119527010	11952729	0.042953	-31.65238	12	7.6389	39.291	Tmem185b
chr10	81082000	81082037	0.042953	-31.54100	12	11.111	42.652	Thop1
chr5	73564260	73564368	0.042953	-31.1773	12	6.5774	37.755	
chr10	76433342	76433758	0.042953	-30.54563	12	6.1111	36.657	Pcnt
chr10	80008283	80008429	0.042953	-30.49768	12	2.0833	32.581	Abca7
chr11	66084706	66084980	0.042953	-30.31084	12	0.83333	31.144	Dnah9
chr10	1.19E+08	1.19E+08	0.042953	-30.1984	12	3.2738	33.472	
chr1	190161540	19016168	0.042953	-30.05787	12	1.1574	31.215	Prox1
chr4	1.54E+08	1.54E+08	0.042953	-29.9339	12	0.8333	30.767	
chr3	87995511	87995686	0.042953	-29.78174	12	5.5556	35.337	Bcan
chr5	139183624	13918383	0.042953	-29.73379	12	3.125	32.859	Dnaaf5
chr19	55154886	55155174	0.042953	-29.6528	12	4.1667	33.819	
chr11	116144023	11614441	0.042953	-29.48412	12	1.0417	30.526	Fbf1
chr6	113679763	11367999	0.042953	-29.16681	12	1.0417	30.208	Irak2
chr10	59871625	59871678	0.042953	-29.0278	12	0.4167	29.444	Gm7413
chr8	44687153	44687405	0.042953	-28.8194	12	4.1667	32.986	
chr5	1.29E+08	1.29E+08	4.30E-02	-28.7798	12	2.9167	31.696	Rimbp2
chr15	96649203	96649364	0.042953	-28.5384	12	14.54	43.079	
chr15	87876572	87876922	0.042953	-28.399	12	2.3313	30.73	
chr9	44692548	44693063	0.042953	-28.34325	12	2.2222	30.565	Phldb1
chr15	82203208	82203328	0.042953	-28.25396	12	2.619	30.873	Srebf2
chr12	1.15E+08	1.15E+08	0.042953	-28.0704	12	1.3889	29.459	
chr5	103834712	10383529	0.042953	-27.83234	12	3.5417	31.374	Aff1
chr11	72378458	72379129	0.042953	-27.5694	12	1.0417	28.611	
chr11	59249971	59250071	0.042953	-27.40064	12	5.0183	32.419	Wnt3a
chr18	11952042	11952479	0.042953	-27.3211	12	1.9481	29.269	
chr15	1.02E+08	1.02E+08	0.042953	-27.0602	12	0.9259	27.986	
chr14	60766562	60766948	0.042953	-26.6399	20	7.7917	34.432	
chr14	120798918	12079939	0.042953	-26.59035	12	5.3175	31.908	Gm25781
chr12	84681216	84681593	0.042953	-26.52777	12	7.2222	33.75	Syndig1l
chr7	139919743	13992006	0.042953	-26.51124	12	4.4444	30.956	Kndc1
chr11	5997285	5997455	0.042953	-25.69444	12	2.7778	28.472	Camk2b
chr17	5768274	5768710	0.042953	-25.2778	12	3.1994	28.477	
chr18	74700819	74701524	0.042953	-24.6729	12	4.2163	28.889	Myo5b
chr8	12471475	12471895	0.042953	-24.1468	12	0.6944	24.841	
chr4	154161772	15416196	0.042953	-23.67355	12	1.882	25.556	Wrap73
chr11	118758733	11875918	0.042953	-23.63905	12	5.3427	28.982	Rbfox3
chr4	135561147	13556159	0.042953	-23.57804	12	3.6706	27.249	Grhl3
chr17	28807017	28807473	0.042953	-23.24404	12	1.9841	25.228	Brpf3
chr7	143480419	14348050	0.042953	-23.10515	12	2.2222	25.327	Slc22a18
chr11	1.07E+08	1.07E+08	0.042953	-22.7083	12	4.1667	26.875	
chr7	127882432	12788256	0.042953	-22.65241	12	3.5119	26.164	Zfp646
chr2	26481514	26481831	0.042953	-22.62103	20	2.5089	25.13	Notch1
chr6	1.25E+08	1.25E+08	0.042953	-22.4653	12	2.0833	24.549	Pianp
chr10	1.18E+08	1.18E+08	0.042953	-22.1101	12	1.3889	23.499	
chr2	1.59E+08	1.59E+08	0.042953	-21.994	12	1.0417	23.036	Dhx35
chr5	37155657	37156096	0.042953	-21.83531	12	3.7202	25.556	Gm1043
chr4	41563291	41563465	0.042953	-21.54100	12	2.0833	23.624	Fam219a
chr15	91709902	91710054	0.042953	-21.2563	12	3.7536	25.01	Lrrk2
chr4	1.37E+08	1.37E+08	0.042953	-20.8614	12	3.6706	24.532	Epha8
chr18	36535422	36535582	0.042953	-20.2745	12	3.8889	24.163	Slc4a9
chr4	155328903	15532908	0.042953	-20.04629	12	4.6875	24.734	Prkcz
chr18	77995591	77995789	0.042953	-19.98511	12	0.52083	20.506	Epg5

chr12	111329867	11133000[illegible]	0.042953	-19.48195	12	7.9167	27.399	Cdc42bpb
chr4	131819399	13181952[illegible]	0.042953	-19.33722	12	2.9726	22.31	Ptpru
chr14	31162371	31162577	0.042953	-18.45978	12	4.1997	22.66	Stab1
chr5	113566314	11356637[illegible]	0.042953	-17.92989	12	2.0833	20.013	Wscd2
chr7	80338874	80339090	0.042953	-17.84692	12	3.9352	21.782	Unc45a
chr2	164215198	16421538[illegible]	0.042953	-16.98908	12	2.2222	19.211	Wfdc15b
chr5	1.15E+08	1.15E+08	0.042953	-16.3863	12	0.5208	16.907	2210016L21Rik
chr5	1.21E+08	1.21E+08	0.042953	-15.8069	20	3.7946	19.602	
chr11	97301565	97301721	0.042953	-15.7986	12	1.3889	17.188	
chr17	25381514	25381591	0.042953	-15.41305	12	15.341	30.754	Cacna1h
chr9	61020034	61020747	0.042953	-12.1063	12	2.381	14.487	
chr8	23234267	23235439	0.043441	-28.14236	24	3.8542	31.997	Gins4
chr5	115620011	11562020[illegible]	0.043481	-15.82994	18	2.3721	18.202	Gcn1l1
chr17	66379718	66379819	0.045057	-21.45189	14	4.6471	26.099	Mtcl1
chr16	90674472	90675233	0.045964	-41.89814	18	9.4907	51.389	AC160759.1
chr5	65064425	65065058	0.046033	-27.2332	18	4.6296	31.863	Tmem156
chr4	43029018	43029625	0.046033	-22.29055	14	5.5014	27.792	Stoml2
chrUn_	43767	45064	0.046094	-8.61987	26	10.921	19.541	
chr3	38455795	38455996	0.046421	-14.94841	25	1	15.948	Ankrd50
chr11	84897235	84897753	0.046565	-27.24316	18	5.0562	32.299	Myo19
chr14	35044256	35044826	0.046826	-27.47669	11	2.2727	29.749	Grid1
chr7	40388367	40388570	0.046879	-65.08333	10	2.25	67.333	Gm28807
chr12	94661917	94662155	0.046879	-62.3333	10	10	72.333	
chr3	135526587	13552666[illegible]	0.046879	-60.00000	10	2.5	62.5	Manba
chr3	85183697	85184205	0.046879	-59.14682	10	8.2143	67.361	Gm37240
chr12	110816070	11081616[illegible]	0.046879	-58.66666	10	1.6667	60.333	Mok
chr6	76496723	76496842	0.046879	-58.28125	16	4.2188	62.5	Gm9008 & Gm44166
chr10	11856248	11856277	0.046879	-57.9167	10	9.1667	67.083	
chr5	114953326	11495336[illegible]	0.046879	-57.44047	10	2.5	59.94	Hnf1a
chr16	97502214	97502413	0.046879	-56.91666	10	2.5	59.417	Fam3b
chr8	40016430	40016565	0.046879	-56.8333	10	1.6667	58.5	
chr9	1.03E+08	1.03E+08	0.046879	-52.9167	10	6.25	59.167	Ky
chr12	85306194	85306476	0.046879	-52.59523	10	3.2143	55.81	Nek9
chr11	91576755	91576866	0.046879	-52.57142	10	9.9286	62.5	Kif2b
chr5	136275089	13627516[illegible]	0.046879	-52.41666	10	2.5	54.917	Cux1 & Gm16599
chr10	1.19E+08	1.19E+08	0.046879	-52.25	10	10.417	62.667	
chr15	97758851	97759052	0.046879	-51.79166	10	1	52.792	Rapgef3
chr11	43388413	43388628	0.046879	-51.25	10	1.6667	52.917	
chr2	1.69E+08	1.69E+08	0.046879	-51.1905	10	2.5	53.69	
chr10	62222644	62222886	0.046879	-51.15476	10	2.8333	53.988	Tspan15
chr4	1.4E+08	1.4E+08	0.046879	-51.0238	10	1	52.024	
chr1	136070681	13607076[illegible]	0.046879	-50.45634	10	1.25	51.706	Cacna1s
chr16	23245436	23245627	0.046879	-49.97619	10	2.6667	52.643	St6gal1
chr4	117111004	11711122[illegible]	0.046879	-49.61767	10	4.9061	54.524	Ptch2
chr4	152466690	15246674[illegible]	0.046879	-49.58333	10	5.8333	55.417	Kcnab2
chr9	102886016	10288614[illegible]	0.046879	-49.42857	10	2.5	51.929	Ryk
chr1	1.27E+08	1.27E+08	0.046879	-49.0833	10	1	50.083	Mgat5
chr9	89008178	89008338	0.046879	-48.9405	10	30.643	79.583	
chr5	1.51E+08	1.51E+08	0.046879	-48.3611	10	1.5556	49.917	
chr1	118022428	11802255[illegible]	0.046879	-48.33333	10	36.667	85	Gm28358
chr18	4336250	4336529	0.046879	-48.19841	10	1.5476	49.746	Map3k8
chr17	84633498	84633596	0.046879	-48.08333	10	2.5	50.583	Dync2li1
chr5	88657039	88658014	0.046879	-48	10	1	49	
chr4	129476799	12947717[illegible]	0.046879	-47.66666	10	1.25	48.917	Bsdc1
chr10	21489196	21489651	0.046879	-47.5833	10	7.5	55.083	
chr2	165655618	16565622[illegible]	0.046879	-47.54761	10	1	48.548	Eya2
chr5	136859103	13685927[illegible]	0.046879	-47.50000	10	5	52.5	Col26a1

chr13	1.15E+08	1.15E+08	0.046879	-47.0833	10	3.75	50.833	
chr5	1.2E+08	1.2E+08	0.046879	-46.8333	10	1.25	48.083	
chr2	32266369	32266727	0.046879	-46.381	10	1	47.381	
chr5	125452604	125452697	0.046879	-45.97222	10	1.25	47.222	Bri3bp
chr7	1.42E+08	1.42E+08	0.046879	-45.8561	10	3.5	49.356	
chr4	155445763	155446149	0.046879	-45.16666	10	1.25	46.417	Cfap74
chr18	68119943	68120790	0.046879	-44.94642	10	2.0536	47	Ldlrad4
chr7	34843016	34843377	0.046879	-44.7976	10	0.9167	45.714	
chr2	156505895	156506206	0.046879	-44.67857	10	2.9167	47.595	Epb41l1
chr10	96450998	96451435	0.046879	-44.5833	10	3.75	48.333	
chr10	95015681	95015953	0.046879	-44.25	10	1.6667	45.917	
chr8	122683086	122683228	0.046879	-44.23809	10	2.4286	46.667	Gm20388 & CBFA2/RUNX1 translocation partner 3
chr4	124685278	124685781	0.046879	-44.16666	10	1	45.167	Utp11
chr12	111263715	111263814	0.046879	-44.14881	10	0.625	44.774	Traf3
chr3	83916336	83916487	0.046879	-44.1111	10	3.0556	47.167	Tmem131l
chr13	96088441	96088658	0.046879	-44.02525	10	4.4747	48.5	Sv2c
chr17	67632303	67632551	0.046879	-43.8333	10	2.5	46.333	Lrrc30
chr4	1.52E+08	1.52E+08	0.046879	-43.75	10	2.5	46.25	Camta1
chr4	57585849	57586262	0.046879	-43.6667	10	2.5	46.167	Palm2 & Pakap
chr17	63469610	63470150	0.046879	-43.4588	10	2.7143	46.173	Fbxl17
chr2	132515107	132515342	0.046879	-43.33333	10	1.25	44.583	1700026D11Rik
chrX	48397570	48398165	0.046879	-43.16666	10	1	44.167	Bcorl1
chr1	1.35E+08	1.35E+08	0.046879	-43.0087	10	7.7917	50.8	Nav1
chr3	89166909	89167068	0.046879	-43.00000	10	3.6667	46.667	Clk2
chr2	1.64E+08	1.64E+08	0.046879	-42.9419	11	1.2987	44.241	
chr16	34030532	34031210	0.046879	-42.69480	11	1.5152	44.21	Kalrn
chr9	65278511	65278676	0.046879	-42.6667	10	3.3333	46	Cilp
chr4	1.07E+08	1.07E+08	0.046879	-42.6071	10	1	43.607	
chr7	28221006	28221153	0.046879	-42.6042	16	27.708	70.313	
chr15	58418557	58419047	0.046879	-42.48214	10	5.1726	47.655	Fam91a1
chr10	1.28E+08	1.28E+08	0.046879	-42.3889	10	1.6667	44.056	Timeless
chr7	25824100	25824351	0.046879	-42.25	10	2	44.25	
chrX	1.47E+08	1.47E+08	0.046879	-42.1548	10	1.4286	43.583	
chr2	75593674	75593903	0.046879	-42.0694	10	2.5	44.569	
chr8	88021474	88021650	0.046879	-42	10	2.9167	44.917	
chr6	56966109	56966295	0.046879	-41.9821	10	8.1955	50.178	
chr7	98897461	98897969	0.046879	-41.91666	10	0.83333	42.75	Gm45188 & UV radiation resistance associated gene
chr17	24513230	24513321	0.046879	-41.66666	10	2.5	44.167	Traf7
chr4	1.39E+08	1.39E+08	0.046879	-41.619	10	1.25	42.869	Nbl1
chr10	121243561	121243667	0.046879	-41.18813	10	0.90909	42.097	Gm35404
chr11	1.18E+08	1.18E+08	0.046879	-40.9802	10	2.381	43.361	
chrX	48273087	48273392	0.046879	-40.83333	10	2.5	43.333	Utp14a
chr5	22706040	22706821	0.046879	-40.76190	10	1	41.762	6030443J06Rik
chr9	78388983	78389836	0.046879	-40.5087	11	1.1364	41.645	Gm8087
chr8	13112082	13112417	0.046879	-40.50000	10	6.25	46.75	Cul4a
chr10	79867021	79867241	0.046879	-40.25	10	2.5	42.75	Plppr3
chrX	11283506	11283780	0.046879	-40.1786	10	41.488	81.667	
chr5	1.34E+08	1.34E+08	0.046879	-40.1667	10	4.1667	44.333	Gtf2i
chr9	97980773	97981541	0.046879	-40.16666	10	0.83333	41	Clstn2
chr3	51690405	51690543	0.046879	-39.85714	10	1.6667	41.524	Maml3
chr8	47355974	47356252	0.046879	-39.69047	10	6	45.69	Stox2
chr8	79998627	79998833	0.046879	-39.5833	10	2.5	42.083	Hhip

chr10	88434453	88434990	0.046879	-39.50000	10	1.6667	41.167	Gnptab
chr5	1.15E+08	1.15E+08	0.046879	-39.5	10	2	41.5	
chr8	1.25E+08	1.25E+08	0.046879	-39.4643	10	3	42.464	Disc1
chr12	1.07E+08	1.07E+08	0.046879	-39.4524	10	2.6667	42.119	
chr11	103076028	10307639[illegible]	0.046879	-39.33333	10	1.6667	41	Plcd3
chr11	94566977	94567027	0.046879	-39.27579	10	0.625	39.901	Acsf2 & Chad
chr2	1.68E+08	1.68E+08	0.046879	-39.2381	10	1.25	40.488	
chr11	64071623	64071882	0.046879	-39.16666	16	7.2917	46.458	Cox10
chr2	118718269	11871882[illegible]	0.046879	-39.16666	10	1.25	40.417	Plcb2
chr2	1.73E+08	1.73E+08	0.046879	-39.0833	10	2.5	41.583	
chr4	33225916	33226120	0.046879	-39.02777	10	3.8889	42.917	Srsf12
chr16	93807318	93807786	0.046879	-38.97420	10	2	40.974	Dop1b
chr8	110592272	11059264[illegible]	0.046879	-38.68284	10	3.8846	42.567	Hydin
chr10	99595411	99595685	0.046879	-38.6607	14	18.929	57.589	
chr17	25970350	25970750	0.046879	-38.62481	10	5	43.625	Capn15
chr8	13001660	13001826	0.046879	-38.56196	10	5.5833	44.145	Mcf2l
chr6	49957952	49958501	0.046879	-38.4686	11	2.2727	40.741	
chr11	97514605	97515257	0.046879	-37.95238	10	0.71429	38.667	Srcin1
chr4	135497511	13549778[illegible]	0.046879	-37.91666	10	6	43.917	Stpg1
chr8	22678881	22679135	0.046879	-37.9107	10	3.3929	41.304	Ikbkb
chr10	1.18E+08	1.18E+08	0.046879	-37.7662	10	4.4084	42.175	
chr5	149568429	14956868[illegible]	0.046879	-37.75000	10	10.583	48.333	Wdr95
chr9	1.03E+08	1.03E+08	0.046879	-37.75	10	1.75	39.5	
chr2	59726033	59726351	0.046879	-37.5833	10	1.25	38.833	Tanc1
chr7	142492185	14249241[illegible]	0.046879	-37.47321	10	2.2619	39.735	Prr33 & Lsp1
chr17	56352539	56352612	0.046879	-37.45951	10	13.621	51.081	Kdm4b
chr17	72388730	72389033	0.046879	-37.37103	10	5.125	42.496	Alk
chr5	124120118	12412051[illegible]	0.046879	-37.34722	10	4.3333	41.681	Pitpnm2
chr16	14391395	14392048	0.046879	-37.26190	10	3	40.262	Abcc1
chr4	117145390	11714563[illegible]	0.046879	-37.24404	10	1.6667	38.911	Best4-ps
chr4	79142986	79143366	0.046879	-36.8333	10	2.5	39.333	
chr2	1.75E+08	1.75E+08	0.046879	-36.7857	10	2.7976	39.583	
chr10	33178817	33179106	0.046879	-36.50000	10	4.1667	40.667	Trdn
chr16	38326556	38326664	0.046879	-36.50000	10	7.25	43.75	Maats1
chr2	94682528	94682971	0.046879	-36.4702	10	1.4286	37.899	
chr10	108356690	10835688[illegible]	0.046879	-36.41666	10	0.83333	37.25	Pawr
chr2	1.68E+08	1.68E+08	0.046879	-36.377	10	2.5	38.877	
chr1	74191589	74191926	0.046879	-36.3636	11	2.6515	39.015	Cxcr1
chrX	1.23E+08	1.23E+08	0.046879	-36.3333	10	1.6667	38	Cldn34c1
chr17	24585485	24585797	0.046879	-36.25000	10	2.5	38.75	Pkd1
chr7	30173693	30174195	0.046879	-36.1845	10	7.75	43.935	Gm5113
chr4	1.4E+08	1.4E+08	0.046879	-36.1667	10	5	41.167	Igsf21
chr7	1.03E+08	1.03E+08	0.046879	-36.131	10	5.1667	41.298	Trim68
chr7	28174514	28174811	0.046879	-35.97420	10	2.6667	38.641	Fbl
chr13	98161038	98161130	0.046879	-35.97222	10	2.5	38.472	Arhgef28
chr7	1.44E+08	1.44E+08	0.046879	-35.9345	10	2.6667	38.601	
chr11	106543572	10654375[illegible]	0.046879	-35.73214	10	2.1032	37.835	Tex2
chr2	1.63E+08	1.63E+08	0.046879	-35.6925	10	9.4742	45.167	
chr10	94587023	94587223	0.046879	-35.5824	16	11.293	46.875	Tmcc3os & transmembrane and coiled coil domains 3
chr2	127238257	12723865[illegible]	0.046879	-35.53084	10	1	36.531	Snrnp200
chr12	98679234	98679459	0.046879	-35.48484	10	2.5	37.985	Ptpn21
chr11	119857915	11985807[illegible]	0.046879	-35.47222	10	4.1667	39.639	Rptor
chr7	110282058	11028280[illegible]	0.046879	-35.42099	10	2.5	37.921	Swap70
chr15	79963341	79963893	0.046879	-35.3452	10	1.25	36.595	Cbx7
chr11	113834680	11383493[illegible]	0.046879	-35.33549	10	5.5833	40.919	Sdk2

chr10	76772247	76772809	0.046879	-35.16666	10	2.9643	38.131	Pcbp3
chr2	155810319	15581039	0.046879	-35.09523	10	1.25	36.345	Mmp24
chr15	59323582	59324200	0.046879	-35.0833	10	1.25	36.333	Sqle
chr11	88845969	88846167	0.046879	-35.02777	10	1.25	36.278	Akap1
chr14	30096214	30096438	0.046879	-35.00000	10	2.6667	37.667	Cacna1d
chr4	1.35E+08	1.35E+08	0.046879	-34.9708	10	3	37.971	
chr15	78705530	78705854	0.046879	-34.87500	10	1.25	36.125	Elfn2
chr18	19130382	19130600	0.046879	-34.7857	10	9.1786	43.964	
chr2	28548389	28548681	0.046879	-34.72420	10	5.6786	40.403	Ralgds
chr8	88305843	88306454	0.046879	-34.66010	10	0.55556	35.216	Adcy7
chr10	77054812	77054939	0.046879	-34.65187	11	3.4091	38.061	Slc19a1 &
chr15	79126933	79127051	0.046879	-34.51190	10	7.5	42.012	Micall1
chr14	34036744	34037058	0.046879	-34.44047	10	5	39.44	Gm5460
chr8	114972080	11497251	0.046879	-34.38888	10	11.833	46.222	Wwox
chr4	117876927	11787695	0.046879	-34.33333	10	5	39.333	B4galt2
chr14	32919119	32919290	0.046879	-34.25	10	5	39.25	Vstm4
chr2	1.61E+08	1.61E+08	0.046879	-34.2262	10	5	39.226	
chr1	92403835	92404353	0.046879	-34.2053	10	3.8095	38.015	
chr2	1.79E+08	1.79E+08	0.046879	-34.1468	10	2	36.147	
chr4	1.36E+08	1.36E+08	0.046879	-34.125	10	6.25	40.375	Asap3
chr5	62777540	62778024	0.046879	-34.0909	11	2.2727	36.364	
chr5	115444956	11544517	0.046879	-33.89484	10	0.71429	34.609	Msi1
chr7	118434121	11843417	0.046879	-33.82341	10	13.333	47.157	Syt17
chr15	84647234	84647604	0.046879	-33.7976	10	1	34.798	
chr2	31090048	31090375	0.046879	-33.75000	10	1.6667	35.417	Fnbp1
chr11	116118927	11611916	0.046879	-33.50000	10	1	34.5	Trim47
chr18	73954291	73954600	0.046879	-33.46428	10	1	34.464	Mapk4
chr8	105779712	10577993	0.046879	-33.45238	10	4.2143	37.667	Ranbp10
chr16	91917178	91917623	0.046879	-33.3333	10	3.0833	36.417	Atp5o & Itsn1
chr2	165380992	16538126	0.046879	-33.31011	16	4.0104	37.321	Zfp334
chr11	33283908	33284765	0.046879	-33.26190	10	3.3333	36.595	Ranbp17
chr1	72399045	72399198	0.046879	-33.131	10	1.6667	34.798	
chr2	5699740	5700379	0.046879	-33.03030	10	3.6667	36.697	Camk1d
chr7	126492471	12649268	0.046879	-32.91666	10	1.6667	34.583	Atxn2l
chr11	43380015	43380460	0.046879	-32.8734	11	2.9221	35.795	
chr4	148594169	14859453	0.046879	-32.60101	11	8.5354	41.136	Srm
chr4	47836668	47837118	0.046879	-32.56818	10	2.2917	34.86	Gm38343
chr8	109550536	10955080	0.046879	-32.55952	10	3.4286	35.988	Dhx38
chr5	137666427	13766664	0.046879	-32.54761	10	2.0833	34.631	Agfg2
chr4	151346426	15134676	0.046879	-32.52232	16	2.5	35.022	Camta1
chr3	1.35E+08	1.35E+08	0.046879	-32.5	10	2.5	35	
chr13	55550159	55550565	0.046879	-32.4603	10	5.373	37.833	Fam193b
chr11	1.09E+08	1.09E+08	0.046879	-32.4098	22	6.9859	39.396	Axin2
chr4	152308791	15230887	0.046879	-32.39682	10	3.7222	36.119	Rnf207
chr3	89443508	89443688	0.046879	-32.35119	14	3.7202	36.071	Pbxip1
chr10	120813563	12081399	0.046879	-32.32684	11	6.8182	39.145	Msrb3
chr4	82884618	82884778	0.046879	-32.2619	10	2.5	34.762	Cer1
chr4	126593535	12659400	0.046879	-32.16666	10	6.2619	38.429	Clspn
chr7	1.14E+08	1.14E+08	0.046879	-32.0991	10	1.25	33.349	Rras2
chr1	120012534	12001283	0.046879	-31.89059	14	0.5102	32.401	Sctr
chr5	15644117	15644671	0.046879	-31.88131	11	2.0202	33.902	Gm21847
chr4	1.32E+08	1.32E+08	0.046879	-31.8333	10	4.1667	36	
chr3	44024286	44024510	0.046879	-31.8214	10	11.917	43.738	
chr11	88088412	88089115	0.046879	-31.7778	10	1.6667	33.444	
chr8	125803156	12580330	0.046879	-31.70238	10	8.5714	40.274	Pcnx2
chr8	1.15E+08	1.15E+08	0.046879	-31.6922	14	4.4388	36.131	Wwox
chr10	80130774	80130852	0.046879	-31.45833	10	2.0833	33.542	Cbarp
chr17	56784700	56784784	0.046879	-31.41071	10	2.5476	33.958	Rfx2

chr17	49587515	49587876	0.046879	-31.4048	10	4.4286	35.833	
chr9	45439345	45439647	0.046879	-31.39285	10	1.25	32.643	Dscaml1
chr17	25855694	25855849	0.046879	-31.21018	14	5.1763	36.386	Wdr90 & Gm26694
chr9	60197492	60197693	0.046879	-31.17857	10	3.2619	34.44	Thsd4
chr5	1.14E+08	1.14E+08	0.046879	-31.0556	10	8.75	39.806	Ssh1
chr2	1.73E+08	1.73E+08	0.046879	-31.0476	10	2.5	33.548	
chr1	134743834	134743986	0.046879	-30.93452	10	2.381	33.315	Syt2
chr9	83720499	83720873	0.046879	-30.9107	10	3.006	33.917	
chr15	76070800	76070970	0.046879	-30.84172	10	1.25	32.092	Puf60
chr4	132498920	132499172	0.046879	-30.78769	10	4.5833	35.371	Sesn2
chr5	139455517	139455639	0.046879	-30.78571	10	1	31.786	3110082I17Rik
chr3	79821517	79821740	0.046879	-30.77777	10	1.25	32.028	Tmem144
chr19	47655004	47655108	0.046879	-30.76190	10	4.75	35.512	Col17a1
chr11	1.04E+08	1.04E+08	0.046879	-30.7608	10	1.6667	32.427	Mapt
chr9	120958803	120959838	0.046879	-30.75320	16	5.1215	35.875	Ctnnb1 & Ulk4
chr13	55526301	55526802	0.046879	-30.6955	10	1.25	31.946	Dok3
chr2	103732010	103732364	0.046879	-30.56944	10	5	35.569	Nat10
chr3	95263917	95264640	0.046879	-30.55036	10	2.5	33.05	Prune1
chr2	128191659	128192232	0.046879	-30.54166	10	5.375	35.917	Gm14009 & Gm14005
chr2	166074618	166074994	0.046879	-30.53174	10	1.6667	32.198	Sulf2
chr15	73092903	73093514	0.046879	-30.47619	10	3.4286	33.905	Chrac1
chr11	113279126	113279392	0.046879	-30.33333	10	4.5238	34.857	Slc39a11
chrX	7592883	7592993	0.046879	-30.30357	10	0.625	30.929	Foxp3 & Gm36995
chr17	73574813	73574927	0.046879	-30.29166	10	3.5119	33.804	Galnt14
chr7	45348533	45348750	0.046879	-30.28645	16	1.6667	31.953	Ppfia3
chr7	127193815	127193929	0.046879	-30.26190	10	1.6667	31.929	Cd2bp2
chr7	29343227	29343339	0.046879	-30.2262	10	0.8333	31.06	Sipa1l3
chr9	1.21E+08	1.21E+08	0.046879	-30.1964	10	1.25	31.446	
chr19	46642582	46642719	0.046879	-30.1389	10	2.5	32.639	Wbp1l
chr9	1.2E+08	1.2E+08	0.046879	-30.0972	10	2.625	32.722	
chr17	66586077	66586303	0.046879	-30.00000	10	5	35	Themis3
chr8	21993128	21993537	0.046879	-30.00000	10	2.5	32.5	Atp7b
chr2	35712960	35713407	0.046879	-29.9603	10	1.1111	31.071	Dab2ip
chr7	30581656	30581676	0.046879	-29.91666	10	6.25	36.167	Kmt2b
chr5	142749794	142750217	0.046879	-29.90336	10	2.8407	32.744	Tnrc18
chr8	104594458	104594573	0.046879	-29.79166	10	1.6667	31.458	Pdp2
chr8	11518466	11518772	0.046879	-29.77777	10	1.6667	31.444	Cars2
chr10	75638832	75639045	0.046879	-29.72402	10	1.5855	31.31	Susd2
chr18	38603468	38604021	0.046879	-29.71428	10	2.5	32.214	Arhgap26
chr10	119211462	119211526	0.046879	-29.66666	10	1.25	30.917	Cand1
chr1	1.34E+08	1.34E+08	0.046879	-29.5754	10	1.8948	31.47	
chr14	70339914	70340278	0.046879	-29.56818	10	5	34.568	Slc39a14
chr1	36781122	36781199	0.046879	-29.54491	10	4.9015	34.446	Zap70
chr10	80241150	80241301	0.046879	-29.53968	10	6.6667	36.206	Mum1
chr12	55564919	55565230	0.046879	-29.513	11	4.5455	34.058	
chr12	84920002	84920306	0.046879	-29.44047	10	0.71429	30.155	Arel1
chr5	112425596	112425779	0.046879	-29.36507	10	1.6667	31.032	Sez6l
chr10	81499436	81499520	0.046879	-29.30096	11	3.5021	32.803	S1pr4
chr3	62817116	62817535	0.046879	-29.1667	10	1.25	30.417	
chr11	1.03E+08	1.03E+08	0.046879	-29.119	10	0.7143	29.833	
chr8	85081666	85081962	0.046879	-29.08854	16	3.125	32.214	Wdr83os
chr8	121487675	121487903	0.046879	-29.05952	10	1.6667	30.726	Gm20388
chr19	44850217	44850468	0.046879	-29.0359	10	4.095	33.131	
chr17	24674037	24674476	0.046879	-28.98015	10	3.381	32.361	Zfp598

chr16	17877049	17877365	0.046879	-28.94047	10	1.6667	30.607	Gm20518 & DiGeorge syndrome critical region gene 2
chr4	150164624	150165195	0.046879	-28.75000	10	1.25	30	Slc2a7
chr7	1.39E+08	1.39E+08	0.046879	-28.6667	10	8.0833	36.75	4930543N07Rik
chr8	14532798	14533358	0.046879	-28.66666	10	0.83333	29.5	Dlgap2
chr18	84208841	84209019	0.046879	-28.64682	10	1.7143	30.361	Zfp407
chr11	118519355	118519637	0.046879	-28.55952	10	1.5476	30.107	Rbfox3
chr6	113948459	113948819	0.046879	-28.54166	10	3.5417	32.083	Atp2b2
chr13	55936426	55937299	0.046879	-28.4647	16	3.4959	31.961	
chr17	8981799	8982033	0.046879	-28.4643	10	1.25	29.714	Pde10a
chr7	28616800	28617132	0.046879	-28.30086	11	1.1364	29.437	Acp7
chr1	128286341	128286579	0.046879	-28.29166	10	3.125	31.417	Lct
chr15	94001325	94001749	0.046879	-28.2738	14	2.6786	30.952	
chr9	1.08E+08	1.08E+08	0.046879	-28.1618	10	5.2976	33.459	Rnf123 & Gm20529
chr14	25524199	25524366	0.046879	-28.01641	10	1.6667	29.683	Zmiz1
chr4	14290709	14291081	0.046879	-27.9821	10	5	32.982	
chr3	1.52E+08	1.52E+08	0.046879	-27.9583	10	3.125	31.083	
chr16	31548185	31548604	0.046879	-27.75	10	5	32.75	
chr5	1.14E+08	1.14E+08	0.046879	-27.6541	16	1.2277	28.882	Mvk
chr11	55495299	55495411	0.046879	-27.58928	10	3.125	30.714	G3bp1
chr12	36372365	36373184	0.046879	-27.4246	10	3.75	31.175	
chr19	6375521	6375625	0.046879	-27.4048	10	11.524	38.929	Sf1
chr7	126947083	126947622	0.046879	-27.40277	10	0.625	28.028	Asphd1 & Gm21984
chr1	91499924	91500380	0.046879	-27.39285	10	1.25	28.643	Traf3ip1
chr17	87346162	87346349	0.046879	-27.3214	10	5.3571	32.679	Ttc7
chr19	43043320	43043536	0.046879	-27.2262	10	5	32.226	Hpse2
chr8	106264584	106264749	0.046879	-27.21428	10	0.83333	28.048	Gm26786 & sphingomyelin phosphodiesterase 3, neutral
chr10	4130197	4130465	0.046879	-27.12698	10	3.0754	30.202	Mthfd1l
chr11	52662065	52662341	0.046879	-27.1131	14	0.4464	27.56	
chr6	1.44E+08	1.44E+08	0.046879	-27.0876	14	0.4464	27.534	Sox5
chr19	58809501	58809596	0.046879	-27.0119	10	2.5	29.512	Hspa12a
chr13	38694502	38694789	0.046879	-27	10	4.1667	31.167	
chr14	70117359	70117997	0.046879	-26.99724	11	2.2727	29.27	Bin3
chr9	45942668	45942822	0.046879	-26.95436	10	2.5	29.454	Sidt2
chr9	44580294	44580786	0.046879	-26.75	10	1.25	28	Gm47230
chr3	124291885	124292295	0.046879	-26.61904	10	1.25	27.869	Gm9372 & Gm43731
chr7	99717821	99718057	0.046879	-26.5714	10	1.25	27.821	
chr2	167611058	167611670	0.046879	-26.56318	10	1.6667	28.23	Ube2v1 & Gm20431
chr16	92106583	92106662	0.046879	-26.54365	10	3.4722	30.016	Mrps6 & AC144408.3
chr7	122006344	122006676	0.046879	-26.54155	11	1.1364	27.678	Gga2
chr8	117605347	117606060	0.046879	-26.48122	26	4.9061	31.387	Plcg2
chr15	81008240	81008360	0.046879	-26.34859	10	6.6667	33.015	Sgsm3
chr7	107731054	107731508	0.046879	-26.30952	10	6.6667	32.976	Ppfibp2
chr9	120057308	120057513	0.046879	-26.23809	10	1	27.238	Cx3cr1
chr11	118482777	118482946	0.046879	-26.22096	10	1.1111	27.332	Engase
chrX	99321849	99322233	0.046879	-26.2024	10	5.4762	31.679	
chr2	168636006	168636326	0.046879	-26.16305	10	5.4545	31.618	Atp9a
chr2	26392525	26392633	0.046879	-26.15476	10	0.83333	26.988	Pmpca

chr2	31420179	31420352	0.046879	-26.15046	11	2.8409	28.991	Hmcn2
chr10	62967293	62967526	0.046879	-26.1369	10	1.25	27.387	Dna2
chr8	88918393	88918689	0.046879	-26.0833	10	6	32.083	
chr7	122625928	122626740	0.046879	-26.04166	16	5.2083	31.25	Prkcb
chr5	37112895	37113011	0.046879	-25.9286	10	4.7619	30.69	Jakmip1 & Gm1043
chr12	4682147	4682581	0.046879	-25.78968	10	1.25	27.04	Itsn2
chr11	103345332	103345693	0.046879	-25.76221	10	3.4045	29.167	Arhgap27os3 & Arhgap27
chr11	120064863	120065200	0.046879	-25.76082	10	0.71429	26.475	Cep131
chr5	108332688	108333332	0.046879	-25.72817	10	0.625	26.353	Pigg
chr4	1.32E+08	1.32E+08	0.046879	-25.7224	22	4.2424	29.965	Ptpru
chr5	113764022	113764167	0.046879	-25.64285	10	6.7857	32.429	Sart3
chr18	69564544	69564773	0.046879	-25.631	10	1	26.631	Tcf4
chr11	78897252	78897697	0.046879	-25.6111	10	2.2619	27.873	
chr4	1.08E+08	1.08E+08	0.046879	-25.5736	11	2.2727	27.846	Lrp8
chr3	153349918	153350015	0.046879	-25.55952	10	3.3333	28.893	St6galnac3
chr8	109557755	109557938	0.046879	-25.49873	10	5	30.499	Dhx38
chr13	92757999	92758031	0.046879	-25.27777	10	5.5556	30.833	Thbs4
chr9	108849428	108849567	0.046879	-25.26599	10	3.2697	28.536	Celsr3
chr8	13668670	13668945	0.046879	-25.20833	10	0.83333	26.042	Rasa3
chr18	69506861	69507160	0.046879	-25.16666	10	5	30.167	Tcf4
chr10	70169288	70169566	0.046879	-25.15331	11	12.626	37.78	Ccdc6
chr15	74958296	74958578	0.046879	-25.05657	11	1.9751	27.032	Ly6e
chr3	144447084	144447239	0.046879	-25.04761	10	1.25	26.298	Hs2st1
chr5	1.24E+08	1.24E+08	0.046879	-25.0437	10	0.4167	25.46	Pitpnm2
chr7	126445125	126445264	0.046879	-25.02597	10	1.4286	26.455	Rabep2
chr10	1.11E+08	1.11E+08	0.046879	-24.9762	10	10	34.976	E2f7
chr17	25744604	25744792	0.046879	-24.93686	10	1.4583	26.395	Mslnl
chr15	95228914	95229072	0.046879	-24.93213	16	2.0833	27.015	Nell2
chr11	29069697	29069886	0.046879	-24.9047	10	2.9167	27.821	
chr15	79373494	79373853	0.046879	-24.88095	10	0.83333	25.714	Tmem184b
chr5	140463343	140463748	0.046879	-24.83820	10	4.9286	29.767	Gm4869
chr17	86700021	86700661	0.046879	-24.7848	10	0.8333	25.618	
chr8	111628525	111628625	0.046879	-24.75198	14	3.9087	28.661	Ldhd
chr7	34245025	34245619	0.046879	-24.62585	14	2.8316	27.457	4931406P16Rik
chr9	21093223	21093685	0.046879	-24.601	18	6.6667	31.268	1700084C06Rik
chr11	101599022	101599699	0.046879	-24.52448	16	4.0451	28.57	Gm11634
chr4	137715910	137716095	0.046879	-24.51959	10	2.5	27.02	Rap1gap
chr2	151858178	151858632	0.046879	-24.43650	10	1.6667	26.103	Rspo4
chr11	61703284	61703457	0.046879	-24.41666	10	5	29.417	Slc5a10 & Fam83g
chr6	39466721	39466945	0.046879	-24.41450	10	5	29.415	Dennd2a
chr7	67693043	67693282	0.046879	-24.36904	10	1.25	25.619	Ttc23
chr19	6493294	6493664	0.046879	-24.3344	11	0.9091	25.244	Nrxn2
chr7	141400680	141401128	0.046879	-24.32142	10	2.9167	27.238	Taldo1
chr15	58219868	58219980	0.046879	-24.18452	10	1.6667	25.851	Gm15943
chrX	161718145	161718434	0.046879	-24.17857	10	2.9167	27.095	Rai2
chr15	84639387	84639604	0.046879	-24.1647	10	0.8333	24.998	
chr2	1.51E+08	1.51E+08	0.046879	-24.125	10	1.6667	25.792	Pygb
chr10	127333578	127333729	0.046879	-23.87682	16	0.96726	24.844	Gli1 & Gm47200
chr4	119255773	119256640	0.046879	-23.83297	22	7.9924	31.825	Gm12927 & Cldn19
chr15	87555915	87556181	0.046879	-23.78826	14	1.3138	25.102	Fam19a5
chr15	76105939	76106542	0.046879	-23.57978	19	1.2427	24.822	Eppk1
chr5	143268793	143269040	0.046879	-23.57539	10	2.5	26.075	Zfp316
chr13	12499850	12499986	0.046879	-23.50000	10	7	30.5	Edaradd
chr7	29023520	29023757	0.046879	-23.49422	10	1.3889	24.883	Ryr1
chr17	25491485	25491588	0.046879	-23.3126	10	1.25	24.563	Sstr5

chr7	16218655	16218840	0.046879	-23.28571	10	0.71429	24	Dhx34
chr11	88348907	88348943	0.046879	-23.2361	10	5.6944	28.931	Msi2
chr8	95095391	95095815	0.046879	-23.1526	26	6.3721	29.525	Katnb1
chrX	9572021	9572639	0.046879	-23.10119	10	0.625	23.726	H2al1o
chr11	121513605	121514217	0.046879	-23.07837	10	0.55556	23.634	Zfp750 & Tbcd
chr10	116448010	116448383	0.046879	-23.03571	10	0.71429	23.75	Kcnmb4
chr10	40999589	40999790	0.046879	-22.8869	10	3.3333	26.22	
chr17	28155529	28155801	0.046879	-22.8269	10	7.113	29.94	Scube3
chr12	85221450	85221651	0.046879	-22.78210	10	1.2879	24.07	Eif2b2
chr10	8488731	8489589	0.046879	-22.75	10	1.6667	24.417	Ust
chr7	25225805	25225909	0.046879	-22.7341	10	0.8333	23.567	Zfp526
chr11	32067398	32067832	0.046879	-22.6786	10	6.25	28.929	
chr4	154581103	154581283	0.046879	-22.64285	10	1.25	23.893	Prdm16
chrX	139808832	139809041	0.046879	-22.55808	10	1.4286	23.987	Cldn2
chr10	41629961	41630094	0.046879	-22.4874	11	0.9091	23.396	Ccdc162
chr19	56905460	56906394	0.046879	-22.31241	22	1.7893	24.102	Vwa2
chr18	54899047	54899306	0.046879	-22.30663	10	4.3452	26.652	Zfp608
chr12	1.08E+08	1.08E+08	0.046879	-22.1478	16	1.5625	23.71	Bcl11b
chr4	1.25E+08	1.25E+08	0.046879	-22.1429	10	3.2143	25.357	Epha10
chr16	10417538	10418236	0.046879	-22.097	16	0.8929	22.99	Nubp1
chr5	112160914	112161361	0.046879	-21.98809	10	1.4286	23.417	1700016B01Rik
chr9	58578624	58579712	0.046879	-21.8939	11	4.5455	26.439	
chr10	56388488	56388655	0.046879	-21.83134	10	4.2361	26.067	Gja1
chr7	19400616	19401211	0.046879	-21.83106	14	5.6293	27.46	Klc3
chr9	21402663	21402904	0.046879	-21.63385	10	1.6667	23.301	Ilf3
chr4	1.41E+08	1.41E+08	0.046879	-21.6006	11	1.2987	22.899	Hspb7
chr2	27227052	27227214	0.046879	-21.53113	10	1.6667	23.198	Sardh & Sardhos
chr19	23508163	23508478	0.046879	-21.5238	10	2.5	24.024	
chr11	94988088	94988253	0.046879	-21.5159	10	1.25	22.766	
chr10	58738216	58738907	0.046879	-21.4918	16	3.3854	24.877	
chr11	113869836	113870500	0.046879	-21.43939	11	3.7879	25.227	Sdk2
chr3	146438758	146438941	0.046879	-21.41666	10	6.0833	27.5	Ssx2ip
chr1	189798790	189799306	0.046879	-21.26190	20	3.5833	24.845	Ptpn14
chr9	120018910	120019092	0.046879	-21.25992	16	6.653	27.913	Xirp1 & Cx3cr1
chr2	167996149	167996287	0.046879	-21.25595	10	1.6667	22.923	Ripor3
chr19	5672786	5673281	0.046879	-21.16883	11	8.1061	29.275	Pcnx3
chr14	69877909	69878004	0.046879	-21.10714	10	1	22.107	Pebp4
chr5	32944032	32944929	0.046879	-21.05615	10	6	27.056	Depdc5
chr8	110730059	110730186	0.046879	-20.91071	10	2.5	23.411	Mtss1l
chr6	99006984	99007298	0.046879	-20.87719	19	1.3158	22.193	Foxp1
chr2	6440640	6440988	0.046879	-20.7875	16	1.5625	22.35	Usp6nl
chr5	1.13E+08	1.13E+08	0.046879	-20.621	10	5	25.621	
chr18	82518109	82518157	0.046879	-20.61309	10	1.6667	22.28	Mbp
chr2	26992960	26993283	0.046879	-20.5617	10	2.1726	22.734	Adamts13
chr8	124564597	124564917	0.046879	-20.51785	10	1.9643	22.482	Agt
chr4	141903759	141903998	0.046879	-20.49007	10	2.4567	22.947	Fhad1
chr5	111753054	111753216	0.046879	-20.41666	10	2.5	22.917	E130006D01Rik
chr7	3181186	3181266	0.046879	-20.41161	10	4.7551	25.167	Gm3375
chr8	72686928	72687193	0.046879	-20.30257	10	3.5417	23.844	Nwd1
chr7	143390459	143390615	0.046879	-20.18650	10	4.0556	24.242	Kcnq1
chr15	86126493	86126881	0.046879	-20.1605	11	1.3384	21.499	Gramd4
chr11	59080117	59080180	0.046879	-19.996	10	9.1667	29.163	Obscn
chr17	32135393	32135896	0.046879	-19.94791	16	1.0417	20.99	Gm17276 & Notch3
chr10	99274978	99275271	0.046879	-19.91666	10	1.25	21.167	Gm48089
chr9	78704570	78705084	0.046879	-19.65999	22	3.8817	23.542	Cd109
chr2	33242094	33243137	0.046879	-19.56259	22	1.2121	20.775	Angptl2 &
chr10	43510043	43510446	0.046879	-19.5605	27	4.4496	24.01	Bend3

chr2	173158399	173158583	0.046879	-19.54573	19	1.9841	21.53	Pck1
chr1	84912655	84913337	0.046879	-19.40886	29	2.1689	21.578	Slc16a14
chr11	82370015	82370292	0.046879	-19.4048	10	3.5	22.905	
chr2	118873234	118873428	0.046879	-19.37500	10	3.3333	22.708	Ivd
chr1	1.61E+08	1.61E+08	0.046879	-19.1667	10	2.5	21.667	Zbtb37
chr8	1.2E+08	1.2E+08	0.046879	-19.1593	16	1.2277	20.387	Gse1 & Gm20388
chr5	110602921	110603414	0.046879	-19.02381	10	1.7361	20.76	Galnt9
chr2	93533369	93533612	0.046879	-18.9131	10	1.6667	20.58	
chr2	156489064	156489606	0.046879	-18.87500	10	6.4583	25.333	Epb41l1
chr4	42956741	42956963	0.046879	-18.84800	26	7.9227	26.771	Dnajb5
chr15	77995104	77995184	0.046879	-18.79670	10	2.5	21.297	Cacng2
chr17	14195901	14195957	0.046879	-18.75595	10	1.4286	20.185	Dact2
chr16	18950916	18951514	0.046879	-18.69446	11	0.90909	19.604	Hira & Gm28539
chr9	78042879	78043667	0.046879	-18.2187	27	5.787	24.006	
chr8	95709212	95709549	0.046879	-18.16575	10	1.6111	19.777	Ndrg4
chr10	22696235	22696369	0.046879	-17.90476	10	1	18.905	Slc2a12
chr11	4737808	4737946	0.046879	-17.8694	14	1.1054	18.975	Cabp7
chr10	81315979	81316167	0.046879	-17.82539	10	1	18.825	Pip5k1c
chr18	56739565	56739921	0.046879	-17.6448	10	7.3571	25.002	Lmnb1
chr15	25920927	25921487	0.046879	-17.64224	10	3.2143	20.857	Retreg1
chr11	1.03E+08	1.03E+08	0.046879	-17.6154	10	0.5556	18.171	
chr16	94267880	94267948	0.046879	-17.53156	10	0.45455	17.986	Hlcs
chr9	106223928	106224158	0.046879	-17.44083	10	0.625	18.066	Tlr9
chr9	21438467	21438900	0.046879	-17.37500	10	3.25	20.625	Dnm2
chr7	141863914	141864232	0.046879	-17.33928	10	1.25	18.589	Muc5b
chr11	68892672	68892757	0.046879	-17.32738	10	0.625	17.952	Rnf222
chr4	155342159	155342742	0.046879	-17.27381	10	2.5476	19.821	Prkcz
chr19	58913794	58913947	0.046879	-17.1806	10	6	23.181	
chr10	95110823	95111124	0.046879	-16.9119	23	17.346	34.258	
chr17	32357884	32358529	0.046879	-16.85993	27	5.3755	22.235	Wiz
chr7	16907159	16907249	0.046879	-16.64087	10	7.4167	24.058	Ptgir
chr11	102018639	102018861	0.046879	-16.56903	16	3.0506	19.62	Mpp3
chr2	36242904	36244293	0.046879	-16.45600	29	3.5345	19.99	Ptgs1
chr11	79686522	79686656	0.046879	-16.24591	19	1.4035	17.649	Rab11fip4
chr5	110378002	110378250	0.046879	-16.15740	18	0.46296	16.62	Fbrsl1
chr19	5047812	5048006	0.046879	-15.96836	10	5.6515	21.62	Brms1
chr7	67693320	67693481	0.046879	-15.85813	16	8.75	24.608	Ttc23
chr17	34802733	34802978	0.046879	-15.83333	10	1.6667	17.5	Cyp21a1
chr10	76436437	76436499	0.046879	-15.72916	16	3.125	18.854	Pcnt
chr10	79974483	79974668	0.046879	-15.68121	18	4.9405	20.622	Grin3b
chr15	101373193	101373638	0.046879	-15.62418	22	3.0186	18.643	Gm35853
chr4	1.29E+08	1.29E+08	0.046879	-15.2014	14	3.6905	18.892	Phc2
chr11	97104151	97104499	0.046879	-15.10328	10	5.6029	20.706	Tbx21
chr7	24936601	24937001	0.046879	-14.88807	10	1.2879	16.176	Gm4881
chr17	31912548	31912761	0.046879	-14.6863	16	1.4967	16.183	
chr2	1.7E+08	1.7E+08	0.046879	-14.0357	10	1.6667	15.702	
chr2	169885906	169886059	0.046879	-13.93140	10	2.6786	16.61	Tshz2 & Gm26883
chr12	113161515	113161851	0.046879	-13.57034	10	3.5	17.07	Tedc1
chr5	122115066	122115370	0.046879	-13.20560	16	1.5625	14.768	Ccdc63
chr11	94667150	94667273	0.046879	-13.09127	10	5.1389	18.23	Xylt2
chr17	30744562	30744609	0.046879	-12.36111	10	7.1429	19.504	Dnah8
chr11	118886553	118886789	0.046879	-11.71266	10	2.5	14.213	Rbfox3
chr2	1.68E+08	1.68E+08	0.046886	-24.9645	19	1.4912	26.456	
chr7	45350068	45350821	0.047021	-33.3772	19	1.8045	35.182	Ppfia3
chr4	132204160	132204764	0.047021	-28.92272	29	1.4943	30.417	Ythdf2
chr13	35064370	35065211	0.047463	-29.15079	15	1.3333	30.484	Gm40910
chr2	29812311	29812617	0.04858	-29.58749	13	12.729	42.316	Slc27a4
chr4	1.48E+08	1.48E+08	0.04858	-22.5427	13	4.8504	27.393	

chr5	1.4E+08	1.4E+08	0.04858	-19.3619	31	5.5191	24.881	Ints1
chr14	31138856	31139038	0.048908	-33.49810	23	8.1884	41.687	Nt5dc2 & Stab1
chr5	135532253	13553259	0.049086	-13.83022	23	2.9348	16.765	Hip1
chr7	19394302	19394508	0.049102	-17.23612	17	5.0468	22.283	Ercc2 & Klc3
chr12	100301674	10030181	0.049188	-12.78873	13	1.9231	14.712	Gm10432 & Ttc7b
chr1	75624213	75624553	0.049255	-33.3392	13	3.007	36.346	
chr8	11124360	11124865	0.049666	-35.4579	13	2.8846	38.342	
chr17	29570382	29570789	0.049687	-23.14273	24	3.5764	26.719	Tbc1d22b & Gm28052
chr11	94911295	94911894	0.049714	-34.6154	13	0.9615	35.577	
chr9	47091928	47092568	0.049832	-30.50303	17	3.1489	33.652	Gm31698
chr1	39453913	39454128	0.049896	-24.25	15	2.8333	27.083	Tbc1d8
chr7	1.22E+08	1.22E+08	0.0499	-30.5495	13	8.0128	38.562	
chr2	1.56E+08	1.56E+08	0.049941	-30.9341	13	5.0366	35.971	Acss2 & Gss
chr9	1.08E+08	1.08E+08	0.049941	-21.004	15	5.9246	26.929	Camkv
chr1	1.41E+08	1.41E+08	0.049954	-33.6722	13	2.9487	36.621	

Identified 201 hypo-DMRs between ND and HFD fetal oocyte at E13.5.

dmr_ chrom	dmr_ start	dmr_ end	qval	diff	cpgs	mean_ ND	mean_ HFD	genes_ name
chr6	30738030	30738286	1.0656e-1	18.259804	51	19.894	1.634	Mest
chr7	126759632	12675972	0.0001117	68.055556	12	72.222	4.1667	Mapk3
chr16	11144062	11144141	0.0001482	5.628299	34	12.484	6.8553	Gm23935 & zinc finger CCCH type containing 7 A
chr11	51436536	51436656	0.0003618	54.259259	12	55.926	1.6667	Col23a1
chr3	1.12E+08	1.12E+08	0.000397	61.98413	15	65.833	3.8492	
chr9	75347883	75348030	0.0004754	18.974359	26	24.038	5.0641	Bcl2l10
chr5	36973835	36974044	0.0005755	34.144491	17	39.733	5.5882	Wfs1
chr17	10389591	10389632	0.00059	45.11905	21	58.214	13.095	
chr16	11144021	11144062	0.0008451	8.308586	10	15.861	7.5525	Zc3h7a
chr6	90905316	90905645	0.0009299	77.777778	15	79.444	1.6667	Iqsec1
chr8	121784949	12178503	0.0010365	26.091270	12	27.272	1.1806	Jph3 & Gm20388
chr19	4103309	4103482	0.001037	13.87756	16	14.919	1.0417	Pitpnm1
chr16	17734137	17734214	0.0010366	25.257937	12	26.925	1.6667	AC087802.4
chr5	122432655	12243278	0.0014756	37.458333	10	38.083	0.625	Anapc7
chr6	112722888	11272306	0.0016747	29.272727	15	29.828	0.55556	Srgap3
chrY	90761708	90761827	0.0016747	38.303999	19	48.163	9.8591	Mid1-ps1
chr1	191615720	19161592	0.0017613	42.070578	14	44.048	1.977	Ints7
chr5	142886491	14288671	0.0018528	13.082011	18	16.118	3.0357	Fbxl18
chr17	12741368	12741531	0.001889	23.39286	18	24.782	1.3889	Igf2r & Airn
chr17	23819922	23820109	0.0021919	29.180958	20	43.808	14.627	Srrm2
chr12	1.1E+08	1.1E+08	0.002497	12.15079	10	16.794	4.6429	
chr5	136100837	13610096	0.0024971	27.579365	10	28.413	0.83333	Rasa4
chr12	1.05E+08	1.05E+08	0.002497	28.83333	15	29.667	0.8333	Gm47648
chr13	59115430	59115665	0.0024971	31.282596	14	33.316	2.0337	Ntrk2
chrY	90742656	90742864	0.002639	22.11271	30	59.848	37.736	
chr7	127136262	12713643	0.0026416	30.051407	12	40.468	10.417	Spn
chrX	39694801	39694884	0.002642	49.36673	12	58.742	9.375	
chr9	75528426	75528720	0.0026496	31.005291	18	37.533	6.5278	Tmod3
chr16	93757138	93757295	0.002681	26.25676	12	29.729	3.4722	Dop1b
chr19	35584700	35585124	0.002681	45.87912	13	51.007	5.1282	
chr4	118212350	11821249	0.002685	24.214108	12	25.047	0.83333	Ptprf
chr10	126008428	12600860	0.0040315	27.669753	18	30.633	2.963	Lrig3
chr7	118757020	11875729	0.0042119	52.852564	13	66.667	13.814	Vps35l
chrX	58911421	58911672	0.0045488	24.083333	10	26.083	2	4930550L24Rik
chr1	1.61E+08	1.61E+08	0.004549	29.55646	10	33.396	3.8393	Rabgap1l
chr15	75657874	75657991	0.0045488	37.750000	10	43.083	5.3333	Top1mt
chr4	1.31E+08	1.31E+08	0.004549	61.08333	10	65	3.9167	
chr13	92215511	92215909	0.0046666	43.541667	12	44.375	0.83333	Msh3
chr13	119488292	11948839	0.0054923	24.116929	35	58.708	34.591	3110070M22Rik & Tmem267
chr8	109575826	10957594	0.0062583	35.000000	15	47.889	12.889	Hp
chr7	93347316	93347631	0.006505	46.00397	20	77.048	31.044	
chr16	23966102	23966225	0.006893	14.90873	10	19.909	5	Bcl6
chr18	38197779	38197906	0.0068931	19.941710	19	22.05	2.1084	Pcdh1
chr11	78839697	78839803	0.0068931	21.427300	12	26.636	5.2083	Lyrm9
chr8	1.02E+08	1.02E+08	0.006893	22.39796	14	27.16	4.7619	
chr2	75049878	75050061	0.006893	28.07143	10	28.905	0.8333	
chr1	1.58E+08	1.58E+08	0.006893	30.5	10	31.75	1.25	
chr4	109848632	10984886	0.0068931	32.374861	10	33.625	1.25	Faf1
chr18	78755354	78755448	0.0068931	32.896825	21	38.056	5.1587	Setbp1
chr10	80381665	80381712	0.0068931	33.482143	10	34.732	1.25	Mex3d
chr19	31723554	31723787	0.0068931	35.583333	10	48.083	12.5	Prkg1
chr9	114404639	11440483	0.0068931	36.246032	10	38.508	2.2619	Tmppe & Glb1

chr7	80345678	80345782	0.006893	37.1131	10	38.113	1	Hddc3 & Gm18310
chr17	56776922	56777029	0.0068931	37.750000	10	39.583	1.8333	Rfx2
chr1	52045752	52046014	0.0068931	45.833333	10	49.167	3.3333	Stat4
chr13	53157090	53157254	0.006893	46.546	10	53.897	7.3512	Ror2
chr17	29211475	29211756	0.006893	48.01984	10	49.448	1.4286	Cpne5
chr6	83402812	83402975	0.0068931	51.797619	10	53.464	1.6667	Tet3
chr4	43581458	43581549	0.006893	57.18865	13	66.163	8.9744	Rgp1
chr16	15279759	15279840	0.006893	58.75	10	71.25	12.5	
chr6	22436245	22436299	0.006893	66.34615	13	85.577	19.231	
chr4	65613384	65613620	0.007997	18.773313	25	21.931	3.1579	Trim32 & Astn2
chr1	87125674	87125786	0.010148	29.583333	14	37.44	7.8571	Akp3
chrY	90744457	90744578	0.010311	23.90796	24	59.257	35.349	
chr12	112174161	112174295	0.010423	23.163306	19	25.686	2.5231	Kif26a
chr16	20179545	20179834	0.011374	29.479167	16	30.521	1.0417	Yeats2
chr9	99794481	99794706	0.012128	45.02604	16	63.073	18.047	
chr9	66220574	66220704	0.012468	14.886905	10	16.137	1.25	Dapk2
chr17	6494241	6494302	0.012468	31.14484	10	55.524	24.379	
chr19	11026078	11026391	0.012468	32.87698	10	37.083	4.2063	
chr6	112605796	112606186	0.012468	47.083333	10	51.083	4	Gm5578
chr1	1.13E+08	1.13E+08	0.012468	50.39683	12	68.056	17.659	
chr7	125571668	125572149	0.012547	33.897516	23	34.933	1.0352	Gm44876 & interleukin 4 receptor, alpha
chr12	8447650	8448054	0.012832	25.75926	15	27.148	1.3889	
chr17	91088374	91088412	0.013204	14.758334	15	15.061	0.30303	Gm47307 & neurexin I
chr4	155289639	155289802	0.013236	12.821549	15	23.933	11.111	Prkcz
chr2	27267134	27267490	0.013678	21.723058	19	26.591	4.8684	Vav2
chr12	55395817	55395983	0.015348	22.418316	14	23.133	0.71429	Rpl18a-ps1 & Psma6
chr15	85507778	85507870	0.015361	18.96426	14	23.993	5.0283	
chr1	189850702	189850862	0.015361	27.453529	14	31.084	3.631	Ptpn14
chr4	155202154	155202429	0.017053	10.401991	13	14.889	4.4872	Ski
chr8	109994674	109994987	0.017477	22.640223	13	27.485	4.8443	Tat
chr11	1.09E+08	1.09E+08	0.01792	9.405466	19	14.699	5.2935	
chr15	78673208	78673340	0.01792	11.05898	10	12.684	1.625	Elfn2 & 1700041B01Rik
chr4	121092902	121093038	0.01792	16.547619	12	18.862	2.3148	Zmpste24
chr4	136946158	136946222	0.01792	18.710982	19	24.185	5.4745	Epha8
chr12	1.13E+08	1.13E+08	0.01792	18.72189	12	22.055	3.3333	Tedc1
chr9	95670102	95670210	0.01792	18.981481	12	22.222	3.2407	Pcolce2
chrX	169984861	169985058	0.01792	19.389748	21	43.639	24.249	Gm15726 & midline 1
chr8	1.2E+08	1.2E+08	0.01792	19.39153	12	20.433	1.0417	
chr10	69994293	69994592	0.01792	20.761905	10	28.262	7.5	Ank3
chr8	109724292	109724395	0.01792	21.547619	10	22.548	1	Zfp821
chr19	4489119	4489405	0.01792	24.88492	10	29.885	5	
chr3	66573534	66573632	0.01792	28.9881	10	32.917	3.9286	
chr11	53820906	53821004	0.01792	29.178241	12	31.51	2.3313	Gm12216
chr5	117349255	117349347	0.01792	29.189815	12	39.028	9.838	Gm15727 & Vsig10
chr14	52008395	52008455	0.01792	29.332168	10	40.166	10.833	Zfp219
chr7	11829739	11829917	0.01792	29.41138	12	33.925	4.5139	
chr7	141406680	141406853	0.01792	29.595238	10	35.595	6	Gm4535
chr19	58622510	58622682	0.01792	30.4881	10	34.363	3.875	1810007D17Rik
chr5	121434210	121434586	0.01792	30.742424	10	35.242	4.5	Naa25 & Erp29
chr9	79700350	79700458	0.01792	30.833333	10	32.083	1.25	Col12a1

chr6	50308227	50308370	0.01792	30.931818	10	38.182	7.25	Osbpl3
chr17	22230743	22230846	0.01792	32.47619	10	33.31	0.8333	
chr5	1.36E+08	1.36E+08	0.01792	33.77381	10	37.524	3.75	
chr6	143607450	143607631	0.01792	34.250000	10	35.917	1.6667	1700060C16Rik
chr3	38488269	38488530	0.01792	35.33333	10	36.583	1.25	
chr17	47012875	47013111	0.01792	35.7384	10	39.583	3.8449	
chr13	54999772	54999896	0.01792	37.116402	12	37.95	0.83333	Unc5a
chr5	135682297	135682598	0.01792	38.333333	10	39.583	1.25	Por
chr12	1.11E+08	1.11E+08	0.01792	40.875	10	41.875	1	Ppp2r5c
chr5	138093171	138093263	0.01792	46.773810	10	49.542	2.7679	Zkscan1
chr19	53537404	53537615	0.01792	47.107143	10	53.357	6.25	Dusp5
chr11	6242466	6242502	0.01792	49.666667	10	55.417	5.75	Gm11401
chr12	58586284	58586371	0.01792	50	12	54.861	4.8611	
chr7	125792153	125792208	0.01792	63.750000	10	85	21.25	D430042O09Rik
chr7	45795137	45795368	0.019567	32.770563	33	34.336	1.5657	Lmtk3
chr2	5379247	5379355	0.022262	10.014791	18	16.703	6.6878	Camk1d
chr15	85263282	85263367	0.022503	21.851852	18	26.435	4.5833	Fbln1
chr5	24589183	24589326	0.022503	24.789074	16	29.061	4.2722	Chpf2
chr8	29194170	29194296	0.023898	46.812835	13	57.596	10.783	Unc5d
chr9	40371635	40372053	0.023933	37.852564	13	39.135	1.2821	Gramd1b
chr5	1.14E+08	1.14E+08	0.026908	27.96876	17	34.132	6.1629	Ung
chr17	46540213	46540328	0.027754	16.133478	15	17.14	1.0067	Gm26904 & cullin 9
chr7	102239702	102239923	0.027754	20.920635	15	29.524	8.6032	Rhog
chr7	44462054	44462131	0.031941	12.746337	10	13.964	1.2179	Lrrc4b
chr17	35682322	35682497	0.031941	15.154585	24	20.746	5.5913	Ddr1
chr13	119472048	119472667	0.031941	18.069728	14	18.963	0.89286	4833420G17Rik
chr1	1.33E+08	1.33E+08	0.031941	18.2408	10	25.741	7.5	
chr16	20525922	20525961	0.031941	26.854798	10	31	4.1452	Dvl3
chr11	64094399	64094555	0.031941	30.83333	10	33.5	2.6667	
chr2	118615469	118615570	0.031941	30.935374	14	35.06	4.1241	Bub1b
chr16	93919225	93919357	0.031941	43.02083	20	45.53	2.5089	Cldn14
chrX	15796117	15796264	0.031941	46.05952	10	68.054	21.994	
chr5	76928549	76928679	0.031941	51.500000	10	56.667	5.1667	Ppat & 2310040G07Rik
chr9	16606605	16606960	0.032717	42.14286	21	54.524	12.381	
chr6	147107846	147108092	0.033678	42.467758	16	48.703	6.2351	Klhl42
chr2	174345184	174345496	0.034138	30.418803	25	37.318	6.8992	Gm20721 & GNAS (guanine nucleotide binding protein, alpha stimulating) complex locus
chr6	30637880	30638421	0.037846	33.68437	13	35.928	2.2436	
chr10	1.05E+08	1.05E+08	0.038011	38.46154	13	53.205	14.744	
chr3	95874417	95874634	0.038038	31.346154	13	39.423	8.0769	C920021L13Rik
chr1	54025553	54025641	0.040779	34.623016	12	59.861	25.238	Hecw2
chr1	60900875	60901156	0.04167	18.382353	17	21.324	2.9412	Rpl18-ps1 & Ctla4
chrX	1.12E+08	1.12E+08	0.041981	62.42064	12	79.861	17.44	
chr6	84593710	84593914	0.042235	17.772817	12	20.451	2.6786	Cyp26b1
chr6	50378499	50378811	0.042518	21.839451	12	23.315	1.4757	Osbpl3
chr6	55377651	55378151	0.042953	10.425084	12	12.508	2.0833	Ghrhr
chr7	121118156	121118713	0.042953	15.924272	12	16.445	0.52083	Otoa
chr7	19944635	19944774	0.042953	18.561508	12	20.645	2.0833	Igsf23
chr4	141952097	141952229	0.042953	20.157077	12	23.522	3.3647	Fhad1
chrX	169980904	169981077	0.042953	23.779668	18	49.326	25.546	Gm15726 & midline 1
chr9	94590182	94590361	0.042953	25.34722	12	30.208	4.8611	

chr10	1.11E+08	1.11E+08	0.042953	25.35053	12	26.739	1.3889	
chr13	38930423	38930587	0.042953	31.35417	12	32.917	1.5625	
chr3	90920955	90921032	0.042953	32.98611	12	37.153	4.1667	
chr7	30719053	30719197	0.042953	36.064815	12	36.991	0.92593	Atp4a
chr15	78777964	78778182	0.042953	37.501653	12	44.329	6.8271	Card10
chr10	1.27E+08	1.27E+08	0.042953	37.97619	12	40.06	2.0833	
chr11	101336510	101336575	0.042953	51.736111	12	53.819	2.0833	Aoc3
chr15	89304997	89305239	0.046229	19.683812	18	26.171	6.4868	Sbf1
chr4	149929935	149930274	0.046879	10.305603	11	11.564	1.2587	Spsb1
chr16	23431150	23431262	0.046879	12.034701	10	13.518	1.4835	Rtp1
chr6	82728109	82728445	0.046879	12.87915	10	17.212	4.3333	Hk2
chr1	89145604	89145790	0.046879	14.101190	10	15.768	1.6667	Sh3bp4
chr15	12926095	12926158	0.046879	16.76786	10	17.601	0.8333	Drosha
chr1	87719523	87719644	0.046879	17.178571	10	18.429	1.25	Inpp5d
chr10	56504724	56504776	0.046879	17.47732	14	27.299	9.8214	
chr4	141051080	141051118	0.046879	18.011905	10	20.44	2.4286	Crocc
chr7	46614365	46614545	0.046879	18.273810	10	21.607	3.3333	Sergef
chr10	43510957	43511129	0.046879	18.5464	10	19.546	1	Bend3
chr8	121558343	121558601	0.046879	18.655247	10	21.084	2.4286	Fbxo31 & Gm20388
chr1	36533053	36533316	0.046879	20.934524	10	25.935	5	Ankrd23 & Gm42417
chr10	61229676	61229770	0.046879	21.729978	10	27.333	5.6034	Adamts14
chr15	8722410	8722766	0.046879	23.38095	10	25.762	2.381	
chr13	73494642	73495139	0.046879	23.461039	10	25.128	1.6667	Lpcat1
chrY	90741280	90741326	0.046879	25.06533	14	76.799	51.734	
chr2	76736464	76736529	0.046879	25.521825	10	30.522	5	Gm13938 & titin
chr8	3635654	3635730	0.046879	26.04167	10	29.345	3.3036	Stxbp2
chr8	15039641	15039767	0.046879	27.22931	14	35.843	8.6139	
chr2	31952347	31952650	0.046879	27.924242	10	33	5.0758	Aif1l
chr11	119997003	119997143	0.046879	28.422619	10	30.089	1.6667	Baiap2
chr3	91335121	91335348	0.046879	29.7803	10	32.28	2.5	
chr14	58893566	58893636	0.046879	30.380952	10	38	7.619	Rpl13-ps3
chr5	1.06E+08	1.06E+08	0.046879	32.3682	14	33.559	1.1905	
chr13	52508062	52508264	0.046879	33.596781	18	38.662	5.065	Diras2
chrX	92120578	92120808	0.046879	33.781746	10	35.448	1.6667	Mageb18
chr1	189862943	189863082	0.046879	35.750000	10	37.75	2	Ptpn14
chr17	82959412	82959607	0.046879	35.79167	10	39.792	4	
chr7	26283902	26284024	0.046879	37.19183	10	52.608	15.417	
chr7	46267455	46267595	0.046879	37.293651	10	41.27	3.9762	Otog
chr2	1.54E+08	1.54E+08	0.046879	37.70833	10	39.167	1.4583	Dnmt3b
chr10	44140858	44140940	0.046879	38.517857	10	53.304	14.786	Crybg1
chr7	1.13E+08	1.13E+08	0.046879	39.17857	10	53.75	14.571	
chr10	116788872	116788996	0.046879	39.880952	11	58.333	18.452	Myrfl
chr17	28837448	28837495	0.046879	40.333333	10	42.833	2.5	Brpf3
chr1	131181173	131181403	0.046879	41.411565	14	44.643	3.2313	Eif2d & Rassf5
chr2	60979404	60979812	0.046879	44.91667	10	49.917	5	
chr2	118123481	118123850	0.046879	47.166667	10	49.667	2.5	Gm29233 & thrombospondin
chr6	25988593	25988948	0.046879	50.42411	16	73.914	23.49	
chr10	19985422	19985629	0.046879	56.000000	10	67.5	11.5	Map3k5
chr12	9805777	9805928	0.046879	60	10	67.5	7.5	

Identified 63 hyper-DMRs between ND and HFD fetal oocyte at E18.5.

dmr_ chrom	dmr_ start	dmr_ end	qval	diff	cpgs	mean_ ND	mean_ HFD	genes_ name
chr5	112307800	11230794[illegible]	4.2391e-0	-24.05856	31	0.5914	24.65	Tpst2
chr4	149898145	14989845[illegible]	1.6005e-0	-45.52404	17	0.3268	45.851	Spsb1
chr2	30256021	30256383	3.4139e-0	-22.53607	25	1.8889	24.425	Lrrc8a &
chr15	81783006	81783309	4.1586e-0	-36.02460	23	2.1014	38.126	Zc3h7b
chr5	112333032	11233319[illegible]	0.0004092	-49.37376	16	4.5176	53.891	Tfip11
chr13	56533911	56534329	0.0004092	-35.77740	14	0.5102	36.288	Fbxl21
chr13	47158722	47158855	0.0006533	-44.95879	13	0.64103	45.6	Rnf144b
chr16	93754972	93755511	0.0006649	-18.48785	18	0.70547	19.193	Dop1b
chr12	14721507	14722069	0.002375	-43.4804	17	6.8627	50.343	
chrX	140600455	14060057[illegible]	0.0025446	-34.87484	13	0.64103	35.516	Tsc22d3
chr11	35708004	35708177	0.004994	-23.46088	28	9.8214	33.282	Slit3
chr5	8043075	8043153	0.0050184	-86.75000	10	3.25	90	Gm15731
chr13	38941614	38941639	0.0050184	-71.66666	10	2.5	74.167	Slc35b3
chr5	114069631	11406971[illegible]	0.0050184	-50.58333	10	9.3333	59.917	Usp30 & Svop
chr17	25718779	25719804	0.0051199	-41.08098	27	3.5384	44.619	Gng13 & Chtf18
chr1	36103801	36104108	0.0053939	-40.92460	30	0.51852	41.443	Hs6st1
chr4	107040986	10704119[illegible]	0.0059865	-60.76388	12	4.1667	64.931	Ssbp3
chr12	66284270	66284360	0.0059865	-60.69444	12	1.4583	62.153	Rpl10l
chr5	63924533	63924957	0.0059865	-25.96813	15	1.1111	27.079	Gm15819 & RELT-like 1
chr15	103248824	10324893[illegible]	0.0088244	-41.47875	17	0.3268	41.806	Nfe2
chr10	76603682	76603845	0.0090343	-30.20220	10	2.1212	32.323	Col6a2
chr6	143221779	14322203[illegible]	0.0098505	-59.82142	12	0.59524	60.417	Etnk1
chr15	78940164	78940594	0.011702	-29.42734	23	0.36232	29.79	Nol12
chr5	140718105	14071839[illegible]	0.012013	-15.97332	18	2.3644	18.338	Brat1
chr8	75097028	75097544	0.013966	-29.98366	17	0.98039	30.964	Hmox1
chr11	113562688	11356278[illegible]	0.01551	-54.88888	10	1.25	56.139	Slc39a11
chr5	105866599	10586674[illegible]	0.01551	-51.83333	10	2.5	54.333	Gm43818
chr8	94346116	94346189	0.01551	-50.42857	10	1.6667	52.095	Slc12a3
chr7	45047017	45047144	0.01551	-45.64285	10	1.25	46.893	Prr12
chr17	66377909	66377968	0.01551	-45.61904	10	2.381	48	Mtcl1
chr17	35670065	35670347	0.01551	-30.86904	15	0.66667	31.536	Gtf2h4
chr12	71318609	71318774	0.01551	-20.32936	15	1.7262	22.056	Dact1 & 4930474H06Rik
chr19	53843093	53843558	0.01551	-18.06842	35	1.958	20.026	Rbm20
chr16	34243242	34243833	0.016545	-28.30357	14	0.44643	28.75	Kalrn
chr11	81397581	81397614	0.016763	-51.73611	12	16.667	68.403	4930527B05Rik & Asic2
chr7	105384224	10538434[illegible]	0.016763	-38.58974	13	1.2821	39.872	Fam160a2
chr2	4549247	4549555	0.016763	-22.99007	20	1.131	24.121	Frmd4a
chr5	125427324	12542771[illegible]	0.021184	-28.21180	16	2.6228	30.835	Dhx37
chr17	46726219	46726422	0.025446	-16.46935	18	0.55556	17.025	Gnmt & Gm26904
chr7	118434157	11843441[illegible]	0.027301	-27.47549	17	2.6961	30.172	Syt17
chr5	38398677	38398694	0.028563	-72.91666	10	15.833	88.75	Slc2a9
chr5	137300341	13730045[illegible]	0.028563	-51.64881	10	1.4583	53.107	Srrt
chr15	76186395	76186859	0.042104	-37.38888	15	1.9444	39.333	Plec
chr9	110386909	11038707[illegible]	0.0427	-21.63359	15	0.55556	22.189	Ptpn23
chr7	4856711	4856986	0.048657	-68.05555	12	2.0833	70.139	Isoc2b
chr5	1.34E+08	1.34E+08	0.048657	-61.0595	10	0.8333	61.893	
chr17	64145602	64145787	0.048657	-49.8333	10	2.5	52.333	
chr2	70902957	70903421	0.048657	-46.6987	13	0.4808	47.179	
chr11	65187502	65187579	0.048657	-44.70072	12	9.1187	53.819	Myocd
chr15	59024025	59024212	0.048657	-40.60317	10	0.83333	41.437	Mtss1
chr10	80382279	80382379	0.048657	-37.45518	10	1.25	38.705	Mex3d & Gm22721

chr6	115865784	11586591[illegible]	0.048657	-36.40664	19	1.5789	37.986	Ift122
chr9	120174289	12017433[illegible]	0.048657	-35.00000	10	5	40	Mobp
chr11	69345522	69345583	0.048657	-32.98992	12	2.4267	35.417	Chd3
chr11	96550154	96550651	0.048657	-32.70833	12	1.3889	34.097	Skap1
chr15	77796103	77796323	0.048657	-31.72805	10	1.25	32.978	Myh9
chr11	116221536	11622166[illegible]	0.048657	-31.25000	12	0.83333	32.083	Evpl
chr11	77843125	77843230	0.048657	-27.67857	10	0.71429	28.393	Myo18a
chr2	32563494	32563686	0.048657	-27.01326	13	2.8846	29.898	Fam102a
chr2	85058713	85058808	0.048657	-26.18668	10	0.625	26.812	Tnks1bp1
chr18	61524370	61524841	0.048657	-23.95502	12	1.3889	25.344	Arhgef37
chr19	41086236	41086392	0.048657	-21.70634	10	0.83333	22.54	Tll2
chr7	12061327	12061443	0.048962	-54.3074	11	36.223	90.53	

Identified 28 hypo-DMRs between ND and HFD fetal oocyte at E18.5.

dmr_ chrom	dmr_ start	dmr_ end	qval	diff	cpgs	mean_ ND	mean_ HFD	genes_name
chr17	39844829	39845149	1.6207e-1	11.167016	78	22.732	11.565	Gm26917
chr17	39845726	39845981	3.6753e-0	10.793253	73	19.706	8.9125	Gm26917
chrM	229	2281	8.2622e-0	26.735958	87	62.799	36.063	mt-Rnr1 & mt-Tv & mt-Rnr2
chrM	2408	4094	1.6748e-0	20.043559	86	62.255	42.211	mt-Rnr2 & mt-Tl1 & mt-Nd1 & mt-Ti & mt-Tq & mt-Tm & mt-Nd2
chr17	39844152	39844298	0.0005622	12.065972	37	18.949	6.8831	Gm26917
chr17	39848445	39848622	0.0023525	7.479594	56	13.822	6.3429	CT010467.1 & Gm42418
chr11	102153366	10215345[illegible]	0.0029146	42.595238	10	52.012	9.4167	Tmem101
chr17	39845481	39845627	0.0039733	5.580028	44	15.216	9.6359	Gm26917
chr5	73552784	73553200	0.0048859	48.058608	13	56.52	8.4615	Dcun1d4
chr17	39843647	39843916	0.004994	5.144823	38	13.729	8.5846	Gm26917
chr17	12943156	12943398	0.0050184	35.583333	10	37.25	1.6667	Acat2
chr13	55672161	55672219	0.0050184	66.750000	10	69.25	2.5	Ddx46
chr1	1.32E+08	1.32E+08	0.005987	29.67442	14	30.567	0.8929	
chrM	5339	5888	0.0086977	30.542879	17	55.974	25.431	mt-Co1
chrM	5902	6114	0.0090343	35.745277	10	62.734	26.989	mt-Co1
chr14	1.14E+08	1.14E+08	0.01372	30.62325	17	32.584	1.9608	
chrX	123103476	12310364[illegible]	0.01551	17.704425	15	21.593	3.8889	Cldn34c1
chr3	8805733	8805874	0.01551	50.148810	10	55.149	5	Mrps28
chr16	93690404	93690637	0.016763	23.625992	12	25.293	1.6667	Cbr3
chr18	37006784	37006932	0.016763	25.937500	12	27.326	1.3889	Pcdha1 & Pcdha2 & Pcdha3 & Pcdha4 & Pcdha5 & Pcdha6 & Pcdha7 & Pcdha8 & Pcdha9 & Pcdha11 & Gm42416 & Gm37013 & Gm37388
chrM	12086	13345	0.020929	16.766969	60	72.376	55.609	mt-Nd5
chr17	39848641	39848762	0.022804	10.631959	33	18.81	8.1785	CT010467.1 & Gm42418
chr16	57391451	57391592	0.025446	7.029938	36	15.731	8.7014	Cmss1 & Filip1l
chr8	122497547	12249787[illegible]	0.027301	26.907890	22	29.815	2.9072	Piezo1 & Gm20388
chr10	13892797	13893677	0.031795	20.92648	29	33.275	12.348	
chrM	6130	6432	0.033356	27.171379	16	58.087	30.915	mt-Co1
chr15	97758852	97758974	0.048657	20.079365	12	21.468	1.3889	Rapgef3
chr10	112425216	11242534[illegible]	0.048657	39.849206	10	40.349	0.5	Kcnc2

Table S5

Primer sequences of genes for Bisulfite sequencing
F1: outer forward; R1: outer reverse; F2: inner forward; R2: inner reverse

Gene	Primer sequence	Size（bp）
Brat1	F1: 5'–GTGTTATTTTTTTGGTTGGGTG–3'	455
	R1: 5'–ACCAACTCCAAACCCTACA–3'	
	F2: 5'–TGGTAGGAGTTTGAGGTAGTTG–3'	425
	R2: 5'–ACCAACTCCAAACCCTACA–3'	
Sohlh2	F1: 5'–TGGAGAAAGTAAAATTGTTGGT–3'	327
	R1: 5'–CCCCAAACCCTTAATAAAAC–3'	
	F2: 5'–TGGAGAAAGTAAAATTGTTGGT–3'	288
	R2: 5'–TCCCTCCAAATCCTACCTAC–3'	
Kmt2d	F1: 5' –ATAGTTGAGAAGGAGGAATTTA–3'	353
	R1: 5'– ACAATTTATACACTCCAAATCA–3'	
	F2: 5' –GAGAAGGAGGAATTTAGGTTAGT–3'	251
	R2: 5'–ACACTCCAAATCATCTCAATAC–3'	
Ddx4	F1: 5'–AGATAGGATGTTGAGGTTTTGG–3'	370
	R1: 5'–TCCTCTACCTTCCAAACCTAAC–3'	
	F2: 5' –AATTGTTTTGAGTYGGGTTT–3'	326
	R2: 5'–TCTACCTTCCAAACCTAACCC–3'	
Mest	F1: 5'–GGTATTAATATATGGGAAGGTT–3'	366
	R1: 5'–CAAAAACAACAAACAACAA–3'	
	F2: 5'–TTTTTTTATTAGAATTTGGGGT–3'	345
	R2: 5'–CAAAAACAACAAACAACAACT–3'	
Igf2r	F1: 5'–GGTTTTAGGTTGGAGTAAGAGG–3'	344
	R1: 5'–TAAAACCCTTCCCTCCTATAAA–3'	
	F2: 5'–GAGTTAAAGTTTGGTGAGGTTG–3'	320
	R2: 5'–TAAAACCCTTCCCTCCTATAAA–3'	
Kmt2b	F1: 5'–GGGGGTTGGTTGTTATTAGTTA–3'	299
	R1: 5'–AATTCCCTTACTAAAACTCCCC–3'	
	F2: 5'–GGTGTTGAAGGAAGAGAGTTTT–3'	272
	R2: 5'–AATTCCCTTACTAAAACTCCCC–3'	
Usp30	F1: 5'–TGGTTTTTTTATTTGAATGTGT–3'	352
	R1: 5'–CCAAACCTAATAACCTAAATTCA–3'	

	F2: 5’–AGGTTTTTTGTTGATGTGTTTT–3’<br>R2: 5’–CCAAACCTAATAACCTAAATTCAA–3’	313
